# Angiosperm-Wide and Family-Level Analyses of *AP2*/*ERF* Genes Reveal Differential Retention and Sequence Divergence After Whole-Genome Duplication

**DOI:** 10.3389/fpls.2019.00196

**Published:** 2019-02-26

**Authors:** Linbo Wang, Hong Ma, Juan Lin

**Affiliations:** ^1^State Key Laboratory of Genetic Engineering and Ministry of Education Key Laboratory of Biodiversity Sciences and Ecological Engineering, Collaborative Innovation Center for Genetics and Development, Institute of Plant Biology, Institute of Biodiversity Sciences, School of Life Sciences, Fudan University, Shanghai, China; ^2^Department of Biology, Huck Institutes of the Life Sciences, Pennsylvania State University, University Park, PA, United States

**Keywords:** *AP2/ERF* genes, gene duplication, preferential retention, functional divergence, stress response, angiosperm evolution, ecological niche

## Abstract

Plants are immobile and often face stressful environmental conditions, prompting the evolution of genes regulating environmental responses. Such evolution is achieved largely through gene duplication and subsequent divergence. One of the most important gene families involved in regulating plant environmental responses and development is the *AP2/ERF* superfamily; however, the evolutionary history of these genes is unclear across angiosperms and in major angiosperm families adapted to various ecological niches. Specifically, the impact on gene copy number of whole-genome duplication events occurring around the time of the origins of several plant families is unknown. Here, we present the first angiosperm-wide comparative study of *AP2/ERF* genes, identifying 75 Angiosperm OrthoGroups (AOGs), each derived from an ancestral angiosperm gene copy. Among these AOGs, 21 retain duplicates with increased copy number in many angiosperm lineages, while the remaining 54 AOGs tend to maintain low copy number. Further analyses of multiple species in the Brassicaceae family indicated that family-specific duplicates experienced differential selective pressures in coding regions, with some paralogs showing signs of positive selection. Further, *cis* regulatory elements also exhibit extensive divergence between duplicates in *Arabidopsis*. Moreover, comparison of expression levels suggested that *AP2/ERF* genes with frequently retained duplicates are enriched for broad expression patterns, offering increased opportunities for functional diversification via changes in expression patterns, and providing a mechanism for repeated duplicate retention in some AOGs. Our results represent the most comprehensive evolutionary history of the *AP2/ERF* gene family, and support the hypothesis that *AP2/ERF* genes with broader expression patterns are more likely to be retained as duplicates than those with narrower expression profiles, which could lead to a higher chance of duplicate gene subfunctionalization. The greater tendency of some AOGs to retain duplicates, allowing expression and functional divergence, may facilitate the evolution of complex signaling networks in response to new environmental conditions.

## Introduction

Plants face numerous environmental stresses, including salinity, temperature extremes, toxicity, pathogen attack, and wounding. Such environmental conditions constrain plant development and productivity, thereby affecting their geographical distribution and limiting their economic value and ecological impact (Zou et al., 2009). How plants cope with abiotic stress conditions is a particularly intriguing topic, given the extraordinary diversity of habitats and ecological niches that plants have colonized, spanning extremely arid, hot, and cold environments on land, as well as marine, brackish, and freshwater aquatic habitats. Colonization of these habitats would have imposed novel challenges and requirements for genes responsive to abiotic stressors. That is, diverse ecological niches present varying evolutionary pressures on plant genes, particularly those responsive to the stresses. To survive and reproduce in various ecological niches, plants have evolved multiple adaptive traits governed by complex signaling and regulatory systems, with downstream networks regulated by transcription factors (TFs) (Licausi et al., 2013; Phukan et al., 2017). For example, the AP2/ERF family of plant TFs has important roles in regulating responses to environmental stressors, growth and development, and metabolism (Licausi et al., 2013; Phukan et al., 2017; Supplementary Table S1).

Plant *AP2/ERF* genes are members of a superfamily comprising several families, namely the *AP2*, *DREB/ERF*, *RAV*, and Soloist families, as defined previously (Sakuma et al., 2002; Kim et al., 2006; Nakano et al., 2006; Zumajo-Cardona and Pabón-Mora, 2016). The *DREB/ERF* family can be further subdivided into the *DREB* and *ERF* subfamilies, each of which has several subgroups, referred to as Groups I–IV and Groups V–X, respectively (Nakano et al., 2006). For simplicity, hereafter we will use *DREB* or *ERF* “family” for the respective groups collectively and “subfamily” to refer to these groups individually. Proteins in the AP2 family contain two AP2 domains, whereas members of the other families have only one AP2 domain (Nakano et al., 2006). Many AP2/ERF family members have known functions in plant development and stress responses (Licausi et al., 2013; Phukan et al., 2017). *AP2* family genes also control floral organ identity (*AP2*), and embryo development and flowering time (*TOE1*) (Licausi et al., 2013; Zhang et al., 2015; Phukan et al., 2017). *DREB* genes are crucial for responses to abiotic stressors, including drought (*DREB2*) (Krishnaswamy et al., 2011), high salt (*ESE*) (Zhang et al., 2011), low temperature (*DREB1*/*CBFs*) (Jia et al., 2016; Zhou et al., 2017), wounding (*WIND*) (Iwase et al., 2016), and floral organ senescence (*FUF1*) (Chen et al., 2015). *ERF* members participate in pathogen responses (Licausi et al., 2013; Phukan et al., 2017), water availability (*SNORKEL1* and *SNORKEL2*) (Hattori et al., 2009), root and shoot growth (*TINY*) (Wilson, 1996; Wei et al., 2005), low oxygen responses (*RAP2.12*, *RAP2.2*, *AtRAP2.3*, *AtHRE1*, *AtHRE2*) (Licausi et al., 2010, 2011; Weits et al., 2014; Bui et al., 2015; Kosmacz et al., 2015), and floral meristem identity (*PUCHI*, *DRN*, *DRNL*) (Chandler and Werr, 2017). *RAV* family members have roles in regulating hormone and stress responses, independent of ABA (Fu et al., 2014).

In plants, gene duplication is widespread and often results from whole-genome duplication (WGD) (Jiao et al., 2011, 2012; Vanneste et al., 2014; Huang et al., 2016b; Xiang et al., 2016; Ren et al., 2018), providing raw genetic materials for functional innovations, including regulation of development and physiology and contributing to the success of plants under different environmental conditions (Li et al., 2016; Panchy et al., 2016). Duplication of the *AP2/ERF* genes has been investigated in phylogenetic studies with limited sampling of angiosperms, mostly restricted to core eudicot and monocot models, or single species (Song et al., 2013; Cui et al., 2016; Dossa et al., 2016; Zeng et al., 2016; Liu and Zhang, 2017; Li et al., 2018). Some studies have focused on a specific family or subclade of genes [i.e., the *AP2* family (Rashotte and Goertzen, 2010; Zumajo-Cardona and Pabón-Mora, 2016)]. Thus, the patterns of *AP2/ERF* gene retention following WGD across angiosperms remain unclear, as do the phylogenetic relationships among *AP2/ERF* genes of multiple members of the same plant family. Specifically, it is not clear when and where *AP2/ERF* duplicates were retained in angiosperm history, or whether the same duplicate(s) generated in an ancestor tend to be retained in multiple species adapted to different ecological niches, or retained in some species but lost in others. Further, how duplication and divergence of *AP2/ERF* genes contribute to the evolution of stress response pathways is also not well understood. The patterns of retention and loss of *AP2/ERF* duplicates have the potential to provide insights into the evolution of regulatory genes for plant development and environmental responses more generally (LehtiShiu et al., 2015).

It has been proposed that speciation in plants involves rapid evolution of stress response genes (Zou et al., 2009). *AP2/ERF* duplicates are associated with environmental adaptation, including habitat colonization. For example, natural variation in the *DREB* family members, C-repeat-binding factor (*CBF*) genes, among populations of the model plant, *Arabidopsis thaliana*, along the Yangtze River in China, is the molecular basis of divergence in freezing tolerance (Kang et al., 2013); however, it is unclear whether *AP2/ERF* genes are associated with adaptation in other plants. Relatives of *A. thaliana* in the Brassicaceae, *Eutrema salsugineum* (Yang et al., 2013) and *Schrenkiella parvula* (Dassanayake et al., 2011), are tolerant of alkaline soils, but it is unknown whether this is associated with lineage-specific expansions of *AP2/ERF* genes. The species *Medicago truncatula*, *Ananas comosus*, *Zea mays*, *Sorghum bicolor*, and *Setaria italica* are drought-tolerant. This property and the phylogenetic placements among angiosperms provide great opportunities for investigation of the evolution of *AP2/ERF* genes from an ecological perspective. Among angiosperms with sequenced genomes, three, the sacred lotus (*Nelumbo nucifera*) (Ming et al., 2013), duckweed (*Spirodela polyrhiza*) (Wang et al., 2014), and seagrass (*Zostera marina*) (Olsen et al., 2016), are aquatic, and have independently colonized their habitats. As *AP2/ERF*s are involved in plant responses to water availability, examination of the evolutionary patterns of *AP2/ERF* genes in these parallel lineages would be a worthwhile exercise.

To better understand the diversity and evolution of the *AP2/ERF* superfamily and to obtain clues about their functional evolution and their potential contributions to the radiations of angiosperms, and different angiosperm groups in various habitats, we performed comprehensive analyses of *AP2/ERF* genes in 37 species, covering the major lineages of angiosperms. First, we detailed the initial history of *AP2/ERF* gene duplication in representative major angiosperm groups, identifying multiple duplications in the core eudicots, Brassicaceae, Fabaceae, Asteraceae, Commelinids, Poales, and Poaceae, and differential expansion of the *AP2*/*ERF* family. Next, we identified preferential retention of recent *AP2/ERF* gene duplicates that had undergone previous duplication and/or exhibited broad expression. Then, we examined patterns of sequence divergence and evidence of positive selection, and found that disordered protein regions evolved more rapidly than those that were ordered. Finally, we discuss experimental support from the literature for a strong correlation between duplicate retention, resulting in expanded *AP2*/*ERF* Angiosperm OrthoGroups (AOGs) and broad expression patterns, allowing subfunctionalization in expression patterns. This study provides rich information supporting hypotheses on how *AP2*/*ERF* genes have expanded and evolved during angiosperm history through adaptation to different ecological niches.

## Materials and Methods

### Data Collection

The genome sequences of 38 species were downloaded from public databases, 32 species (including the outgoup, *Selaginella moellendorffii*) from Phytozome v12.0 (Goodstein et al., 2012)^1^; three species, *Aethionema arabicum*, *Leavenworthia alabamica*, and *Sisymbrium irio* (Rosenbloom et al., 2015) using the University of California at Santa Cruz Genome Browser^2^; *S. parvula*^3^ (Dassanayake et al., 2011); *N. nucifera* from LOTUS-DB^4^ (Ming et al., 2013); and *Helianthus annuus* from the Genome Database^5^ (Badouin et al., 2017). Species information is detailed in Supplementary Table S2.

### Sequence Retrieval and Identification

For this study, *AP2/ERF* genes were identified using HMMER v3.1^6^ (Finn et al., 2011) using the AP2 entry [A profile hidden Markov model (HMM) from the seed alignment] (Pfam accession number PF00847) of Pfam-A, downloaded from Pfam v27.0^7^ (Finn et al., 2016), and named with a four-letter species designations, including the first two letters of the genus and the species (Supplementary Table S3). All retrieved protein sequences containing the AP2 domain were verified by a batch search of the NCBI Conserved Domain Database^8^ (Marchler-Bauer et al., 2015), with a threshold of *e*-value < 10^-5^; sequences lacking the AP2 domain were discarded before further analyses.

### Phylogenetic Analysis

We employed OrthoFinder (Emms and Kelly, 2015) to infer AOGs, each with one copy in the most recent common ancestor (MRCA) of angiosperms, for AP2/ERF proteins. Next, protein sequences from each AOG were aligned using Multiple Sequence Alignment (MSA) in Fast Fourier Transform (MAFFT) v7.273 (Katoh and Standley, 2013), and regions of low quality were automatically removed using trimAl v1.4 (Capella-Gutiérrez et al., 2009) with default settings. Then, a preliminary approximate maximum-likelihood (ML) phylogenetic tree was generated based on the alignments, using FastTree v2.1.7 (Price et al., 2009). If a tree indicated a duplication event at a node before the MRCA of angiosperms, we split it into two subtrees to ensure that each subtree represented a single ancestral angiosperm gene and its descendants. A second round of MSA was performed for each AOG separately. To improve the alignment of relatively variable N-terminal and C-terminal regions, the “–localpair” and “–maxiterrate 1000” commands were used. As AP2/ERF sequences often lacked sufficient phylogenetic information to yield well-supported gene tree topologies, thereby affecting subsequent analyses, we incorporated a gene tree improvement procedure using TreeFix (Wu et al., 2013), a gene tree–species tree reconciliation algorithm that performs topological changes to gene trees and searches for alternative topologies that minimize the duplication/loss cost, while having a likelihood not statistically significantly worse than that of the ML topology. Briefly, ML trees were first constructed for the AOGs using IQ-TREE (Nguyen et al., 2015), based on trimmed protein sequence alignments previously obtained using the amino acid substitution (LG+F+G) model (Le and Gascuel, 2008). The trees were rooted arbitrarily, as rooting is needed for TreeFix, whereas the specific position of the root is not important. Subsequently, using a reference species tree (Zeng et al., 2014, 2017; Huang et al., 2016a), a file was generated that mapped leaves of the gene tree onto those of the species tree. In addition, codon-based CDS (DNA sequence) alignments, generated from the amino acid sequence alignments, were back translated using TreeBeST v1.9.2 (Vilella et al., 2009) and used for analysis with TreeFix, under the default reconciliation model (duplication/loss cost), with rooted gene trees to generate reconstructed gene trees, increasing the number of iterations to 1000. Furthermore, branch lengths and SH-aLRT support for the reconstructed gene trees generated by TreeFix (Wu et al., 2013) were computed using IQ-TREE v.1.5.5, with the best-fit codon model, as determined using ModelFinder (Kalyaanamoorthy et al., 2017).

### Evolutionary Analysis of Brassicaceae Genes

Paralogous pairs of Brassicaceae-specific duplicates and their orthologs in *Theobroma cacao* and *Carica papaya* were aligned using PRANK v140603 (Veidenberg et al., 2016) and back translated using TreeBeST v1.9.2 (Vilella et al., 2009). Gene trees reconciled with the species tree were generated using the same pipeline described in the section “Phylogenetic analysis.” To detect sequence differences, as evidence for relaxed selection or positive selection, the gene tree and codon-based alignment were imported into RELAX^9^ (Wertheim et al., 2015), to detect whether selective strength was relaxed in post-duplication branches relative to their pre-duplication branches, and codeml implemented in the program to estimate non-synonymous (Ka) to synonymous (Ks) substitutions rates (ω = Ka/Ks) values and test for positive selection using the PAML 4.8 package (Yang, 2007).

To detect different selective pressures that affect specific lineages, we compared the two-ratios model (Model 2), which allows a different ω ratio for foreground lineages, with the one-ratio model, which assumes that the ω ratio is constrained along all branches in the phylogeny (one-ratio Model 0) (Yang, 1998; Yang et al., 1998). The positive selection that affected some sites in the post-duplication branches was detected by comparing the branch-site model (Model A), assuming one class of sites in the foreground lineage, ω > 1, with null model A (Zhang et al., 2005), where ω was fixed and ≤1 (no positive selection). To detect differences (or divergence) in selective constraints among entire clades that had evolved following duplication events, we compared the site-specific discrete Model 1a, which assumes two classes of sites with different ω ratios, to clade Model C (Bielawski and Yang, 2003), which allows selective pressure at one class of sites (foreground clade) to differ from the rest of the phylogeny. The likelihood ratio test statistic (similar to a chi-square distribution) was used to compare nested likelihood models. These test statistics are calculated as 2 × abs (ln*l*_1_ – ln*l*_0_) (i.e., twice the difference between the ln*l* value under each model), with critical values of 2.71 at 5% and 5.41 at 1%. Pairs of orthologous codon-based CD alignments were obtained using the same procedure described in the section “Phylogenetic analysis.” Ka, Ks, and ω were estimated with default parameters using PAML (Yang, 2007).

### Analysis of Sequence Difference as Evidence for Functional Divergence

Following duplication, the sequence difference between paralogs can suggest functional divergence. To detect such sequence divergence, we used the program DIVERGE v3.0 (Gu et al., 2013) to analyze sequence differences in Brassicaceae-specific duplicates. In this program, two types of sequence differences are defined as evidence for functional divergence: Type-I functional divergence is defined as amino acid patterns that are highly conserved in one duplicate among various species, but highly variable in the other duplicate among the same set of species; Type-II functional divergence describes amino acid patterns that differ between paralogs in terms of biochemical properties, but are highly conserved among the set of species under consideration. These types of sequence divergence between paralogs were tested using ML estimates of θ and standard error (SE). *Z*-values were calculated by dividing θ by the SE, and *P*-values were calculated using a two-tailed Z-score test (normal distribution test). The effective number of sites exhibiting such functional divergence was also counted using DIVERGE (Gu et al., 2013).

### Amino Acid Substitution Rates and Sequence-Based Predictions

Per site amino acid substitution rates were estimated in Rate4Site v3.2 using an empirical Bayesian method (Mayrose et al., 2004). The default JTT amino acids substitution model was used as the model of evolution, with 16 categories of gamma distribution. Structural disorder was predicted using DISOPRED3 (Jones and Cozzetto, 2015). The amino acid substitution rates and confidence scores for each sequence were plotted using the ggplot program in the R package. The borders of the AP2 domain were predicted using the SMART Web server (Schultz et al., 1998).

### Cis-Elements and Expression Pattern Analyses

*Cis*-elements in *A. thaliana AP2/ERF* gene promoters were obtained from AGRIS (Yilmaz et al., 2011) and *A. thaliana AP2/ERF* gene expression data were obtained from different datasets: the At-TAX tilling array dataset (Laubinger et al., 2008), AtGenExpress (Schmid et al., 2005), and Expression Atlas (Petryszak et al., 2016). *AP2/ERF* gene expression data from *Glycine max* and *Oryza sativa* were obtained from Severin et al. (2010) and Davidson et al. (2012), respectively. Tissue specificity was measured using the index, τ (Yanai et al., 2005).

## Results

### Phylogenetic Classification and Expansion of AP2/ERF Genes

**FIGURE 1:**
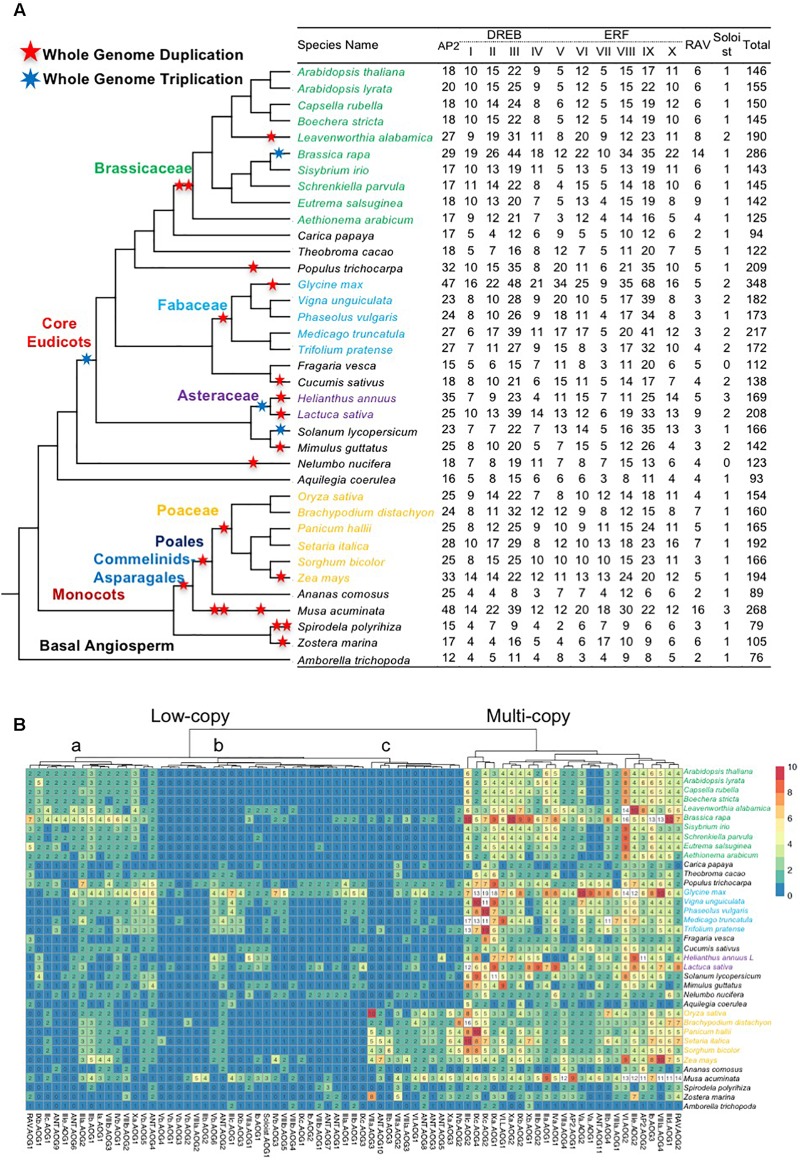
Copy number variation *AP2*/*ERF* genes in each species. **(A)** A species phylogeny and number of *AP2*/*ERF* genes in each subfamily. The species tree in the study was inferred from based on the phylogeny of conserved nuclear genes (Zeng et al., 2014, 2017; Huang et al., 2016a). Genome duplication and triplication events were according to the literature (Jiao et al., 2011, 2012; Vanneste et al., 2014; Huang et al., 2016b; Xiang et al., 2016; Ren et al., 2018), and are depicted by stars. The copy number of each subfamily was indicated. Only sequences that can be aligned and clearly classified are included. **(B)** Copy number variation in each AOGs inferred from gene trees. A heat map showing clustering according to copy number. Rows represent species and columns represent the AOGs.

To systematically analyze genes of the *AP2/ERF* superfamily, we selected 37 angiosperm species (26 eudicots, 10 monocots, 1 basal angiosperm) with genome sequences to represent the major angiosperm lineages. The numbers of *AP2/ERF* genes varied greatly (Figure 1A), from 76 (basal angiosperm *Amborella trichopoda*; *Amborella* hereafter), to 79–268 (monocots), and 93–348 (eudicots), with 348 in *G. max* (Fabaceae, eudicot). Species with large numbers (≥190) of *AP2/ERF* genes were associated with proposed recent WGD events [i.e., *Brassica rapa* (286), *Populus trichocarpa* (209), *G. max* (348), and *Z. mays* (194)], suggesting that WGD events likely increase *AP2/ERF* gene numbers at the family or genus levels. In contrast, aquatic plants, such as seagrass (*Z. marina*) (105) and duckweed (*S. polyrhiza*) (79), have relatively few *AP2/ERF* genes, despite having putative historical WGD events, presumably because they do not need the function of *AP2/ERF* genes for drought response in their aquatic habitats. AP2 protein sequences from 37 angiosperms were classified into 75 AOGs (see the section “Materials and Methods”), each likely derived from a single ancestral angiosperm copy and usually supported by genes from *Amborella*, a sister of other extant angiosperms. The AOGs were classified into subfamilies according to *AP2/ERF* genes from *A. thaliana* or *O. sativa* in each AOG. The Soloist subfamily showed least variation, with one copy in most species and two in some species with recent WGD. Other subfamilies experienced expansion from smaller numbers of ancestral angiosperm genes during angiosperm history (Figure 1A).

Next, we examined differences in duplicate retention among AOGs by constructing a copy number profile matrix (Figure 1B), which provided the number of genes in each AOG for a species. Furthermore, we clustered AOGs based their copy number profiles, using Ward’s hierarchical clustering method, and found that AOGs can be clustered into two groups (Figure 1B): Group 1 contains 21/75 (28%) AOGs covering 87/146 (59.59%) *Arabidopsis AP2/ERF* genes, and 81/159 (50.9%) rice *AP2/ERF* genes; while Group 2 contains 54 AOGs covering 59/146 (40.4%) *Arabidopsis* genes and 78/159 (49.1%) rice *AP2/ERF* genes. AOGs in Group 1 have multiple (generally ≥ 3) copies in most species; however, usually only one in *Amborella*, which lacks evidence of WGD events after divergence from other angiosperms (Amborella Genome Project, 2013). In contrast, AOGs in Group 2 usually have one or a few (generally ≤ 3) genes in a species, and they are even absent in some species. Thus Group 1 is referred to hereafter as the multi-copy group, and Group 2 as the low-copy group. Group 2 can be further divided into three subgroups: Group 2a (generally contains 0–3 genes in the majority of angiosperms), Group 2b (generally contains 0 or 1 copy in the majority of angiosperms, except in some species with recent WGD events), and Group 2c (generally contains 0 or 1 copy in eudicots, but two copies in monocots). Within Group 2, some species show increased copy number, indicating that low-copy AOGs likely experienced lineage-specific duplications, whereas others exhibit a strong tendency to return to single-copy after WGD events. Ten AOGs (four in the ERF V family) in low-copy Group 2b and three in low-copy Group 2c were not recovered from any Brassicaceae genomes we analyzed, supporting their losses in the ancestor of Brassicaceae (Figure 1B). There were also 4 and 14 losses in the MRCA of Fabaceae and Poaceae, respectively.

### Repeated Retention of AP2/ERF Genes in the Multi-copy Group in Multiple Angiosperm Lineages

**FIGURE 2:**
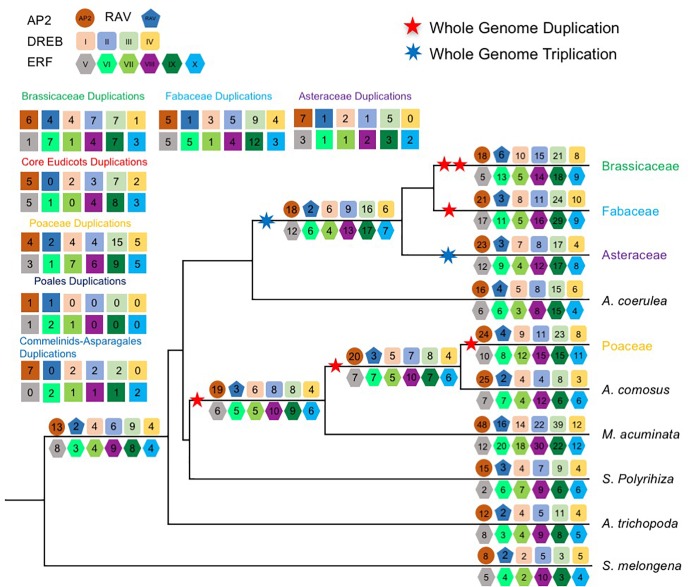
Duplication events inferred from gene trees. Schematic representation of the minimum inferred AP2/ERF protein complement and gene duplication events in the last common ancestor of each major plant group. A numbered geometry represents the copy number in each *AP2*/*ERF* subfamily. The numbers in the top and left geometries represent the minimum inferred gene duplication events in the last common ancestor of each major angiosperm groups. Duplication events were inferred from gene trees and species tree.

**FIGURE 3:**
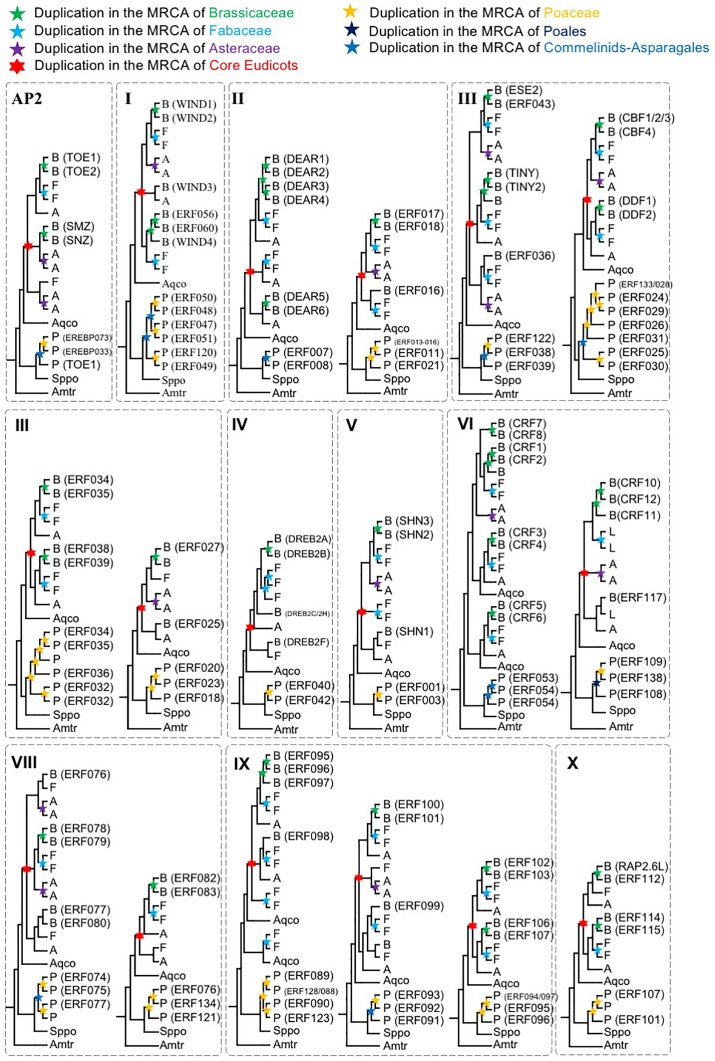
Schematic phylogenetic diagrams of *AP2*/*ERF* genes (simplified phylogenetic trees). The topologies are shown for each AOG generated by combining sequence data and species tree information. See the section “Materials and Methods” and (Supplementary Figures S1–S13) for more complete information on taxon sampling and support values for all nodes. Duplication events of Brassicaceae, Fabaceae, Asteraceae, Core Eudicots, Poaceae, Commelinids, Poales, are indicated by green, light blue, purple, red, orange, blue, and deep blue stars, respectively. Genes from *Aquilegia coerulea*, *Spirodela polyrhiza*, and *Amborella trichopoda* were shown as Aqco, Sppo, and Amtr, respectively, and are not necessarily single copy. The abbreviations used are as follows: B, Brassicaceae; F, Fabaceae; A, Asteraceae.

To further understand the duplication histories of the different AOGs, we investigated the duplication patterns of *AP2/ERF* genes in each AOG (Figure 2) for lineages with known WGD events using gene tree reconciliation (see the section “Materials and Methods”). The results (Figure 3 and Supplementary Figures S1–S13) showed that AOGs in the multi-copy group have retained duplicates corresponding to both WGD events shared by multiple families [γ shared by core eudicots, the Commelinids-specific WGD (τ), or the Poales-specific WGD (σ)] and family-level WGD events [i.e., Brassicaceae WGD (α/β), Poaceae WGD (ρ), Fabaceae WGD, and Asteraceae WGD] (Figure 3). However, duplicates generated by the core eudicot-specific γ were not equally retained in the three eudicot families, Brassicaceae, Fabaceae, and Asteraceae, following their family-level WGD events. For example, three duplicates were generated by γ in DREBIb-AOG3: *AtWIND1*/*AtWIND2*, *AtWIND3*, and *AtWIND4*/*AtERF056*/*AtERF060* [Figure 3(I) and Supplementary Figure S2D]. Among these, only *AtWIND1*/*AtWIND2* retained duplicates from family-specific WGD in Brassicaceae, Fabaceae, and Asteraceae. *AtWIND3* was not duplicated in Brassicaceae and Asteraceae, and was lost in the Fabaceae. The *AtWIND4*/*AtERF056*/*AtERF060* genes were duplicated in the Brassicaceae, corresponding to two duplicates in the Fabaceae, but without a counterpart in the Asteraceae. In another example, two duplicates were generated by the core eudicot-specific γ WGD in DREBIIIc-AOG2: *AtCBFs* and *AtDDFs*. These were followed by additional duplicates from family-specific WGD events in the Brassicaceae and Poaceae; however, only *AtCBFs*, and not *AtDDFs*, were subject to additional duplication during family-specific WGD within the Asteraceae [Figure 3(III) and Supplementary Figure S4F]. In addition, almost all species had several copies of *AtCBF* homologs, generated by species or lineage-specific duplications, whereas this was not the case for *AtDDFs* genes. For example, in *M. truncatula* there are 13 members in the *AtCBF* clade, while there are only 4 in the *AtDDF* clade (Supplementary Figure S4F). In contrast, AOGs in Group 2 tend to have retained a few duplicates from WGD events, either shared by multiple families or specific to a family, thus retaining their low-copy status, even after lineage-specific losses (Supplementary Figures S1–S13). For example, no duplicates from γ were retained and there was Commelinids–Asparagales-specific WGD (τ) in AP2-AOG1 (Supplementary Figure S1A). Nevertheless, some duplicates were generated from family-specific WGD in the Brassicaceae (*AtAP2*/*AtTOE3*), Fabaceae, and Asteraceae, and order-specific WGD in Poales. Two duplicates were generated by γ in VIIIb-AOG3: the *AtLEP* and *AtERF087* lineages (Supplementary Figure S9G); however, no duplicates were retained from subsequent family-specific WGD events. In IIIc-AOG1, genes were lost in the Brassicaceae, whereas three duplicates were retained in the Fabaceae (Supplementary Figure S4E). In IVb-AOG2, three duplicates related to *AtABI4* were found in the Poaceae; however, the copy number was unchanged in the Brassicaceae, Fabaceae, and Asteraceae (Supplementary Figure S5C). The VI-AOG1 gene was lost in the Brassicaceae, whereas two duplicates were retained in the Fabaceae and Asteraceae (Supplementary Figure S7A). In Xa-AOG3, two duplicates were retained from the τ WGD, and duplicates were again retained from the Poaceae-specific WGD (ρ) (Supplementary Figure S11C), whereas no duplicates were generated by WGD events in eudicots. These and other results indicate that low-copy AOGs experienced limited and differential expansion in different angiosperm lineages.

### Additional Gene Clade-Specific Expansions of AP2/ERF Genes

In addition to the duplications described above, we observed that *AP2/ERF* genes exhibit a high incidence of gene clade-specific expansions in different angiosperms. For example, in the clades IIIe-AOG2 (Supplementary Figure S4I) and VIIa-AOG3 (Supplementary Figure S8C), seagrass genes have undergone expansion to a degree not observed in the freshwater duckweed (*S. polyrhiza*), suggesting that the increased gene copies may be important for adaptation to the marine environment. In the sacred lotus, several specific duplications occurred in clades IIId-AOG1 (Supplementary Figure S4G), IVa-AOG1 (Supplementary Figure S5A), and IXc-AOG2 (Supplementary Figure S10F). IIId-AOG1 also contained three lineage-specific duplicates in seagrass and two specific duplicates in duckweed (Supplementary Figure S4G). In IVa-AOG1, nine lineage-specific duplicates were found in lettuce (*Lactuca sativa*) (Supplementary Figure S5A), while in VIIIa-AOG1, one gene clade generated by the γ WGD has undergone specific expansions in *M. truncatula* (six genes); however, their close homologs have been lost in the Brassicaceae (Supplementary Figure S9A). These gains may have contributed to the adaption of corresponding angiosperm lineages to their habitats.

### Duplicates Evolved Under Purifying Selection No Less Than Single Copy Genes

**FIGURE 4:**
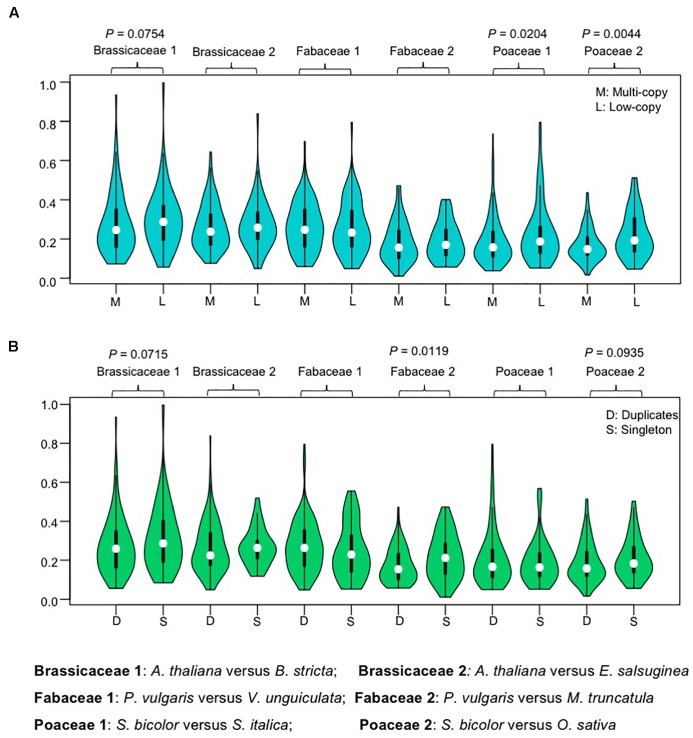
Violin plots showing ω (Ka/Ks) ratio for one-to-one orthologous pairs. **(A)** Violin plots showing ω (Ka/Ks) values for multi-copy group and low copy group gene sets. The data are separated into multi-copy group and low copy group according to the copy number profile matrix. **(B)** Violin plots showing ω (Ka/Ks) values for recent duplicates and singleton sets. The data are separated into recent duplicates (family-level WGDs) and singletons according to previous described gene trees. All evolutionary rates were calculated by comparison of orthologous pairs from selected species. Values were compared using the Wilcoxon rank sum test and *P*-values less than 0.1 are shown above each pair of Violin plots.

Previous genome-wide studies found that the retention of genes is skewed toward duplicates that have minimal phenotypic impact (O’Toole et al., 2017), and some analyses also indicated that conserved proteins tend to exhibit higher gene duplicability (Davis and Petrov, 2004; Liang and Li, 2007). To test these two possibilities for *AP2/ERF* genes, we measured the ratio of non-synonymous (Ka) to synonymous (Ks) substitutions rates (ω = Ka/Ks), as a proxy for the evolutionary rate, using codon-based models in PAML (Yang, 2007). We measured ω values between one-to-one orthologous pairs from two representative species in each of the three angiosperm families. We found that the ω-values had broad distributions for genes in both the multi-copy and low-copy groups, with median ω-values slightly lower in the multi-copy, compared with the low-copy, groups, except for one Fabaceae orthologous pair (*Phaseolus vulgaris* versus *Vigna unguiculata*), perhaps because the phylogenetic relationship of these legume species is too close (Figure 4). The differences in ω-values between multi-copy and low-copy group genes were significant in Poaceae (between *S. bicolor* and *S. italic*; and between *S. bicolor* and *O. sativa*, *P* = 0.020 and *P* = 0.004, respectively; Wilcoxon rank-sum test) (Figure 4). The low ω-values detected suggest both groups have experienced purifying selection during the history of these families, and further imply that genes in the multi-copy group have generally not been under relaxed selection during the evolutionary histories of these families, but may even be under higher evolutionary constraint.

The effect of recent gene duplication on evolutionary constraint was also assessed by sequence comparisons between orthologs in groups that retained duplicates from recent WGD (duplicates) and those that did not (singletons). Duplicates of Brassicaceae, Fabaceae, and Poaceae *AP2/ERF* genes were identified from gene trees. The median -values of recent duplicates were slightly lower than those for singletons, except in one Fabaceae pair 1, where there was a statistically significant difference in the -values of orthologous pairs between the groups with duplicates or singletons in the Fabaceae 2 (*P. vulgaris* versus *M. truncatula*) (Figure 4B). These results further support the idea that *AP2/ERF* duplicates did not experience relaxed selection relative to singletons after the speciation event, and may have experienced higher evolutionary constraints.

To test whether the observed higher evolutionary constraints in duplicated genes reflect post-duplicate neofunctionalization or persistence of an ancestral state, we sought to estimate the evolutionary rates of genes prior to, and independent of, duplication events. We chose to examine two species, *L. alabamica* (Haudry et al., 2013) and *B. rapa* (Wang et al., 2011), both Brassicaceae, which have undergone recent lineage-specific WGD events. In contrast, *A. thaliana*, *Boechera stricta*, and *Eutrema salsuginea* exhibited no evidence of WGD after divergence from other species in the same family. As evolutionary rates for a particular gene are highly correlated within the same family, we reasoned that the evolutionary rates of genes in these outgroups can serve as proxies for the rate of the ancestral gene in the common ancestor of *Arabidopsis* and *B. rapa* in the Brassicaceae. We then partitioned every pair of family-specific duplicates (corresponding to Brassicaceae WGD events) into two categories with greater and fewer gene copies relative to one another. We found that gene clades from the outgroup contained more retained duplicates, and had evolved significantly more slowly, than the sister clade, supporting the model of preferential retention of slower evolving *AP2/ERF* genes (Supplementary Figure S14). As *AP2/ERF* gene copy numbers in *G. max* and *Z. mays* were the same in most family-specific (Fabaceae or Poaceae) duplicate pairs, we could not conduct this analysis for these two families.

### AP2/ERF Clades With Duplicates From WGD Events Are Enriched for Broad Gene Expression Patterns

**FIGURE 5:**
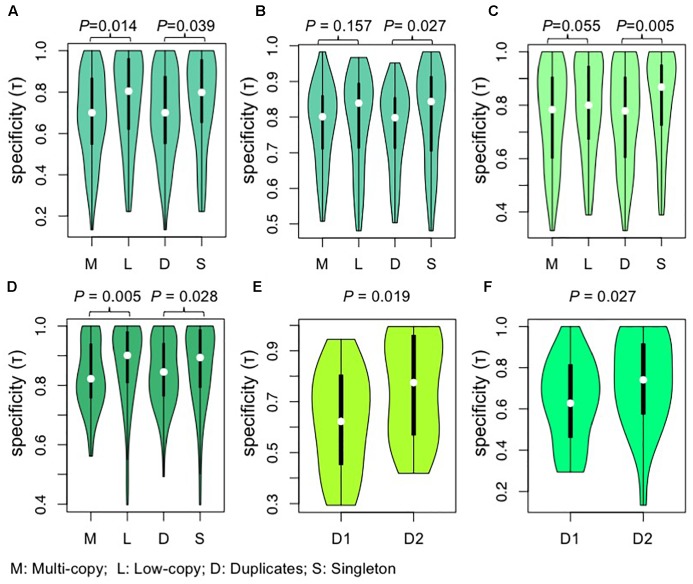
Violin plots showing the comparisons of tissue specificity (τ) among different groups of AP2/ERF genes. **(A,B)** The comparisons of tissue specificity (τ) among different groups of genes from *A. thaliana*. **(A)** Gene expression data of roots, leaves, flowers, and siliques were obtained by high-throughput sequencing of *A. thaliana* from expression atlas (Petryszak et al., 2016). **(B)** Gene expression data of 79 samples were obtained from AtGenExpress expression atlas (Schmid et al., 2005). **(C)** The comparisons of tissue specificity (τ) among different groups of genes from *G. max*. Gene expression data of young leaf, flower, pods, seeds, roots, and nodules were obtained by high-throughput sequencing of *G. max*. Gene expression data were download from RNA-Seq atlas of *G. max* (Severin et al., 2010). **(D)** The comparisons of tissue specificity (τ) among different groups of genes from *O. Sativa*. Gene expression data of 10 tissues (seedling four-leaf stage, leaves-20 days, shoots, pre-emergence inflorescence, emerging inflorescence, anther, pistil, seed-5 DAP, seed-10 DAP, endosperm-25 DAP) were download from Rice Genome Annotation Project (Davidson et al., 2012). Values were compared using the Wilcoxon rank-sum test and *P*-values are shown above each pair of Violin plots. **(E, F)** Tissue specificity (τ) differences between two paralogous clades in Brassicaceae. D that each duplicate is responsive to. The duplicate pairs generated near the origin of Brassicaceae are separated into D1 and D2 according to their copy number of *AP2*/*REF* genes from *Leavenworthia alabamica* **(E)** or *B. rapa* **(F)**, with the D1 having more gene copy in *L. alabamica* **(E)** or *B. rapa* **(F)**. Values were compared using the Wilcoxon rank-signed tests and *P*-values are shown above each pair of Violin plots. M, multi-copy genes; L, low-copy genes; D, duplicates; S, singleton; DAP, days after pollination.

Highly and broadly expressed genes tend to evolve slowly (Liao et al., 2007). To test whether these trends held true for different clades within the *AP2/ERF* superfamily, and to investigate a possible relationship between duplicate retention and expression pattern, we compared gene expression profiles among *AP2/ERF* gene duplication categories for species with expression data. The results showed that multi-copy genes were expressed more broadly (higher expression breadth) than low-copy genes in *A. thaliana*, *G. max*, and *O. sativa* (Figure 5A–D). Further, we compared the expression patterns of relatively recent duplicates from family-specific WGD events with those of single-copy genes, with similar results (Figure 5A–D). We also tested whether the observed high expression breadth in duplicated genes reflected post-duplicate neofunctionalization or persistence of an ancestral state, based on the suggestion that the expression of orthologous genes is generally conserved within the same family. We found that gene clades containing more retained duplicates had lower tissue specificity than sister clades in the outgroup (Figure 5E,F), supporting the model of preferential retention of genes with lower tissue specificity.

### Divergence in *Cis*-Elements, Partitioning, and Expression Patterns Between Paralogs in Brassicaceae

Generally, duplicated genes are retained after functional differentiation (Zou et al., 2009), and some analyses indicate that dosage balance sensitivity is a general characteristic of repeatedly retained genes (Gout and Lynch, 2015; Xie et al., 2016; Tasdighian et al., 2017). To test these possibilities, we analyzed the divergence of 96 duplicates (44 paralogous pairs/trios) generated by the WGD that occurred near the origin of the Brassicaceae. Mutations in *cis*-regulatory regions have been hypothesized to serve as the mechanistic basis for functional differentiation (Zou et al., 2009); therefore, we investigated whether there were changes in the *cis*-element content in the promoter regions of duplicate *AP2/ERF* genes in *A. thaliana*. Specifically, we asked whether there were duplicate pairs with asymmetric partitioning of *cis*-elements. First, we examined the pattern of partitioning of *cis*-elements among duplicates and found that it tended to be asymmetric (Supplementary Figure S15). The number of intersections between *cis*-elements of duplicates was small, suggesting that the *cis*-elements are retained in both duplicates, possibly causing the duplicates to function in ways complementary to one another. To further test for divergence in expression, we examined expression datasets (Laubinger et al., 2008) and found that paralogs mainly exhibited three differential gene expression patterns (Supplementary Figure S16): (1) one copy predominates in all tissues (*TOE1* versus *TOE2*, *ERF017* versus *ERF018*, *DEAR1* versus *DEAR2*, *ERF042* versus *ERF043*, and *ERF114* versus *ERF115*); (2) one copy had more expression in some tissues (*ERF034* versus *ERF035*, *TINY* versus *TINY2*, and *TEM1* versus *TEM2*); and different paralogs had high expression levels in different tissues (*AIL6* versus *AIL7*, *ERF110* versus *ERF108*, and *ERF112* versus *ERF113*).

### Sequence Divergence of *AP2/ERF* Duplicates and Evidence of Positive Selection

**FIGURE 6:**
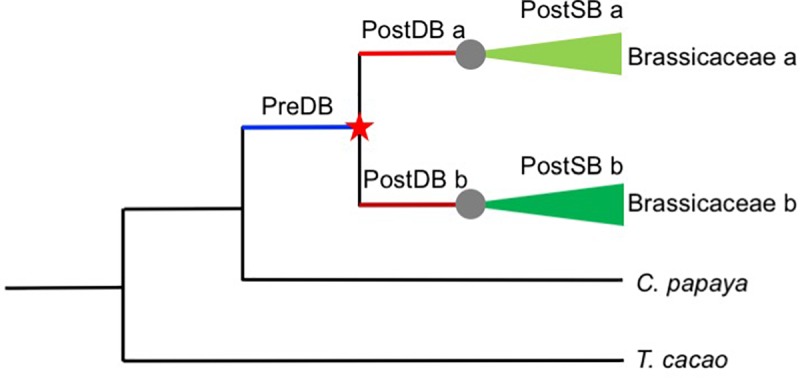
Schematic representation of the *AP2*/*ERF* gene duplication data sets employed in this study. Red star indicates a duplication event and gray circles speciation event. Pre-duplication branch (PreDB), Post-duplication branch (PostDB), and Post-speciation (PostSB).

Duplicated genes often evolve more rapidly than the ancestral gene before duplication, in a time-dependent process (Pegueroles et al., 2013), presumably due to relaxed selection for the duplicates, compared with the single-copy ancestor. To tested whether post-duplication *AP2/ERF* genes experienced relaxation of selective constraints relative to their pre-duplication ancestral genes, we used the RELAX program to analyze codon-based alignments of Brassicaceae-specific duplicates and their orthologs in *T. cacao* and *C. papaya* and the corresponding phylogenetic tree. Given two categories of branches within a phylogeny, a set of test branches and a set of “reference” branches, RELAX tests whether the strength of selection has been relaxed or intensified in test branches compared with “reference” branches (Wertheim et al., 2015). To compare the strength of selection of different branches before and after duplication, we focused on genes duplicated before the divergence of the extant Brassicaceae species included in this study. We divided the branches in gene trees into three types (Figure 6): Pre-Duplication Branches (PreDB, before the α/β WGD events shared by Brassicaceae taxa), Post-Duplication Branches (PostDB, after the α/β WGD events, but before the divergence of extant Brassicaceae taxa), and Post-Speciation Branches (PostSB, since the first divergence of Brassicaceae lineages). In the *AP2*/*ERF* trees, there were 44 pairs/trios (PreDBs) in the Brassicaceae with 96 PostDBs. Compared with the PreDBs, only four PostDBs underwent relaxed selection (Supplementary Tables S4, S5), suggesting that most duplicates have been under selective constraint to a similar extent as their ancestors.

To further examine sequence divergence as evidence for positive selection and functional divergence of duplicates from the α/β WGD events in Brassicaceae, we determined the ω ratio [Ka/Ks, using codon-based models in PAML (Yang, 2007) for 96 PostDBs, as an indication of selective pressure. The mean ω, estimated using the one-ratio model (Model 0), ranged from 0.056 to 0.219 for these duplicates, suggesting that they were under purifying selection during the history of the Brassicales. In addition, analyses using the two-ratio branch model showed that only 17/96 (17.71%) PostDBs differed significantly from the background ω value (Supplementary Table S6, yellow background; *P* < 0.05), indicating that the majority of PostDBs did not experience a shift in selective pressure, was consistent with the results of analyses using RELAX (see above). Among these 17 branches, 7 were in the *AP2* subfamily (12 PostDBs in total), 9 genes were in the *DREB*/*ERF* subfamilies (78 PostDBs in total), and 1 gene was in the *RAV* subfamily (6 PostDBs in total). To examine whether PostDBs were under positive selection before the radiation of the Brassicaceae taxa, we used branch-site models (Yang et al., 2005; Zhang et al., 2005). Of the 96 PostDBs we tested, signals of positive selection were detected in 20 (20.83%) branches from 14 (31.82%) pairs/trios (Table 1 and Supplementary Figure S17, green background; *P* < 0.05), indicating that functional divergence of PostDBs was, at least in part, related to positive selection and the potential for adaptive evolution in these genes along the Brassicaceae. Among the 20 branches, 4 branches were in the *AP2* subfamily, 15 in the *DREB/ERF* subfamilies, and 1 in the *RAV* subfamily.

**Table 1:** List of detected positive selection on the post-duplication branches of *AP2*/*ERF* family in Brassicaceae using branch-site model.

Foreground branch	Model	In *L*	2Δ*l*	Site class	Positive selection
PLT2	Model A null	9245.76	14.59^∗∗^	ω0 = 0.041 ω1 = 1.000 ω2 = 998.999	412S^∗∗^ 498D^∗∗^
	Model A	9238.47			
PLT1	Model A null	9245.06	9.37^∗∗^	ω0 = 0.041 ω1 = 1.000 ω2 = 998.999	539T^∗∗^
	Model A	9240.38			
AIL6	Model A null	7797.22	2.89^∗^	ω0 = 0.062 ω1 = 1.000 ω2 = 9.034	346N#^∗^
	Model A	7795.78			
TOE1	Model A null	4054.23	3.59^∗^	ω0 = 0.099 ω1 = 1.000 ω2 = 9.123	106S^∗^
	Model A	4052.44			
WIND1	Model A null	3882.48	11.29^∗∗^	ω0 = 0.055 ω1 = 1.000 ω2 = 999.000	294A^∗∗^
	Model A	3876.83			
ERF053	Model A null	3883.26	6.07^∗∗^	ω0 = 0.099 ω1 = 1.000 ω2 = 12.640	270S^∗^ 282A^∗^
	Model A	3880.22			
ERF017	Model A null	3064.50	10.10^∗∗^	ω0 = 0.057 ω1 = 1.000 ω2 = 999.000	79N^∗^
	Model A	3059.45			
ERF014	Model A null	3526.90	36.35^∗∗^	ω0 = 0.056 ω1 = 1.000 ω2 = 1.284	31R 32M^∗^ 109N^∗∗^ 115S^∗^ 125A^∗∗^
	Model A	3508.72			
ERF043	Model A null	3955.99	8.69^∗∗^	ω0 = 0.046 ω1 = 1.000 ω2 = 999.000	55M#^∗∗^ 93R^∗∗^
	Model A	3951.65			
CBF2	Model A null	4800.62	3.83^∗^	ω0 = 0.032 ω1 = 1.000 ω2 = 999.000	37P
	Model A	4798.71			
RAP2.2	Model A null	4219.29	7.69^∗∗^	ω0 = 0.078 ω1 = 1.000 ω2 = 999.000	152E#^∗^
	Model A	4215.45			
RAP2.10	Model A null	2956.35	6.48^∗∗^	ω0 = 0.112 ω1 = 1.000 ω2 = 998.998	180K^∗^ 181V^∗^
	Model A	2953.11			
ESR1	Model A null	4953.85	3.01^∗^	ω0 = 0.035 ω1 = 1.000 ω2 = 998.993	103F 232K^∗^
	Model A	4952.34			
ESR2	Model A null	4955.52	2.82^∗^	ω0 = 0.039 ω1 = 1.000 ω2 = 998.932	6M 197I
	Model A	4954.11			
ERF015	Model A null	5763.05	2.78^∗^	ω0 = 0.065 ω1 = 1.000 ω2 = 9.358	25N 133T#
	Model A	5761.66			
ORA59	Model A null	5762.71	3.36^∗^	ω0 = 0.066 ω1 = 1.000 ω2 = 999.000	129L#
	Model A	5761.03			
ERF100	Model A null	3890.53	7.91^∗∗^	ω0 = 0.051 ω1 = 1.000 ω2 = 999.000	104E^∗∗^
	Model A	3886.57			
ERF101	Model A null	3892.97	16.45^∗∗^	ω0 = 0.057 ω1 = 1.000 ω2 = 999.000	82D
	Model A	3884.75			
RAP2.6L	Model A null	3654.68	2.91^∗^	ω0 = 0.051 ω1 = 1.000 ω2 = 999.000	192Q
	Model A	3653.22			
RAV1	Model A null	10,643.41	3.55^∗∗^	ω0 = 0.059 ω1 = 1.000 ω2 = 34.664	275S^∗∗^
	Model A	10,641.65			


*Model A null (ω2 is fixed as 1) vs. Model A; degree of freedom (df) = 1 for all the comparisons; In *L*: the log-likelihood difference between the two models; 2Δ*l*: twice the log-likelihood difference between the two models. The amino acid sequence of *A. thaliana* was used as the reference sequence, and positive selected sites were identified with posterior probability. For positive selected sites, posterior probabilities (PP) > 90% (>95% indicated with asterisks, >99% indicated with double asterisks) are shown. The arrangement of positive selected sites in *A. thaliana* AP2 domains are indicated with pound sign.*

In addition, paralogs might experience different selective pressures, allowing differential sequence changes, and potentially resulting in functional divergence. To detect such differences between paralogous clades, as evidence for differential functional constraints and selective pressure, we examined paralogous clades for differences in sequence divergence using Clade Model C (Bielawski and Yang, 2003) implemented in PAML (Yang, 2007). This model allows the selective constraint on a proportion of sites to vary among three partitions (0 < ω_0_ < 1,ω_1_ = 1,ω_2_ and _3_) for different clades of the phylogeny. Through comparison to a null model that allows two partitions (0 < ω_0_ < 1,ω_1_ = 1), Clade Model C tests for a long-term shift in the intensity of selection (i.e., divergent selective pressures). Our results showed that the functional constraints (as evidence for shift in selective pressure) were not similar between paralogous clades in 43 of 44 pairs/trios (97.73%; *P* < 0.05) (Supplementary Table S6), suggesting that natural selection, likely mediated via ecological niches, has had a substantial influence on *AP2*/*ERF* duplicate evolution in the Brassicaceae. To identify amino acid sites likely responsible for functional changes between two paralogs, we used the DIVERGE program (Gu et al., 2013), which is based on functional constraint patterns in MSAs and corresponding gene trees. Significant support for Type-I functional divergence (see the section “Materials and Methods”) was identified between paralog clades in 44/58 pairs (75.86%) (Supplementary Tables S7, S8). Hence, sequence divergence results from both Clade Model C and DIVERGE analyses strongly support that a majority of *AP2*/*ERF* paralogous pairs generated in the early Brassicaceae have subsequently experienced differential selective pressures and undergone functional divergence. This is likely due to the long-term selection pressures acting on Brassicaceae species.

### Evolutionary Rates and Structural Disorder Across Brassicaceae Paralogs

To examine whether different regions/sites of AP2 proteins experienced more rapid or slower transition than other regions/sites, evolutionary rates for amino acid sequence substitutions were calculated based on a MSA of Brassicaceae-specific paralogous pairs and their orthologs in *T. cacao* and *C. papaya*, with the corresponding gene trees, using Rate4Site v3.2 (Mayrose et al., 2004). The results showed that amino acids located in the AP2 domain substituted more slowly than those in regions flanking this domain (Supplementary Figure S17). In addition, there were also some other slowly transitioning sites, such as those C-terminal of the AP2 domain in subfamilies II and III, suggesting that they are functionally important and experienced purifying selection. Rapidly transitioning sites were usually only conserved on the level of pairs of paralogs, rather than the subfamily level, suggesting different functional constraints in different paralogs.

In addition, we investigated the propensity for structural disorder and potential protein binding sites in the amino acid sequences of *AP2*/*ERF* duplicates retained in *Arabidopsis* using DISOPRED3 (Jones and Cozzetto, 2015). Disordered regions were predicted in each AP2/ERF protein and their locations in pairs of paralogs were generally conserved. The presence of disordered regions in each AP2/ERF protein suggests the importance of conformational flexibility for their functions (Supplementary Figure S18). The regions C-terminal of the AP2 domains in members of the DREB II and III subfamilies are predicted to be ordered. In addition, members of the DREB III subfamily also possess ordered regions near the C terminus (Supplementary Figure S18). In the ERF VI subfamily, the N-terminal region flanking the AP2 domain is ordered, with an adjacent disordered region. Also, the linker regions between two AP2 domains are generally ordered in members of the AP2 subfamily. These profiles indicate that the positions of disordered regions are similar among different subfamilies, suggesting that these regions are prone to be disordered, or that these disordered regions are functionally important. We then compared the evolutionary rates of the predicted disordered and ordered regions, and found that disordered regions have higher evolutionary rates than those of ordered regions (*P* < 2.2*e*-16, Wilcoxon Rank-Sum test) (Supplementary Figure S19).

### Inferred Ancestral Expression Supports Subfunctionalization of Widely Expressed AP2/ERF Genes With Repeated Retention Patterns

**FIGURE 7:**
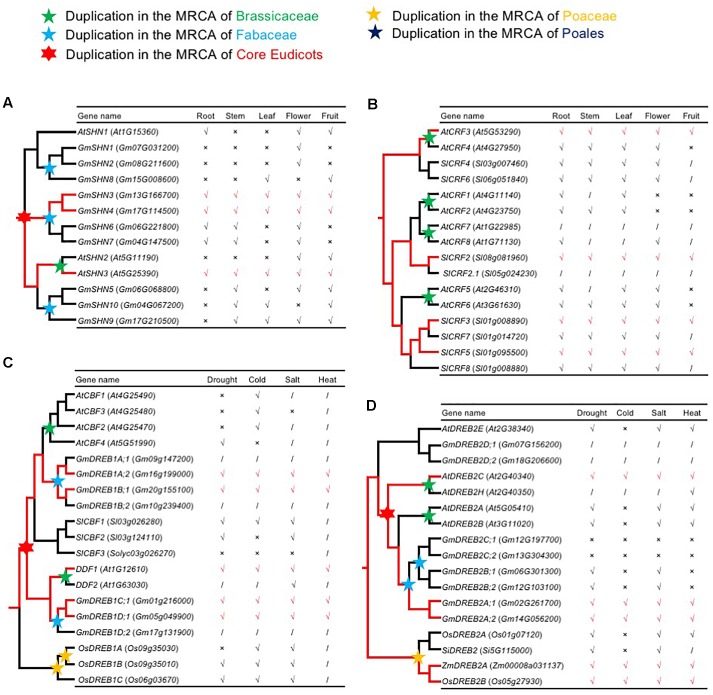
The reconstruction of ancestral expression state. **(A,B)** The ancestral expression profiles of *SHNs* and *CRFs* in roots, stems, leafs, flowers, and fruits were inferred. Tick, Cross, and Slash indicate that gene expression is detected expression, not detected expression, and none data, respectively. Putative ancestral expression state is highlighted in red. **(A)** The reconstruction of ancestral expression of *SHNs*. Expression data of *A. thaliana SHNs* were obtained from previous studies (Aharoni, 2004). Expression data of *G. max SHNs* were obtained from previous studies (Xu et al., 2016). **(B)** The reconstruction of ancestral expression of *CRFs*. Expression data of *CRFs* from *A. thaliana* (Zwack et al., 2012; Jeon et al., 2016) and *S. lycopersicum* (Shi et al., 2012; Gupta and Rashotte, 2014; Shi et al., 2014) were obtained from previous studies. **(C,D)** The ancestral expression profiles of *DREB1s* and *DREB2s* in response to cold, drought, salt, and heat stress were inferred. Tick, Cross, and Slash indicate that gene expression is up-regulated and not changed and none data, respectively. Putative ancestral stress responses are highlighted in red. **(C)** The reconstruction of ancestral stress responses of *DREB1s*. Expression data of *DREB1s* from *A. thaliana* (Medina et al., 1999; Kang et al., 2011) and *G. max* (Kidokoro et al., 2015) were obtained from previous studies. **(D)** The reconstruction of ancestral stress responses of *DREB2s*. Expression data of *DREB2s* from *A. thaliana* (Krishnaswamy et al., 2011), *S. italica* (Lata et al., 2011), *O. sativa* (Matsukura et al., 2010), and *Z. mays* (Qin et al., 2007) were obtained from previous studies.

Several duplicate *AP2*/*ERF* genes show differential expression patterns, providing an opportunity to test the idea that widely expressed *AP2*/*ERF* genes with strong, repeated retention patterns experience subfunctionalization following duplication. In one case, three *Arabidopsis AP2*/*ERF* genes in the same clade differed in spatial and temporal expression patterns, with one encoding SHINE1/WAX INDUCER1 (SHN1/WIN1), a regulator of cuticular wax synthesis and flower development (Aharoni, 2004). Reconstruction of the ancestral expression states allows inference of gain and loss of expression among gene duplicates (Zou et al., 2009). Therefore, we inferred the expression patterns of the ancestral SHINE clade genes in root, stem, leaf, flower, and fruit, as described below. In the SHINE clade, Va-AOG2, three copies were generated by the core eudicot-specific WGD (γ): the *AtSHN1* clade, the *AtSHN2*/*AtSHN3* clade, and another clade lacking genes in *A. thaliana* (Figure 7A and Supplementary Figure S6B). In the *AtSHN1* clade, *AtSHN1* expression was detected in flower, fruit, and root tissues, but not in stem and leaf (Aharoni, 2004); *GmSHN1* and *GmSHN2* expression were detected only in flower, and *GmSHN8* expression was detected in leaf and fruit (Xu et al., 2016). In the *AtSHN2*/*AtSHN3* clade, *AtSHN2* expression was detected in flower and fruit; *AtSHN3* was most broadly expressed, with transcription in all plant organs (Aharoni, 2004); *GmSHN9* was expressed in adult stem and leaf; *GmSHN5* in adult stem, flower, and fruit; and *GmSHN10* in adult stem, leaf, and fruit (Xu et al., 2016). In the clade without *A. thaliana* genes, *GmSHN3* and *GmSHN4* were detected in all plant organs, with *GmSHN6* and *GmSHN7* in root, stem, and flower (Xu et al., 2016). *AtSHN3* and *GmSHN3*/*GmSHN4* were detected in all plant organs; however, they are in different clades. According to Occam’s Razor and the rule, “degenerative mutations are much more frequent than beneficial mutations” (Force et al., 1999), we speculate that the single ancestral gene in the MRCA of extant angiosperms of *SHINEs* should be expressed in all plant organs (Figure 7A). Expression of some genes in the SHINE clade was lost or significantly reduced in some tissues. We speculate that the ancestral gene of the *AtSHN2/AtSHN3* clade was expressed in all plant organs and that the ancestral gene of the *AtSHN1* clade was expressed in root, leaf, flower, and fruit. The tissue specificity of the ancestral gene of the *AtSHN2*/*AtSHN3* clade was lower than that of the ancestral gene of the *AtSHN1* clade. We observed more retention after WGD events in the *AtSHN2*/*AtSHN3* clade than the *AtSHN1* clade in different plant groups, confirming the model, “preferential retention of *AP2*/*ERF* genes with wide expression.”

Cytokinin response factor genes (*CRFs*) encode TFs belonging to a small family within the ERF VI subfamily [Figure 3(VI) and Supplementary Figure S7B]. Recent studies have revealed the biological functions of some *Arabidopsis* CRFs, providing insight into the role of these plant TFs in integrating environmental and hormonal signals for plant adaptation. There are three clades in ERF VI-AOG2, each of which had at least one gene with expression detected in all organs (*AtCRF3*, *SlCRF2*, *SlCRF3*, and *SlCRF5*) (Shi et al., 2012; Zwack et al., 2012; Gupta and Rashotte, 2014; Shi et al., 2014; Jeon et al., 2016) (Figure 7B), indicating that their single ancestral gene, in the MRCA of extant angiosperms, was also expressed in all plant organs, and that other genes originated from this single ancestral gene may have lost, or significantly reduced, their expression in some tissues.

Some *AP2*/*ERF* genes are responsive to various environmental stressors, while others are only responsive to a few environmental stressors. To test the hypothesis that some *AP2/ERF* genes lost responsiveness, or became less responsive to stressors, we inferred the stress response patterns of ancestral *AP2*/*ERF* genes. *CBF*/*DREB1* is reported to be associated with regulation of gene expression under stress conditions. *DDF1* and *DDF2* are induced by salt and confer tolerance to salt stress when ectopically expressed (Magome et al., 2004). These two clades were generated by core eudicot-specific WGD (γ) in DREB IIIc-AOG2 [Figure 3(III) and Supplementary Figure S4F], and each clade has genes responsive to drought, salt, cold, and heat (*GmDREB1A;2* and *GmDREB1B;1* in the CBF clade; *AtDDF1*, *GmDREB1C;1*, and *GmDREB1D;1* in the DDF clade) (Medina et al., 1999; Magome et al., 2004; Kang et al., 2011; Kidokoro et al., 2015). These results indicate that the single ancestral gene of IIIc-AOG2 was likely drought, salt, cold, and heat responsive, and that some genes have retained more ancestral stress responses, while others have lost, or significantly reduced, these responses (Figure 7C). *AtCBF1-3* genes are induced under cold stress, while *AtCBF4* gene expression is up-regulated by drought stress, but not by low temperature. Overexpression of *AtCBF4* in transgenic *Arabidopsis* plants results in activation of downstream genes involved in cold acclimation and drought adaptation. Consequently, the transgenic plants are more tolerant to freezing and drought stress (Novillo et al., 2004). Given the physiological similarity between freezing and drought stress, and the sequence and structural similarity of the AtCBF/DREB1 and AtCBF4 proteins, we propose that a plant responsive to cold and drought evolved with a common CBF-like TF, first through gene duplication and then via the evolution of promoter and coding sequences. Some *DREB2* genes in DREB IVa-AOG1 [Figure 3(IV) and Supplementary Figure S4F] are also responsive to drought, salt, cold, and heat (Qin et al., 2007; Matsukura et al., 2010; Krishnaswamy et al., 2011; Lata et al., 2011), which also indicates that the single ancestral gene of DREB IVa-AOG1 was broadly responsive to stress (Figure 7D), further implying that broadly responsive *AP2*/*ERF* genes are more likely to be retained after WGD events.

## Discussion

### Highly Similar Patterns of AP2/ERF Duplicate Retention Across all Angiosperms

Gene duplication is a major source of evolutionary novelty and plays a key role in speciation (Ren et al., 2018). Here, we found that the MRCA of extant angiosperms possessed at least 75 *AP2*/*ERF* genes, indicating numerous duplication events and substantial preexisting functional diversity before angiosperm radiation. Further analyses of the duplicate retention patterns of *AP2*/*ERF* genes in 37 angiosperm genomes indicated an important contribution of WGD to the increase in *AP2*/*ERF* numbers. Although most duplicates originating from WGD were subsequently lost (Li et al., 2016; Ren et al., 2018), a substantial fraction of *AP2*/*ERF* duplicates was retained after WGD events of different ages in the MRCA of core eudicots, Brassicaceae, Fabaceae, Asteraceae, Commelinids, Poales, and Poaceae. The *AP2*/*ERF* duplicate retention patterns following these WGD events were highly similar across angiosperm genomes, with over 72% (54/75) of AOGs maintaining a low-copy status, whereas a smaller set (28%, 21/75) retained duplicates from multiple lineage-specific WGD events. Relatively few gene families have expanded greatly during evolution (i.e., there is “dominance by a selected few”) (Karev et al., 2002, 2003, 2004; Novozhilov et al., 2006), with the size distribution of gene families in many genomes being similar to a generalized Pareto distribution. Our results, indicating that only 21 (28%) among 75 AOGs have expanded, suggesting that “dominance by a selected few” may also hold true at the level of OrthoGroups within a gene family.

An inspection of the annotated functions of *AP2*/*ERF* genes shows that stress-related *AP2*/*ERF* genes are enriched in the multi-copy group in *Arabidopsis*. Among 87 *Arabidopsis AP2*/*ERF* genes in the multi-copy group, 38 (43.68%) are related to stress responses, including *AtWINDs* (Iwase et al., 2016), *AtDREB2s* (Chen et al., 2012), *AtCBFs* (Kang et al., 2013; Haake et al., 2016), *AtSHN1* (Aharoni, 2004), *AtCRFs* (Jeon et al., 2016; Kim, 2016), *AtDDFs* (Kang et al., 2011), *AtHREs* (Licausi et al., 2010), and *AtRAP2.6L* (Krishnaswamy et al., 2011) (Figure 3 and Supplementary Table S1). Indeed, every annotated AOG in the DREB/ERF family multi-copy group had at least one stress response-related gene. Among 57 *Arabidopsis AP2*/*ERF* genes in the low-copy group, only 12 (20.34%) are related to stress responses, with 18 (30.51%) associated with development, including *WRI1* (fatty acid biosynthesis) (To et al., 2012), *FUF1* (flower senescence) (Chen et al., 2015), *TINY* (shoot development) (Wilson, 1996; Wei et al., 2005), *DRN* and *DRNL* (morphogenesis), *PUCHI* (morphogenesis) (Chandler and Werr, 2017), and genes in the AP2 family (Licausi et al., 2013). Thus, in addition to previous findings of preferential retention of duplicates involved in signaling and transcriptional regulation (Li et al., 2016; Ren et al., 2018), our results suggest further differential retention of duplicates in the same TF family, with preference for OrthoGroups involved in environmental responses, possibly contributing to adaption to highly variable environmental conditions, consistent with morphological and ecological evolutionary findings in angiosperms (Zeng et al., 2014).

### Lineage-Specific Expansions and Their Role in Adaptation to Aquatic Environments

Tolerance to submergence and low oxygen availability (hypoxia) are influenced by different members of subfamily VII in *Arabidopsis* [*AtRAP2.12*; *AtRAP2.2* (Licausi et al., 2011; Paul et al., 2016); *AtRAP2.3* (Paul et al., 2016); *AtHRE1* and *AtHRE2* (Licausi et al., 2010)] and rice [(*OsSUB1s* (Xu et al., 2006); *OsSK1* and *OsSK2* (Hattori et al., 2009)]. The copy number of the VII subfamily was almost unchanged from the MRCA of angiosperms (4) to the MRCA of each major plant group [(Brassicaceae (5), Fabaceae (5), Asteraceae (4), core eudicots (4), Commelinids (5), and Poales (5)], but it was significantly expanded in Seagrasses (17), and slightly expanded in duckweed (7) and sacred lotus (7). Specifically, *AtRAP2.12*, *AtRAP2.2*, and *AtRAP2.3* are important for hypoxia survival (Paul et al., 2016), and their orthologs are also duplicated in seagrasses, duckweed, and sacred lotus (Supplementary Figures S8A,D). There are three *AtRAP2.3* orthologs in sacred lotus. Similarly, rice *SUB1A*, *SUB1B*, *SUB1C* (Xu et al., 2006), *SK1*, and *SK2*, are orthologs of *AtHRE1*, and contribute to adaptation to deep water (Hattori et al., 2009) and improved water use efficiency, as do paralogs in VIIa-AOG3 from Poaceae-specific and rice-specific duplications (Supplementary Figure S8C). The orthologs of *AtHRE1* in seagrasses (eight duplicates) also expanded independently, while those in duckweed and sacred lotus did not (Supplementary Figure S8C), suggesting different pathways during these parallel, but independent, colonization events. *AtHRE2* has five orthologs in seagrass, three in duckweed, and two in sacred lotus (Supplementary Figure S8B). The rice ERF-VII proteins contain an N-terminal sequence, the MC motif, that is important for oxygen-dependent degradation of these proteins, rendering them more stable under hypoxia and causing increased transcriptional activation of genes induced by low-oxygen (Licausi et al., 2011). Six seagrass homologs of *OsSUB1A-1* and one duckweed homolog (*Nenu.14844*) of *AtRAP2.3* lack the MC motif, likely making their stable insensitivity to oxygen constitutively active. In addition, several ERF-VII TFs in seagrass and duckweed have altered amino acids in the MC motif (Supplementary Figure S20). These changes in crucial regulators of low-oxygen responses may have had important functions in adaptation to persistent hypoxic environments.

### Preferential Retention of Widely Expressed AP2/ERF Genes Following Subfunctionalization

**FIGURE 8:**
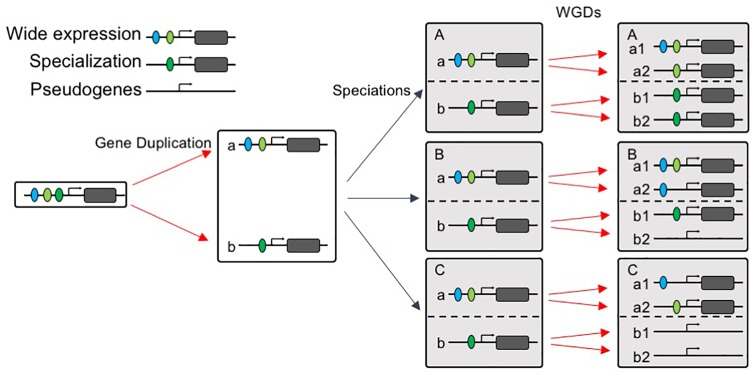
“Wide expression-high duplicability” model. A widely expressed gene were subfunctionalized into gene *a* and gene *b* after duplication, with gene a expressed more widely than gene b. After speciations, species A, B, and C all retained gene *a* and gene *b*, when species A, B, and C experienced WGD, wide expressed gene *a* tends to be retained in all species and one or both copies subfunctionalized, but gene *b* with narrower expression tends to be retained in few species.

Our data reveal that the multi copy *AP2*/*ERF* genes, which exhibit repeated duplicate retention patterns in angiosperms (i.e., the *AP2*/*ERF* genes that show the strongest patterns of preferential retention after WGD are repeatedly retained in the genomes of angiosperms), did not evolve more rapidly, and some even evolved more slowly, on average than low-copy genes. Slower rates of protein evolution correlate with higher levels, and greater breadth, of expression, as well as a larger number of protein interactions (Liao et al., 2007; Yang and Gaut, 2011). Hence, we compared the expression patterns of the two groups of genes and found that, across the angiosperms as a whole, widely expressed *AP2*/*ERF* genes were more likely to be retained than those with narrower expression profiles, consistent with the preferential subfunctionalization of slow-evolving genes (Sémon and Wolfe, 2008). These findings suggest that, consistent with previous findings, the evolutionary trajectory of *AP2*/*ERF* duplicates may possess two stages: post-duplication functional divergence, followed by a generally slow evolutionary rate, owing to the higher level of functional constraints after functional divergence (Davis and Petrov, 2004; Pegueroles et al., 2013). Next, we asked a different type of question: namely, why are some types of gene are more likely to be duplicated than others? Several models of duplicate gene preservation have explained why slowly evolving genes may have an increased likelihood of being preserved. Some models predict that repeatedly retained genes will be retained as duplicates following WGD events, simply because they are beneficial for gene dosage (Gout and Lynch, 2015; Xie et al., 2016; Tasdighian et al., 2017). Other models predict the preferential preservation of genes expressed in numerous tissues (Force et al., 1999), or of genes that encode multidomain proteins (Liang and Li, 2007). As degenerative mutations are much more frequent than beneficial alterations, it follows that subfunctionalization may be a much more common mechanism of duplicate-gene preservation than neofunctionalization (Force et al., 1999); our results, and several examples of *AP2*/*ERF* genes, support the latter model. These observations lead us to propose that widely expressed genes are more easily subfunctionalized, and therefore more easily retained, after WGD events. Our model of gene evolution after WGD is illustrated in Figure 8.

Paralogs exhibit differential rates of evolution and differential shifts in expression patterns (Wagner and Conant, 2003). In our model, pairs of diverging sequences also accumulate differences in gene duplicability, but to a greater extent in genes with narrower expression than those with wide expression patterns. When another WGD event occurs, the widely expressed *AP2*/*ERF* genes are more likely to be retained again and subfunctionalized into various expression patterns in different species. The fact that early WGD gene duplicates are expressed more specifically than those from recent WGD events (Yang and Gaut, 2011) may also be the result of preferential retention of the more widely expressed paralog. In contrast, the *AP2*/*ERF* genes that are already specialized only tended to be duplicated in specific lineages, possibly allowing further adaptation to specialized niches.

### Specialization of AP2/ERF Genes With Broad Abiotic Responses Can Enhance Resistance and Minimize Growth Inhibition

*AP2*/*ERF* genes are important for stress responses and development. For example, *AtCRFs* (1–8) are paralogs from a single ancestral angiosperm gene, with important roles in multiple aspects of plant defense, growth, and development (Kim, 2016). Genes in the SHINE clade activate wax biosynthesis, confer drought tolerance when overexpressed in *Arabidopsis* (Aharoni, 2004), and influence the surface pattern of *Arabidopsis* flower organs (Shi et al., 2011). Also, some AP2/ERF TFs can function as a nexus between stress and development. For example, RAP2.4 acts at, or downstream of, the convergence point of light and ethylene signaling pathways, to coordinately regulate multiple developmental processes and stress responses (Lin et al., 2008). These functions of *AP2*/*ERF* genes in growth and defense may originate from common ancestral functions.

Plants must both grow and defend themselves to survive and reproduce, with trade-offs between growth and defense (Huot et al., 2014). While the deployment of defense mechanisms is imperative for plant survival, defense activation generally comes at the expense of plant growth. Therefore, evolutionarily, plants have had to develop efficient strategies to balance growth and defense. Overexpression of some *AP2*/*ERF* genes in transgenic *Arabidopsis* plants often causes dwarfed phenotypes, because they induce strong expression of target genes under unstressed conditions (Supplementary Table S1) (Licausi et al., 2013). Some *AP2*/*ERF* genes, such as *AtDDF1*, *AtDREB2C*, *ZmDREB2A*, and *OsDREB2B*, are broadly up-regulated by cold, drought, and heat, which may improve plant adaptability to adversity; however, plants may invest in an abiotic defense against one stress factor, but not require defenses against other stressors. Some *AP2*/*ERF* genes are only up-regulated by single abiotic stressors. For example, *AtCBF1–3* are induced under cold stress (Novillo et al., 2004; Kang et al., 2013), while *AtCBF4* gene expression is up-regulated by drought stress (Finn et al., 2016). Overexpression of *AtCBF4* results in constitutive expression of both cold- and drought-inducible *AtCBF1*–3 target genes, and the transgenic plants are more tolerant to freezing and drought stress (Haake et al., 2016); these phenotypes have also been observed for the constitutive overexpression of *AtCBF1–3* (Novillo et al., 2004; Kang et al., 2013; Zhou et al., 2017), indicating that cold and drought resistance may have been the ancestral function of the MRCA of *AtCBF1*–*4*. Therefore, we propose that the response of the plant to cold and drought has evolved from a common CBF-like TF and that, after duplication, *AtCBF1–3* and *AtCBF4* subfunctionalized to become cold- and drought-inducible, respectively. *AtCBF1*, *2*, and *3* also exhibit different patterns of induction in response to low temperatures (Novillo et al., 2004), which may facilitate more precise regulation of cold responses. There are also some specific abiotic-responsive and broadly abiotic-responsive *DREB2* genes in DREB IVa-AOG1 (Figure 8; Supplementary Figure S4F). For example, both *AtDREB2A* and *AtDREB2C* can be up-regulated by heat stress (Chen et al., 2012); however, *AtDREB2C* also has an important role in promoting oxidative stress tolerance in *Arabidopsis* (Hwang et al., 2012). Hence, we speculate that other specific abiotic responsive *AP2*/*ERF* genes may also have evolved from broadly abiotic responsive *AP2*/*ERF* genes by subfunctionalization. Subfunctionalization of broadly abiotic responsive genes can simultaneously result in enhanced resistance to specific abiotic stress and minimize the inhibition of plant growth. Considering the diversity of ecological niches colonized by angiosperms, it is conceivable that having multiple *AP2*/*ERF* paralogs does not merely increase the expression of *AP2*/*ERF* genes, but also leads to the expression of different specialized forms of the *AP2*/*ERF* TFs, facilitating adaption to new environmental conditions.

### Divergence of AP2/ERF Duplicates Facilitates More Sophisticated Regulation of Signaling Networks and Diversification

Some *AP2*/*ERF* duplicates generated by WGD in early core eudicots (γ) are conserved and diversified in their biological functions, such as *CRFs* (Supplementary Figure S7B), which regulate various aspects of plant growth and development, and mediate interactions between hormonal signaling and the environmental stress response (Supplementary Table S1) (Kim, 2016). In contrast, some *AP2*/*ERF* duplicates, corresponding to WGD in early core eudicots (γ) are already involved in different pathways, suggesting functional specialization or neofunctionalization. For example, the two clades *AtEBE*/*AtERF115* and *AtRAP2*.*6L*/*AtERF112* arose due to WGD in early core eudicots (γ) (Supplementary Figure S11B). *ERF115* controls root quiescent center cell division and stem cell replenishment (Heyman et al., 2013). *EBE* influences shoot architecture in *Arabidopsis* (Mehrnia et al., 2013), and *RAP2.6L* functions in graft healing and delayed waterlogging-induced premature senescence (Matsuoka et al., 2018). The spatiotemporal expression of *PUCHI*, *DRN*, and *DRNL* is largely discrete in the inflorescence meristem and early floral meristem; however, they contribute interdependently to cell fate decisions (Chandler and Werr, 2017). The Clade Model C and DIVERGE analyses implemented in this study identified substantial divergent selection in Brassicaceae *AP2*/*ERF* duplicates, showing that retained *AP2*/*ERF* duplicates likely underwent functional divergence, potentially in adapting to various ecological niches (Supplementary Tables S6, S7). Some Brassicaceae-specific *AP2*/*ERF* duplicates have reported differences in expression, protein–protein interaction, and binding preferences (Supplementary Table S9).

Transcription factors are intricately regulated by conformational changes involving allosterism (Phukan et al., 2017). Disordered peptides in proteins can provide conformational flexibility, and such flexible disordered peptides are strongly associated with signaling and regulation (Wright and Dyson, 2014). Some functions of disordered regions have been experimentally verified. Both the N- and C-terminal regions of AP2/ERFs often act as transactivation or repression domains, which regulate expression of downstream genes (Phukan et al., 2017). For example, deletion of the C-terminal intrinsically disordered region of WRI1 affects its stability (Ma et al., 2015). Also, the disordered C-terminal region of TOE1 (294–449) is necessary and sufficient for interaction with the flowering time protein, CO (Zhang et al., 2015). The CRF domain from the N-terminal region of CRFs alone is sufficient for homo- and hetero-dimerization of CRF proteins and for interaction with the histidine-phosphotransfer proteins, AHP1–AHP5 (Cutcliffe et al., 2011). Increased purifying selection means greater functional constraint and a lower evolutionary rate, thus changes in evolutionary rate can be interpreted as “changes in functional constraints,” which is an indication of functional divergence (Gu et al., 2013). We found that disordered regions have higher evolutionary rates than ordered regions, suggesting that duplicates may diverge in these regions. Thus, mutations could contribute to structural flexibility, conferring a selective advantage in novel regulatory mechanisms, such as interactions and post-translational modifications. For example, TOE1 can interact with the related CO, COL1, COL2, COL3, and COL5 proteins; however, TOE2 can only interact with COL1 and COL5 (Zhang et al., 2015). In addition, among the proteins CRF1–CRF6, only CRF2 and CRF3 do not interact with AHP2 (Cutcliffe et al., 2011). Additional studies in the near future will likely further improve the understanding of the function and evolution of *AP2*/*ERF* genes and their impact on development and environmental responses.

## Conclusion

Our study reveals the evolutionary landscape of the *AP2*/*ERF* family in angiosperms. We found that AP2/ERF protein sequences can be classified into 75 AOGs. For most AOGs, we observed a highly consistent pattern of gene duplicability within the same AOG across species. Specifically, one group (multi-copy) comprising 21 of 75 AOGs has experienced significant expansion in multiple angiosperm lineages, except the basal angiosperms and basal eudicots. In contrast, a second group (low-copy), comprising the other 54 AOGs, tends to have maintained a low-copy status, with some having only a few lineage-specific duplicates and others with lineage-specific losses. We also found that multi-copy genes did not evolve more rapidly than low-copy genes, and that multi-copy genes are expressed with lower specificity than low-copy genes. Moreover, in gene groups with two successive duplications, between two older *AP2*/*ERF* duplicates, the one with relatively broad expression and slower evolutionary rates was more likely to retain both younger duplicates. Duplicates of widely expressed and slower evolving *AP2*/*ERF* genes are retained repeatedly and relatively often, and continue to experience functional constraint. One possible explanation is that the widely expressed *AP2*/*ERF* genes may have a greater chance of subfunctionalization (e.g., tissue specialization, specific responses) following duplication, and the newly subfunctionalized genes can facilitate more precise regulation of signaling networks or adaption to new environmental conditions. *AP2*/*ERF* genes that were already specialized (narrower expression) tended to be duplicated in specific lineages, possibly allowing further adaptation to special niches, or lost in some lineages, which changed their ecological niches.

## Author Contributions

HM and JL designed the study. LW carried out evolutionary analysis. LW and JL drafted the manuscript and HM revised the manuscript. All authors read and approved the final manuscript.

## Conflict of Interest Statement

The authors declare that the research was conducted in the absence of any commercial or financial relationships that could be construed as a potential conflict of interest.

## Footnotes

1 https://phytozome.jgi.doe.gov/pz/portal.html

2 https://genome.ucsc.edu/

3 http://thellungiella.org/

4 http://lotus-db.wbgcas.cn

5 https://www.sunflowergenome.org

6 http://hmmer.janelia.org/

7 http://pfam.sanger.ac.uk/

8 http://www.ncbi.nlm.nih.gov/Structure/cdd/wrpsb.cgi

9 http://test.datamonkey.org/

## Supplementary Material

Figure S1 Phylogenetic trees of the *AP2* subfamily from representative Angiosperms. SH-aLRT supports (approximate likelihood-ratio test and relies on a nonparametric, Shimodaira–Hasegawa–like procedure) above 50% are labeled on internal nodes. The MRCA (circles) and gene duplication events (stars) of each major plant group (Brassicaceae, Fabaceae, Asteraceae, core eudicots, Poaceae, Poales, Commelinids, Monocots, and Angiosperms) are indicated on internal nodes in different colors (green, light blue, purple, red, orange, deep blue, blue, deep red, and black). The numbers in the top left textbox represent the minimum inferred AP2/ERF protein complement and gene duplication events in the last common ancestor of each major plant group.

Figure S2 Phylogenetic trees of the DREB I subfamily from representative Angiosperms. SH-aLRT supports above 50% are labeled on internal nodes. The labeling is the same as in Supplementary Figure S1.

Figure S3 Phylogenetic trees of the DREB II subfamily from representative Angiosperms. SH-aLRT supports above 50% are labeled on internal nodes. The labeling is the same as in Supplementary Figure S1.

Figure S4 Phylogenetic trees of DREB III subfamily from representative Angiosperms. SH-aLRT supports above 50% are labeled on internal nodes. The labeling is the same as in Supplementary Figure S1.

Figure S5 Phylogenetic trees of DREB IV subfamily from representative Angiosperms. SH-aLRT supports above 50% are labeled on internal nodes. The labeling is the same as in Supplementary Figure S1.

Figure S6 Phylogenetic trees of ERF V subfamily from representative Angiosperms. SH-aLRT supports above 50% are labeled on internal nodes. The labeling is the same as in Supplementary Figure S1.

Figure S7 Phylogenetic trees of ERF VI subfamily from representative Angiosperms. SH-aLRT supports above 50% are labeled on internal nodes. The labeling is the same as in Supplementary Figure S1.

Figure S8 Phylogenetic trees of ERF VII subfamily from representative Angiosperms. SH-aLRT supports above 50% are labeled on internal nodes. The labeling is the same as in Supplementary Figure S1.

Figure S9 Phylogenetic trees of ERF VIII subfamily from representative Angiosperms. SH-aLRT supports above 50% are labeled on internal nodes. The labeling is the same as in Supplementary Figure S1.

Figure S10 Phylogenetic trees of ERF IX subfamily from representative Angiosperms. SH-aLRT supports above 50% are labeled on internal nodes. The labeling is the same as in Supplementary Figure S1.

Figure S11 Phylogenetic trees of ERF X subfamily from representative Angiosperms. SH-aLRT supports above 50% are labeled on internal nodes. The labeling is the same as in Supplementary Figure S1.

Figure S12 Phylogenetic trees of RAV subfamily from representative Angiosperms. SH-aLRT supports above 50% are labeled on internal nodes. The labeling is the same as in Supplementary Figure S1.

Figure S13 A phylogenetic tree of Soloist from representative Angiosperms. SH-aLRT supports above 50% are labeled on internal nodes. The labeling is the same as in Supplementary Figure S1.

Figure S14 Boxplots showing Ka, Ks, and ω (Ka/Ks) values for each duplicated clades generated near the origin of Brassicaceae. The duplicates pairs generated near the origin of Brassicaceae are separated into D1 and D2 according to their copy number of *AP2*/*REF* genes from *L. alabamica* **(A–E)** or *B. rapa* **(F–J)**, with the D1 having more gene copy in *L. alabamica* **(A–E)** or *B. rapa* **(F–J)**. All Ka, Ks, and ω (Ka/Ks) values were calculated by comparison of orthologous pairs from selected species. Values in **A–C** and **F–H** were calculated by comparison of orthologous pairs between *A. thaliana* and *B. stricta* sequence. Values in **D–F** and **I–K** were calculated by comparison of orthologous pairs between *A. thaliana* and *E. salsuginea* sequence. Values were compared using the Wilcoxon rank signed rank tests and *P*-values are shown above each pair of boxplots.

Figure S15 *Cis*-regulatory element partitioning among duplicates. **(A)** Subfunction measure (Sub) and asymmetry measure (Asy) definition. Asy was calculated based on the number of *cis*-regulatory element. Extreme asymmetry was defined based on Asy = 1. Sub was calculated based on the intersection and union of *cis*-regulatory element of duplicate pairs. Extreme subfunctionalization was defined based on Sub = 0. The *cis*-regulatory element of each genes is de-duplicated, before the Sub was calculated. **(B)** Relative frequencies of the number of duplicate pairs with various degrees of asymmetric partitioning. **(C)** Relative frequencies of the number of duplicate pairs with various degrees of subfunctionalized partitioning. Majority of *cis*-regulatory element of extant genes was assumed to be ancestral, new *cis*-regulatory element was assumed to be very little.

Figure S16 Gene expression data from At-TAX tilling array in 11 tissues of *Arabidopsis AP2/ERF* genes. (1) Roots, (2) seedlings, (3) expanding leaves, (4) senescing leaves, (5) stem, (6) vegetative shoot meristem, (7) inflorescence shoot meristem, (8) whole inflorescences, (9) whole inflorescences (clv3-7), (10) flowers, and (11) fruits. Brassicaceae-specific *AP2*/*ERF* paralogs were indicated by green left brackets. AOGs duplicates corresponding core eudicots WGD (γ) and Brassicaceae WGD (α/β) were indicated by red brackets. Values to the left of brackets are Pearson’s correlation coefficients for correlated expression between Brassicaceae-specific *AP2*/*ERF* paralogs.

Figure S17 Normalized evolutionary rates per site mapped onto the amino acid sequence. All evolutionary rates were normalized with a mean of zero and standard deviation of one. The confidence interval for the rate estimates was show as error bar. The AP2 domain were colored as cornsilk, the B3 domain were colored as light yellow. Coiled coil region was colored as green. Positive selected sites were indicated by red point; Type I functional divergence amino acid sites that are highly conserved were indicated by yellow point; Type I functional divergence amino acid sites that are variable were indicated by skyblue point. Blue braces represent subfamilies; red brace represent Brassicaceae-specific paralogous pairs.

Figure S18 The region besides AP2 domain is intrinsically disordered. Disorder states are indicated as blue lines; protein-binding disordered residues are indicated as orange lines. Blue braces represent subfamilies; red brace represents Brassicaceae-specific paralogous pairs. The AP2 domain were colored as cornsilk, the B3 domain were colored as lightyellow.

Figure S19 Violin plots of evolutionary rates per site. The median number of evolutionary rates are shown by the red dot. Values were compared using the Wilcoxon rank-sum test and *P*-values are shown above the Violin plots.

Figure S20 MAFFT alignment of ERF VII proteins from representative species with a N-terminal MC-motif (Met–Cys–Gly–Gly–Ala–Ile/Leu/Val, MCGGAI/L/V).

Table S1 Functionally characterized divergences of *AP2*/*ERF* gene from *Arabidopsis*.

Table S2 Taxa included in this study and data collection.

Table S3 A list of *AP2*/*RF* genes included in this study.

Table S4 Test for shifts in selective pressures Using RELAX (Wertheim et al., 2015). The parameter *k* is the estimated selection intensity; values under and above 1 indicate relaxed and intensified selection in relative Branch.

Table S5 RELAX model fits. ω distributions under the Null model and the Alternative model.

Table S6 Estimates of parameters used in codon-substitution evolutionary models for *AP2*/*ERF* paralogs in Brassicaceae.

Table S7 Analysis of FD by DIVERGE.

Table S8 Sites with Type I functional divergence (FD-I) in AP2/ERF functional diversification.

Table S9 Functionally characterized divergences of *AP2*/*ERF* genes from *A. thaliana*.
