# Supplementary material for: Angiosperm-Wide and Family-Level Analyses of AP2/ERF Genes Reveal Differential Retention and Sequence Divergence After Whole-Genome Duplication
Source: Front Plant Sci. 2019 Feb 26;10:196. doi: 10.3389/fpls.2019.00196 (PMC6399210; doi:10.3389/fpls.2019.00196)

S1a

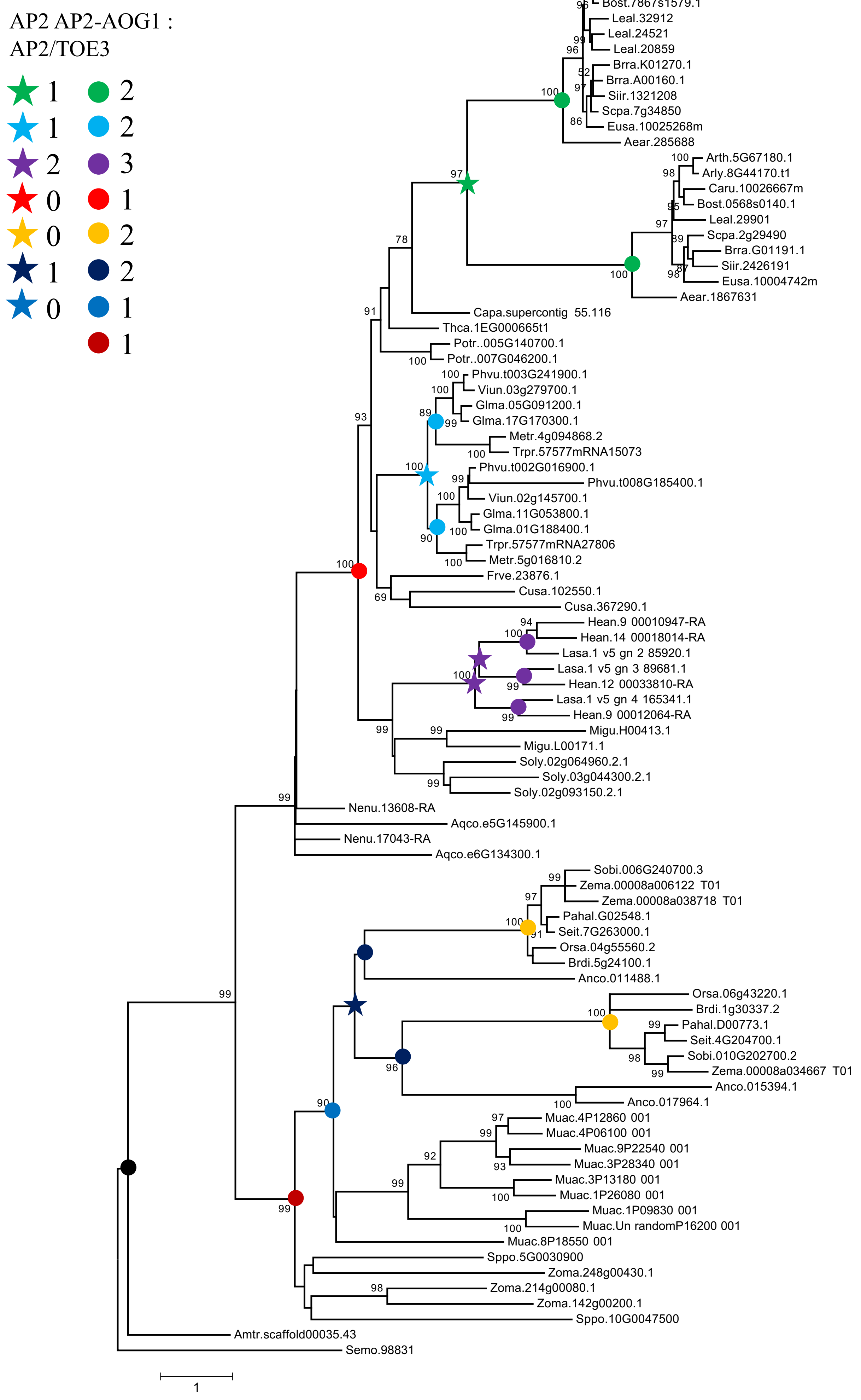

AP2 AP2-AOG1 :
AP2/TOE3
1 2
1 2
2 3
0 1
0 2
1 2
0 1
1
Arth.4G36920.1
Arly.7G13680.t1
Caru.10004818m
Bost.7867s1579.1
Leal.32912
Leal.24521
Leal.20859
Brra.K01270.1
Brra.A00160.1
Siir.1321208
Scpa.7g34850
Eusa.10025268m
Aear.285688
Arth.5G67180.1
Arly.8G44170.t1
Caru.10026667m
Bost.0568s0140.1
Leal.29901
Scpa.2g29490
Brra.G01191.1
Siir.2426191
Eusa.10004742m
Aear.1867631
Capa.supercontig 55.116
Thca.1EG000665t1
Potr..005G140700.1
Potr..007G046200.1
Phvu.t003G241900.1
Viun.03g279700.1
Glma.05G091200.1
Glma.17G170300.1
Metr.4g094868.2
Trpr.57577mRNA15073
Phvu.t002G016900.1
Phvu.t008G185400.1
Viun.02g145700.1
Glma.11G053800.1
Glma.01G188400.1
Trpr.57577mRNA27806
Metr.5g016810.2
Frve.23876.1
Cusa.102550.1
Cusa.367290.1
Hean.9 00010947-RA
Hean.14 00018014-RA
Lasa.1 v5 gn 2 85920.1
Lasa.1 v5 gn 3 89681.1
Hean.12 00033810-RA
Lasa.1 v5 gn 4 165341.1
Hean.9 00012064-RA
Migu.H00413.1
Migu.L00171.1
Soly.02g064960.2.1
Soly.03g044300.2.1
Soly.02g093150.2.1
Nenu.13608-RA
Aqco.e5G145900.1
Nenu.17043-RA
Aqco.e6G134300.1
Sobi.006G240700.3
Zema.00008a006122 T01
Zema.00008a038718 T01
Pahal.G02548.1
Seit.7G263000.1
Orsa.04g55560.2
Brdi.5g24100.1
Anco.011488.1
Orsa.06g43220.1
Brdi.1g30337.2
Pahal.D00773.1
Seit.4G204700.1
Sobi.010G202700.2
Zema.00008a034667 T01
Anco.015394.1
Anco.017964.1
Muac.4P12860 001
Muac.4P06100 001
Muac.9P22540 001
Muac.3P28340 001
Muac.3P13180 001
Muac.1P26080 001
Muac.1P09830 001
Muac.Un randomP16200 001
Muac.8P18550 001
Sppo.5G0030900
Zoma.248g00430.1
Zoma.214g00080.1
Zoma.142g00200.1
Sppo.10G0047500
Amtr.scaffold00035.43
Semo.98831
1

S1b

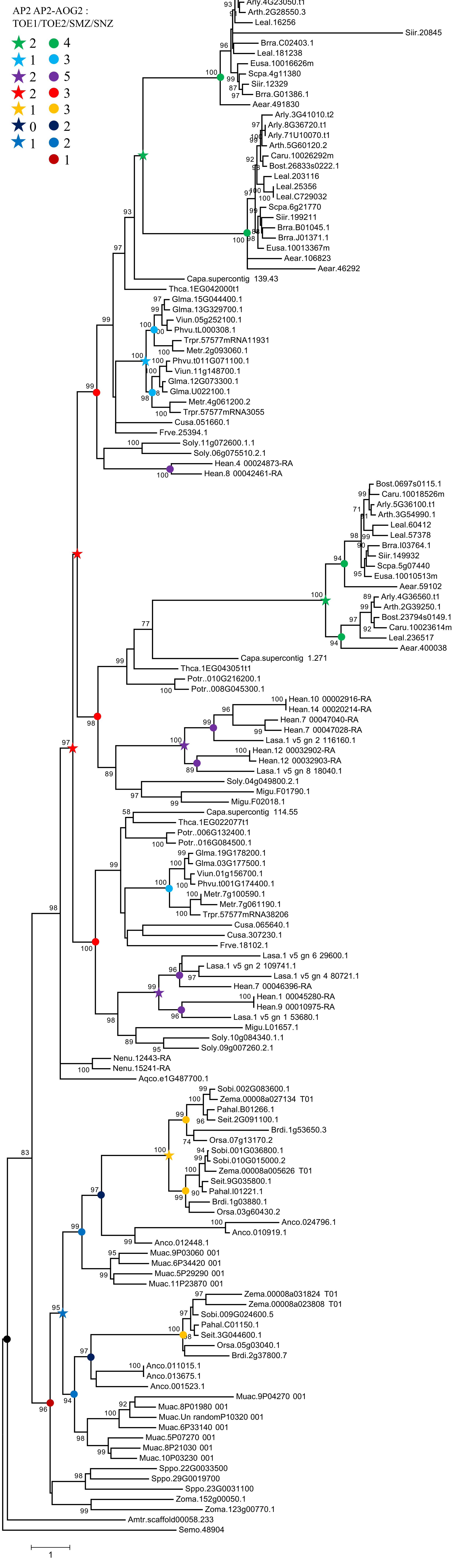
AP2 AP2-AOG2 :
TOE1/TOE2/SMZ/SNZ
Bost.27991s0069.1
Caru.10025387m
Arly.4G23050.t1
Arth.2G28550.3
Leal.16256
Siir.20845
Brra.C02403.1
Leal.181238
Eusa.10016626m
Scpa.4g11380
Siir.12329
Brra.G01386.1
Aear.491830
Arly.3G41010.t2
Arly.8G36720.t1
Arly.71U10070.t1
Arth.5G60120.2
Caru.10026292m
Bost.26833s0222.1
Leal.203116
Leal.25356
Leal.C729032
Scpa.6g21770
Siir.199211
Brra.B01045.1
Brra.J01371.1
Eusa.10013367m
Aear.106823
Aear.46292
Capa.supercontig 139.43
Thca.1EG042000t1
Glma.15G044400.1
Glma.13G329700.1
Viun.05g252100.1
Phvu.tL000308.1
Trpr.57577mRNA11931
Metr.2g093060.1
Phvu.t011G071100.1
Viun.11g148700.1
Glma.12G073300.1
Glma.U022100.1
Metr.4g061200.2
Trpr.57577mRNA3055
Cusa.051660.1
Frve.25394.1
Soly.11g072600.1.1
Soly.06g075510.2.1
Hean.4 00024873-RA
Hean.8 00042461-RA
Bost.0697s0115.1
Caru.10018526m
Arly.5G36100.t1
Arth.3G54990.1
Leal.60412
Leal.57378
Brra.I03764.1
Siir.149932
Scpa.5g07440
Eusa.10010513m
Aear.59102
Arly.4G36560.t1
Arth.2G39250.1
Bost.23794s0149.1
Caru.10023614m
Leal.236517
Aear.400038
Capa.supercontig 1.271
Thca.1EG043051t1
Potr..010G216200.1
Potr..008G045300.1
Hean.10 00002916-RA
Hean.14 00020214-RA
Hean.7 00047040-RA
Hean.7 00047028-RA
Lasa.1 v5 gn 2 116160.1
Hean.12 00032902-RA
Hean.12 00032903-RA
Lasa.1 v5 gn 8 18040.1
Soly.04g049800.2.1
Migu.F01790.1
Migu.F02018.1
Capa.supercontig 114.55
Thca.1EG022077t1
Potr..006G132400.1
Potr..016G084500.1
Glma.19G178200.1
Glma.03G177500.1
Viun.01g156700.1
Phvu.t001G174400.1
Metr.7g100590.1
Metr.7g061190.1
Trpr.57577mRNA38206
Cusa.065640.1
Cusa.307230.1
Frve.18102.1
Lasa.1 v5 gn 6 29600.1
Lasa.1 v5 gn 2 109741.1
Lasa.1 v5 gn 4 80721.1
Hean.7 00046396-RA
Hean.1 00045280-RA
Hean.9 00010975-RA
Lasa.1 v5 gn 1 53680.1
Migu.L01657.1
Soly.10g084340.1.1
Soly.09g007260.2.1
Nenu.12443-RA
Nenu.15241-RA
Aqco.e1G487700.1
Sobi.002G083600.1
Zema.00008a027134 T01
Pahal.B01266.1
Seit.2G091100.1
Brdi.1g53650.3
Orsa.07g13170.2
Sobi.001G036800.1
Sobi.010G015000.2
Zema.00008a005626 T01
Seit.9G035800.1
Pahal.I01221.1
Brdi.1g03880.1
Orsa.03g60430.2
Anco.024796.1
Anco.010919.1
Anco.012448.1
Muac.9P03060 001
Muac.6P34420 001
Muac.5P29290 001
Muac.11P23870 001
Zema.00008a031824 T01
Zema.00008a023808 T01
Sobi.009G024600.5
Pahal.C01150.1
Seit.3G044600.1
Orsa.05g03040.1
Brdi.2g37800.7
Anco.011015.1
Anco.013675.1
Anco.001523.1
Muac.9P04270 001
Muac.8P01980 001
Muac.Un randomP10320 001
Muac.6P33140 001
Muac.5P07270 001
Muac.8P21030 001
Muac.10P03230 001
Sppo.22G0033500
Sppo.29G0019700
Sppo.23G0031100
Zoma.152g00050.1
Zoma.123g00770.1
Amtr.scaffold00058.233
Semo.48904
1

S1c

AP2 ANT-AOG1:
AP2.01

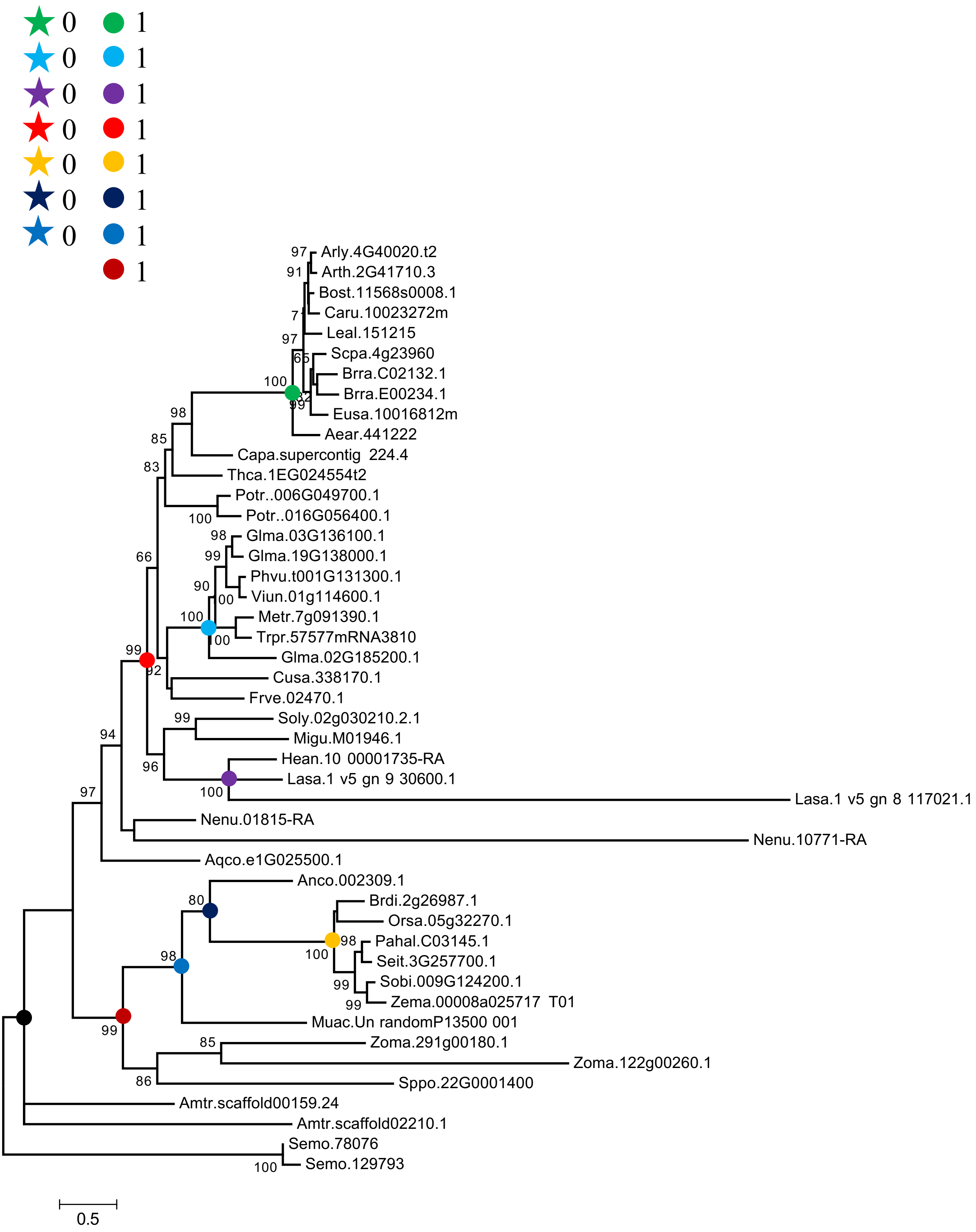

S1d

AP2 ANT-AOG2

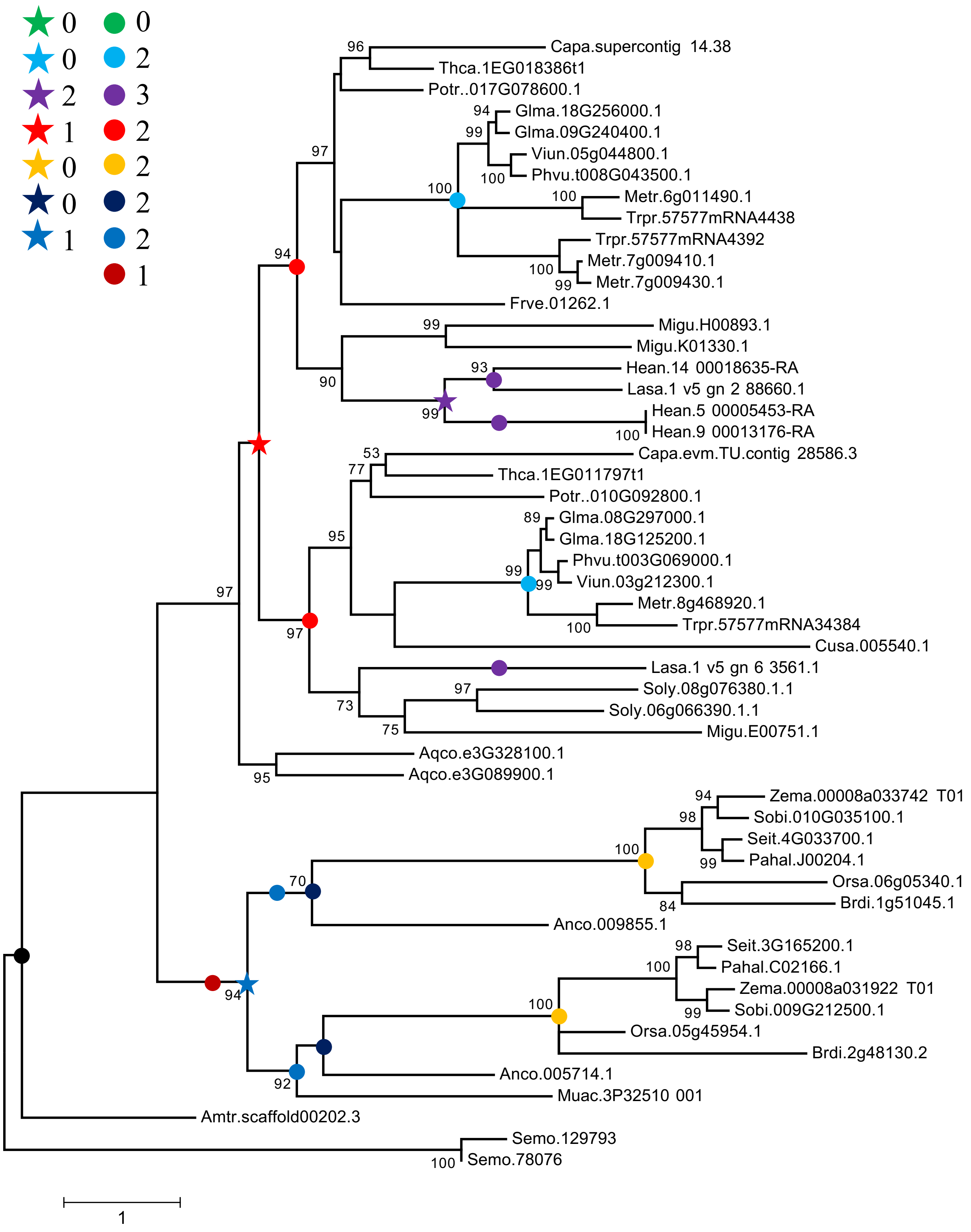

S1e

AP2 ANT-AOG3:
WRI1

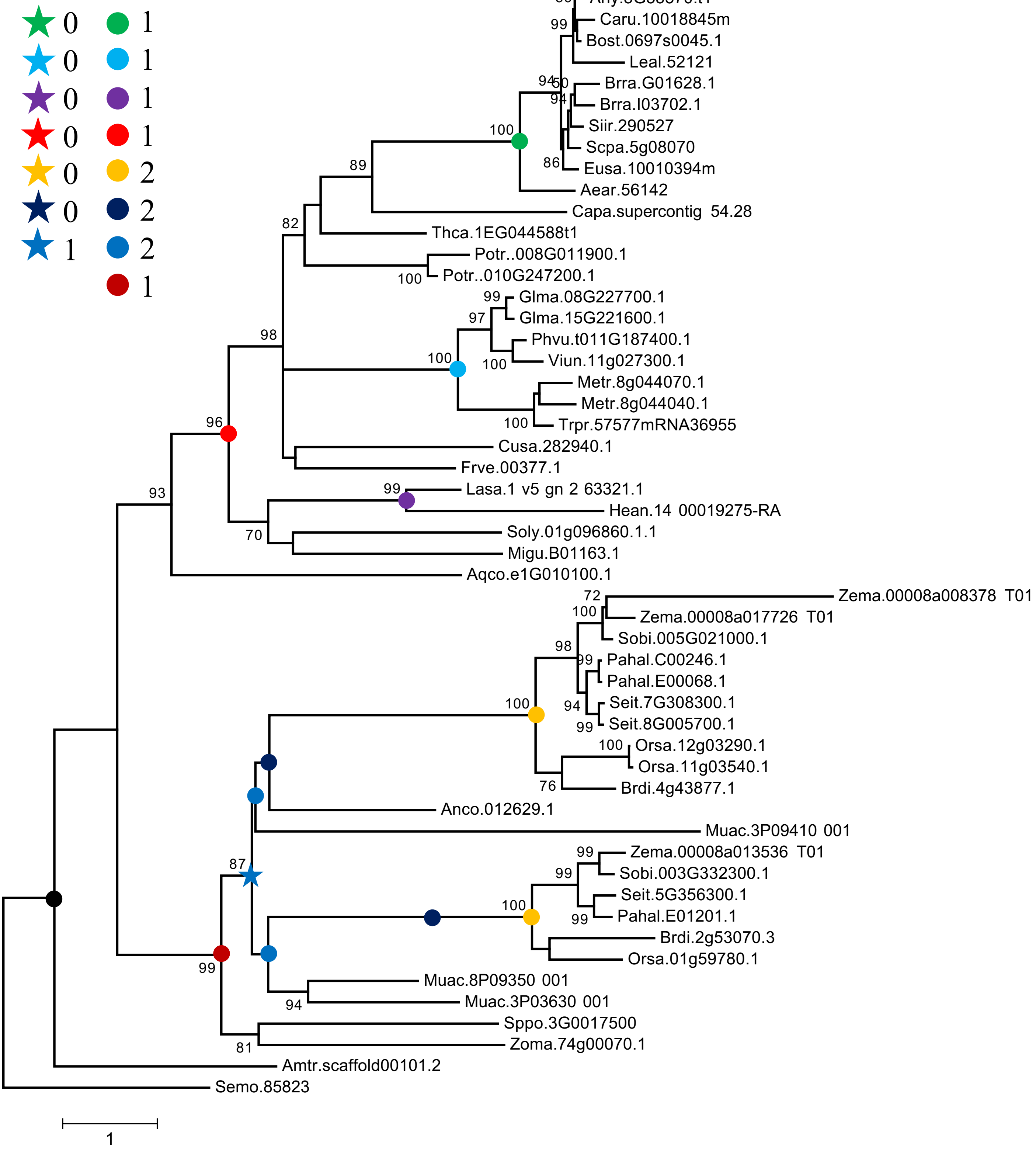

S1f

AP2 ANT-AOG4:
WRI3/WRI3

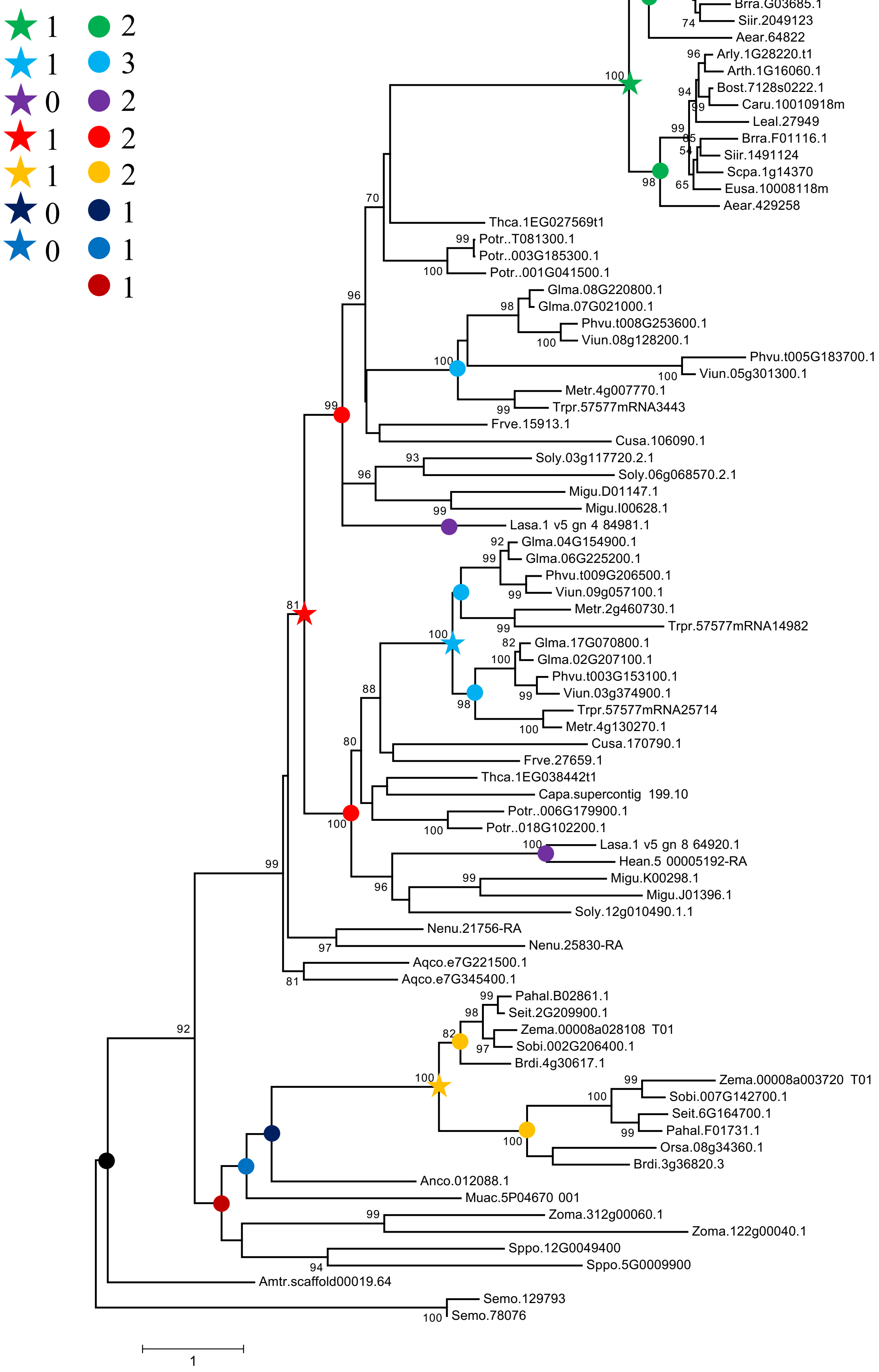

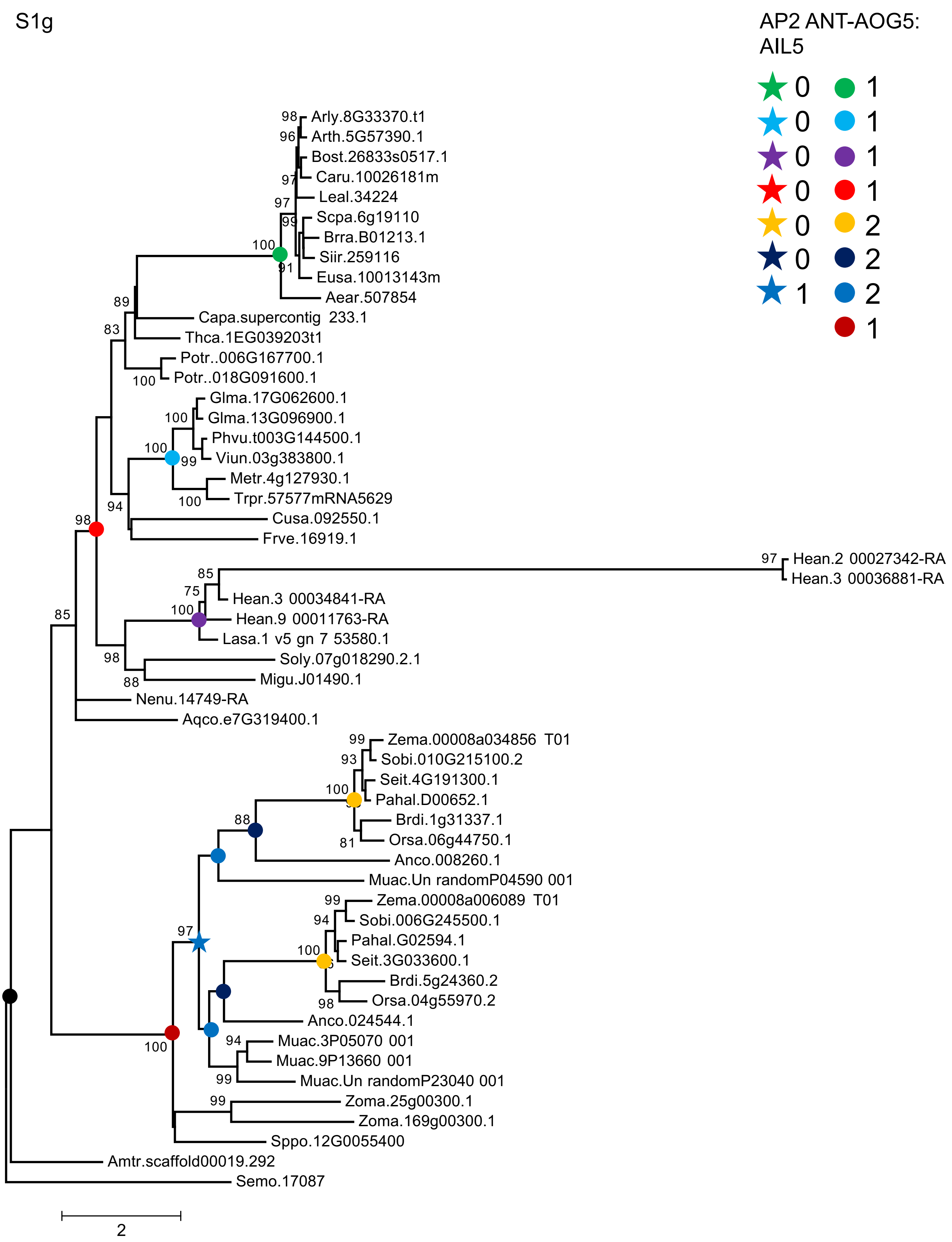
S1g
AP2 ANT-AOG5:
AIL5
0 1
0 1
0 1
0 1
0 2
0 2
1 2
1
Arly.8G33370.t1
Arth.5G57390.1
Bost.26833s0517.1
Caru.10026181m
Leal.34224
Scpa.6g19110
Brra.B01213.1
Siir.259116
Eusa.10013143m
Aear.507854
Capa.supercontig 233.1
Thca.1EG039203t1
Potr..006G167700.1
Potr..018G091600.1
Glma.17G062600.1
Glma.13G096900.1
Phvu.t003G144500.1
Viun.03g383800.1
Metr.4g127930.1
Trpr.57577mRNA5629
Cusa.092550.1
Frve.16919.1
Hean.2 00027342-RA
Hean.3 00036881-RA
Hean.3 00034841-RA
Hean.9 00011763-RA
Lasa.1 v5 gn 7 53580.1
Soly.07g018290.2.1
Migu.J01490.1
Nenu.14749-RA
Aqco.e7G319400.1
Zema.00008a034856 T01
Sobi.010G215100.2
Seit.4G191300.1
Pahal.D00652.1
Brdi.1g31337.1
Orsa.06g44750.1
Anco.008260.1
Muac.Un randomP04590 001
Zema.00008a006089 T01
Sobi.006G245500.1
Pahal.G02594.1
Seit.3G033600.1
Brdi.5g24360.2
Orsa.04g55970.2
Anco.024544.1
Muac.3P05070 001
Muac.9P13660 001
Muac.Un randomP23040 001
Zoma.25g00300.1
Zoma.169g00300.1
Sppo.12G0055400
Amtr.scaffold00019.292
Semo.17087
2

S1h

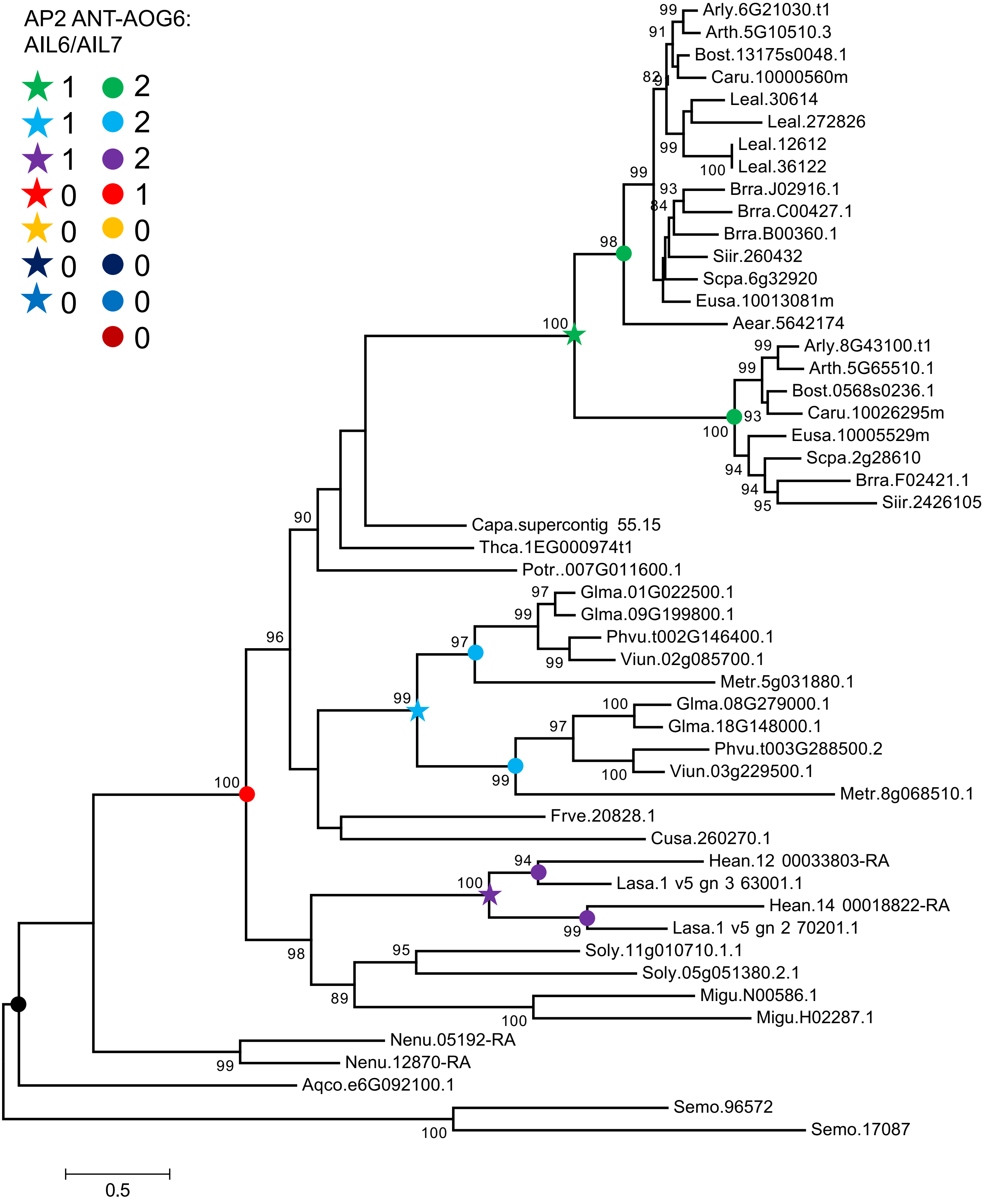

AP2 ANT-AOG6:
AIL6/AIL7
1 2
1 2
1 2
0 1
0 0
0 0
0 0
0
Arly.6G21030.t1
Arth.5G10510.3
Bost.13175s0048.1
Caru.10000560m
Leal.30614
Leal.272826
Leal.12612
Leal.36122
Brra.J02916.1
Brra.C00427.1
Brra.B00360.1
Siir.260432
Scpa.6g32920
Eusa.10013081m
Aear.5642174
Arly.8G43100.t1
Arth.5G65510.1
Bost.0568s0236.1
Caru.10026295m
Eusa.10005529m
Scpa.2g28610
Brra.F02421.1
Siir.2426105
Capa.supercontig 55.15
Thca.1EG000974t1
Potr..007G011600.1
Glma.01G022500.1
Glma.09G199800.1
Phvu.t002G146400.1
Viun.02g085700.1
Metr.5g031880.1
Glma.08G279000.1
Glma.18G148000.1
Phvu.t003G288500.2
Viun.03g229500.1
Metr.8g068510.1
Frve.20828.1
Cusa.260270.1
Hean.12 00033803-RA
Lasa.1 v5 gn 3 63001.1
Hean.14 00018822-RA
Lasa.1 v5 gn 2 70201.1
Soly.11g010710.1.1
Soly.05g051380.2.1
Migu.N00586.1
Migu.H02287.1
Nenu.05192-RA
Nenu.12870-RA
Aqco.e6G092100.1
Semo.96572
Semo.17087
0.5

S1i

AP2 ANT-AOG7:
AIL1

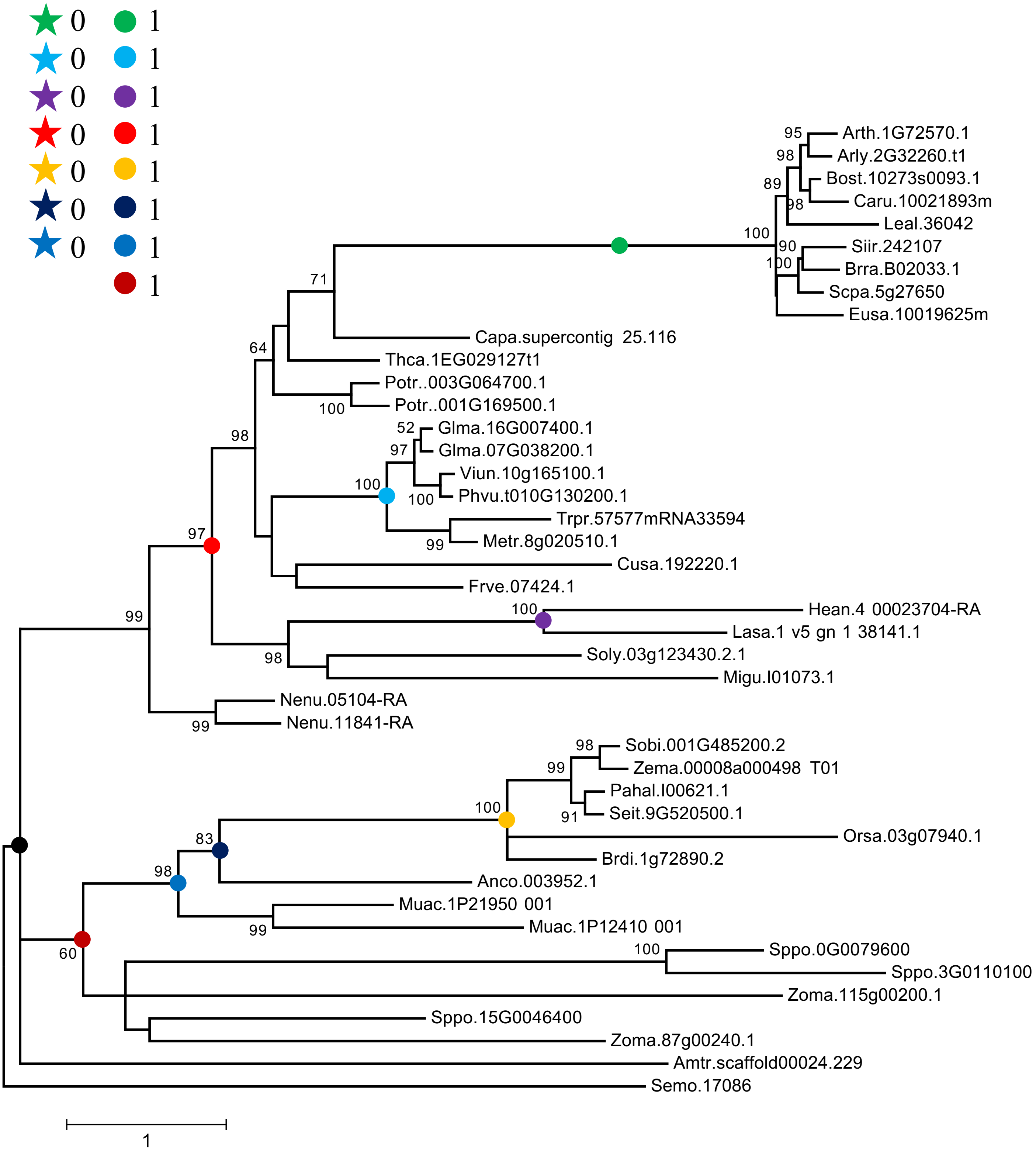

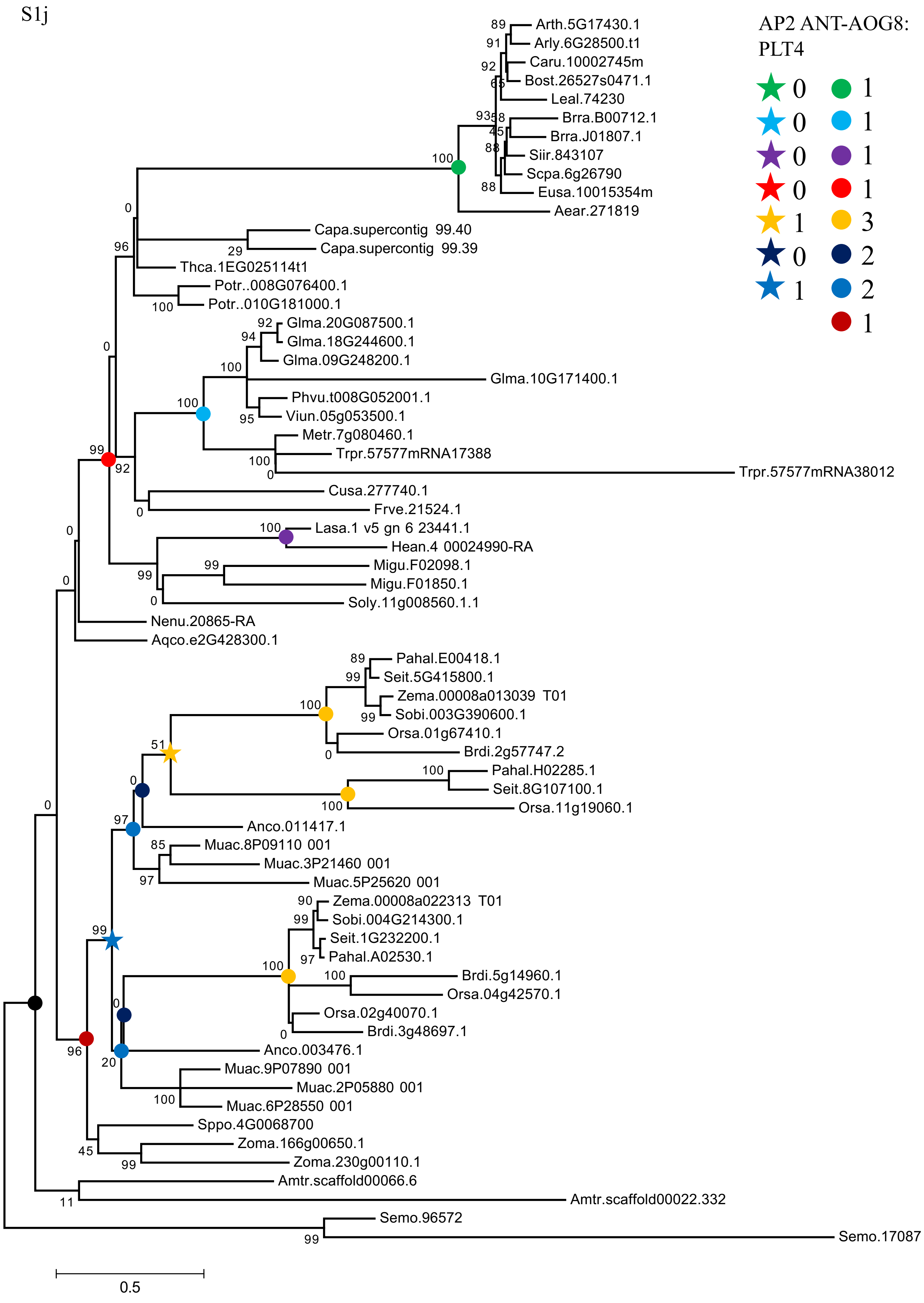
S1j
AP2 ANT-AOG8:
PLT4
0
1
0
1
0
1
0
1
1
3
0
2
1
2
1
Arth.5G17430.1
Arly.6G28500.t1
Caru.10002745m
Bost.26527s0471.1
Leal.74230
Brra.B00712.1
Brra.J01807.1
Siir.843107
Scpa.6g26790
Eusa.10015354m
Aear.271819
Capa.supercontig 99.40
Capa.supercontig 99.39
Thca.1EG025114t1
Potr..008G076400.1
Potr..010G181000.1
Glma.20G087500.1
Glma.18G244600.1
Glma.09G248200.1
Glma.10G171400.1
Phvu.t008G052001.1
Viun.05g053500.1
Metr.7g080460.1
Trpr.57577mRNA17388
Trpr.57577mRNA38012
Cusa.277740.1
Frve.21524.1
Lasa.1 v5 gn 6 23441.1
Hean.4 00024990-RA
Migu.F02098.1
Migu.F01850.1
Soly.11g008560.1.1
Nenu.20865-RA
Aqco.e2G428300.1
Pahal.E00418.1
Seit.5G415800.1
Zema.00008a013039 T01
Sobi.003G390600.1
Orsa.01g67410.1
Brdi.2g57747.2
Pahal.H02285.1
Seit.8G107100.1
Orsa.11g19060.1
Anco.011417.1
Muac.8P09110 001
Muac.3P21460 001
Muac.5P25620 001
Zema.00008a022313 T01
Sobi.004G214300.1
Seit.1G232200.1
Pahal.A02530.1
Brdi.5g14960.1
Orsa.04g42570.1
Orsa.02g40070.1
Brdi.3g48697.1
Anco.003476.1
Muac.9P07890 001
Muac.2P05880 001
Muac.6P28550 001
Sppo.4G0068700
Zoma.166g00650.1
Zoma.230g00110.1
Amtr.scaffold00066.6
Amtr.scaffold00022.332
Semo.96572
Semo.17087
0.5

S1k

AP2 ANT-AOG9:
PLT1/PLT2

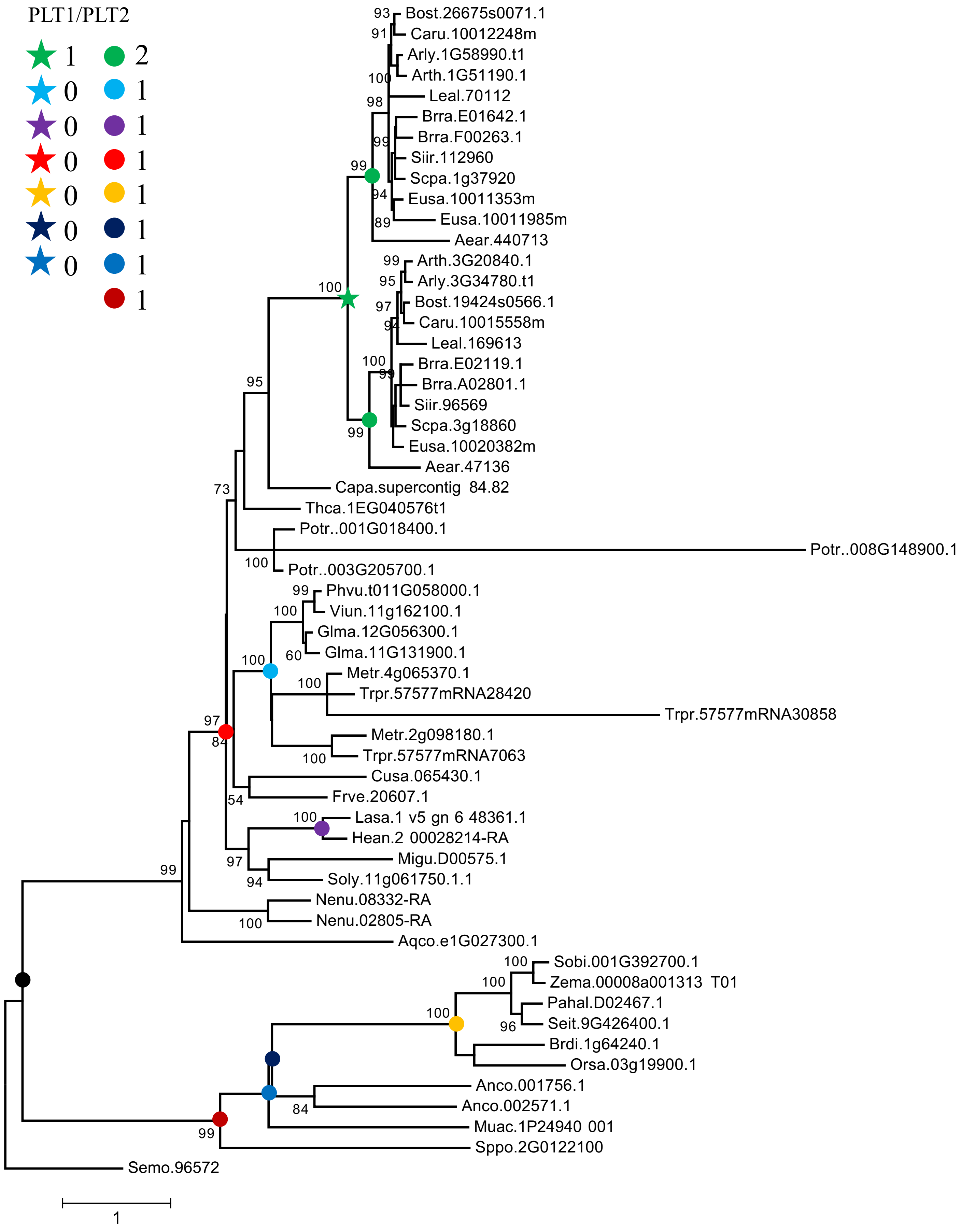

S11

AP2 ANT-AOG10

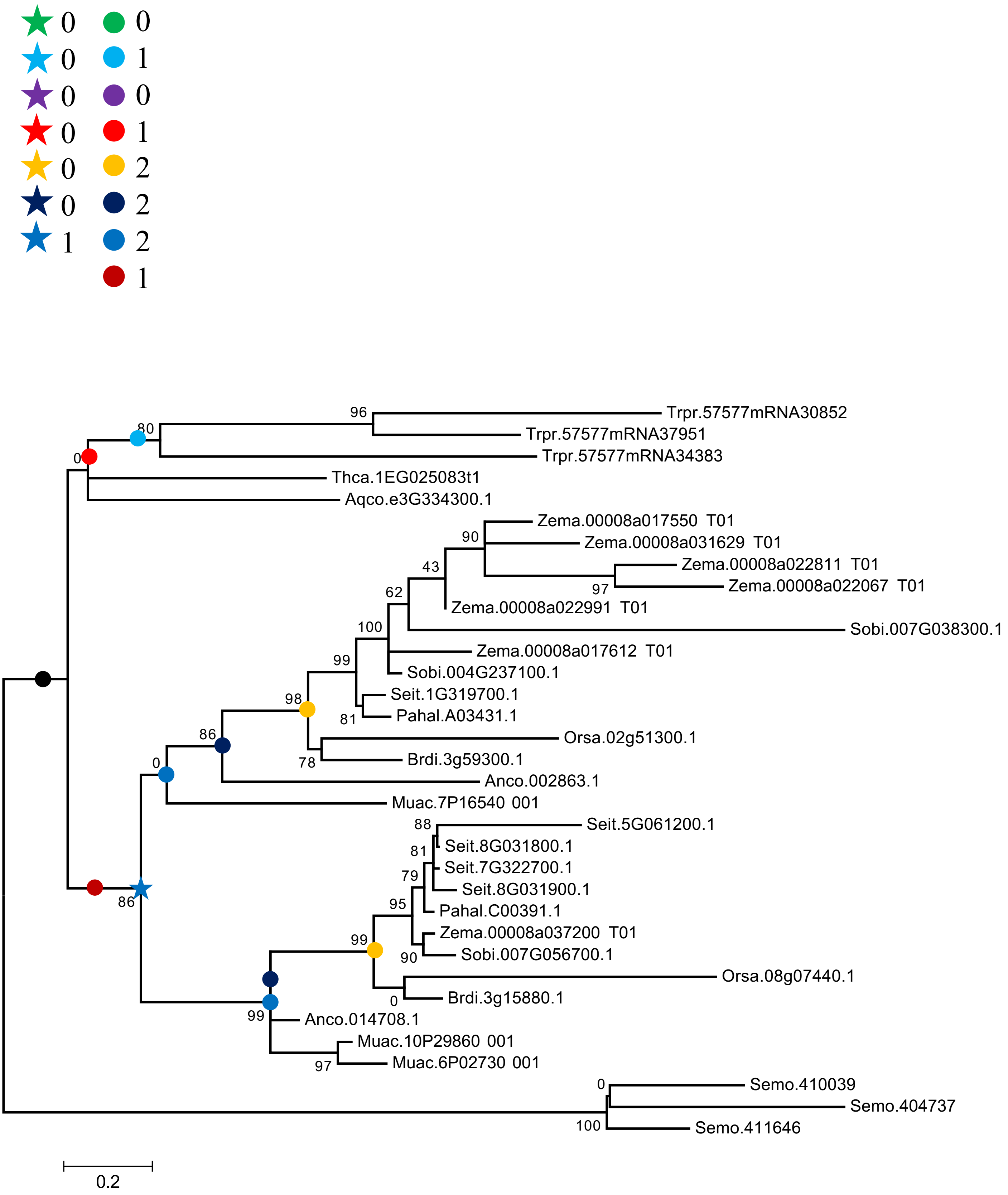

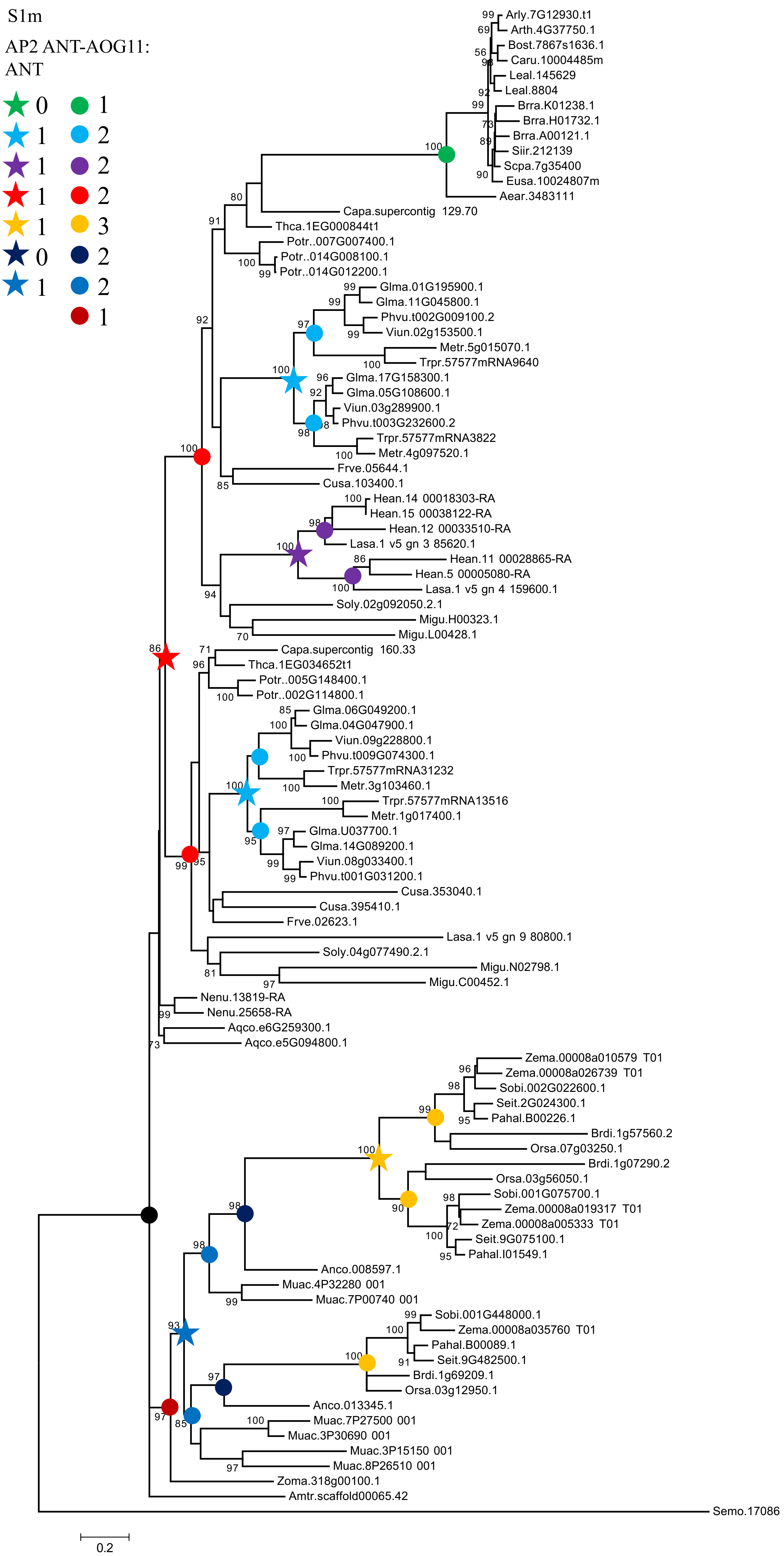

Fig. S1 Phylogenetic trees of the *AP2* subfamily from representative Angiosperms. SH-aLRT supports (Approximate likelihood-ratio test and relies on a nonparametric, Shimodaira–Hasegawa–like procedure) above 50% are labeled on internal nodes. The most recent common ancestor (circles) and gene duplication events (stars) of each major plant group (Brassicaceae, Fabaceae, Asteraceae, core eudicots, Poaceae, Poales, Commelinids, Moncots and Angiosperms) are indicated on internal nodes in different colors (green, light blue, purple, red, orange, deep blue, blue, deep red and black). The numbers in the top left textbox represent the minimum inferred AP2/ERF protein complement and gene duplication events in the last common ancestor of each major plant group.

S2a

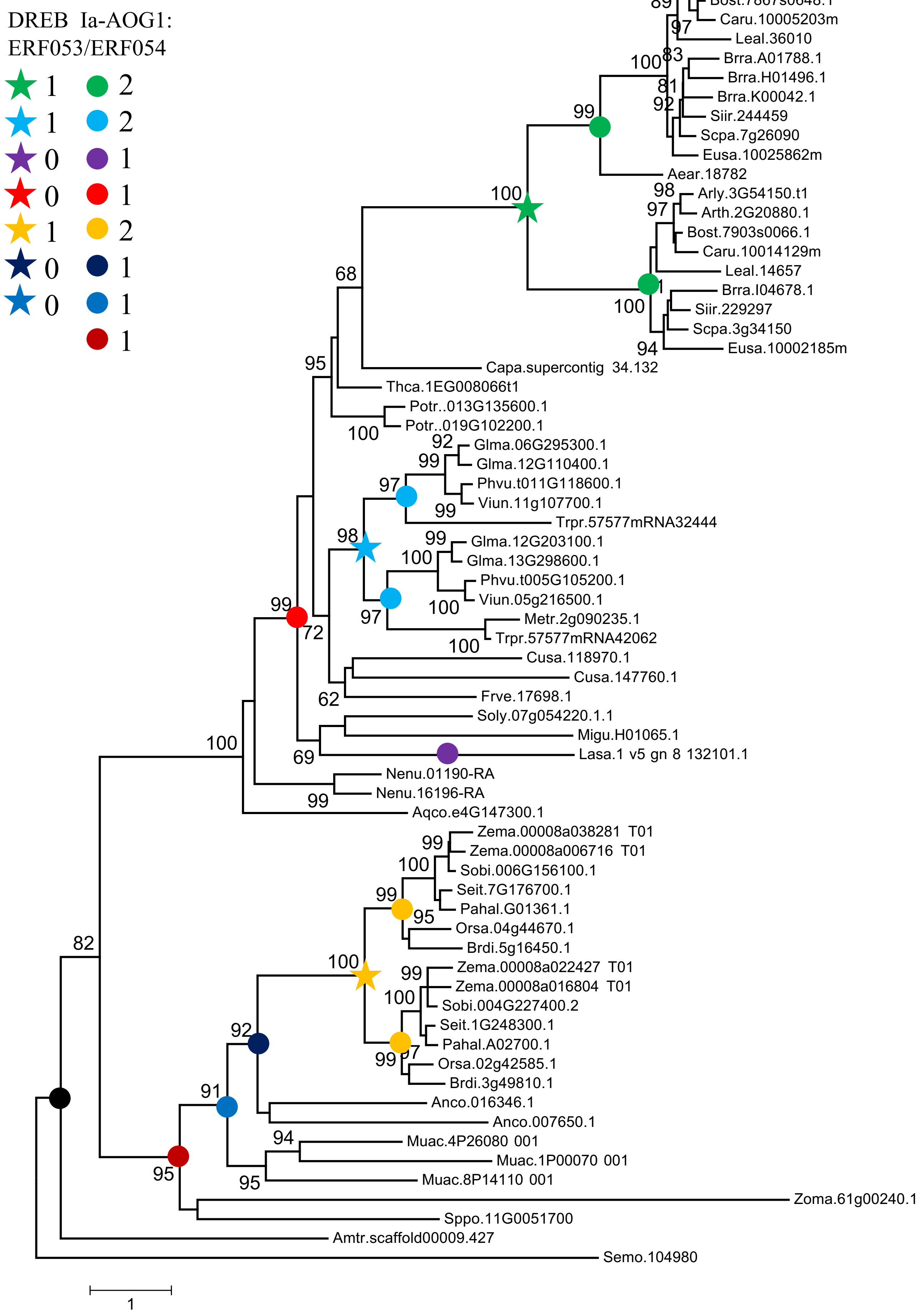

DREB Ia-AOG1:
ERF053/ERF054
1 2
1 2
0 1
0 1
1 2
0 1
0 1
1
Arly.7G24460.t1
Arth.4G28140.1
Bost.7867s0648.1
Caru.10005203m
Leal.36010
Brra.A01788.1
Brra.H01496.1
Brra.K00042.1
Siir.244459
Scpa.7g26090
Eusa.10025862m
Aear.18782
Arly.3G54150.t1
Arth.2G20880.1
Bost.7903s0066.1
Caru.10014129m
Leal.14657
Brra.I04678.1
Siir.229297
Scpa.3g34150
Eusa.10002185m
Capa.supercontig 34.132
Thca.1EG008066t1
Potr..013G135600.1
Potr..019G102200.1
Glma.06G295300.1
Glma.12G110400.1
Phvu.t011G118600.1
Viun.11g107700.1
Trpr.57577mRNA32444
Glma.12G203100.1
Glma.13G298600.1
Phvu.t005G105200.1
Viun.05g216500.1
Metr.2g090235.1
Trpr.57577mRNA42062
Cusa.118970.1
Cusa.147760.1
Frve.17698.1
Soly.07g054220.1.1
Migu.H01065.1
Lasa.1 v5 gn 8 132101.1
Nenu.01190-RA
Nenu.16196-RA
Aqco.e4G147300.1
Zema.00008a038281 T01
Zema.00008a006716 T01
Sobi.006G156100.1
Seit.7G176700.1
Pahal.G01361.1
Orsa.04g44670.1
Brdi.5g16450.1
Zema.00008a022427 T01
Zema.00008a016804 T01
Sobi.004G227400.2
Seit.1G248300.1
Pahal.A02700.1
Orsa.02g42585.1
Brdi.3g49810.1
Anco.016346.1
Anco.007650.1
Muac.4P26080 001
Muac.1P00070 001
Muac.8P14110 001
Zoma.61g00240.1
Sppo.11G0051700
Amtr.scaffold00009.427
Semo.104980
1

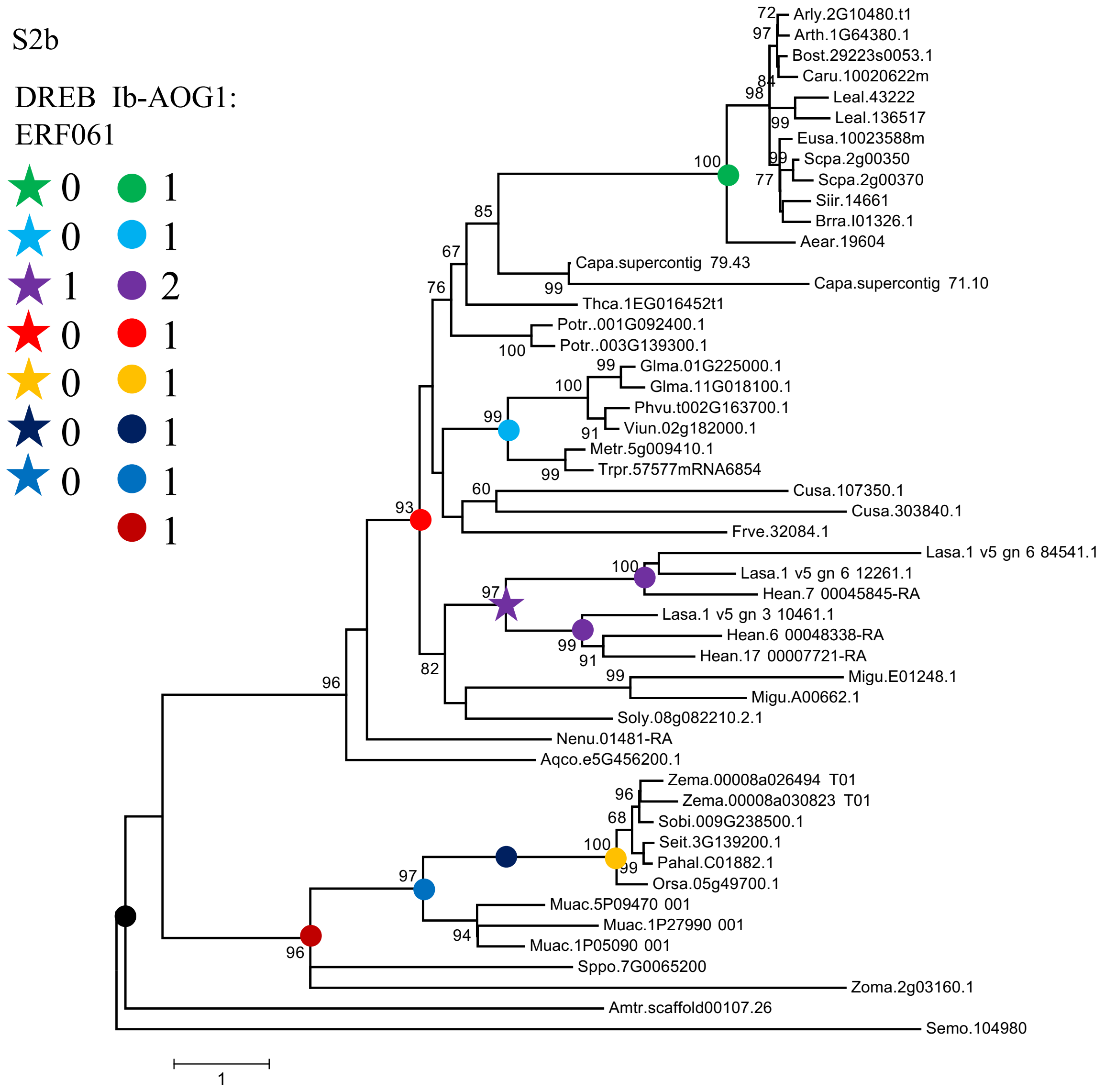
S2b
DREB Ib-AOG1:
ERF061
0 1
0 1
1 2
0 1
0 1
0 1
0 1
1
Arly.2G10480.t1
Arth.1G64380.1
Bost.29223s0053.1
Caru.10020622m
Leal.43222
Leal.136517
Eusa.10023588m
Scpa.2g00350
Scpa.2g00370
Siir.14661
Brra.I01326.1
Aear.19604
Capa.supercontig 79.43
Capa.supercontig 71.10
Thca.1EG016452t1
Potr..001G092400.1
Potr..003G139300.1
Glma.01G225000.1
Glma.11G018100.1
Phvu.t002G163700.1
Viun.02g182000.1
Metr.5g009410.1
Trpr.57577mRNA6854
Cusa.107350.1
Cusa.303840.1
Frve.32084.1
Lasa.1 v5 gn 6 84541.1
Lasa.1 v5 gn 6 12261.1
Hean.7 00045845-RA
Lasa.1 v5 gn 3 10461.1
Hean.6 00048338-RA
Hean.17 00007721-RA
Migu.E01248.1
Migu.A00662.1
Soly.08g082210.2.1
Nenu.01481-RA
Aqco.e5G456200.1
Zema.00008a026494 T01
Zema.00008a030823 T01
Sobi.009G238500.1
Seit.3G139200.1
Pahal.C01882.1
Orsa.05g49700.1
Muac.5P09470 001
Muac.1P27990 001
Muac.1P05090 001
Sppo.7G0065200
Zoma.2g03160.1
Amtr.scaffold00107.26
Semo.104980
1

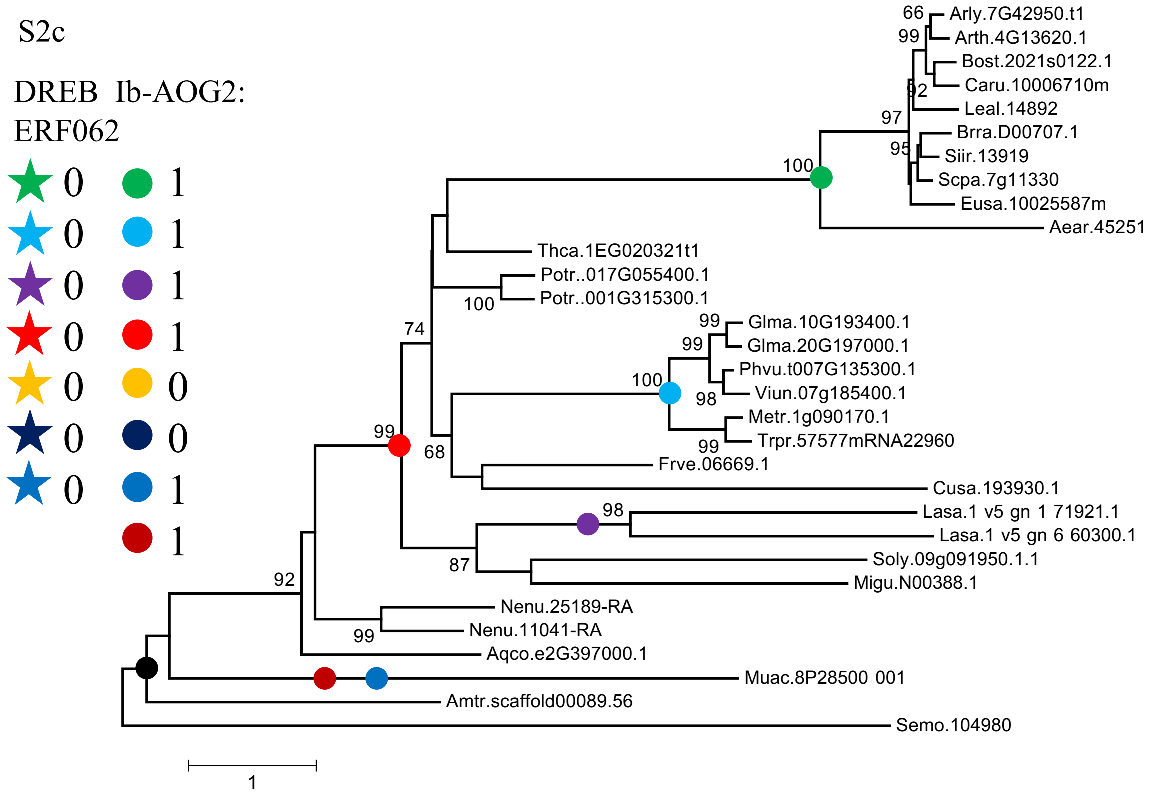
S2c
DREB Ib-AOG2:
ERF062
0 1
0 1
0 1
0 1
0 0
0 0
0 1
1
Arly.7G42950.t1
Arth.4G13620.1
Bost.2021s0122.1
Caru.10006710m
Leal.14892
Brra.D00707.1
Siir.13919
Scpa.7g11330
Eusa.10025587m
Aear.45251
Thca.1EG020321t1
Potr..017G055400.1
Potr..001G315300.1
Glma.10G193400.1
Glma.20G197000.1
Phvu.t007G135300.1
Viun.07g185400.1
Metr.1g090170.1
Trpr.57577mRNA22960
Frve.06669.1
Cusa.193930.1
Lasa.1 v5 gn 1 71921.1
Lasa.1 v5 gn 6 60300.1
Soly.09g091950.1.1
Migu.N00388.1
Nenu.25189-RA
Nenu.11041-RA
Aqco.e2G397000.1
Muac.8P28500 001
Amtr.scaffold00089.56
Semo.104980
1

S2d

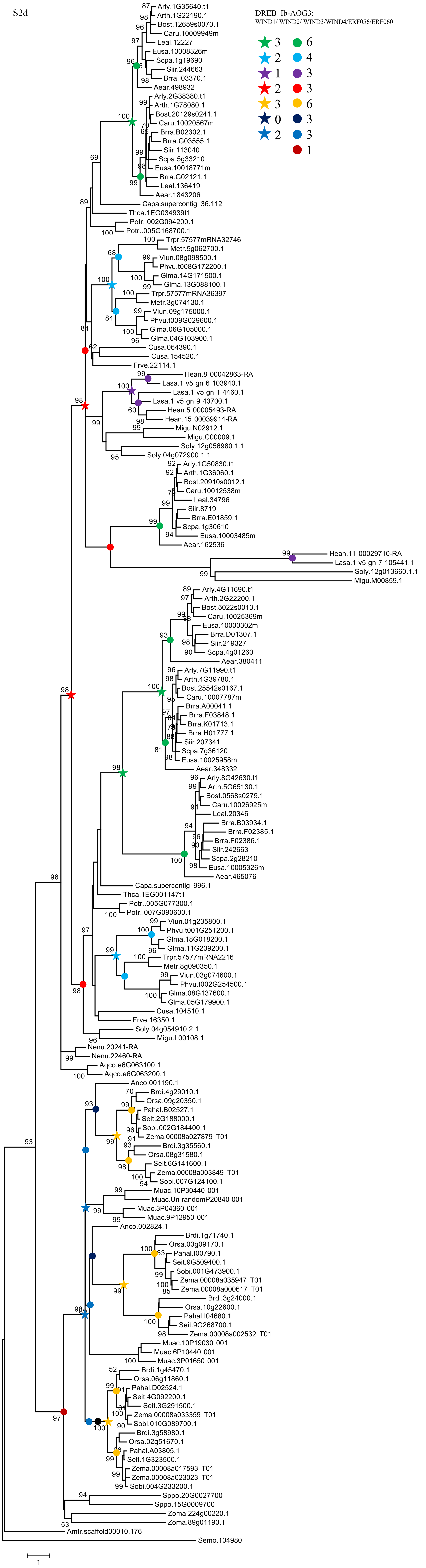

Fig. S2 Phylogenetic trees of the DREB I subfamily from representative Angiosperms. SH-aLRT supports above 50% are labeled on internal nodes. The labeling is the same as in Fig. S1.

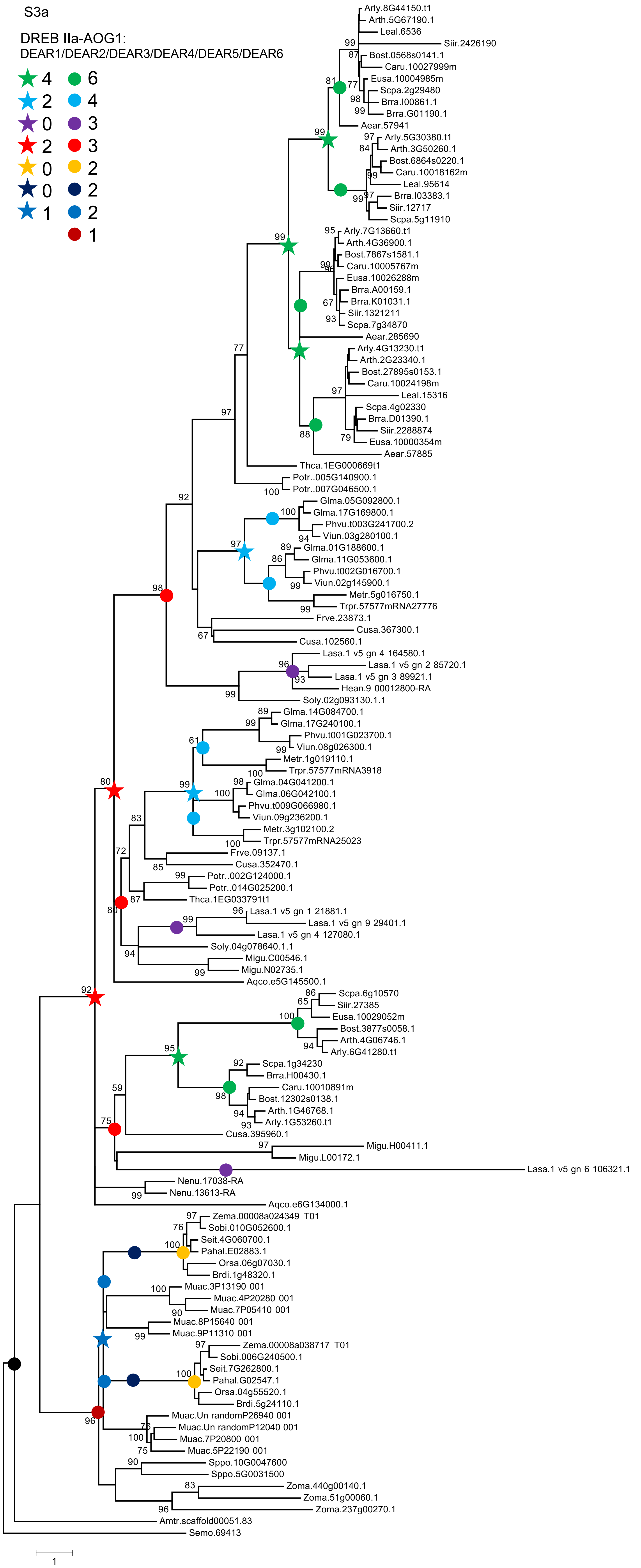
S3a
DREB IIa-AOG1:
DEAR1/DEAR2/DEAR3/DEAR4/DEAR5/DEAR6
4 6
2 4
0 3
2 3
0 2
0 2
1 2
1
Arly.8G44150.t1
Arth.5G67190.1
Leal.6536
Siir.2426190
Bost.0568s0141.1
Caru.10027999m
Eusa.10004985m
Scpa.2g29480
Brra.I00861.1
Brra.G01190.1
Aear.57941
Arly.5G30380.t1
Arth.3G50260.1
Bost.6864s0220.1
Caru.10018162m
Leal.95614
Brra.I03383.1
Siir.12717
Scpa.5g11910
Arly.7G13660.t1
Arth.4G36900.1
Bost.7867s1581.1
Caru.10005767m
Eusa.10026288m
Brra.A00159.1
Brra.K01031.1
Siir.1321211
Scpa.7g34870
Aear.285690
Arly.4G13230.t1
Arth.2G23340.1
Bost.27895s0153.1
Caru.10024198m
Leal.15316
Scpa.4g02330
Brra.D01390.1
Siir.2288874
Eusa.10000354m
Aear.57885
Thca.1EG000669t1
Potr..005G140900.1
Potr..007G046500.1
Glma.05G092800.1
Glma.17G169800.1
Phvu.t003G241700.2
Viun.03g280100.1
Glma.01G188600.1
Glma.11G053600.1
Phvu.t002G016700.1
Viun.02g145900.1
Metr.5g016750.1
Trpr.57577mRNA27776
Frve.23873.1
Cusa.367300.1
Cusa.102560.1
Lasa.1 v5 gn 4 164580.1
Lasa.1 v5 gn 2 85720.1
Lasa.1 v5 gn 3 89921.1
Hean.9 00012800-RA
Soly.02g093130.1.1
Glma.14G084700.1
Glma.17G240100.1
Phvu.t001G023700.1
Viun.08g026300.1
Metr.1g019110.1
Trpr.57577mRNA3918
Glma.04G041200.1
Glma.06G042100.1
Phvu.t009G066980.1
Viun.09g236200.1
Metr.3g102100.2
Trpr.57577mRNA25023
Frve.09137.1
Cusa.352470.1
Potr..002G124000.1
Potr..014G025200.1
Thca.1EG033791t1
Lasa.1 v5 gn 1 21881.1
Lasa.1 v5 gn 9 29401.1
Lasa.1 v5 gn 4 127080.1
Soly.04g078640.1.1
Migu.C00546.1
Migu.N02735.1
Aqco.e5G145500.1
Scpa.6g10570
Siir.27385
Eusa.10029052m
Bost.3877s0058.1
Arth.4G06746.1
Arly.6G41280.t1
Scpa.1g34230
Brra.H00430.1
Caru.10010891m
Bost.12302s0138.1
Arth.1G46768.1
Arly.1G53260.t1
Cusa.395960.1
Migu.H00411.1
Migu.L00172.1
Lasa.1 v5 gn 6 106321.1
Nenu.17038-RA
Nenu.13613-RA
Aqco.e6G134000.1
Zema.00008a024349 T01
Sobi.010G052600.1
Seit.4G060700.1
Pahal.E02883.1
Orsa.06g07030.1
Brdi.1g48320.1
Muac.3P13190 001
Muac.4P20280 001
Muac.7P05410 001
Muac.8P15640 001
Muac.9P11310 001
Zema.00008a038717 T01
Sobi.006G240500.1
Seit.7G262800.1
Pahal.G02547.1
Orsa.04g55520.1
Brdi.5g24110.1
Muac.Un randomP26940 001
Muac.Un randomP12040 001
Muac.7P20800 001
Muac.5P22190 001
Sppo.10G0047600
Sppo.5G0031500
Zoma.440g00140.1
Zoma.51g00060.1
Zoma.237g00270.1
Amtr.scaffold00051.83
Semo.69413
1

S3b

DREB IIb-AOG1 :
ERF012/ERF013/ERF014

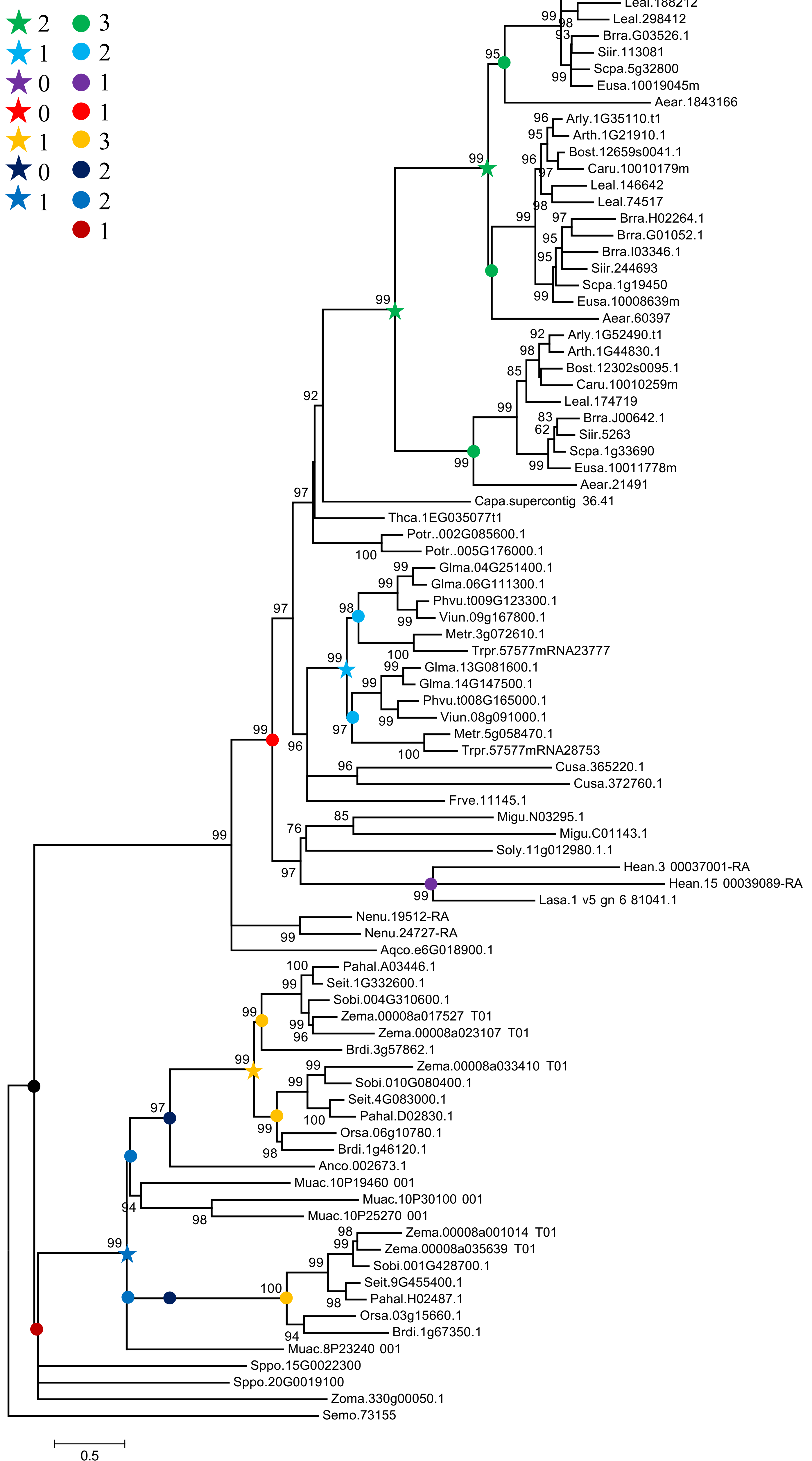

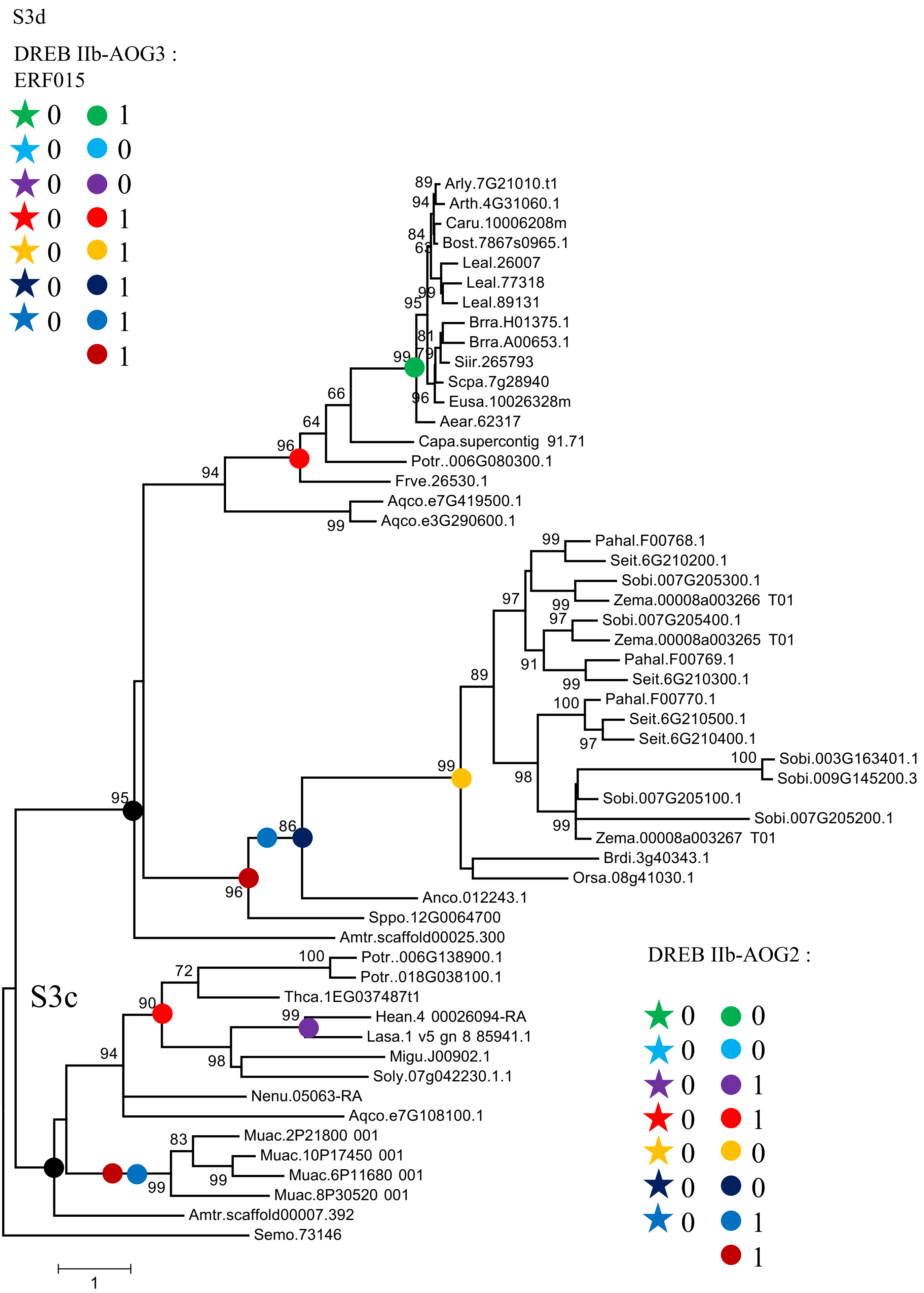
S3d
DREB IIb-AOG3 :
ERF015
0 1
0 0
0 0
0 1
0 1
0 1
0 1
1
Arly.7G21010.t1
Arth.4G31060.1
Caru.10006208m
Bost.7867s0965.1
Leal.26007
Leal.77318
Leal.89131
Brra.H01375.1
Brra.A00653.1
Siir.265793
Scpa.7g28940
Eusa.10026328m
Aear.62317
Capa.supercontig 91.71
Potr..006G080300.1
Frve.26530.1
Aqco.e7G419500.1
Aqco.e3G290600.1
Pahal.F00768.1
Seit.6G210200.1
Sobi.007G205300.1
Zema.00008a003266 T01
Sobi.007G205400.1
Zema.00008a003265 T01
Pahal.F00769.1
Seit.6G210300.1
Pahal.F00770.1
Seit.6G210500.1
Seit.6G210400.1
Sobi.003G163401.1
Sobi.009G145200.3
Sobi.007G205100.1
Sobi.007G205200.1
Zema.00008a003267 T01
Brdi.3g40343.1
Orsa.08g41030.1
Anco.012243.1
Sppo.12G0064700
Amtr.scaffold00025.300
Potr..006G138900.1
Potr..018G038100.1
Thca.1EG037487t1
Hean.4 00026094-RA
Lasa.1 v5 gn 8 85941.1
Migu.J00902.1
Soly.07g042230.1.1
Nenu.05063-RA
Aqco.e7G108100.1
Muac.2P21800 001
Muac.10P17450 001
Muac.6P11680 001
Muac.8P30520 001
Amtr.scaffold00007.392
Semo.73146
S3c
1
DREB IIb-AOG2 :
0 0
0 0
0 1
0 1
0 0
0 0
0 1
1

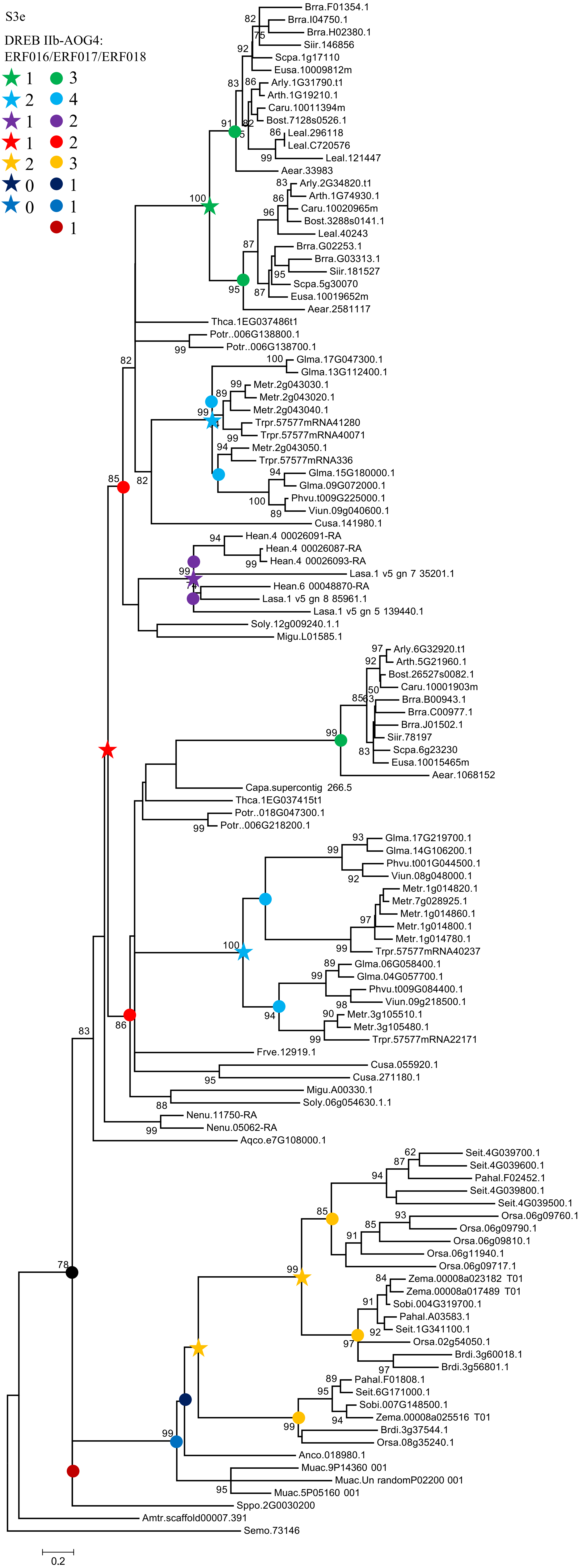
S3e
DREB IIb-AOG4:
ERF016/ERF017/ERF018
1 3
2 4
1 2
1 2
2 3
0 1
0 1
1
Brra.F01354.1
Brra.I04750.1
Brra.H02380.1
Siir.146856
Scpa.1g17110
Eusa.10009812m
Arly.1G31790.t1
Arth.1G19210.1
Caru.10011394m
Bost.7128s0526.1
Leal.296118
Leal.C720576
Leal.121447
Aear.33983
Arly.2G34820.t1
Arth.1G74930.1
Caru.10020965m
Bost.3288s0141.1
Leal.40243
Brra.G02253.1
Brra.G03313.1
Siir.181527
Scpa.5g30070
Eusa.10019652m
Aear.2581117
Thca.1EG037486t1
Potr..006G138800.1
Potr..006G138700.1
Glma.17G047300.1
Glma.13G112400.1
Metr.2g043030.1
Metr.2g043020.1
Metr.2g043040.1
Trpr.57577mRNA41280
Trpr.57577mRNA40071
Metr.2g043050.1
Trpr.57577mRNA336
Glma.15G180000.1
Glma.09G072000.1
Phvu.t009G225000.1
Viun.09g040600.1
Cusa.141980.1
Hean.4 00026091-RA
Hean.4 00026087-RA
Hean.4 00026093-RA
Lasa.1 v5 gn 7 35201.1
Hean.6 00048870-RA
Lasa.1 v5 gn 8 85961.1
Lasa.1 v5 gn 5 139440.1
Soly.12g009240.1.1
Migu.L01585.1
Arly.6G32920.t1
Arth.5G21960.1
Bost.26527s0082.1
Caru.10001903m
Brra.B00943.1
Brra.C00977.1
Brra.J01502.1
Siir.78197
Scpa.6g23230
Eusa.10015465m
Aear.1068152
Capa.supercontig 266.5
Thca.1EG037415t1
Potr..018G047300.1
Potr..006G218200.1
Glma.17G219700.1
Glma.14G106200.1
Phvu.t001G044500.1
Viun.08g048000.1
Metr.1g014820.1
Metr.7g028925.1
Metr.1g014860.1
Metr.1g014800.1
Metr.1g014780.1
Trpr.57577mRNA40237
Glma.06G058400.1
Glma.04G057700.1
Phvu.t009G084400.1
Viun.09g218500.1
Metr.3g105510.1
Metr.3g105480.1
Trpr.57577mRNA22171
Frve.12919.1
Cusa.055920.1
Cusa.271180.1
Migu.A00330.1
Soly.06g054630.1.1
Nenu.11750-RA
Nenu.05062-RA
Aqco.e7G108000.1
Seit.4G039700.1
Seit.4G039600.1
Pahal.F02452.1
Seit.4G039800.1
Seit.4G039500.1
Orsa.06g09760.1
Orsa.06g09790.1
Orsa.06g09810.1
Orsa.06g11940.1
Orsa.06g09717.1
Zema.00008a023182 T01
Zema.00008a017489 T01
Sobi.004G319700.1
Pahal.A03583.1
Seit.1G341100.1
Orsa.02g54050.1
Brdi.3g60018.1
Brdi.3g56801.1
Pahal.F01808.1
Seit.6G171000.1
Sobi.007G148500.1
Zema.00008a025516 T01
Brdi.3g37544.1
Orsa.08g35240.1
Anco.018980.1
Muac.9P14360 001
Muac.Un randomP02200 001
Muac.5P05160 001
Sppo.2G0030200
Amtr.scaffold00007.391
Semo.73146
0.2

S3f

DREB IIc-AOG1 :
ER019/ER020

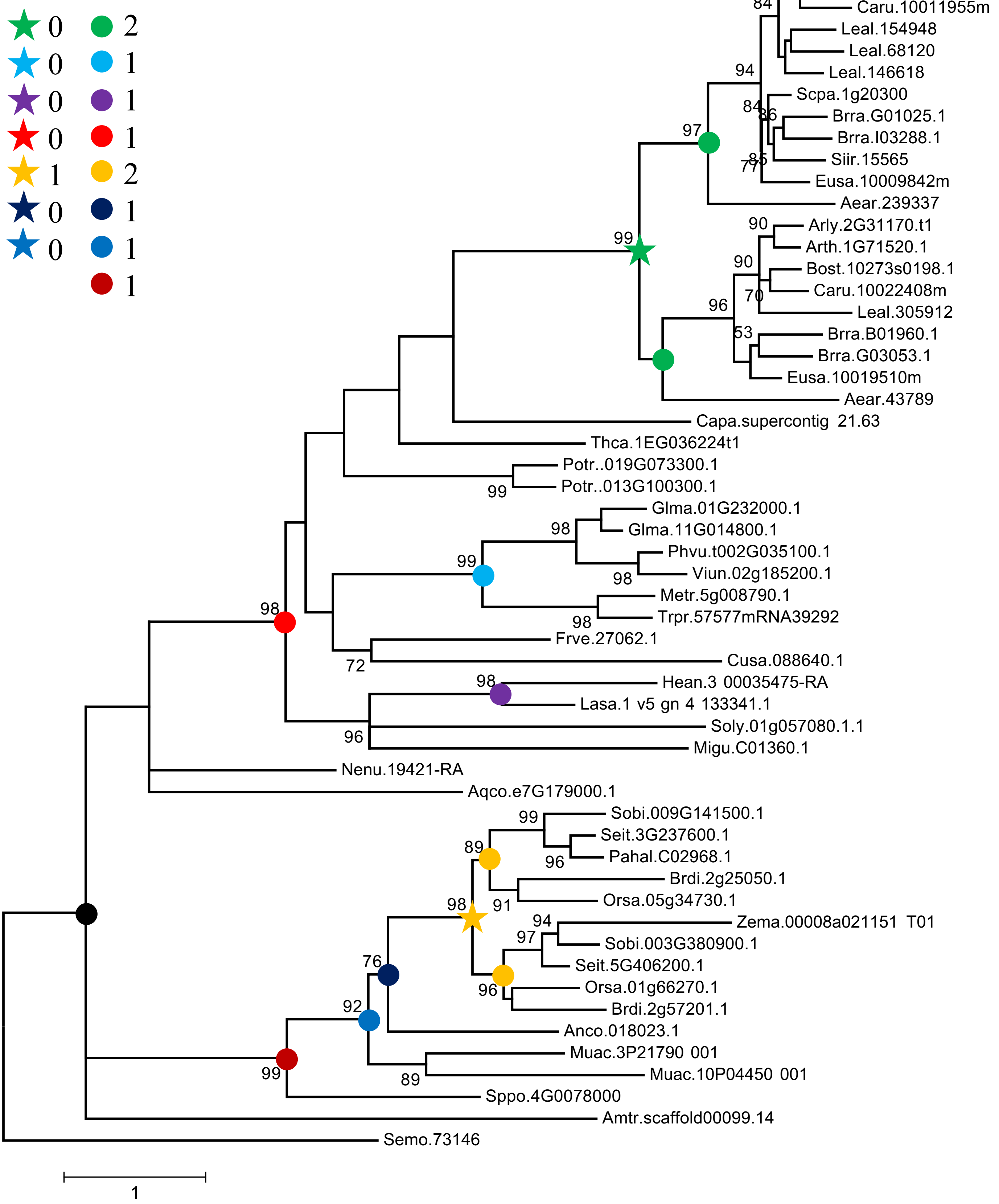

Fig. S3 Phylogenetic trees of the DREB II subfamily from representative Angiosperms. SH-aLRT supports above 50% are labeled on internal nodes. The labeling is the same as in Fig. S1.

S4a

DREB IIIa-AOG1:
ERF023

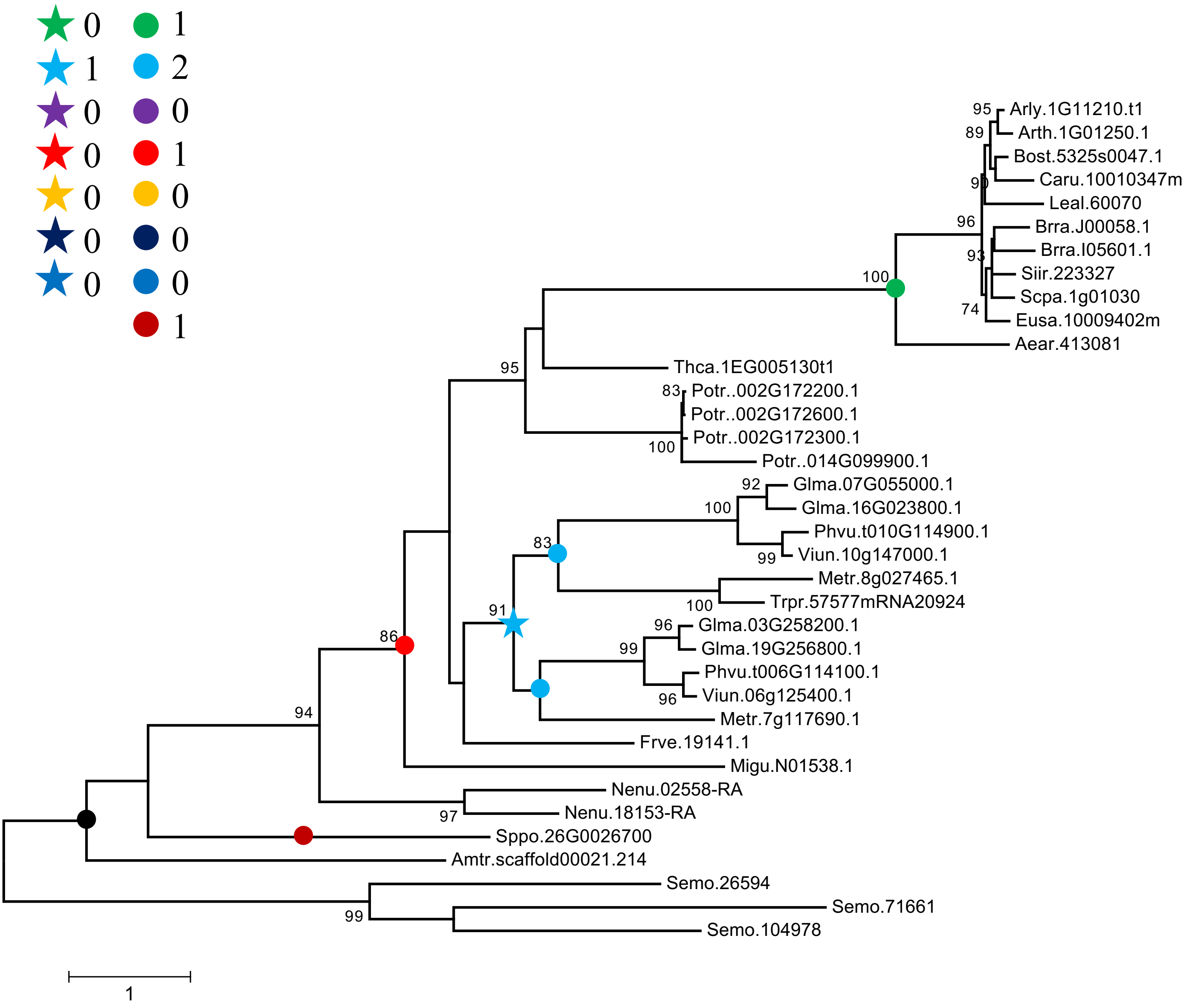

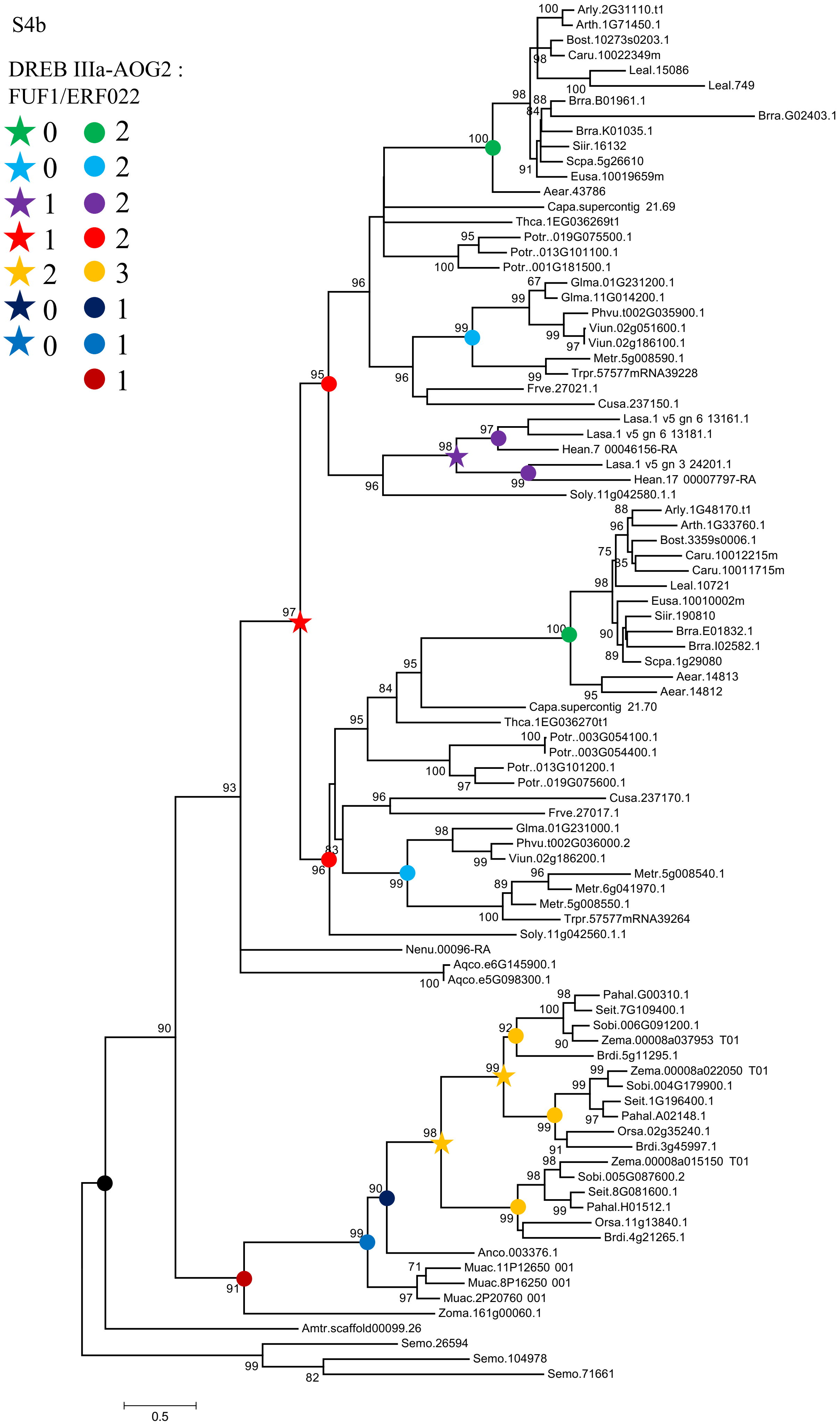

S4b
DREB IIIa-AOG2 :
FUF1/ERF022
0 2
0 2
1 2
1 2
2 3
0 1
0 1
1
Arly.2G31110.t1
Arth.1G71450.1
Bost.10273s0203.1
Caru.10022349m
Leal.15086
Leal.749
Brra.B01961.1
Brra.G02403.1
Brra.K01035.1
Siir.16132
Scpa.5g26610
Eusa.10019659m
Aear.43786
Capa.supercontig 21.69
Thca.1EG036269t1
Potr..019G075500.1
Potr..013G101100.1
Potr..001G181500.1
Glma.01G231200.1
Glma.11G014200.1
Phvu.t002G035900.1
Viun.02g051600.1
Viun.02g186100.1
Metr.5g008590.1
Trpr.57577mRNA39228
Frve.27021.1
Cusa.237150.1
Lasa.1 v5 gn 6 13161.1
Lasa.1 v5 gn 6 13181.1
Hean.7 00046156-RA
Lasa.1 v5 gn 3 24201.1
Hean.17 00007797-RA
Soly.11g042580.1.1
Arly.1G48170.t1
Arth.1G33760.1
Bost.3359s0006.1
Caru.10012215m
Caru.10011715m
Leal.10721
Eusa.10010002m
Siir.190810
Brra.E01832.1
Brra.I02582.1
Scpa.1g29080
Aear.14813
Aear.14812
Capa.supercontig 21.70
Thca.1EG036270t1
Potr..003G054100.1
Potr..003G054400.1
Potr..013G101200.1
Potr..019G075600.1
Cusa.237170.1
Frve.27017.1
Glma.01G231000.1
Phvu.t002G036000.2
Viun.02g186200.1
Metr.5g008540.1
Metr.6g041970.1
Metr.5g008550.1
Trpr.57577mRNA39264
Soly.11g042560.1.1
Nenu.00096-RA
Aqco.e6G145900.1
Aqco.e5G098300.1
Pahal.G00310.1
Seit.7G109400.1
Sobi.006G091200.1
Zema.00008a037953 T01
Brdi.5g11295.1
Zema.00008a022050 T01
Sobi.004G179900.1
Seit.1G196400.1
Pahal.A02148.1
Orsa.02g35240.1
Brdi.3g45997.1
Zema.00008a015150 T01
Sobi.005G087600.2
Seit.8G081600.1
Pahal.H01512.1
Orsa.11g13840.1
Brdi.4g21265.1
Anco.003376.1
Muac.11P12650 001
Muac.8P16250 001
Muac.2P20760 001
Zoma.161g00060.1
Amtr.scaffold00099.26
Semo.26594
Semo.104978
Semo.71661
0.5

S4c

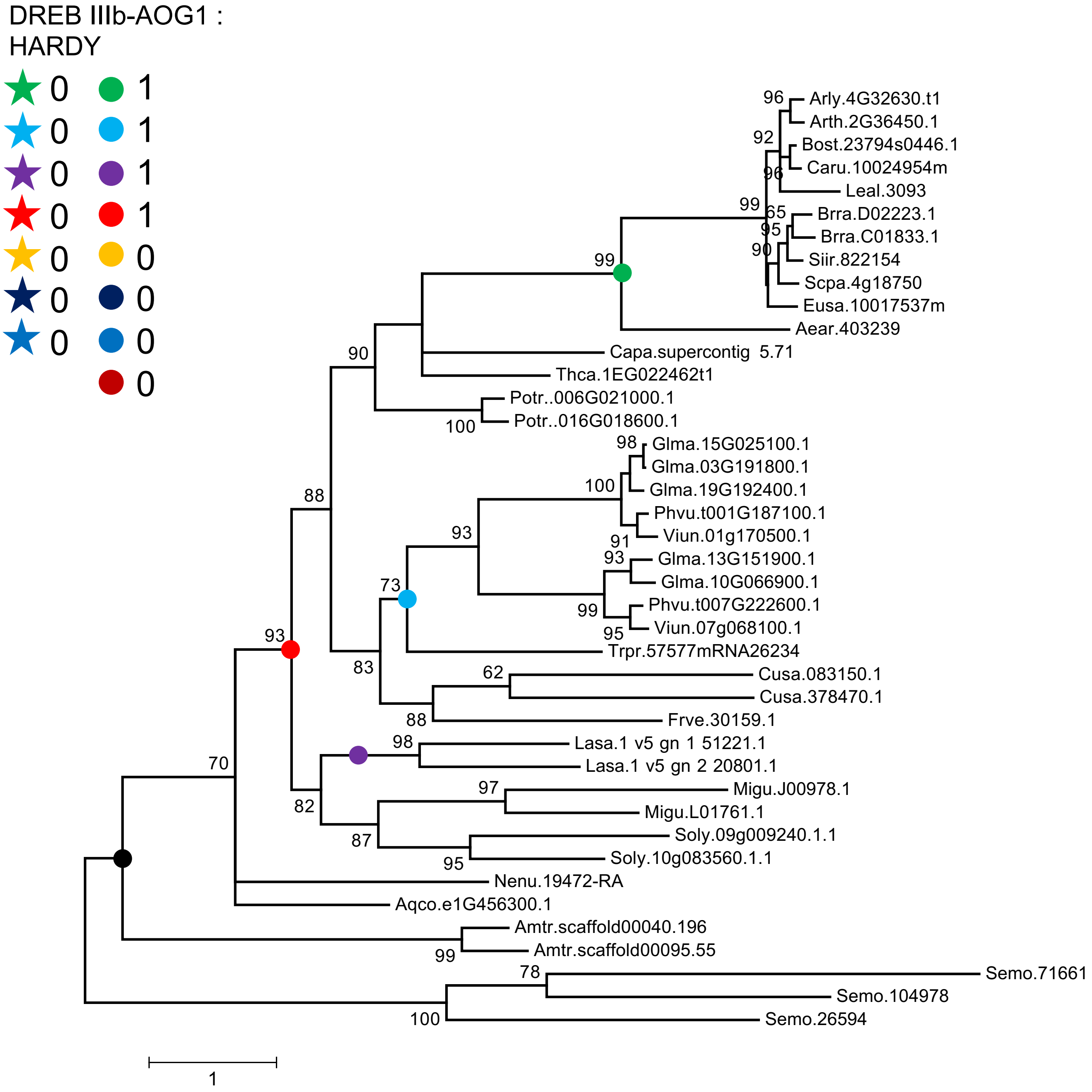
DREB IIIb-AOG1 :
HARDY
0 1
0 1
0 1
0 1
0 0
0 0
0 0
0
Arly.4G32630.t1
Arth.2G36450.1
Bost.23794s0446.1
Caru.10024954m
Leal.3093
Brra.D02223.1
Brra.C01833.1
Siir.822154
Scpa.4g18750
Eusa.10017537m
Aear.403239
Capa.supercontig 5.71
Thca.1EG022462t1
Potr..006G021000.1
Potr..016G018600.1
Glma.15G025100.1
Glma.03G191800.1
Glma.19G192400.1
Phvu.t001G187100.1
Viun.01g170500.1
Glma.13G151900.1
Glma.10G066900.1
Phvu.t007G222600.1
Viun.07g068100.1
Trpr.57577mRNA26234
Cusa.083150.1
Cusa.378470.1
Frve.30159.1
Lasa.1 v5 gn 1 51221.1
Lasa.1 v5 gn 2 20801.1
Migu.J00978.1
Migu.L01761.1
Soly.09g009240.1.1
Soly.10g083560.1.1
Nenu.19472-RA
Aqco.e1G456300.1
Amtr.scaffold00040.196
Amtr.scaffold00095.55
Semo.71661
Semo.104978
Semo.26594
1

S4d

DREB IIIb-AOG2 :
ERF025/ERF027

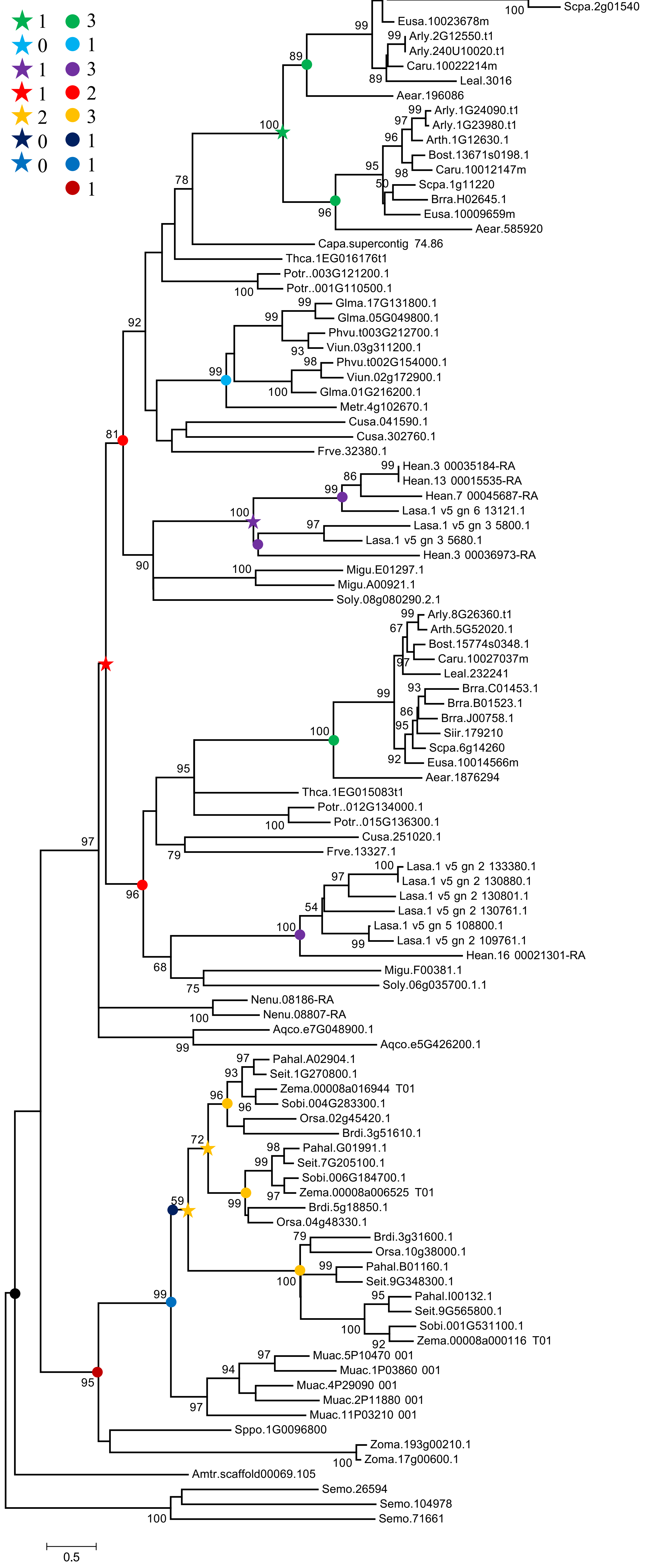

S4e

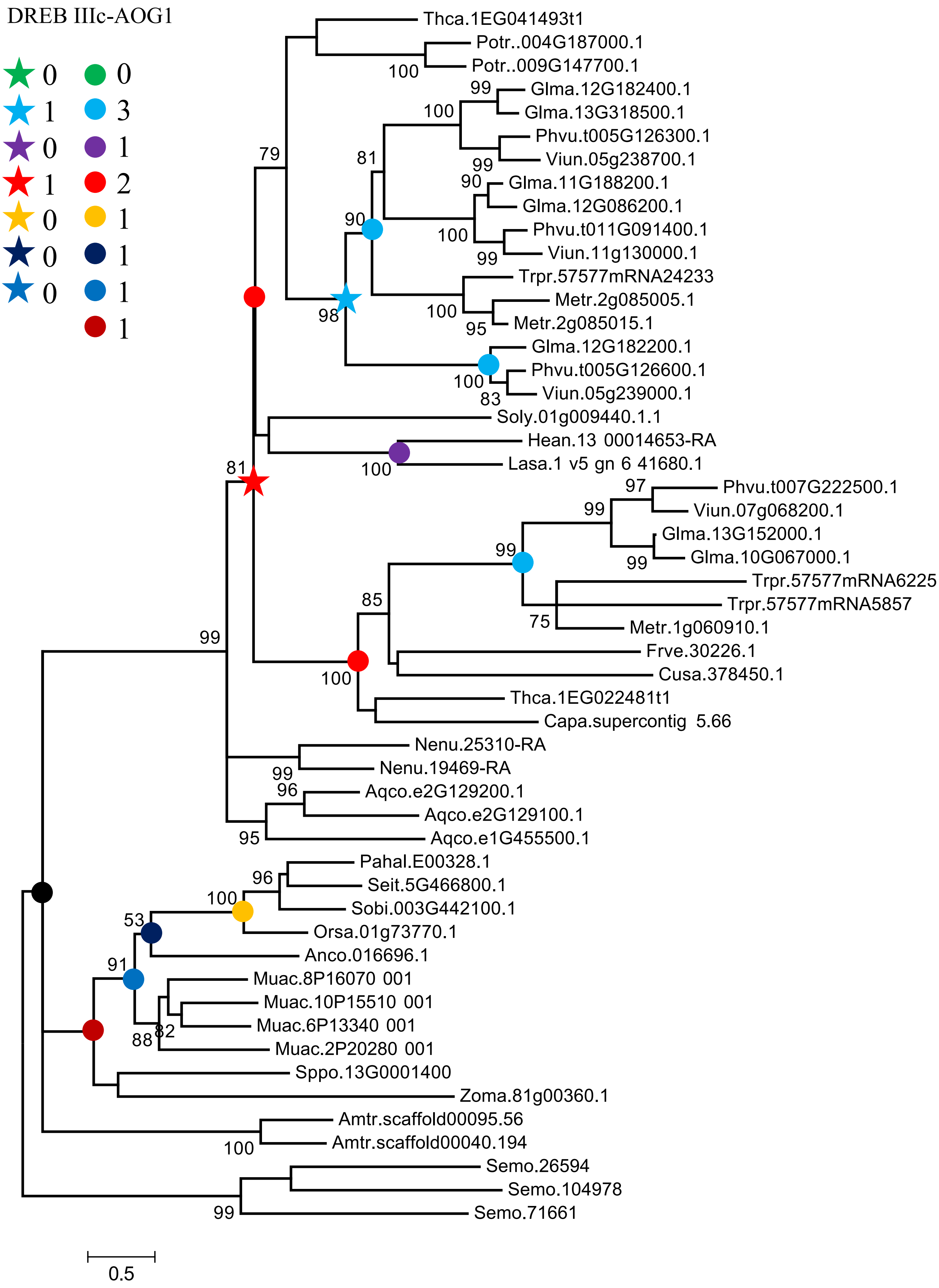
DREB IIIc-AOG1
0
1
0
1
0
0
0
0
3
1
2
1
1
1
1
Thca.1EG041493t1
Potr..004G187000.1
Potr..009G147700.1
Glma.12G182400.1
Glma.13G318500.1
Phvu.t005G126300.1
Viun.05g238700.1
Glma.11G188200.1
Glma.12G086200.1
Phvu.t011G091400.1
Viun.11g130000.1
Trpr.57577mRNA24233
Metr.2g085005.1
Metr.2g085015.1
Glma.12G182200.1
Phvu.t005G126600.1
Viun.05g239000.1
Soly.01g009440.1.1
Hean.13 00014653-RA
Lasa.1 v5 gn 6 41680.1
Phvu.t007G222500.1
Viun.07g068200.1
Glma.13G152000.1
Glma.10G067000.1
Trpr.57577mRNA6225
Trpr.57577mRNA5857
Metr.1g060910.1
Frve.30226.1
Cusa.378450.1
Thca.1EG022481t1
Capa.supercontig 5.66
Nenu.25310-RA
Nenu.19469-RA
Aqco.e2G129200.1
Aqco.e2G129100.1
Aqco.e1G455500.1
Pahal.E00328.1
Seit.5G466800.1
Sobi.003G442100.1
Orsa.01g73770.1
Anco.016696.1
Muac.8P16070 001
Muac.10P15510 001
Muac.6P13340 001
Muac.2P20280 001
Sppo.13G0001400
Zoma.81g00360.1
Amtr.scaffold00095.56
Amtr.scaffold00040.194
Semo.26594
Semo.104978
Semo.71661
0.5

S4f

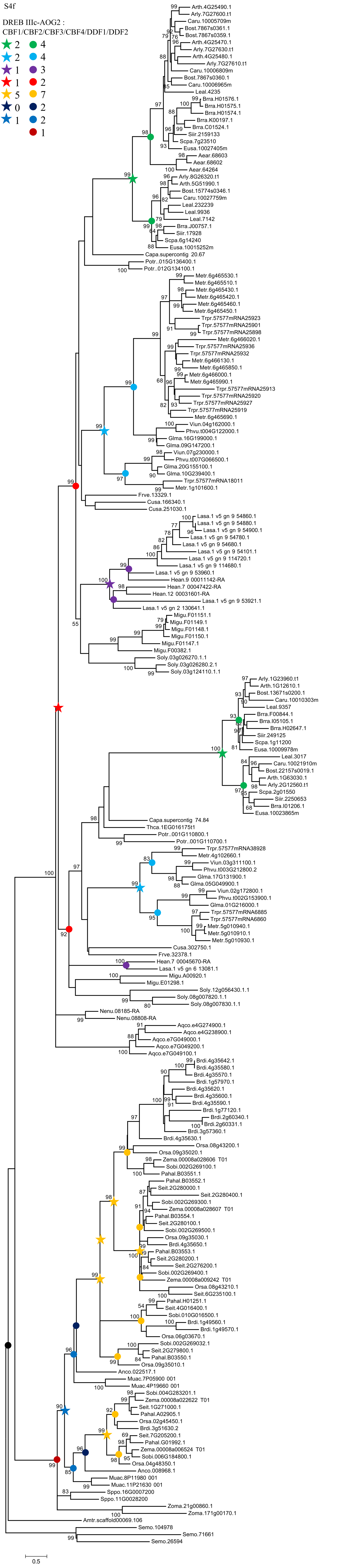
DREB IIIc-AOG2 :
CBF1/CBF2/CBF3/CBF4/DDF1/DDF2
Arth.4G25490.1
Arly.7G27600.t1
Caru.10005709m
Bost.7867s0361.1
Bost.7867s0359.1
Arth.4G25470.1
Arly.7G27630.t1
Arth.4G25480.1
Arly.7G27610.t1
Caru.10006809m
Bost.7867s0360.1
Caru.10006965m
Leal.4235
Brra.H01576.1
Brra.H01575.1
Brra.H01574.1
Brra.K00197.1
Brra.C01524.1
Siir.2159133
Scpa.7g23510
Eusa.10027405m
Aear.68603
Aear.68602
Aear.64264
Arly.8G26320.t1
Arth.5G51990.1
Bost.15774s0346.1
Caru.10027759m
Leal.232239
Leal.9936
Leal.7142
Brra.J00757.1
Siir.17928
Scpa.6g14240
Eusa.10015252m
Capa.supercontig 20.67
Potr..015G136400.1
Potr..012G134100.1
Metr.6g465530.1
Metr.6g465510.1
Metr.6g465430.1
Metr.6g465420.1
Metr.6g465460.1
Metr.6g465450.1
Trpr.57577mRNA25923
Trpr.57577mRNA25901
Trpr.57577mRNA25898
Metr.6g466020.1
Trpr.57577mRNA25936
Trpr.57577mRNA25932
Metr.6g466130.1
Metr.6g465850.1
Metr.6g466000.1
Metr.6g465990.1
Trpr.57577mRNA25913
Trpr.57577mRNA25920
Trpr.57577mRNA25927
Trpr.57577mRNA25919
Metr.6g465690.1
Viun.04g162000.1
Phvu.t004G122000.1
Glma.16G199000.1
Glma.09G147200.1
Viun.07g230000.1
Phvu.t007G066500.1
Glma.20G155100.1
Glma.10G239400.1
Trpr.57577mRNA18011
Metr.1g101600.1
Frve.13329.1
Cusa.166340.1
Cusa.251030.1
Lasa.1 v5 gn 9 54860.1
Lasa.1 v5 gn 9 54880.1
Lasa.1 v5 gn 9 54900.1
Lasa.1 v5 gn 9 54780.1
Lasa.1 v5 gn 9 54680.1
Lasa.1 v5 gn 9 54101.1
Lasa.1 v5 gn 9 114720.1
Lasa.1 v5 gn 9 114680.1
Lasa.1 v5 gn 9 53960.1
Hean.9 00011142-RA
Hean.7 00047422-RA
Hean.12 00031601-RA
Lasa.1 v5 gn 9 53921.1
Lasa.1 v5 gn 2 130641.1
Migu.F01151.1
Migu.F01149.1
Migu.F01148.1
Migu.F01150.1
Migu.F01147.1
Migu.F00382.1
Soly.03g026270.1.1
Soly.03g026280.2.1
Soly.03g124110.1.1
Arly.1G23960.t1
Arth.1G12610.1
Bost.13671s0200.1
Caru.10010303m
Leal.9357
Brra.F00844.1
Brra.I05105.1
Brra.H02647.1
Siir.249125
Scpa.1g11200
Eusa.10009978m
Leal.3017
Caru.10021910m
Bost.22157s0019.1
Arth.1G63030.1
Arly.2G12560.t1
Scpa.2g01550
Siir.2250653
Brra.I01206.1
Eusa.10023865m
Capa.supercontig 74.84
Thca.1EG016175t1
Potr..001G110800.1
Potr..001G110700.1
Trpr.57577mRNA38928
Metr.4g102660.1
Viun.03g311100.1
Phvu.t003G212800.2
Glma.17G131900.1
Glma.05G049900.1
Viun.02g172800.1
Phvu.t002G153900.1
Glma.01G216000.1
Trpr.57577mRNA6885
Trpr.57577mRNA6880
Metr.5g010940.1
Metr.5g010910.1
Metr.5g010930.1
Cusa.302750.1
Frve.32378.1
Hean.7 00045670-RA
Lasa.1 v5 gn 6 13081.1
Migu.A00920.1
Migu.E01298.1
Soly.12g056430.1.1
Soly.08g007820.1.1
Soly.08g007830.1.1
Nenu.08185-RA
Nenu.08808-RA
Aqco.e4G274900.1
Aqco.e4G238900.1
Aqco.e7G049000.1
Aqco.e7G049200.1
Aqco.e7G049100.1
Brdi.4g35642.1
Brdi.4g35580.1
Brdi.4g35570.1
Brdi.1g57970.1
Brdi.4g35620.1
Brdi.4g35600.1
Brdi.4g35590.1
Brdi.1g77120.1
Brdi.2g60340.1
Brdi.2g60331.1
Brdi.3g57360.1
Brdi.4g35630.1
Orsa.08g43200.1
Orsa.09g35020.1
Zema.00008a028606 T01
Sobi.002G269100.1
Pahal.B03551.1
Pahal.B03552.1
Seit.2G280000.1
Seit.2G280400.1
Sobi.002G269300.1
Zema.00008a028607 T01
Pahal.B03554.1
Seit.2G280100.1
Sobi.002G269500.1
Orsa.09g35030.1
Brdi.4g35650.1
Pahal.B03553.1
Seit.2G280200.1
Seit.2G276200.1
Sobi.002G269400.1
Zema.00008a009242 T01
Orsa.08g43210.1
Seit.6G235100.1
Pahal.H01251.1
Seit.4G016400.1
Sobi.010G016500.1
Brdi.1g49560.1
Brdi.1g49570.1
Orsa.06g03670.1
Sobi.002G269032.1
Seit.2G279800.1
Pahal.B03550.1
Orsa.09g35010.1
Anco.022517.1
Muac.7P05900 001
Muac.4P19660 001
Sobi.004G283201.1
Zema.00008a022622 T01
Seit.1G271000.1
Pahal.A02905.1
Orsa.02g45450.1
Brdi.3g51630.2
Seit.7G205200.1
Pahal.G01992.1
Zema.00008a006524 T01
Sobi.006G184800.1
Orsa.04g48350.1
Anco.008968.1
Muac.8P11980 001
Muac.11P21630 001
Sppo.16G0007200
Sppo.11G0028200
Zoma.21g00860.1
Zoma.171g00170.1
Amtr.scaffold00069.106
Semo.104978
Semo.71661
Semo.26594
0.5

S4g

DREB IIId-AOG1 :
ERF034/ERF035/ERF039/ERF038

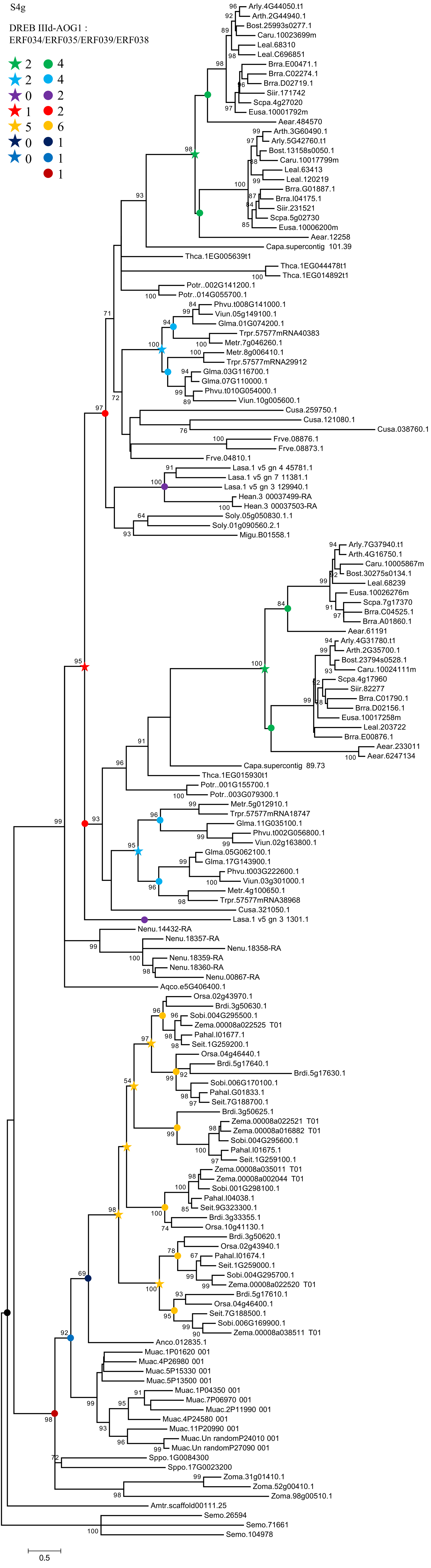

S4h

DREB IIIe-AOG1:
ESE2/ERF043

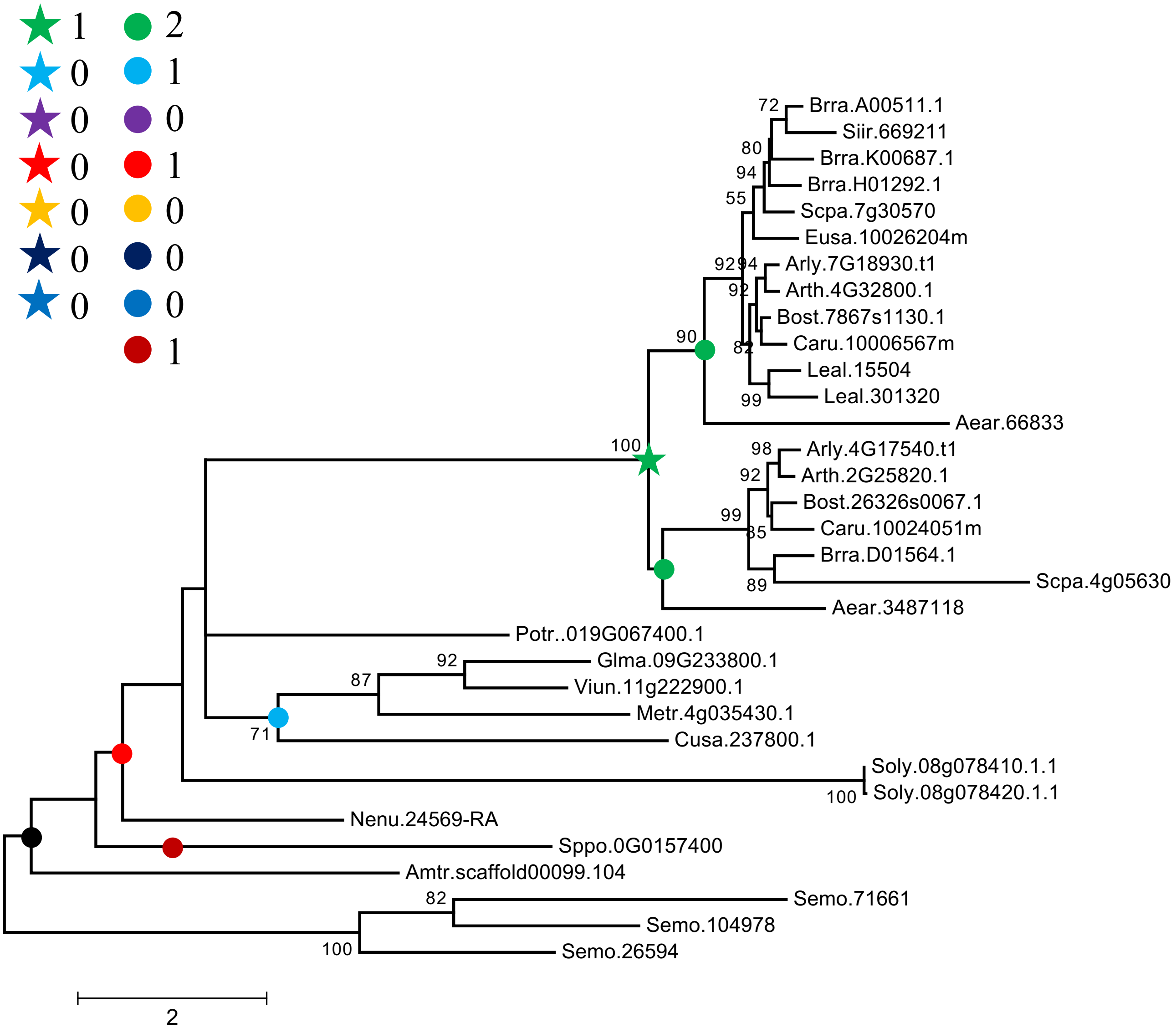

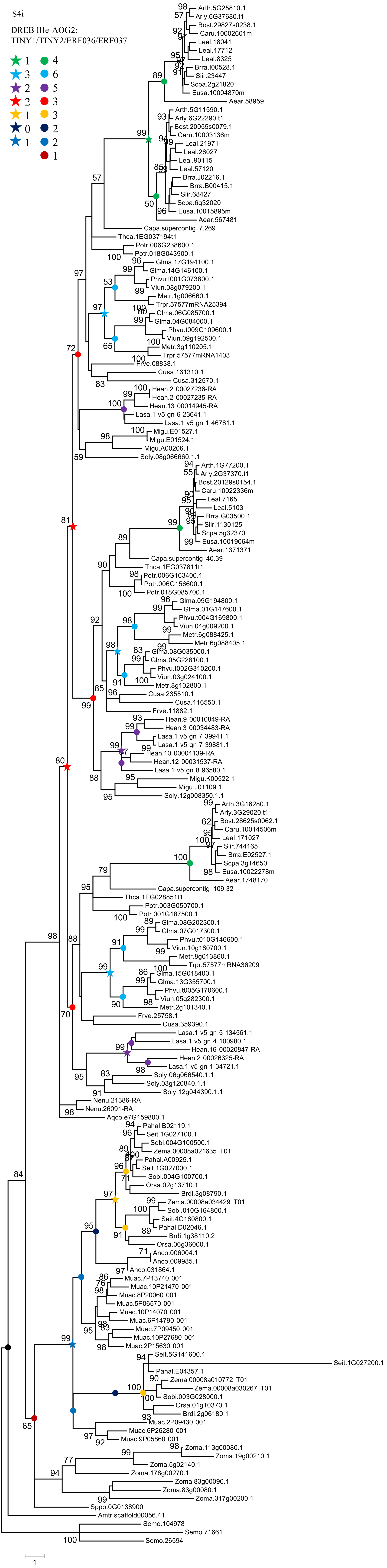

Fig. S4 Phylogenetic trees of DREB III subfamily from representative Angiosperms. SH-aLRT supports above 50% are labeled on internal nodes. The labeling is the same as in Fig. S1.

S5a

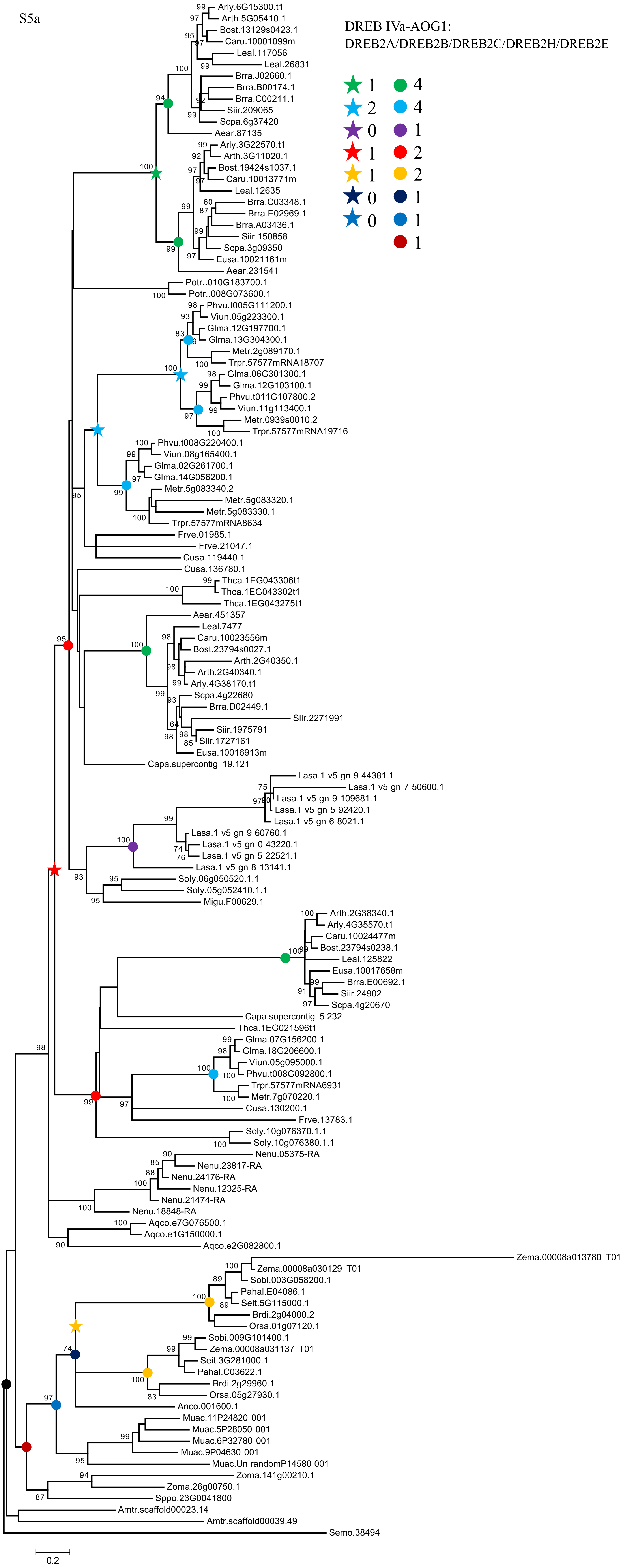
DREB IVa-AOG1:
DREB2A/DREB2B/DREB2C/DREB2H/DREB2E
1 4
2 4
0 1
1 2
1 2
0 1
0 1
1
Arly.6G15300.t1
Arth.5G05410.1
Bost.13129s0423.1
Caru.10001099m
Leal.117056
Leal.26831
Brra.J02660.1
Brra.B00174.1
Brra.C00211.1
Siir.209065
Scpa.6g37420
Aear.87135
Arly.3G22570.t1
Arth.3G11020.1
Bost.19424s1037.1
Caru.10013771m
Leal.12635
Brra.C03348.1
Brra.E02969.1
Brra.A03436.1
Siir.150858
Scpa.3g09350
Eusa.10021161m
Aear.231541
Potr..010G183700.1
Potr..008G073600.1
Phvu.t005G111200.1
Viun.05g223300.1
Glma.12G197700.1
Glma.13G304300.1
Metr.2g089170.1
Trpr.57577mRNA18707
Glma.06G301300.1
Glma.12G103100.1
Phvu.t011G107800.2
Viun.11g113400.1
Metr.0939s0010.2
Trpr.57577mRNA19716
Phvu.t008G220400.1
Viun.08g165400.1
Glma.02G261700.1
Glma.14G056200.1
Metr.5g083340.2
Metr.5g083320.1
Metr.5g083330.1
Trpr.57577mRNA8634
Frve.01985.1
Frve.21047.1
Cusa.119440.1
Cusa.136780.1
Thca.1EG043306t1
Thca.1EG043302t1
Thca.1EG043275t1
Aear.451357
Leal.7477
Caru.10023556m
Bost.23794s0027.1
Arth.2G40350.1
Arth.2G40340.1
Arly.4G38170.t1
Scpa.4g22680
Brra.D02449.1
Siir.2271991
Siir.1975791
Siir.1727161
Eusa.10016913m
Capa.supercontig 19.121
Lasa.1 v5 gn 9 44381.1
Lasa.1 v5 gn 7 50600.1
Lasa.1 v5 gn 9 109681.1
Lasa.1 v5 gn 5 92420.1
Lasa.1 v5 gn 6 8021.1
Lasa.1 v5 gn 9 60760.1
Lasa.1 v5 gn 0 43220.1
Lasa.1 v5 gn 5 22521.1
Lasa.1 v5 gn 8 13141.1
Soly.06g050520.1.1
Soly.05g052410.1.1
Migu.F00629.1
Arth.2G38340.1
Arly.4G35570.t1
Caru.10024477m
Bost.23794s0238.1
Leal.125822
Eusa.10017658m
Brra.E00692.1
Siir.24902
Scpa.4g20670
Capa.supercontig 5.232
Thca.1EG021596t1
Glma.07G156200.1
Glma.18G206600.1
Viun.05g095000.1
Phvu.t008G092800.1
Trpr.57577mRNA6931
Metr.7g070220.1
Cusa.130200.1
Frve.13783.1
Soly.10g076370.1.1
Soly.10g076380.1.1
Nenu.05375-RA
Nenu.23817-RA
Nenu.24176-RA
Nenu.12325-RA
Nenu.21474-RA
Nenu.18848-RA
Aqco.e7G076500.1
Aqco.e1G150000.1
Aqco.e2G082800.1
Zema.00008a013780 T01
Zema.00008a030129 T01
Sobi.003G058200.1
Pahal.E04086.1
Seit.5G115000.1
Brdi.2g04000.2
Orsa.01g07120.1
Sobi.009G101400.1
Zema.00008a031137 T01
Seit.3G281000.1
Pahal.C03622.1
Brdi.2g29960.1
Orsa.05g27930.1
Anco.001600.1
Muac.11P24820 001
Muac.5P28050 001
Muac.6P32780 001
Muac.9P04630 001
Muac.Un randomP14580 001
Zoma.141g00210.1
Zoma.26g00750.1
Sppo.23G0041800
Amtr.scaffold00023.14
Amtr.scaffold00039.49
Semo.38494
0.2

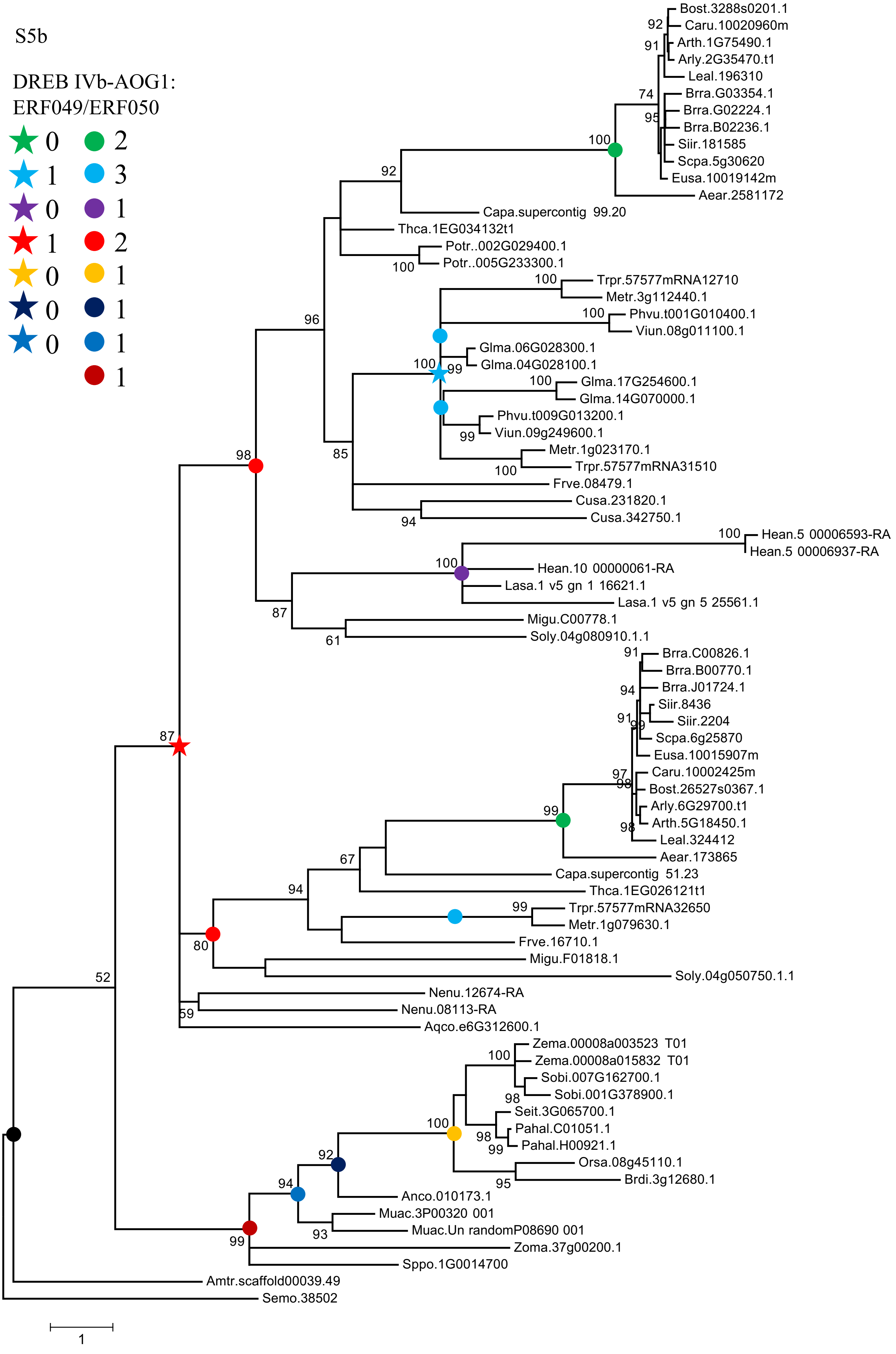
S5b
DREB IVb-AOG1:
ERF049/ERF050
0 2
1 3
0 1
1 2
0 1
0 1
0 1
1
Bost.3288s0201.1
Caru.10020960m
Arth.1G75490.1
Arly.2G35470.t1
Leal.196310
Brra.G03354.1
Brra.G02224.1
Brra.B02236.1
Siir.181585
Scpa.5g30620
Eusa.10019142m
Aear.2581172
Capa.supercontig 99.20
Thca.1EG034132t1
Potr..002G029400.1
Potr..005G233300.1
Trpr.57577mRNA12710
Metr.3g112440.1
Phvu.t001G010400.1
Viun.08g011100.1
Glma.06G028300.1
Glma.04G028100.1
Glma.17G254600.1
Glma.14G070000.1
Phvu.t009G013200.1
Viun.09g249600.1
Metr.1g023170.1
Trpr.57577mRNA31510
Frve.08479.1
Cusa.231820.1
Cusa.342750.1
Hean.5 00006593-RA
Hean.5 00006937-RA
Hean.10 00000061-RA
Lasa.1 v5 gn 1 16621.1
Lasa.1 v5 gn 5 25561.1
Migu.C00778.1
Soly.04g080910.1.1
Brra.C00826.1
Brra.B00770.1
Brra.J01724.1
Siir.8436
Siir.2204
Scpa.6g25870
Eusa.10015907m
Caru.10002425m
Bost.26527s0367.1
Arly.6G29700.t1
Arth.5G18450.1
Leal.324412
Aear.173865
Capa.supercontig 51.23
Thca.1EG026121t1
Trpr.57577mRNA32650
Metr.1g079630.1
Frve.16710.1
Migu.F01818.1
Soly.04g050750.1.1
Nenu.12674-RA
Nenu.08113-RA
Aqco.e6G312600.1
Zema.00008a003523 T01
Zema.00008a015832 T01
Sobi.007G162700.1
Sobi.001G378900.1
Seit.3G065700.1
Pahal.C01051.1
Pahal.H00921.1
Orsa.08g45110.1
Brdi.3g12680.1
Anco.010173.1
Muac.3P00320 001
Muac.Un randomP08690 001
Zoma.37g00200.1
Sppo.1G0014700
Amtr.scaffold00039.49
Semo.38502
1

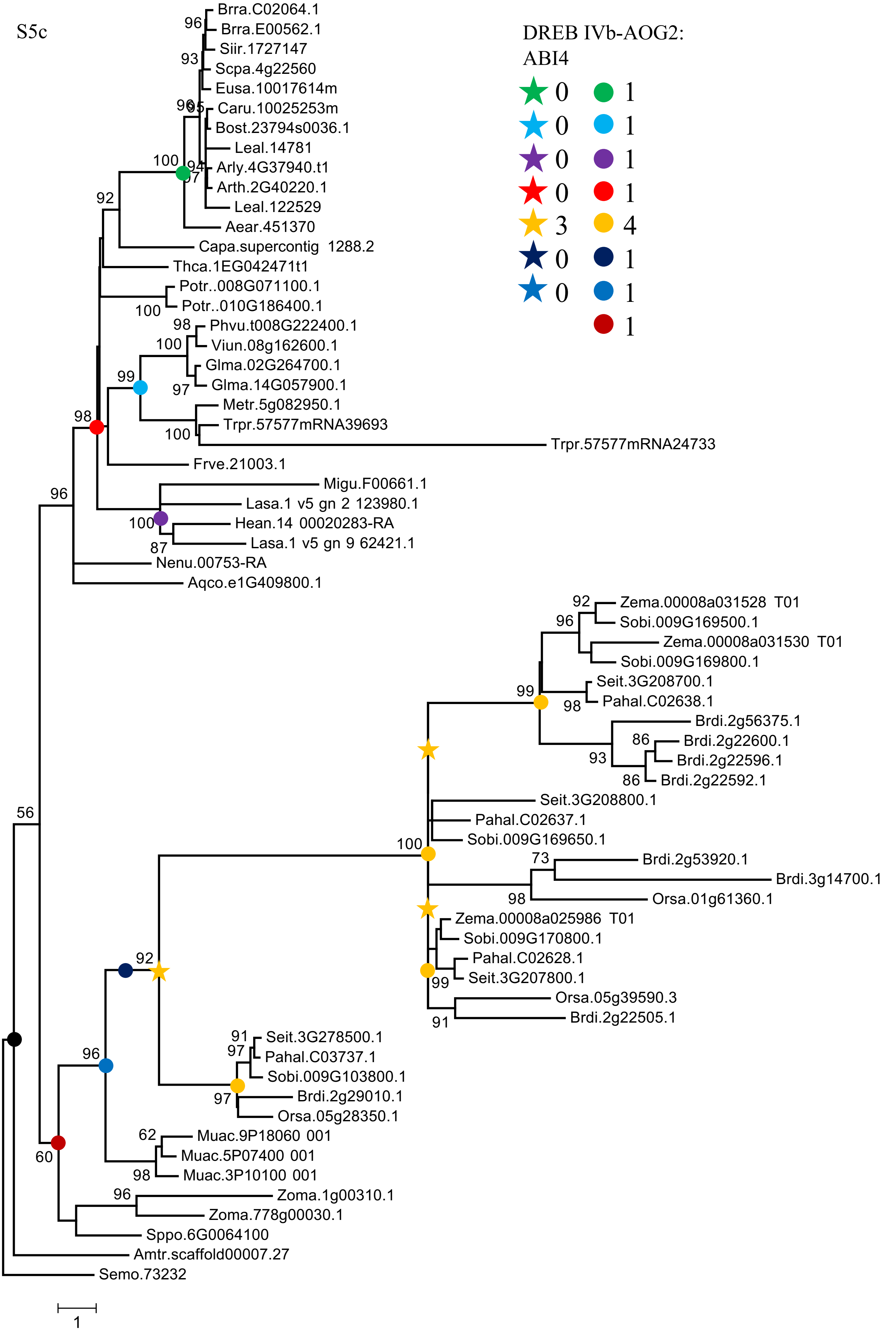
S5c
DREB IVb-AOG2:
ABI4
0 1
0 1
0 1
0 1
3 4
0 1
0 1
1
Brra.C02064.1
Brra.E00562.1
Siir.1727147
Scpa.4g22560
Eusa.10017614m
Caru.10025253m
Bost.23794s0036.1
Leal.14781
Arly.4G37940.t1
Arth.2G40220.1
Leal.122529
Aear.451370
Capa.supercontig 1288.2
Thca.1EG042471t1
Potr..008G071100.1
Potr..010G186400.1
Phvu.t008G222400.1
Viun.08g162600.1
Glma.02G264700.1
Glma.14G057900.1
Metr.5g082950.1
Trpr.57577mRNA39693
Trpr.57577mRNA24733
Frve.21003.1
Migu.F00661.1
Lasa.1 v5 gn 2 123980.1
Hean.14 00020283-RA
Lasa.1 v5 gn 9 62421.1
Nenu.00753-RA
Aqco.e1G409800.1
Zema.00008a031528 T01
Sobi.009G169500.1
Zema.00008a031530 T01
Sobi.009G169800.1
Seit.3G208700.1
Pahal.C02638.1
Brdi.2g56375.1
Brdi.2g22600.1
Brdi.2g22596.1
Brdi.2g22592.1
Seit.3G208800.1
Pahal.C02637.1
Sobi.009G169650.1
Brdi.2g53920.1
Brdi.3g14700.1
Orsa.01g61360.1
Zema.00008a025986 T01
Sobi.009G170800.1
Pahal.C02628.1
Seit.3G207800.1
Orsa.05g39590.3
Brdi.2g22505.1
Seit.3G278500.1
Pahal.C03737.1
Sobi.009G103800.1
Brdi.2g29010.1
Orsa.05g28350.1
Muac.9P18060 001
Muac.5P07400 001
Muac.3P10100 001
Zoma.1g00310.1
Zoma.778g00030.1
Sppo.6G0064100
Amtr.scaffold00007.27
Semo.73232
1

S5d

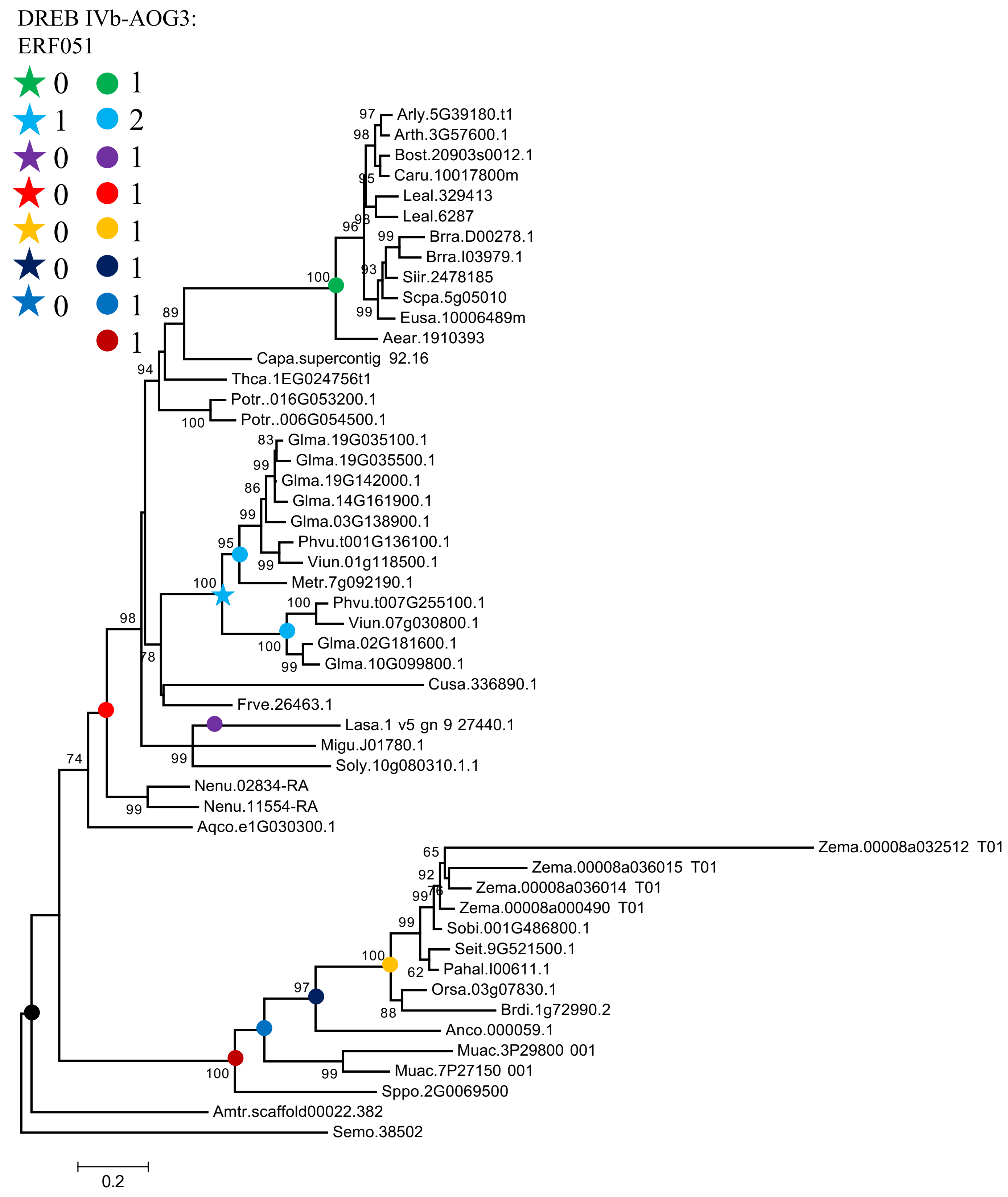

Fig. S5 Phylogenetic trees of DREB IV subfamily from representative Angiosperms. SH-aLRT supports above 50% are labeled on internal nodes. The labeling is the same as in Fig. S1.

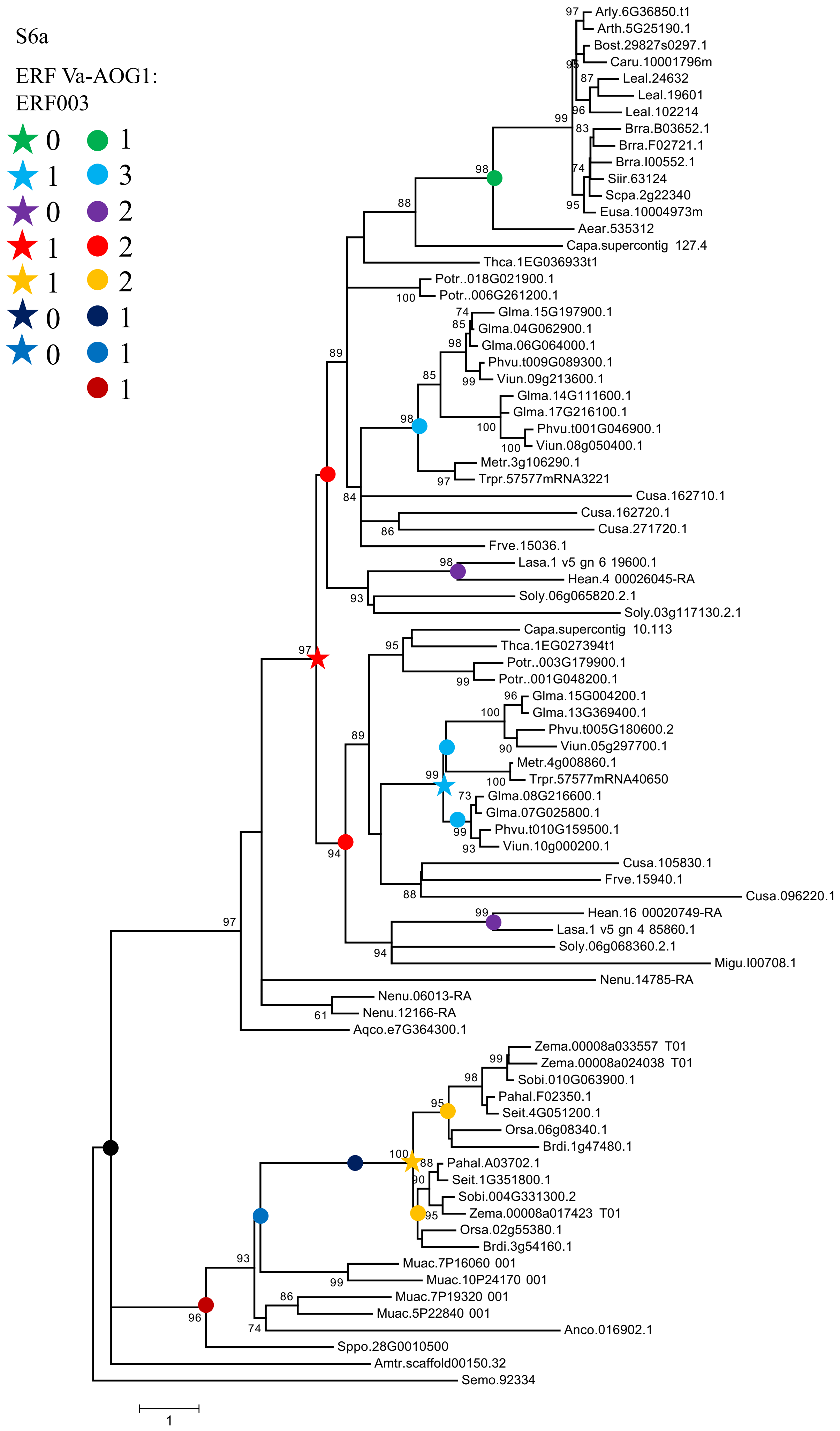
S6a
ERF Va-AOG1:
ERF003
0 1
1 3
0 2
1 2
1 2
0 1
0 1
1
Arly.6G36850.t1
Arth.5G25190.1
Bost.29827s0297.1
Caru.10001796m
Leal.24632
Leal.19601
Leal.102214
Brra.B03652.1
Brra.F02721.1
Brra.I00552.1
Siir.63124
Scpa.2g22340
Eusa.10004973m
Aear.535312
Capa.supercontig 127.4
Thca.1EG036933t1
Potr..018G021900.1
Potr..006G261200.1
Glma.15G197900.1
Glma.04G062900.1
Glma.06G064000.1
Phvu.t009G089300.1
Viun.09g213600.1
Glma.14G111600.1
Glma.17G216100.1
Phvu.t001G046900.1
Viun.08g050400.1
Metr.3g106290.1
Trpr.57577mRNA3221
Cusa.162710.1
Cusa.162720.1
Cusa.271720.1
Frve.15036.1
Lasa.1 v5 gn 6 19600.1
Hean.4 00026045-RA
Soly.06g065820.2.1
Soly.03g117130.2.1
Capa.supercontig 10.113
Thca.1EG027394t1
Potr..003G179900.1
Potr..001G048200.1
Glma.15G004200.1
Glma.13G369400.1
Phvu.t005G180600.2
Viun.05g297700.1
Metr.4g008860.1
Trpr.57577mRNA40650
Glma.08G216600.1
Glma.07G025800.1
Phvu.t010G159500.1
Viun.10g000200.1
Cusa.105830.1
Frve.15940.1
Cusa.096220.1
Hean.16 00020749-RA
Lasa.1 v5 gn 4 85860.1
Soly.06g068360.2.1
Migu.I00708.1
Nenu.14785-RA
Nenu.06013-RA
Nenu.12166-RA
Aqco.e7G364300.1
Zema.00008a033557 T01
Zema.00008a024038 T01
Sobi.010G063900.1
Pahal.F02350.1
Seit.4G051200.1
Orsa.06g08340.1
Brdi.1g47480.1
Pahal.A03702.1
Seit.1G351800.1
Sobi.004G331300.2
Zema.00008a017423 T01
Orsa.02g55380.1
Brdi.3g54160.1
Muac.7P16060 001
Muac.10P24170 001
Muac.7P19320 001
Muac.5P22840 001
Anco.016902.1
Sppo.28G0010500
Amtr.scaffold00150.32
Semo.92334
1

S6b

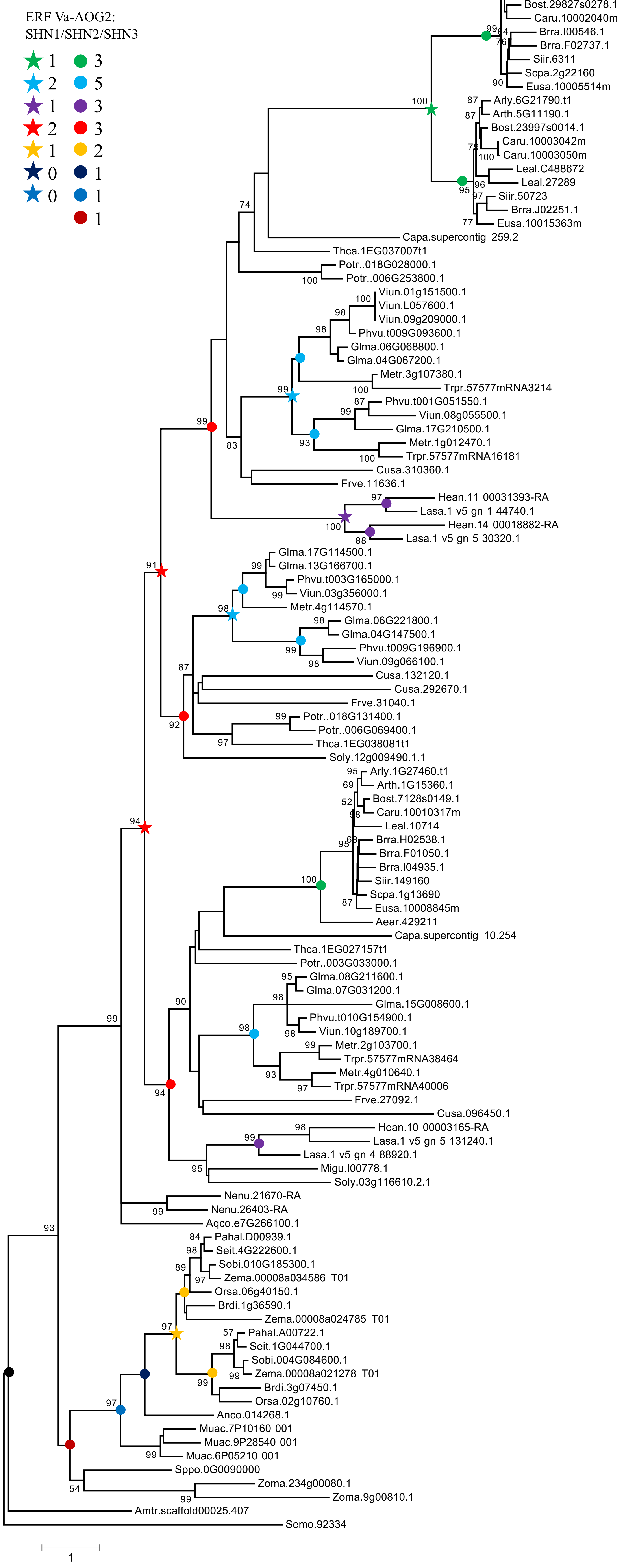
ERF Va-AOG2:
SHN1/SHN2/SHN3
1 3
2 5
1 3
2 3
1 2
0 1
0 1
1
Arly.6G37120.t1
Arth.5G25390.2
Bost.29827s0278.1
Caru.10002040m
Brra.I00546.1
Brra.F02737.1
Siir.6311
Scpa.2g22160
Eusa.10005514m
Arly.6G21790.t1
Arth.5G11190.1
Bost.23997s0014.1
Caru.10003042m
Caru.10003050m
Leal.C488672
Leal.27289
Siir.50723
Brra.J02251.1
Eusa.10015363m
Capa.supercontig 259.2
Thca.1EG037007t1
Potr..018G028000.1
Potr..006G253800.1
Viun.01g151500.1
Viun.L057600.1
Viun.09g209000.1
Phvu.t009G093600.1
Glma.06G068800.1
Glma.04G067200.1
Metr.3g107380.1
Trpr.57577mRNA3214
Phvu.t001G051550.1
Viun.08g055500.1
Glma.17G210500.1
Metr.1g012470.1
Trpr.57577mRNA16181
Cusa.310360.1
Frve.11636.1
Hean.11 00031393-RA
Lasa.1 v5 gn 1 44740.1
Hean.14 00018882-RA
Lasa.1 v5 gn 5 30320.1
Glma.17G114500.1
Glma.13G166700.1
Phvu.t003G165000.1
Viun.03g356000.1
Metr.4g114570.1
Glma.06G221800.1
Glma.04G147500.1
Phvu.t009G196900.1
Viun.09g066100.1
Cusa.132120.1
Cusa.292670.1
Frve.31040.1
Potr..018G131400.1
Potr..006G069400.1
Thca.1EG038081t1
Soly.12g009490.1.1
Arly.1G27460.t1
Arth.1G15360.1
Bost.7128s0149.1
Caru.10010317m
Leal.10714
Brra.H02538.1
Brra.F01050.1
Brra.I04935.1
Siir.149160
Scpa.1g13690
Eusa.10008845m
Aear.429211
Capa.supercontig 10.254
Thca.1EG027157t1
Potr..003G033000.1
Glma.08G211600.1
Glma.07G031200.1
Glma.15G008600.1
Phvu.t010G154900.1
Viun.10g189700.1
Metr.2g103700.1
Trpr.57577mRNA38464
Metr.4g010640.1
Trpr.57577mRNA40006
Frve.27092.1
Cusa.096450.1
Hean.10 00003165-RA
Lasa.1 v5 gn 5 131240.1
Lasa.1 v5 gn 4 88920.1
Migu.I00778.1
Soly.03g116610.2.1
Nenu.21670-RA
Nenu.26403-RA
Aqco.e7G266100.1
Pahal.D00939.1
Seit.4G222600.1
Sobi.010G185300.1
Zema.00008a034586 T01
Orsa.06g40150.1
Brdi.1g36590.1
Zema.00008a024785 T01
Pahal.A00722.1
Seit.1G044700.1
Sobi.004G084600.1
Zema.00008a021278 T01
Brdi.3g07450.1
Orsa.02g10760.1
Anco.014268.1
Muac.7P10160 001
Muac.9P28540 001
Muac.6P05210 001
Sppo.0G0090000
Zoma.234g00080.1
Zoma.9g00810.1
Amtr.scaffold00025.407
Semo.92334
1

S6c

ERF Vb-AOG1

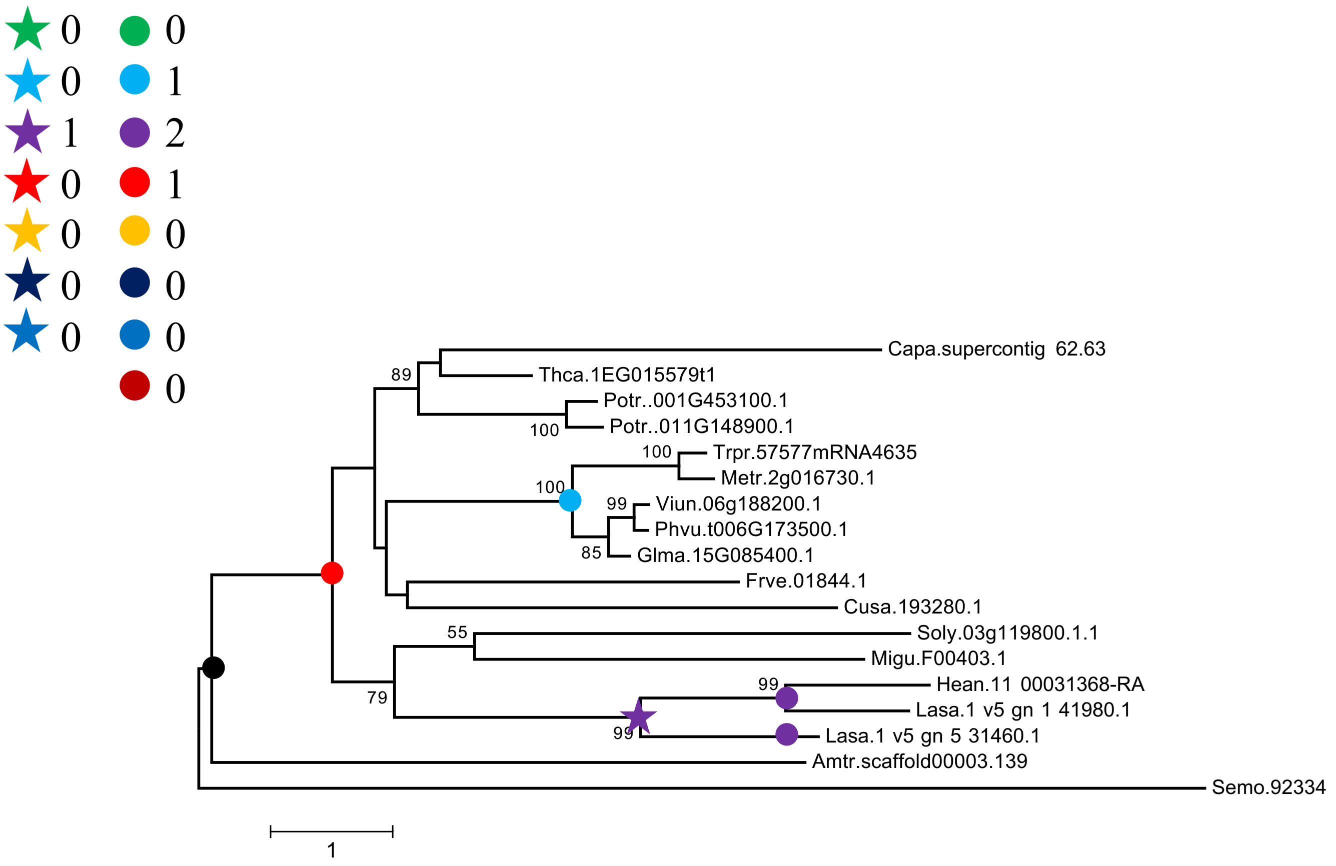

S6d

ERF Vb-AOG2

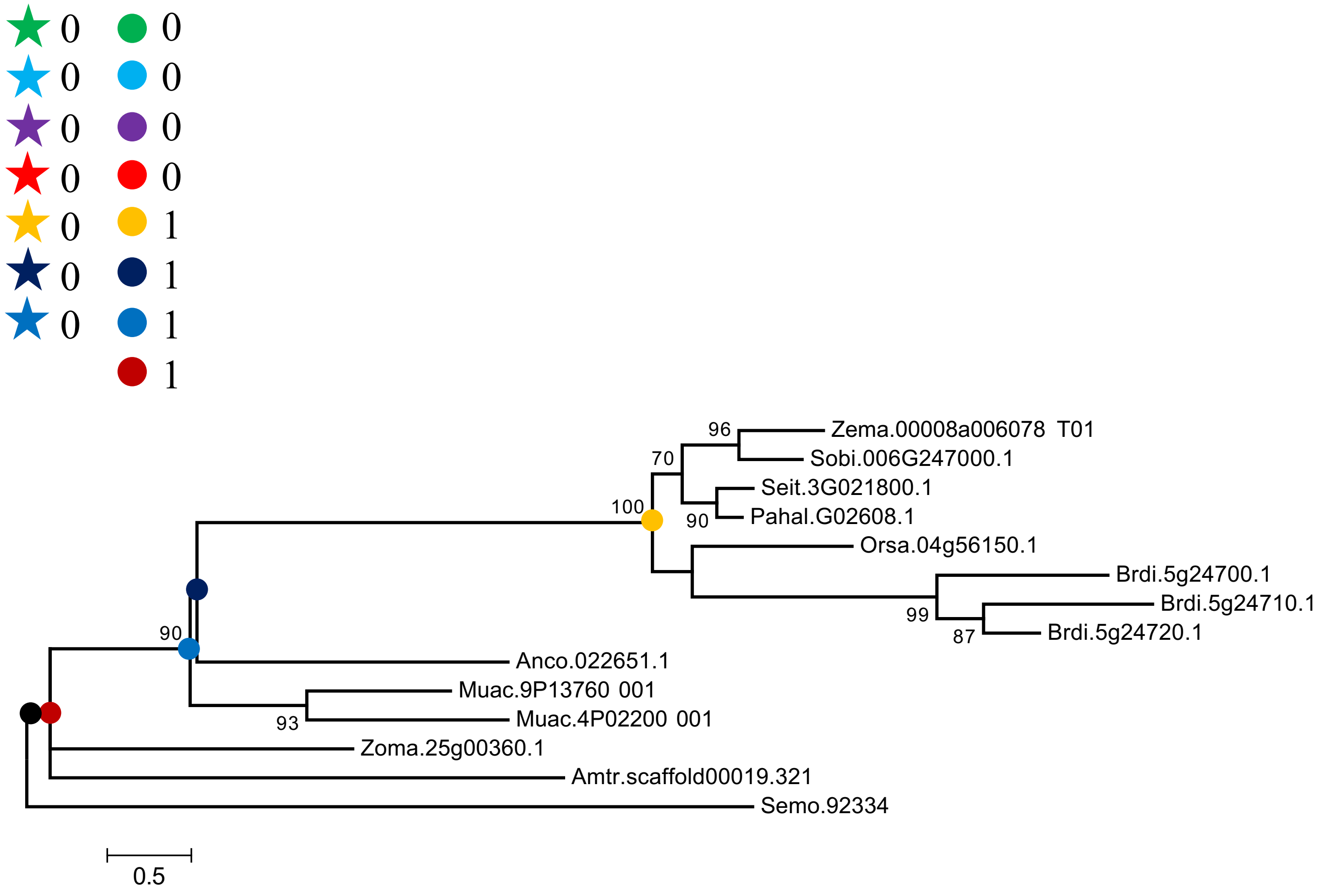

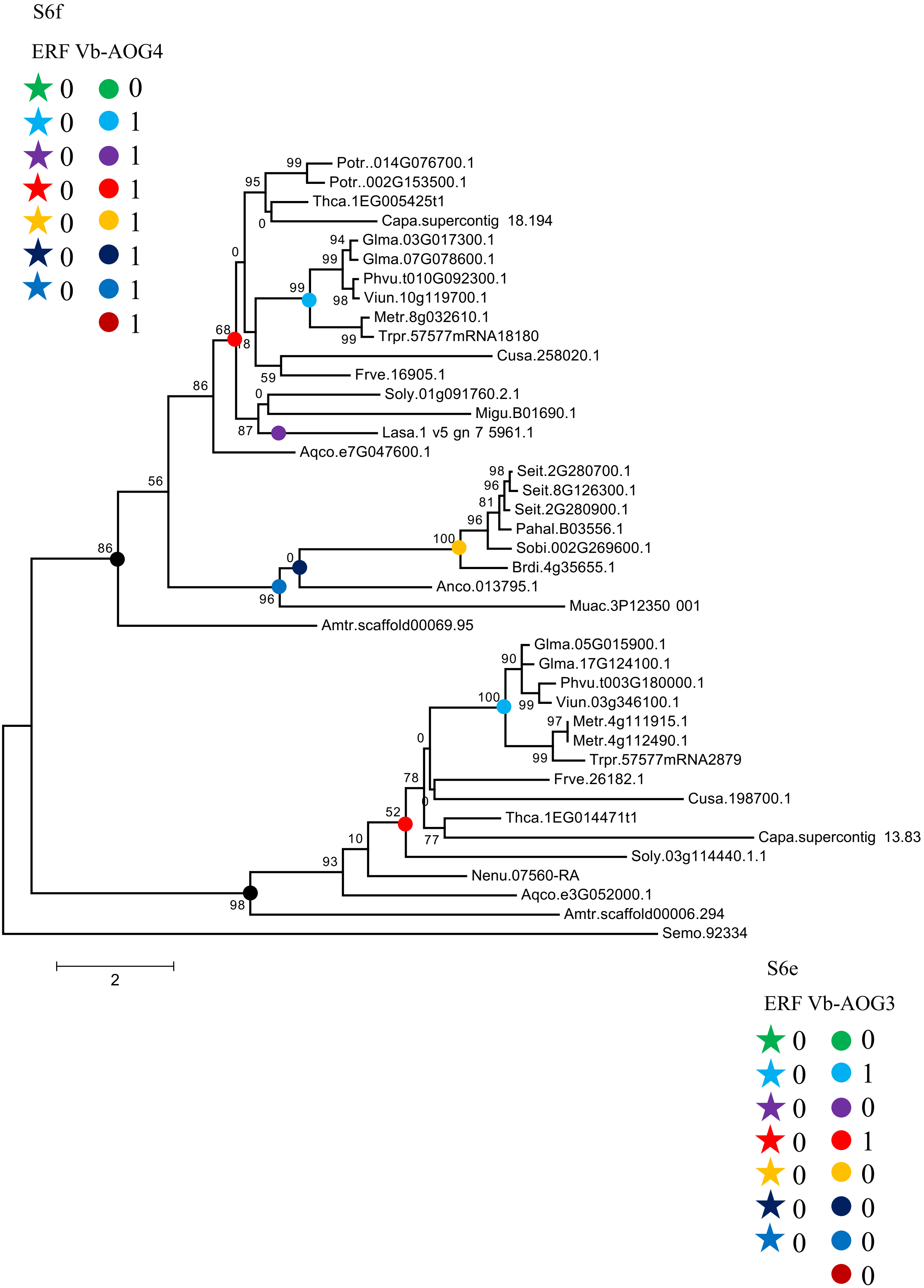
S6f
ERF Vb-AOG4
0 0
0 1
0 1
0 1
0 1
0 1
0 1
1
Potr..014G076700.1
Potr..002G153500.1
Thca.1EG005425t1
Capa.supercontig 18.194
Glma.03G017300.1
Glma.07G078600.1
Phvu.t010G092300.1
Viun.10g119700.1
Metr.8g032610.1
Trpr.57577mRNA18180
Cusa.258020.1
Frve.16905.1
Soly.01g091760.2.1
Migu.B01690.1
Lasa.1 v5 gn 7 5961.1
Aqco.e7G047600.1
Seit.2G280700.1
Seit.8G126300.1
Seit.2G280900.1
Pahal.B03556.1
Sobi.002G269600.1
Brdi.4g35655.1
Anco.013795.1
Muac.3P12350 001
Amtr.scaffold00069.95
Glma.05G015900.1
Glma.17G124100.1
Phvu.t003G180000.1
Viun.03g346100.1
Metr.4g111915.1
Metr.4g112490.1
Trpr.57577mRNA2879
Frve.26182.1
Cusa.198700.1
Thca.1EG014471t1
Capa.supercontig 13.83
Soly.03g114440.1.1
Nenu.07560-RA
Aqco.e3G052000.1
Amtr.scaffold00006.294
Semo.92334
2
S6e
ERF Vb-AOG3
0 0
0 1
0 0
0 1
0 0
0 0
0 0
0

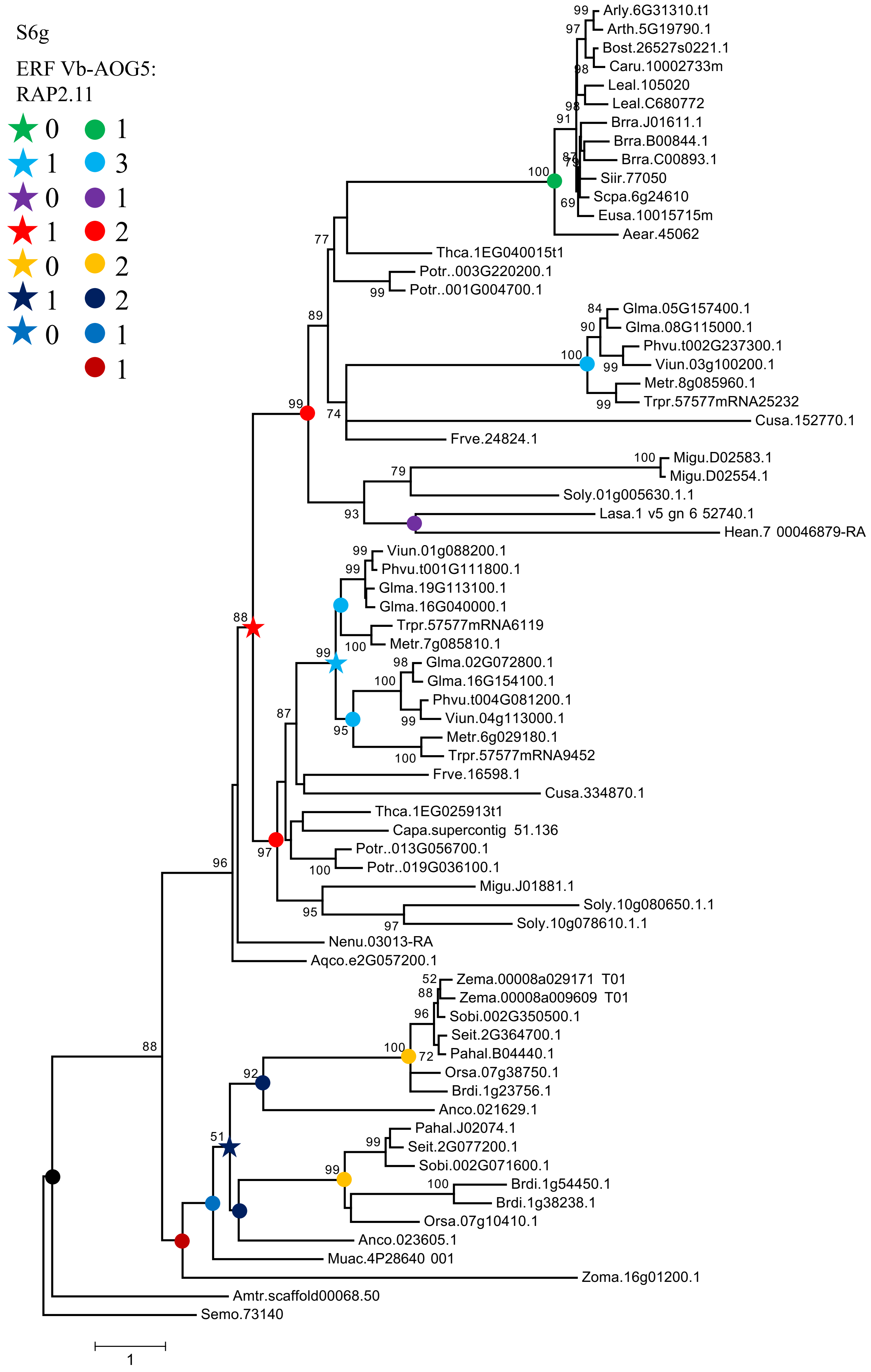
S6g
ERF Vb-AOG5:
RAP2.11
0 1
1 3
0 1
1 2
0 2
1 2
0 1
1
Arly.6G31310.t1
Arth.5G19790.1
Bost.26527s0221.1
Caru.10002733m
Leal.105020
Leal.C680772
Brra.J01611.1
Brra.B00844.1
Brra.C00893.1
Siir.77050
Scpa.6g24610
Eusa.10015715m
Aear.45062
Thca.1EG040015t1
Potr..003G220200.1
Potr..001G004700.1
Glma.05G157400.1
Glma.08G115000.1
Phvu.t002G237300.1
Viun.03g100200.1
Metr.8g085960.1
Trpr.57577mRNA25232
Cusa.152770.1
Frve.24824.1
Migu.D02583.1
Migu.D02554.1
Soly.01g005630.1.1
Lasa.1 v5 gn 6 52740.1
Hean.7 00046879-RA
Viun.01g088200.1
Phvu.t001G111800.1
Glma.19G113100.1
Glma.16G040000.1
Trpr.57577mRNA6119
Metr.7g085810.1
Glma.02G072800.1
Glma.16G154100.1
Phvu.t004G081200.1
Viun.04g113000.1
Metr.6g029180.1
Trpr.57577mRNA9452
Frve.16598.1
Cusa.334870.1
Thca.1EG025913t1
Capa.supercontig 51.136
Potr..013G056700.1
Potr..019G036100.1
Migu.J01881.1
Soly.10g080650.1.1
Soly.10g078610.1.1
Nenu.03013-RA
Aqco.e2G057200.1
Zema.00008a029171 T01
Zema.00008a009609 T01
Sobi.002G350500.1
Seit.2G364700.1
Pahal.B04440.1
Orsa.07g38750.1
Brdi.1g23756.1
Anco.021629.1
Pahal.J02074.1
Seit.2G077200.1
Sobi.002G071600.1
Brdi.1g54450.1
Brdi.1g38238.1
Orsa.07g10410.1
Anco.023605.1
Muac.4P28640 001
Zoma.16g01200.1
Amtr.scaffold00068.50
Semo.73140
1

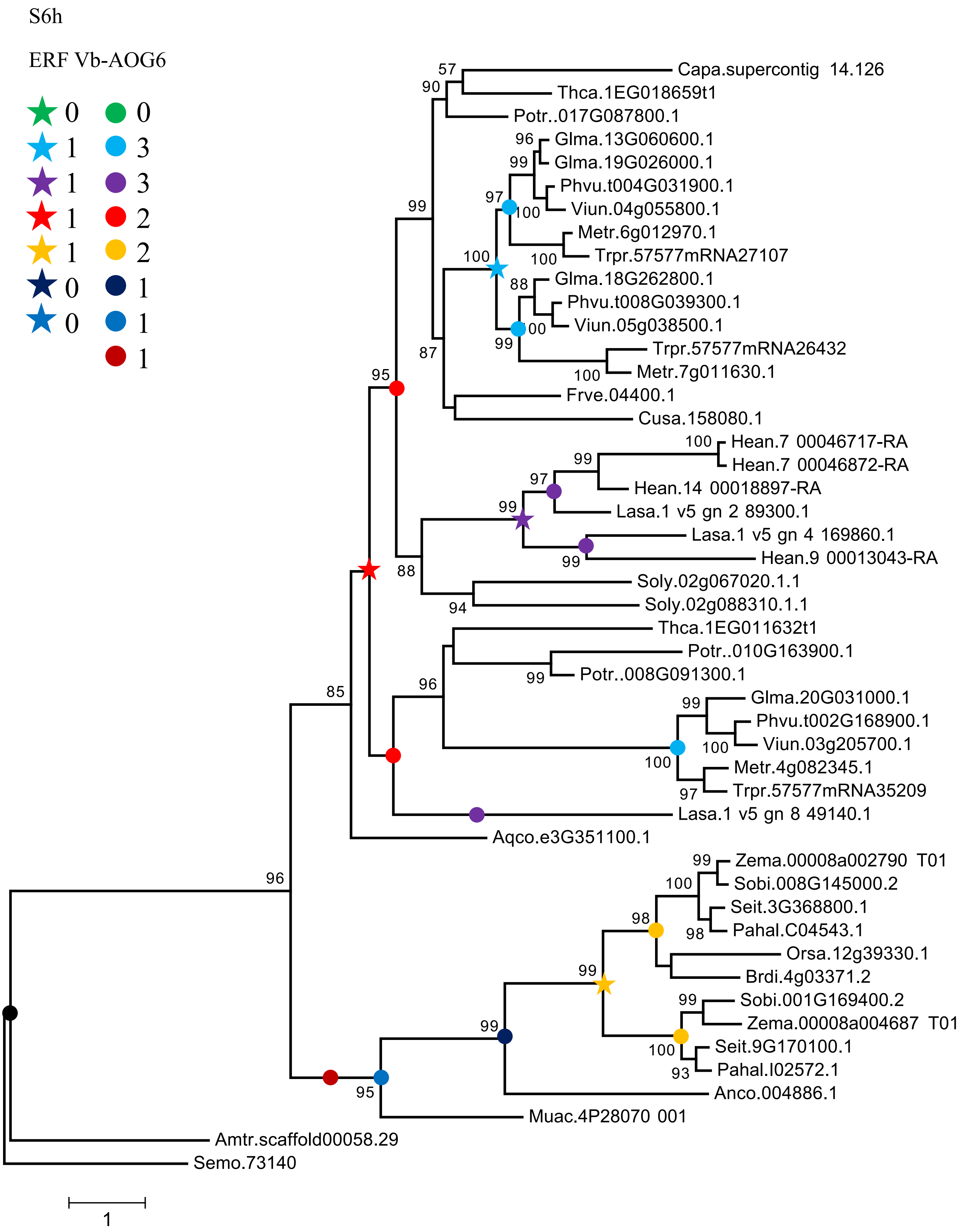

Fig. S6 Phylogenetic trees of ERF V subfamily from representative Angiosperms. SH-aLRT supports above 50% are labeled on internal nodes. The labeling is the same as in Fig. S1.

S7a

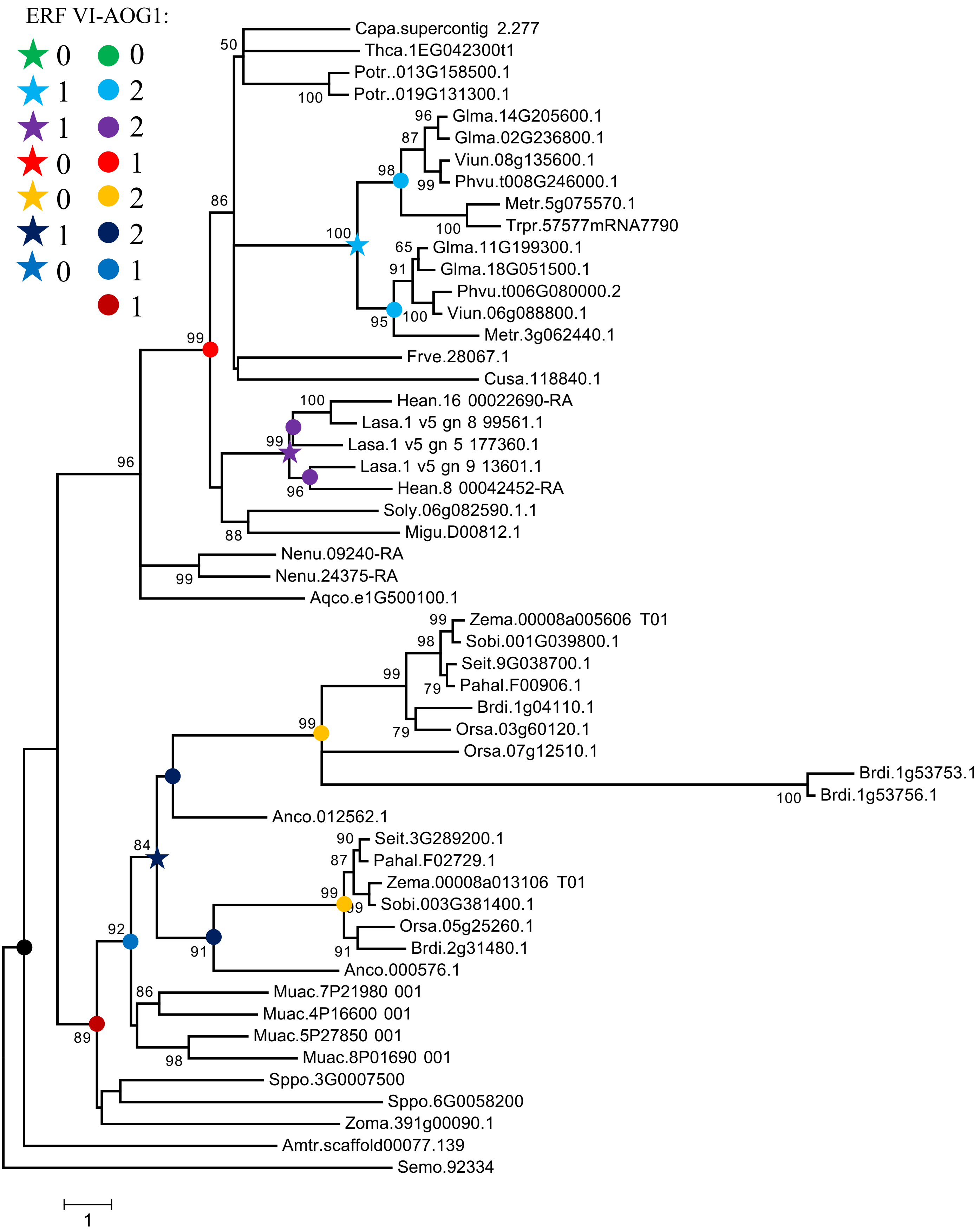

ERF VI-AOG1:
0
1
1
0
0
1
0
0
2
2
1
2
2
1
1
Capa.supercontig 2.277
Thca.1EG042300t1
Potr..013G158500.1
Potr..019G131300.1
Glma.14G205600.1
Glma.02G236800.1
Viun.08g135600.1
Phvu.t008G246000.1
Metr.5g075570.1
Trpr.57577mRNA7790
Glma.11G199300.1
Glma.18G051500.1
Phvu.t006G080000.2
Viun.06g088800.1
Metr.3g062440.1
Frve.28067.1
Cusa.118840.1
Hean.16 00022690-RA
Lasa.1 v5 gn 8 99561.1
Lasa.1 v5 gn 5 177360.1
Lasa.1 v5 gn 9 13601.1
Hean.8 00042452-RA
Soly.06g082590.1.1
Migu.D00812.1
Nenu.09240-RA
Nenu.24375-RA
Aqco.e1G500100.1
Zema.00008a005606 T01
Sobi.001G039800.1
Seit.9G038700.1
Pahal.F00906.1
Brdi.1g04110.1
Orsa.03g60120.1
Orsa.07g12510.1
Brdi.1g53753.1
Brdi.1g53756.1
Anco.012562.1
Seit.3G289200.1
Pahal.F02729.1
Zema.00008a013106 T01
Sobi.003G381400.1
Orsa.05g25260.1
Brdi.2g31480.1
Anco.000576.1
Muac.7P21980 001
Muac.4P16600 001
Muac.5P27850 001
Muac.8P01690 001
Sppo.3G0007500
Sppo.6G0058200
Zoma.391g00090.1
Amtr.scaffold00077.139
Semo.92334
1

S7b

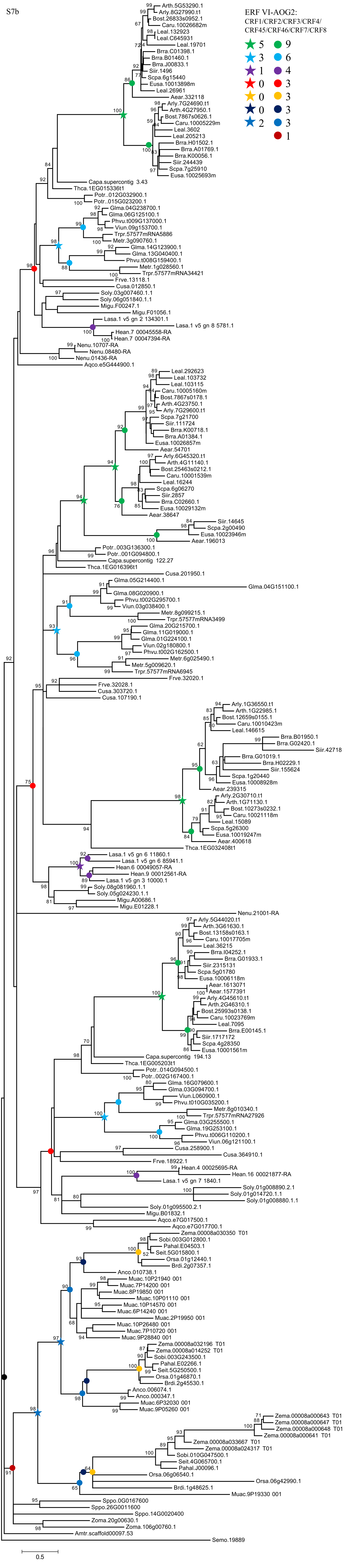
ERF VI-AOG2:
CRF1/CRF2/CRF3/CRF4/
CRF45/CRF46/CRF7/CRF8
5
3
1
0
0
0
2
9
6
4
3
3
3
3
1
0.5

S7c

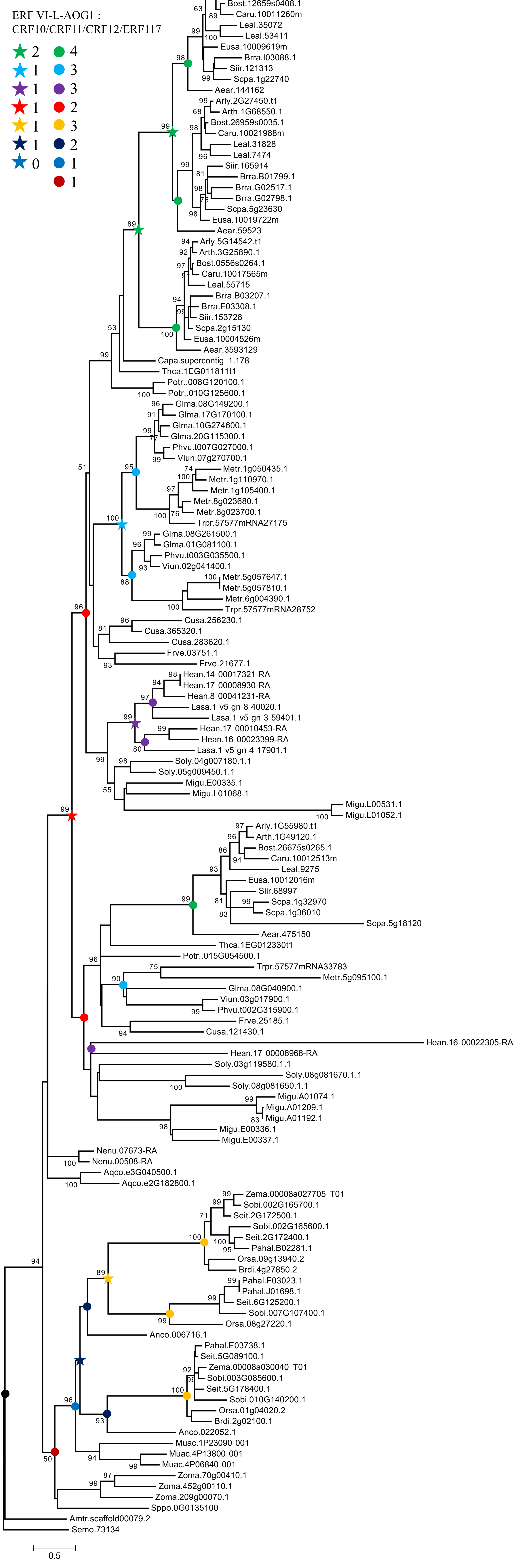

ERF VI-L-AOG1 :
CRF10/CRF11/CRF12/ERF117
2 4
1 3
1 3
1 2
1 3
1 2
0 1
1
Arly.1G39830.t1
Arth.1G25470.1
Bost.12659s0408.1
Caru.10011260m
Leal.35072
Leal.53411
Eusa.10009619m
Brra.I03088.1
Siir.121313
Scpa.1g22740
Aear.144162
Arly.2G27450.t1
Arth.1G68550.1
Bost.26959s0035.1
Caru.10021988m
Leal.31828
Leal.7474
Siir.165914
Brra.B01799.1
Brra.G02517.1
Brra.G02798.1
Scpa.5g23630
Eusa.10019722m
Aear.59523
Arly.5G14542.t1
Arth.3G25890.1
Bost.0556s0264.1
Caru.10017565m
Leal.55715
Brra.B03207.1
Brra.F03308.1
Siir.153728
Scpa.2g15130
Eusa.10004526m
Aear.3593129
Capa.supercontig 1.178
Thca.1EG011811t1
Potr..008G120100.1
Potr..010G125600.1
Glma.08G149200.1
Glma.17G170100.1
Glma.10G274600.1
Glma.20G115300.1
Phvu.t007G027000.1
Viun.07g270700.1
Metr.1g050435.1
Metr.1g110970.1
Metr.1g105400.1
Metr.8g023680.1
Metr.8g023700.1
Trpr.57577mRNA27175
Glma.08G261500.1
Glma.01G081100.1
Phvu.t003G035500.1
Viun.02g041400.1
Metr.5g057647.1
Metr.5g057810.1
Metr.6g004390.1
Trpr.57577mRNA28752
Cusa.256230.1
Cusa.365320.1
Cusa.283620.1
Frve.03751.1
Frve.21677.1
Hean.14 00017321-RA
Hean.17 00008930-RA
Hean.8 00041231-RA
Lasa.1 v5 gn 8 40020.1
Lasa.1 v5 gn 3 59401.1
Hean.17 00010453-RA
Hean.16 00023399-RA
Lasa.1 v5 gn 4 17901.1
Soly.04g007180.1.1
Soly.05g009450.1.1
Migu.E00335.1
Migu.L01068.1
Migu.L00531.1
Migu.L01052.1
Arly.1G55980.t1
Arth.1G49120.1
Bost.26675s0265.1
Caru.10012513m
Leal.9275
Eusa.10012016m
Siir.68997
Scpa.1g32970
Scpa.1g36010
Scpa.5g18120
Aear.475150
Thca.1EG012330t1
Potr..015G054500.1
Trpr.57577mRNA33783
Metr.5g095100.1
Glma.08G040900.1
Viun.03g017900.1
Phvu.t002G315900.1
Frve.25185.1
Cusa.121430.1
Hean.16 00022305-RA
Hean.17 00008968-RA
Soly.03g119580.1.1
Soly.08g081670.1.1
Soly.08g081650.1.1
Migu.A01074.1
Migu.A01209.1
Migu.A01192.1
Migu.E00336.1
Migu.E00337.1
Nenu.07673-RA
Nenu.00508-RA
Aqco.e3G040500.1
Aqco.e2G182800.1
Zema.00008a027705 T01
Sobi.002G165700.1
Seit.2G172500.1
Sobi.002G165600.1
Seit.2G172400.1
Pahal.B02281.1
Orsa.09g13940.2
Brdi.4g27850.2
Pahal.F03023.1
Pahal.J01698.1
Seit.6G125200.1
Sobi.007G107400.1
Orsa.08g27220.1
Anco.006716.1
Pahal.E03738.1
Seit.5G089100.1
Zema.00008a030040 T01
Sobi.003G085600.1
Seit.5G178400.1
Sobi.010G140200.1
Orsa.01g04020.2
Brdi.2g02100.1
Anco.022052.1
Muac.1P23090 001
Muac.4P13800 001
Muac.4P06840 001
Zoma.70g00410.1
Zoma.452g00110.1
Zoma.209g00070.1
Sppo.0G0135100
Amtr.scaffold00079.2
Semo.73134
0.5

S8a

ERF VIIa-AOG1 :
RAP2.3

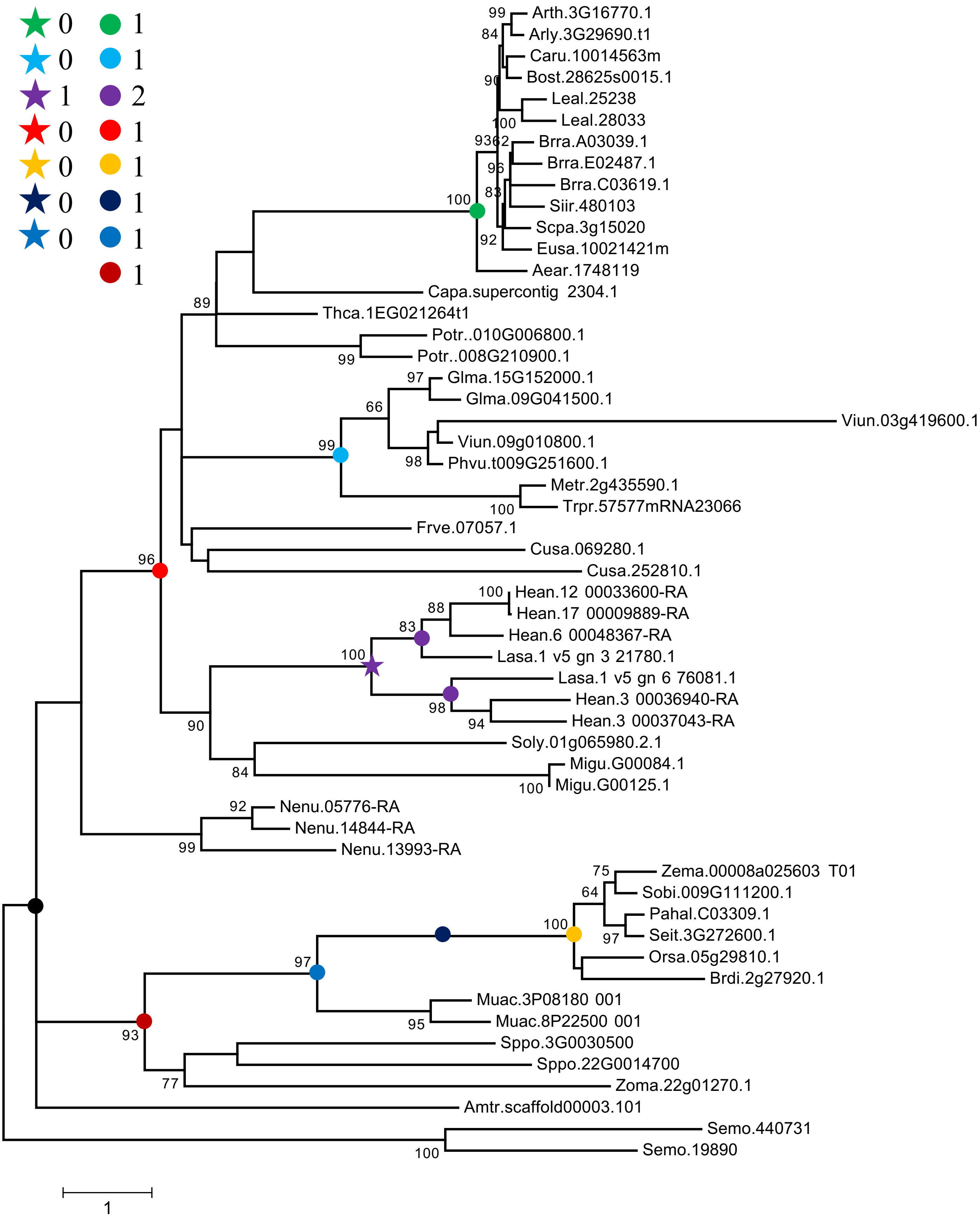

S8b

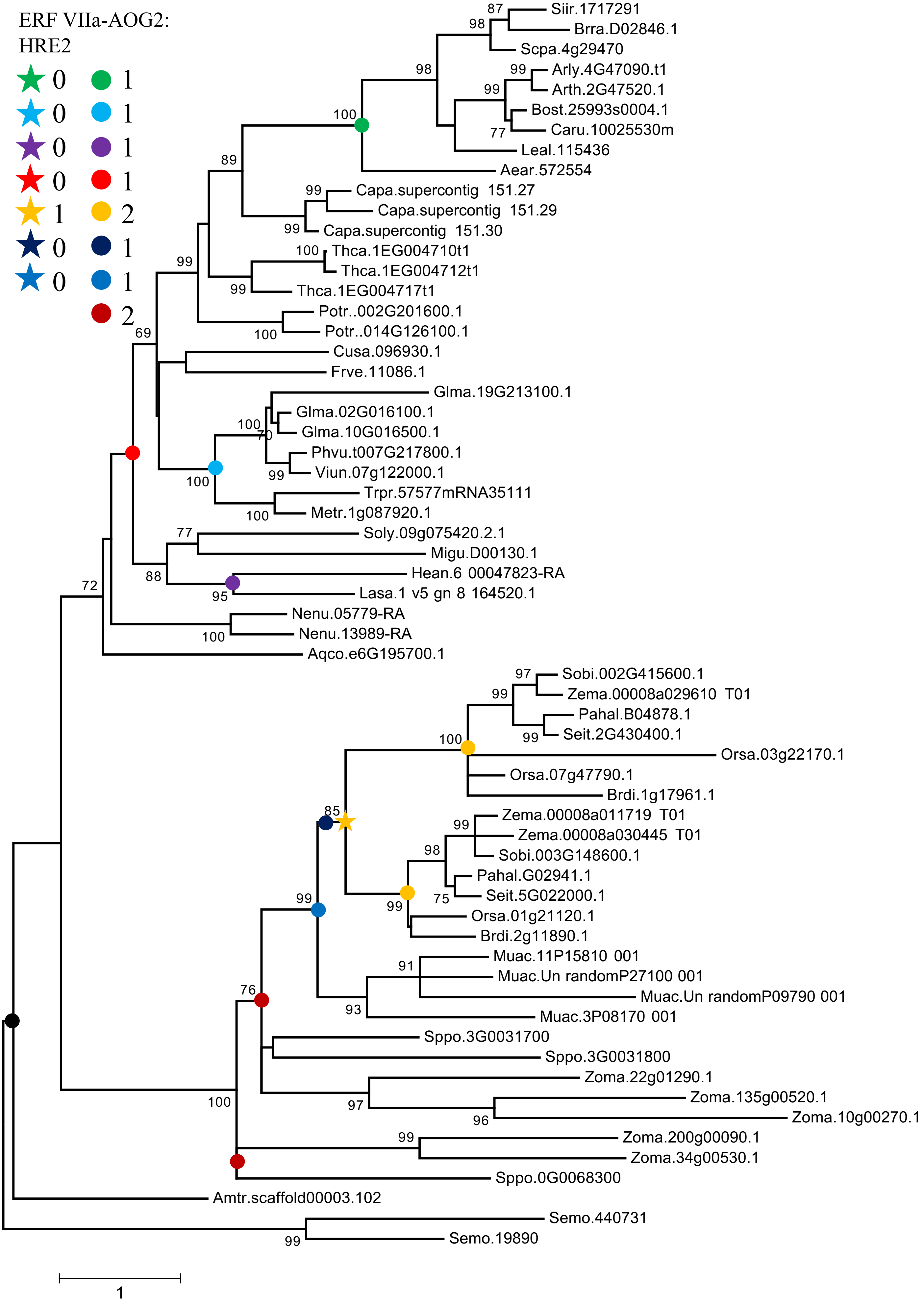
ERF VIIa-AOG2:
HRE2
Siir.1717291
Brra.D02846.1
Scpa.4g29470
Arly.4G47090.t1
Arth.2G47520.1
Bost.25993s0004.1
Caru.10025530m
Leal.115436
Aear.572554
Capa.supercontig 151.27
Capa.supercontig 151.29
Capa.supercontig 151.30
Thca.1EG004710t1
Thca.1EG004712t1
Thca.1EG004717t1
Potr..002G201600.1
Potr..014G126100.1
Cusa.096930.1
Frve.11086.1
Glma.19G213100.1
Glma.02G016100.1
Glma.10G016500.1
Phvu.t007G217800.1
Viun.07g122000.1
Trpr.57577mRNA35111
Metr.1g087920.1
Soly.09g075420.2.1
Migu.D00130.1
Hean.6 00047823-RA
Lasa.1 v5 gn 8 164520.1
Nenu.05779-RA
Nenu.13989-RA
Aqco.e6G195700.1
Sobi.002G415600.1
Zema.00008a029610 T01
Pahal.B04878.1
Seit.2G430400.1
Orsa.03g22170.1
Orsa.07g47790.1
Brdi.1g17961.1
Zema.00008a011719 T01
Zema.00008a030445 T01
Sobi.003G148600.1
Pahal.G02941.1
Seit.5G022000.1
Orsa.01g21120.1
Brdi.2g11890.1
Muac.11P15810 001
Muac.Un randomP27100 001
Muac.Un randomP09790 001
Muac.3P08170 001
Sppo.3G0031700
Sppo.3G0031800
Zoma.22g01290.1
Zoma.135g00520.1
Zoma.10g00270.1
Zoma.200g00090.1
Zoma.34g00530.1
Sppo.0G0068300
Amtr.scaffold00003.102
Semo.440731
Semo.19890
1

S8c

ERF VIIa-AOG3 :
HRE1

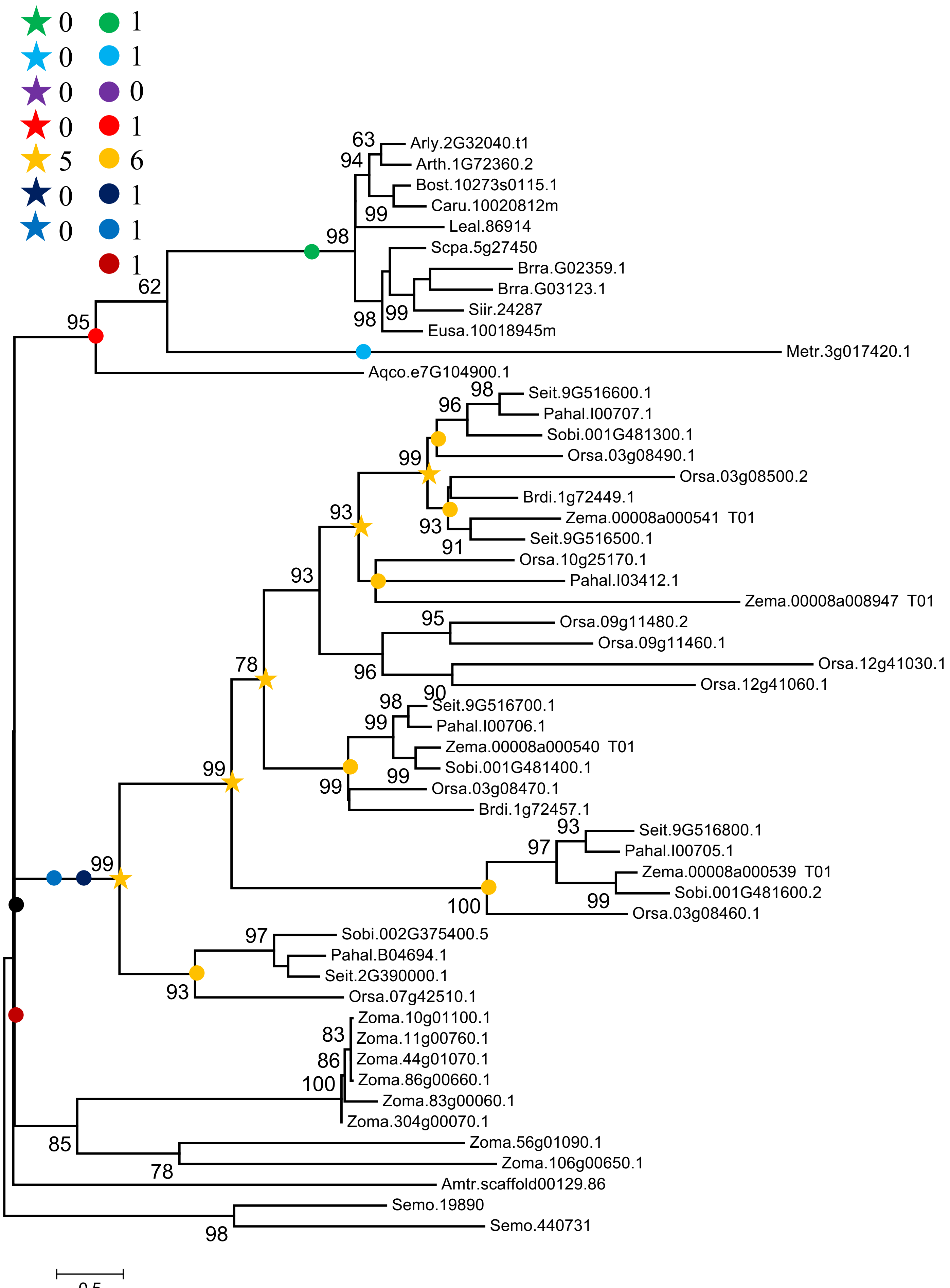

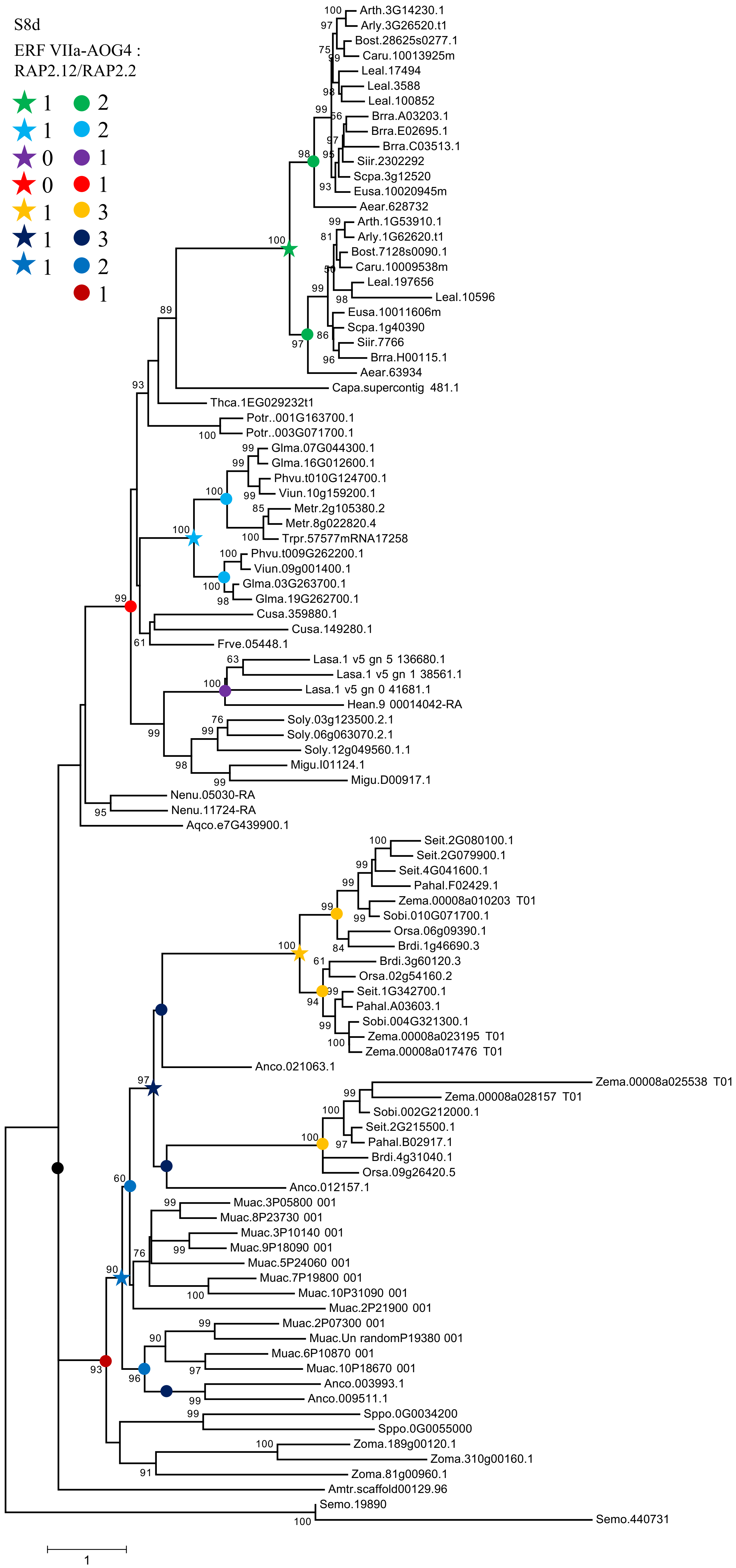

Fig. S8 Phylogenetic trees of ERF VII subfamily from representative Angiosperms. SH-aLRT supports above 50% are labeled on internal nodes. The labeling is the same as in Fig. S1.

S9a

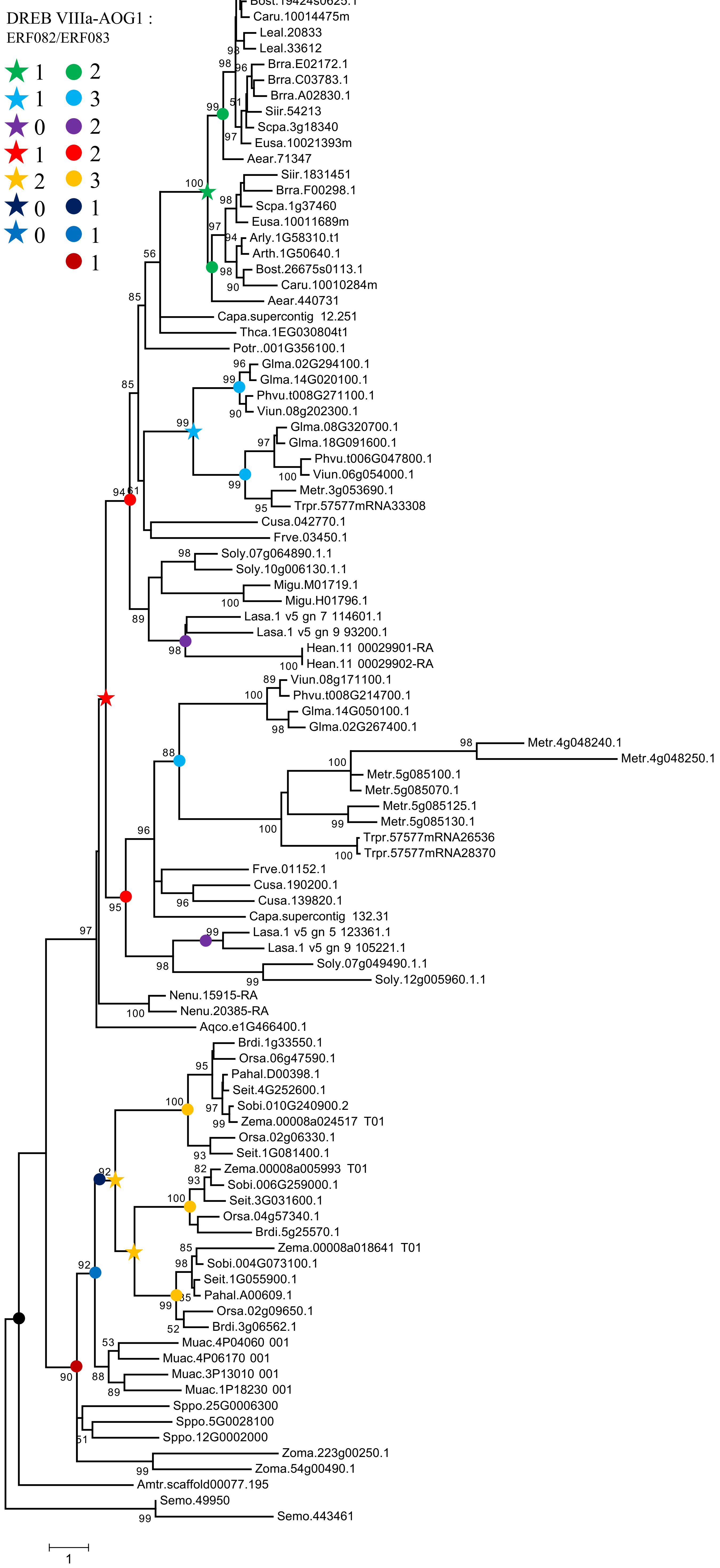
DREB VIIIa-AOG1 :
ERF082/ERF083
1 2
1 3
0 2
1 2
2 3
0 1
0 1
1
Arth.3G20310.1
Arly.3G34030.t1
Bost.19424s0625.1
Caru.10014475m
Leal.20833
Leal.33612
Brra.E02172.1
Brra.C03783.1
Brra.A02830.1
Siir.54213
Scpa.3g18340
Eusa.10021393m
Aear.71347
Siir.1831451
Brra.F00298.1
Scpa.1g37460
Eusa.10011689m
Arly.1G58310.t1
Arth.1G50640.1
Bost.26675s0113.1
Caru.10010284m
Aear.440731
Capa.supercontig 12.251
Thca.1EG030804t1
Potr..001G356100.1
Glma.02G294100.1
Glma.14G020100.1
Phvu.t008G271100.1
Viun.08g202300.1
Glma.08G320700.1
Glma.18G091600.1
Phvu.t006G047800.1
Viun.06g054000.1
Metr.3g053690.1
Trpr.57577mRNA33308
Cusa.042770.1
Frve.03450.1
Soly.07g064890.1.1
Soly.10g006130.1.1
Migu.M01719.1
Migu.H01796.1
Lasa.1 v5 gn 7 114601.1
Lasa.1 v5 gn 9 93200.1
Hean.11 00029901-RA
Hean.11 00029902-RA
Viun.08g171100.1
Phvu.t008G214700.1
Glma.14G050100.1
Glma.02G267400.1
Metr.4g048240.1
Metr.4g048250.1
Metr.5g085100.1
Metr.5g085070.1
Metr.5g085125.1
Metr.5g085130.1
Trpr.57577mRNA26536
Trpr.57577mRNA28370
Frve.01152.1
Cusa.190200.1
Cusa.139820.1
Capa.supercontig 132.31
Lasa.1 v5 gn 5 123361.1
Lasa.1 v5 gn 9 105221.1
Soly.07g049490.1.1
Soly.12g005960.1.1
Nenu.15915-RA
Nenu.20385-RA
Aqco.e1G466400.1
Brdi.1g33550.1
Orsa.06g47590.1
Pahal.D00398.1
Seit.4G252600.1
Sobi.010G240900.2
Zema.00008a024517 T01
Orsa.02g06330.1
Seit.1G081400.1
Zema.00008a005993 T01
Sobi.006G259000.1
Seit.3G031600.1
Orsa.04g57340.1
Brdi.5g25570.1
Zema.00008a018641 T01
Sobi.004G073100.1
Seit.1G055900.1
Pahal.A00609.1
Orsa.02g09650.1
Brdi.3g06562.1
Muac.4P04060 001
Muac.4P06170 001
Muac.3P13010 001
Muac.1P18230 001
Sppo.25G0006300
Sppo.5G0028100
Sppo.12G0002000
Zoma.223g00250.1
Zoma.54g00490.1
Amtr.scaffold00077.195
Semo.49950
Semo.443461
1

S9b

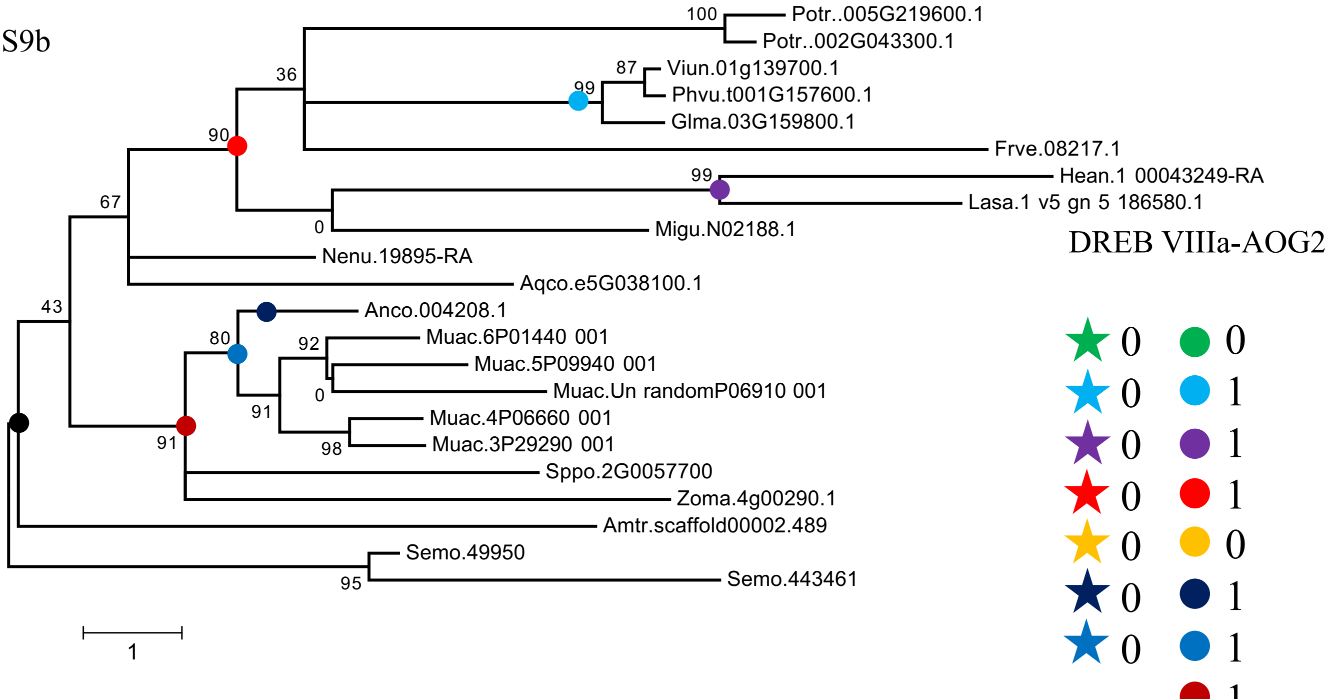

S9c

ERF VIIIa-AOG3 : ERF081

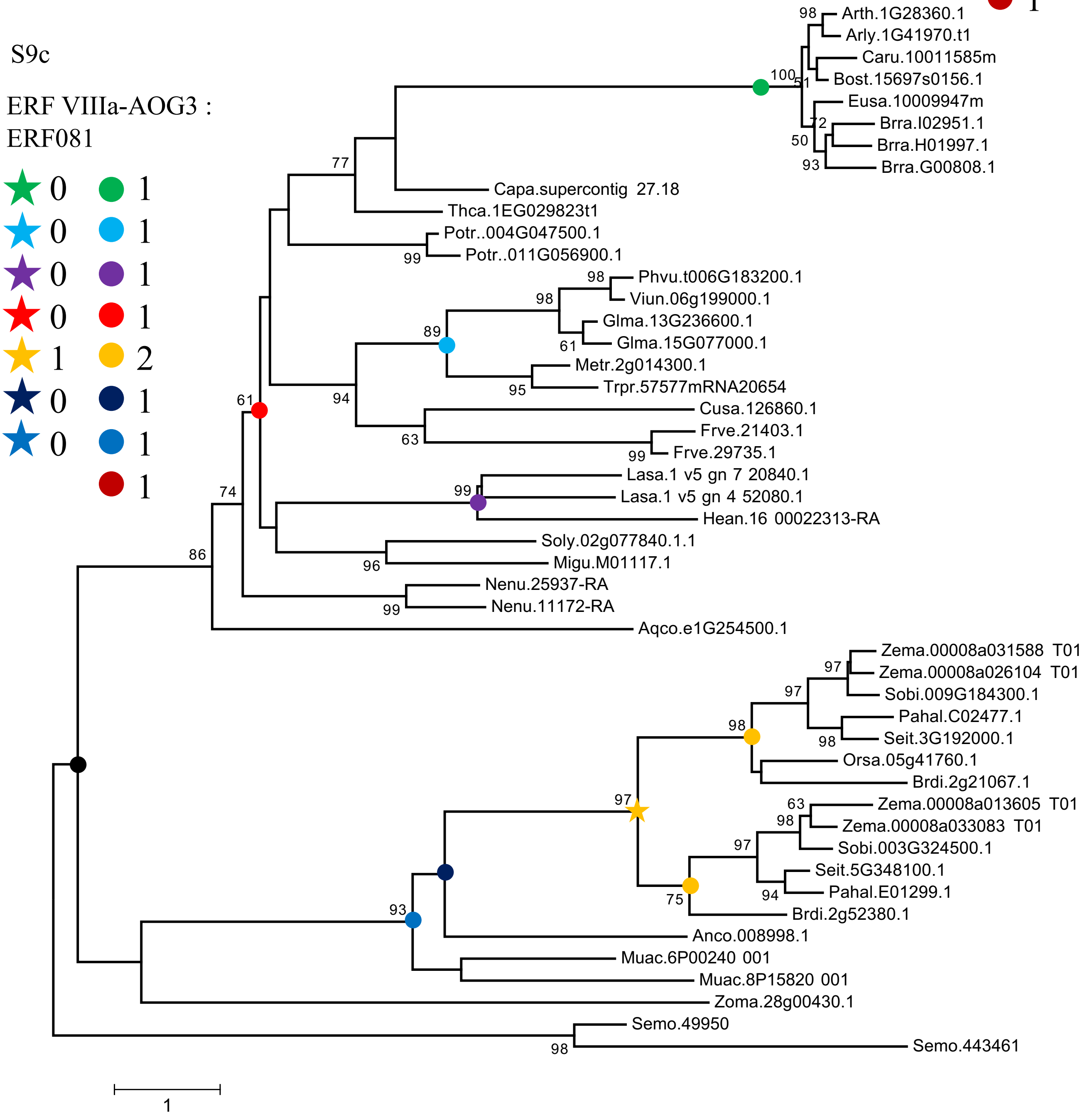

S9d

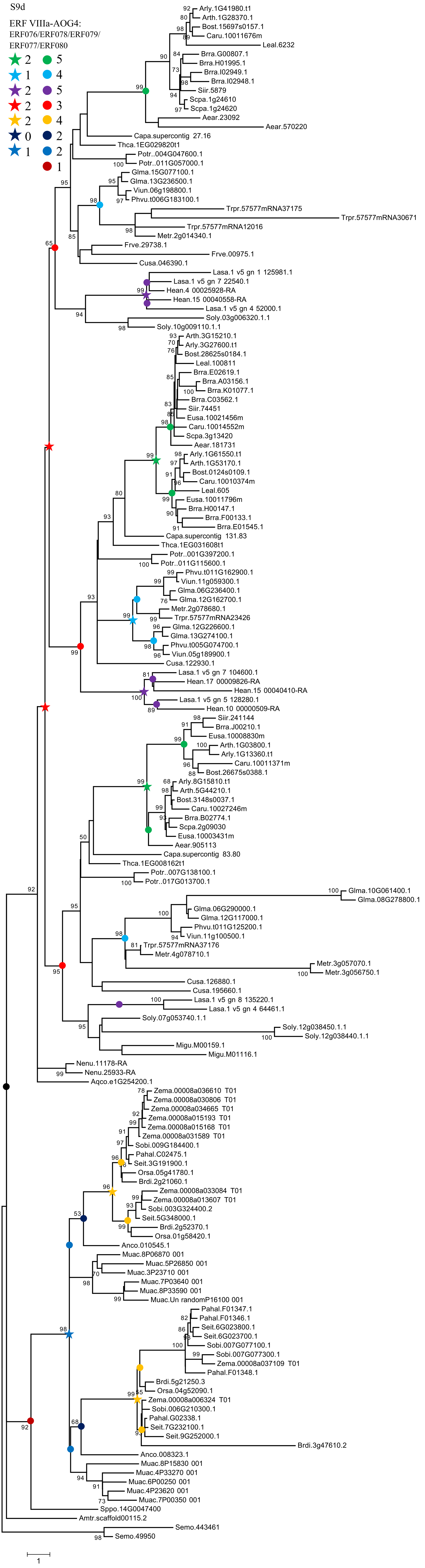
ERF VIIIa-AOG4:
ERF076/ERF078/ERF079/
ERF077/ERF080
2 5
1 4
2 5
2 3
2 4
0 2
1 2
1
Arly.1G41980.t1
Arth.1G28370.1
Bost.15697s0157.1
Caru.10011676m
Leal.6232
Brra.G00807.1
Brra.H01995.1
Brra.I02949.1
Brra.I02948.1
Siir.5879
Scpa.1g24610
Scpa.1g24620
Aear.23092
Aear.570220
Capa.supercontig 27.16
Thca.1EG029820t1
Potr..004G047600.1
Potr..011G057000.1
Glma.15G077100.1
Glma.13G236500.1
Viun.06g198800.1
Phvu.t006G183100.1
Trpr.57577mRNA37175
Trpr.57577mRNA30671
Trpr.57577mRNA12016
Metr.2g014340.1
Frve.29738.1
Frve.00975.1
Cusa.046390.1
Lasa.1 v5 gn 1 125981.1
Lasa.1 v5 gn 7 22540.1
Hean.4 00025928-RA
Hean.15 00040558-RA
Lasa.1 v5 gn 4 52000.1
Soly.03g006320.1.1
Soly.10g009110.1.1
Arth.3G15210.1
Arly.3G27600.t1
Bost.28625s0184.1
Leal.100811
Brra.E02619.1
Brra.A03156.1
Brra.K01077.1
Brra.C03562.1
Siir.74451
Eusa.10021456m
Caru.10014552m
Scpa.3g13420
Aear.181731
Arly.1G61550.t1
Arth.1G53170.1
Bost.0124s0109.1
Caru.10010374m
Leal.605
Eusa.10011796m
Brra.H00147.1
Brra.F00133.1
Brra.E01545.1
Capa.supercontig 131.83
Thca.1EG031608t1
Potr..001G397200.1
Potr..011G115600.1
Phvu.t011G162900.1
Viun.11g059300.1
Glma.06G236400.1
Glma.12G162700.1
Metr.2g078680.1
Trpr.57577mRNA23426
Glma.12G226600.1
Glma.13G274100.1
Phvu.t005G074700.1
Viun.05g189900.1
Cusa.122930.1
Lasa.1 v5 gn 7 104600.1
Hean.17 00009826-RA
Hean.15 00040410-RA
Lasa.1 v5 gn 5 128280.1
Hean.10 00000509-RA
Siir.241144
Brra.J00210.1
Eusa.10008830m
Arth.1G03800.1
Arly.1G13360.t1
Caru.10011371m
Bost.26675s0388.1
Arly.8G15810.t1
Arth.5G44210.1
Bost.3148s0037.1
Caru.10027246m
Brra.B02774.1
Scpa.2g09030
Eusa.10003431m
Aear.905113
Capa.supercontig 83.80
Thca.1EG008162t1
Potr..007G138100.1
Potr..017G013700.1
Glma.10G061400.1
Glma.08G278800.1
Glma.06G290000.1
Glma.12G117000.1
Phvu.t011G125200.1
Viun.11g100500.1
Trpr.57577mRNA37176
Metr.4g078710.1
Metr.3g057070.1
Metr.3g056750.1
Cusa.126880.1
Cusa.195660.1
Lasa.1 v5 gn 8 135220.1
Lasa.1 v5 gn 4 64461.1
Soly.07g053740.1.1
Soly.12g038450.1.1
Soly.12g038440.1.1
Migu.M00159.1
Migu.M01116.1
Nenu.11178-RA
Nenu.25933-RA
Aqco.e1G254200.1
Zema.00008a036610 T01
Zema.00008a030806 T01
Zema.00008a034665 T01
Zema.00008a015193 T01
Zema.00008a015168 T01
Zema.00008a031589 T01
Sobi.009G184400.1
Pahal.C02475.1
Seit.3G191900.1
Orsa.05g41780.1
Brdi.2g21060.1
Zema.00008a033084 T01
Zema.00008a013607 T01
Sobi.003G324400.2
Seit.5G348000.1
Brdi.2g52370.1
Orsa.01g58420.1
Anco.010545.1
Muac.8P06870 001
Muac.5P26850 001
Muac.3P23710 001
Muac.7P03640 001
Muac.8P33590 001
Muac.Un randomP16100 001
Pahal.F01347.1
Pahal.F01346.1
Seit.6G023800.1
Seit.6G023700.1
Sobi.007G077100.1
Sobi.007G077300.1
Zema.00008a037109 T01
Pahal.F01348.1
Brdi.5g21250.3
Orsa.04g52090.1
Zema.00008a006324 T01
Sobi.006G210300.1
Pahal.G02338.1
Seit.7G232100.1
Seit.9G252000.1
Brdi.3g47610.2
Anco.008323.1
Muac.8P15830 001
Muac.4P33270 001
Muac.6P00250 001
Muac.4P23620 001
Muac.7P00350 001
Sppo.14G0047400
Amtr.scaffold00115.2
Semo.443461
Semo.49950
1

S9e

ERF VIIIb-AOG1:
ERF084

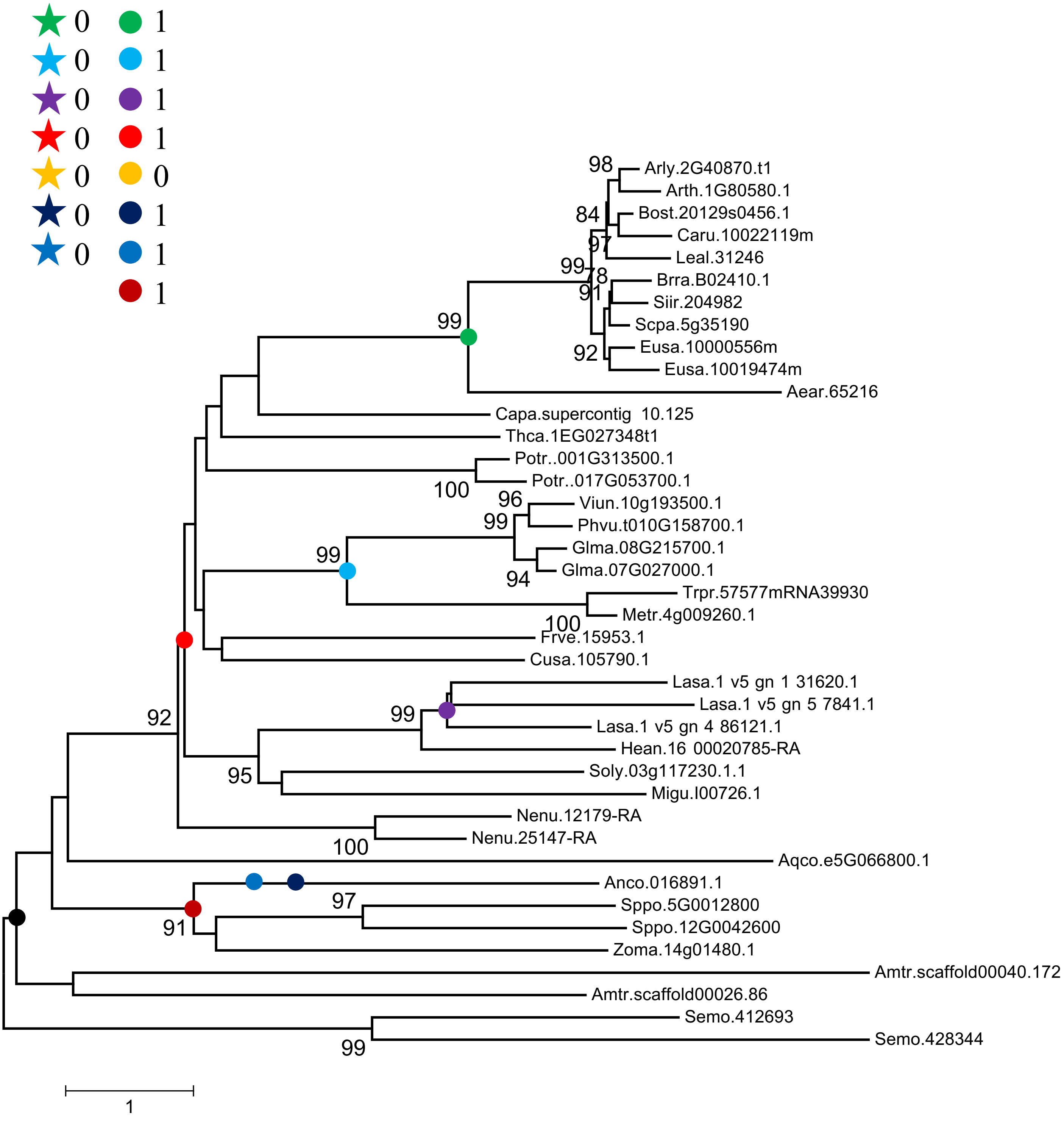

S9f

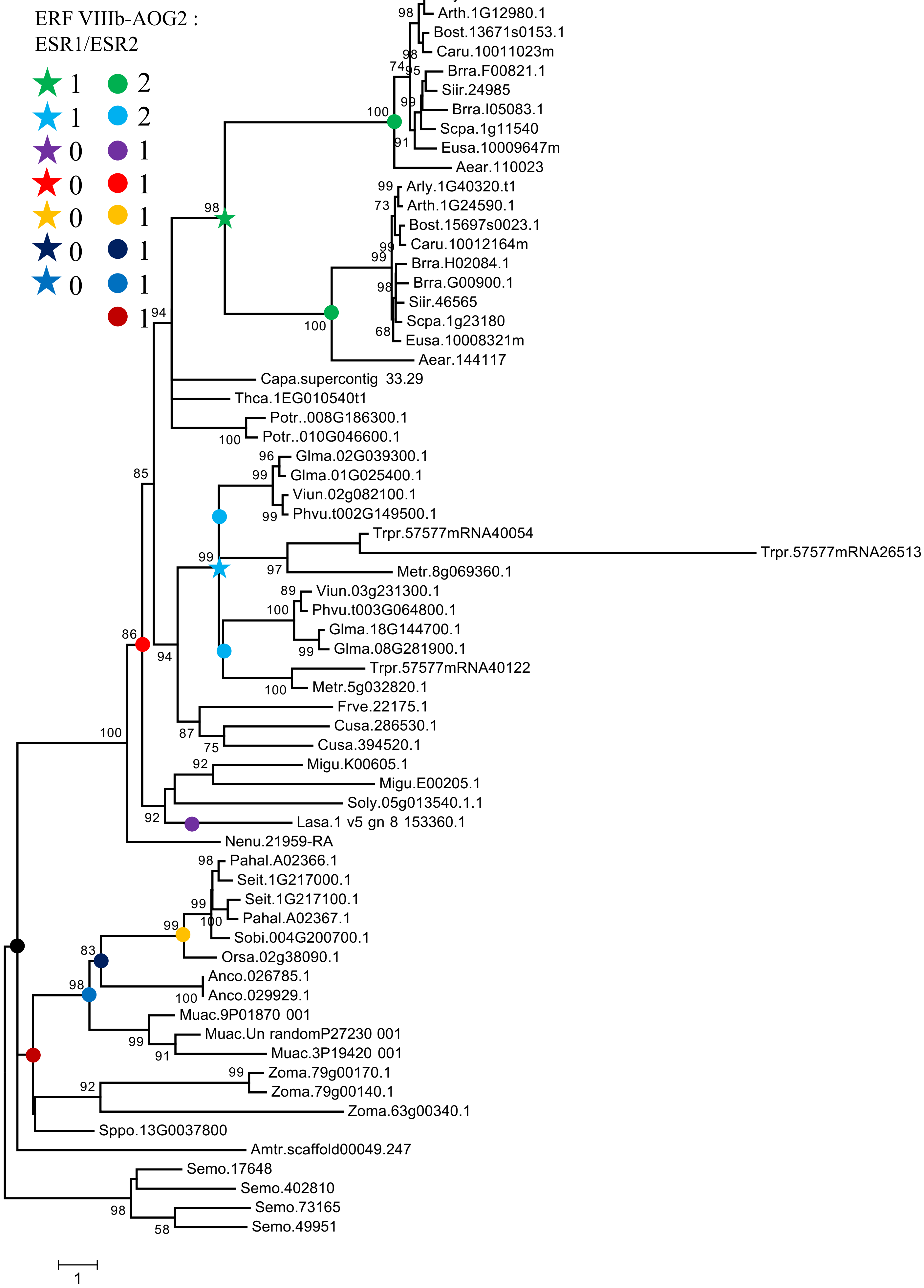

ERF VIIIb-AOG2 :
ESR1/ESR2
1 2
1 2
0 1
0 1
0 1
0 1
0 1
1
Arly.1G24630.t1
Arth.1G12980.1
Bost.13671s0153.1
Caru.10011023m
Brra.F00821.1
Siir.24985
Brra.I05083.1
Scpa.1g11540
Eusa.10009647m
Aear.110023
Arly.1G40320.t1
Arth.1G24590.1
Bost.15697s0023.1
Caru.10012164m
Brra.H02084.1
Brra.G00900.1
Siir.46565
Scpa.1g23180
Eusa.10008321m
Aear.144117
Capa.supercontig 33.29
Thca.1EG010540t1
Potr..008G186300.1
Potr..010G046600.1
Glma.02G039300.1
Glma.01G025400.1
Viun.02g082100.1
Phvu.t002G149500.1
Trpr.57577mRNA40054
Trpr.57577mRNA26513
Metr.8g069360.1
Viun.03g231300.1
Phvu.t003G064800.1
Glma.18G144700.1
Glma.08G281900.1
Trpr.57577mRNA40122
Metr.5g032820.1
Frve.22175.1
Cusa.286530.1
Cusa.394520.1
Migu.K00605.1
Migu.E00205.1
Soly.05g013540.1.1
Lasa.1 v5 gn 8 153360.1
Nenu.21959-RA
Pahal.A02366.1
Seit.1G217000.1
Seit.1G217100.1
Pahal.A02367.1
Sobi.004G200700.1
Orsa.02g38090.1
Anco.026785.1
Anco.029929.1
Muac.9P01870 001
Muac.Un randomP27230 001
Muac.3P19420 001
Zoma.79g00170.1
Zoma.79g00140.1
Zoma.63g00340.1
Sppo.13G0037800
Amtr.scaffold00049.247
Semo.17648
Semo.402810
Semo.73165
Semo.49951
1

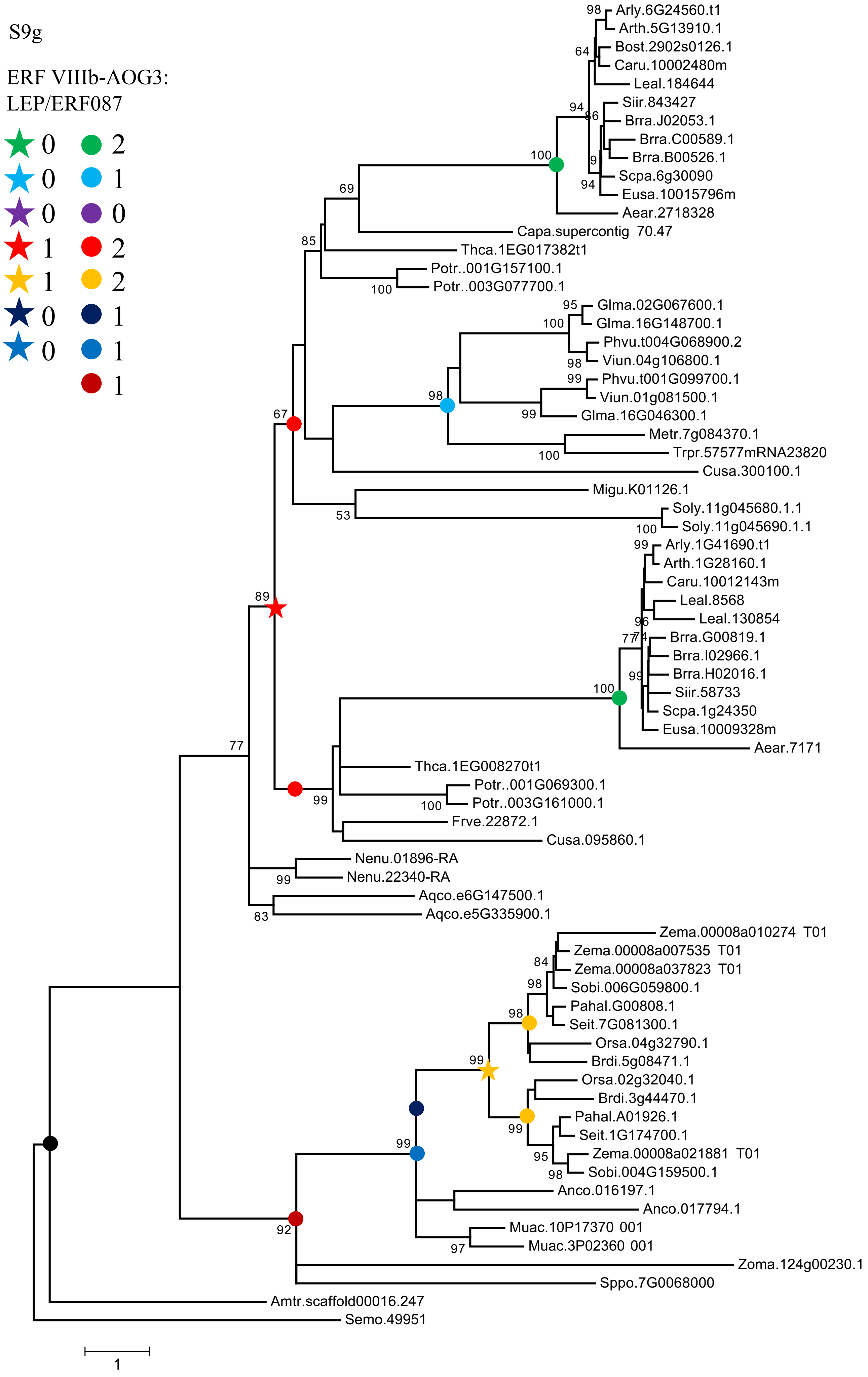
S9g
ERF VIIIb-AOG3:
LEP/ERF087
0 2
0 1
0 0
1 2
1 2
0 1
0 1
1
Arly.6G24560.t1
Arth.5G13910.1
Bost.2902s0126.1
Caru.10002480m
Leal.184644
Siir.843427
Brra.J02053.1
Brra.C00589.1
Brra.B00526.1
Scpa.6g30090
Eusa.10015796m
Aear.2718328
Capa.supercontig 70.47
Thca.1EG017382t1
Potr..001G157100.1
Potr..003G077700.1
Glma.02G067600.1
Glma.16G148700.1
Phvu.t004G068900.2
Viun.04g106800.1
Phvu.t001G099700.1
Viun.01g081500.1
Glma.16G046300.1
Metr.7g084370.1
Trpr.57577mRNA23820
Cusa.300100.1
Migu.K01126.1
Soly.11g045680.1.1
Soly.11g045690.1.1
Arly.1G41690.t1
Arth.1G28160.1
Caru.10012143m
Leal.8568
Leal.130854
Brra.G00819.1
Brra.I02966.1
Brra.H02016.1
Siir.58733
Scpa.1g24350
Eusa.10009328m
Aear.7171
Thca.1EG008270t1
Potr..001G069300.1
Potr..003G161000.1
Frve.22872.1
Cusa.095860.1
Nenu.01896-RA
Nenu.22340-RA
Aqco.e6G147500.1
Aqco.e5G335900.1
Zema.00008a010274 T01
Zema.00008a007535 T01
Zema.00008a037823 T01
Sobi.006G059800.1
Pahal.G00808.1
Seit.7G081300.1
Orsa.04g32790.1
Brdi.5g08471.1
Orsa.02g32040.1
Brdi.3g44470.1
Pahal.A01926.1
Seit.1G174700.1
Zema.00008a021881 T01
Sobi.004G159500.1
Anco.016197.1
Anco.017794.1
Muac.10P17370 001
Muac.3P02360 001
Zoma.124g00230.1
Sppo.7G0068000
Amtr.scaffold00016.247
Semo.49951
1

S9h

ERF VIIIb-AOG4:
ERF088

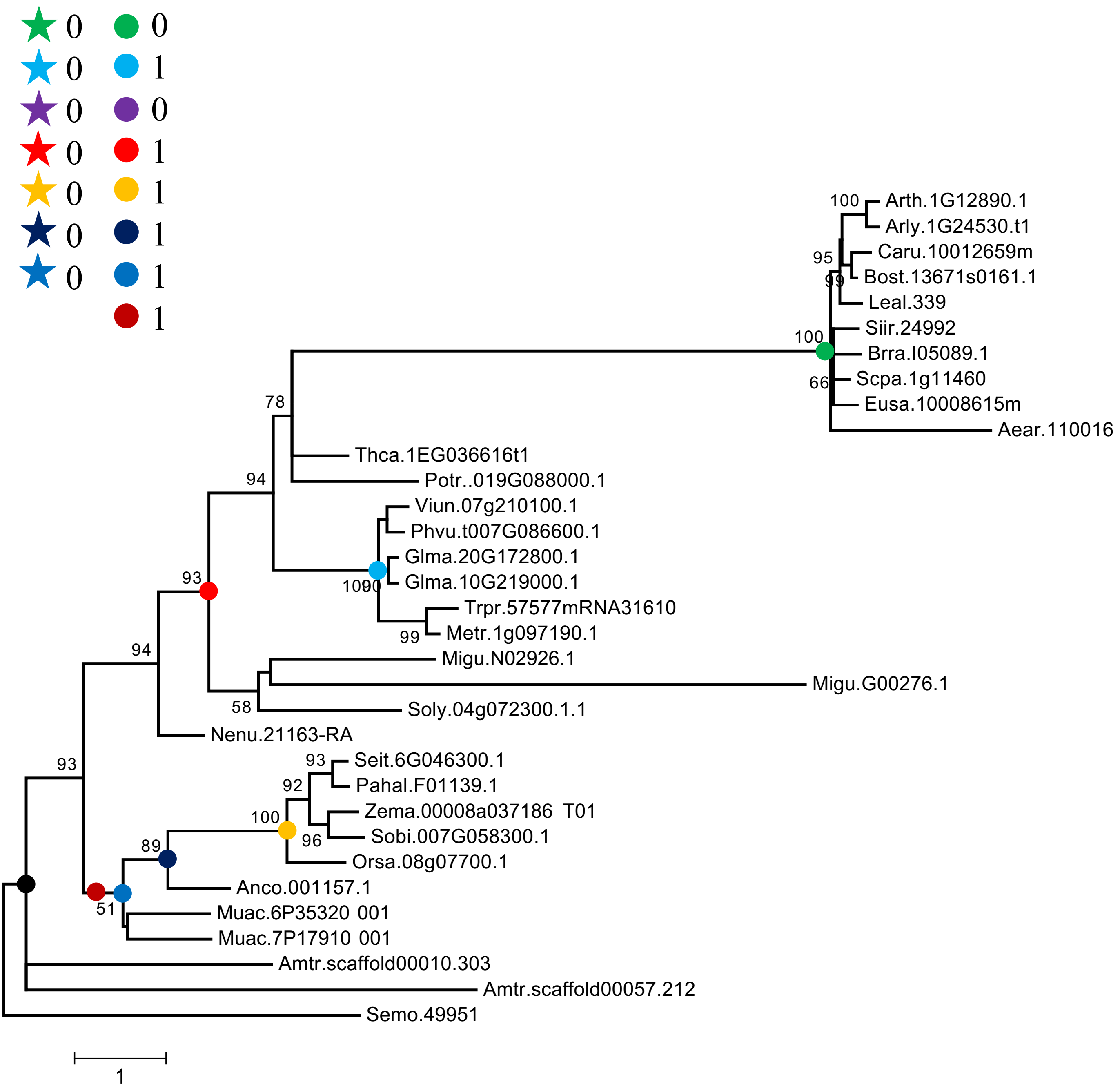

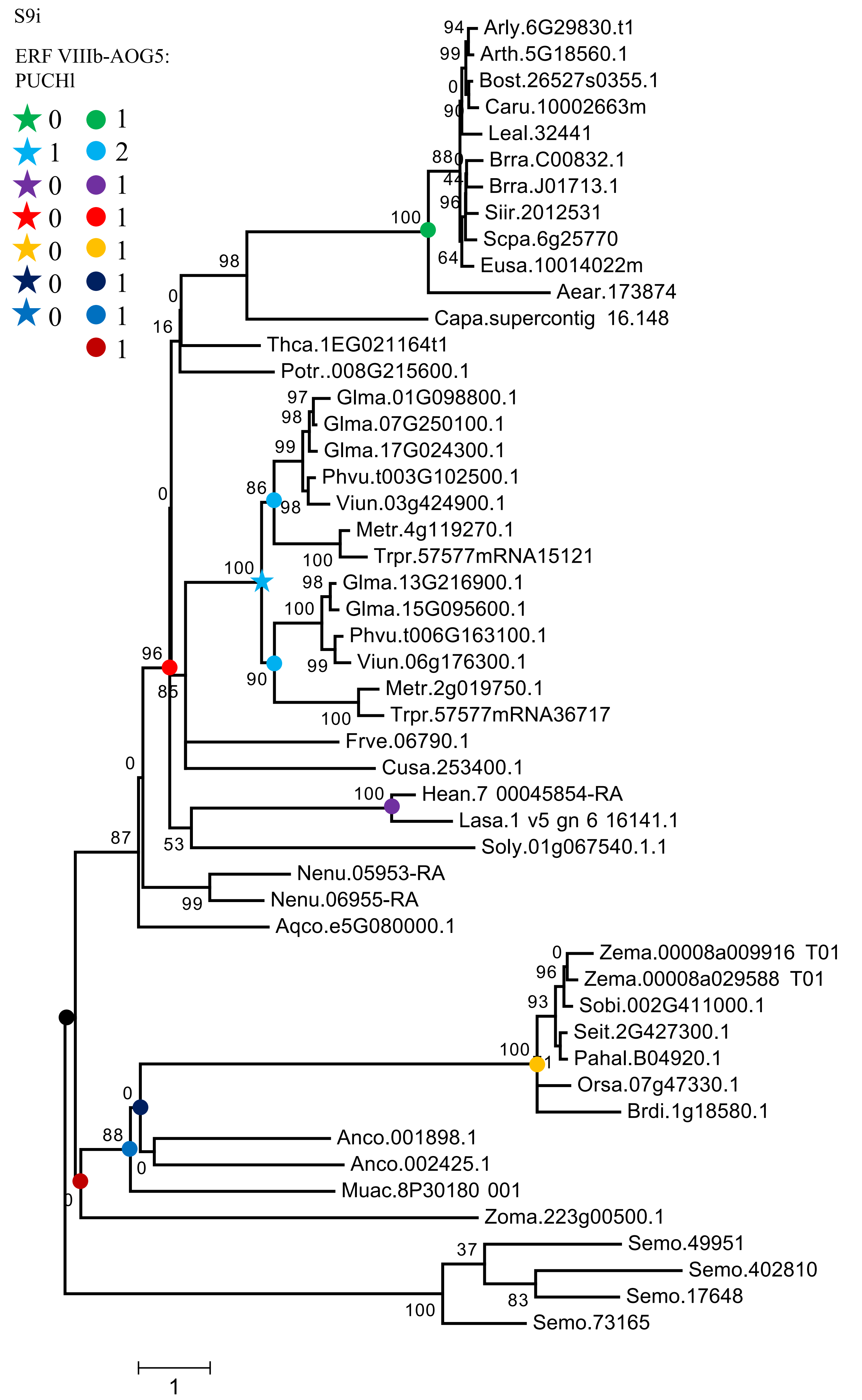

Fig. S9 Phylogenetic trees of ERF VIII subfamily from representative Angiosperms. SH-aLRT supports above 50% are labeled on internal nodes. The labeling is the same as in Fig. S1.

S10a

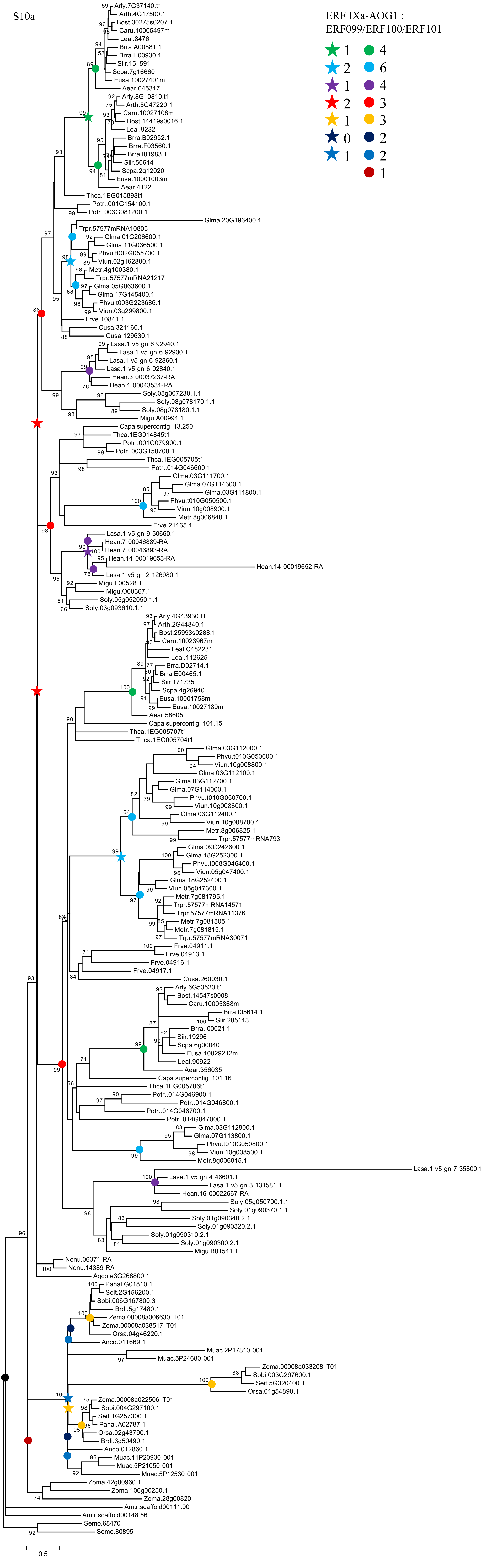

ERF IXa-AOG1 :
ERF099/ERF100/ERF101
1 4
2 6
1 4
2 3
1 3
0 2
1 2
1
Arly.7G37140.t1
Arth.4G17500.1
Bost.30275s0207.1
Caru.10005497m
Leal.8476
Brra.A00881.1
Brra.H00930.1
Siir.151591
Scpa.7g16660
Eusa.10027401m
Aear.645317
Arly.8G10810.t1
Arth.5G47220.1
Caru.10027108m
Bost.14419s0016.1
Leal.9232
Brra.B02952.1
Brra.F03560.1
Brra.I01983.1
Siir.50614
Scpa.2g12020
Eusa.10001003m
Aear.4122
Thca.1EG015898t1
Potr..001G154100.1
Potr..003G081200.1
Glma.20G196400.1
Trpr.57577mRNA10805
Glma.01G206600.1
Glma.11G036500.1
Phvu.t002G055700.1
Viun.02g162800.1
Metr.4g100380.1
Trpr.57577mRNA21217
Glma.05G063600.1
Glma.17G145400.1
Phvu.t003G223686.1
Viun.03g299800.1
Frve.10841.1
Cusa.321160.1
Cusa.129630.1
Lasa.1 v5 gn 6 92940.1
Lasa.1 v5 gn 6 92900.1
Lasa.1 v5 gn 6 92860.1
Lasa.1 v5 gn 6 92840.1
Hean.3 00037237-RA
Hean.1 00043531-RA
Soly.08g007230.1.1
Soly.08g078170.1.1
Soly.08g078180.1.1
Migu.A00994.1
Capa.supercontig 13.250
Thca.1EG014845t1
Potr..001G079900.1
Potr..003G150700.1
Thca.1EG005705t1
Potr..014G046600.1
Glma.03G111700.1
Glma.07G114300.1
Glma.03G111800.1
Phvu.t010G050500.1
Viun.10g008900.1
Metr.8g006840.1
Frve.21165.1
Lasa.1 v5 gn 9 50660.1
Hean.7 00046889-RA
Hean.7 00046893-RA
Hean.14 00019653-RA
Hean.14 00019652-RA
Lasa.1 v5 gn 2 126980.1
Migu.F00528.1
Migu.O00367.1
Soly.05g052050.1.1
Soly.03g093610.1.1
Arly.4G43930.t1
Arth.2G44840.1
Bost.25993s0288.1
Caru.10023967m
Leal.C482231
Leal.112625
Brra.D02714.1
Brra.E00465.1
Siir.171735
Scpa.4g26940
Eusa.10001758m
Eusa.10027189m
Aear.58605
Capa.supercontig 101.15
Thca.1EG005707t1
Thca.1EG005704t1
Glma.03G112000.1
Phvu.t010G050600.1
Viun.10g008800.1
Glma.03G112100.1
Glma.03G112700.1
Glma.07G114000.1
Phvu.t010G050700.1
Viun.10g008600.1
Glma.03G112400.1
Viun.10g008700.1
Metr.8g006825.1
Trpr.57577mRNA793
Glma.09G242600.1
Glma.18G252300.1
Phvu.t008G046400.1
Viun.05g047400.1
Glma.18G252400.1
Viun.05g047300.1
Metr.7g081795.1
Trpr.57577mRNA14571
Trpr.57577mRNA11376
Metr.7g081805.1
Metr.7g081815.1
Trpr.57577mRNA30071
Frve.04911.1
Frve.04913.1
Frve.04916.1
Frve.04917.1
Cusa.260030.1
Arly.6G53520.t1
Bost.14547s0008.1
Caru.10005868m
Brra.I05614.1
Siir.285113
Brra.I00021.1
Siir.19296
Scpa.6g00040
Eusa.10029212m
Leal.90922
Aear.356035
Capa.supercontig 101.16
Thca.1EG005706t1
Potr..014G046900.1
Potr..014G046800.1
Potr..014G046700.1
Potr..014G047000.1
Glma.03G112800.1
Glma.07G113800.1
Phvu.t010G050800.1
Viun.10g008500.1
Metr.8g006815.1
Lasa.1 v5 gn 7 35800.1
Lasa.1 v5 gn 4 46601.1
Lasa.1 v5 gn 3 131581.1
Hean.16 00022667-RA
Soly.05g050790.1.1
Soly.01g090370.1.1
Soly.01g090340.2.1
Soly.01g090320.2.1
Soly.01g090310.2.1
Soly.01g090300.2.1
Migu.B01541.1
Nenu.06371-RA
Nenu.14389-RA
Aqco.e3G268800.1
Pahal.G01810.1
Seit.2G156200.1
Sobi.006G167800.3
Brdi.5g17480.1
Zema.00008a006630 T01
Zema.00008a038517 T01
Orsa.04g46220.1
Anco.011669.1
Muac.2P17810 001
Muac.5P24680 001
Zema.00008a033208 T01
Sobi.003G297600.1
Seit.5G320400.1
Orsa.01g54890.1
Zema.00008a022506 T01
Sobi.004G297100.1
Seit.1G257300.1
Pahal.A02787.1
Orsa.02g43790.1
Brdi.3g50490.1
Anco.012860.1
Muac.11P20930 001
Muac.5P21050 001
Muac.5P12530 001
Zoma.42g00960.1
Zoma.106g00250.1
Zoma.28g00820.1
Amtr.scaffold00111.90
Amtr.scaffold00148.56
Semo.68470
Semo.80895
0.5

S10b

ERF IXb-AOG1:
ERF104/ERF105

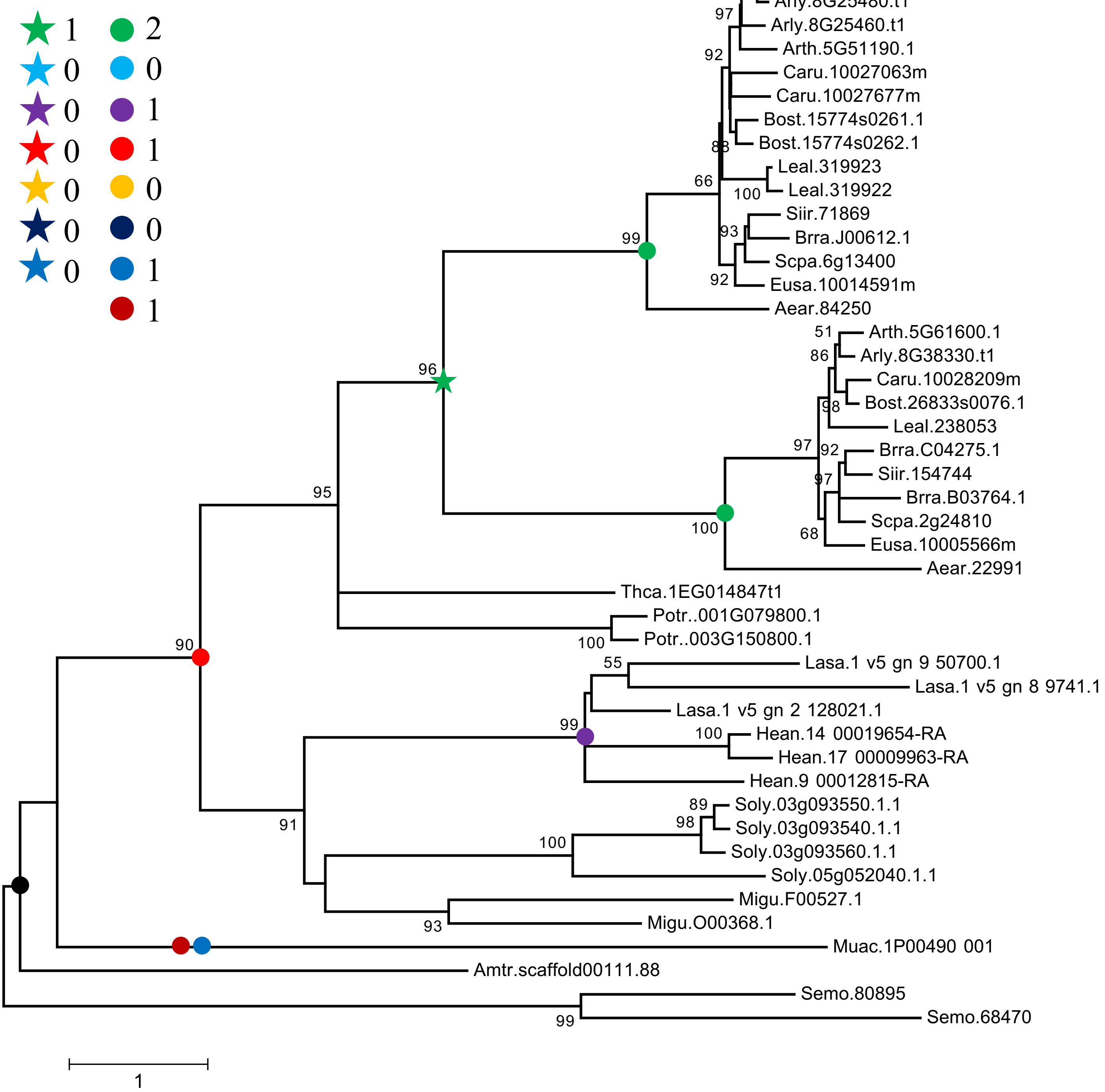

S10c

DREB IXb-AOG2:
ERF102/ERF103/ERF106/ERF107

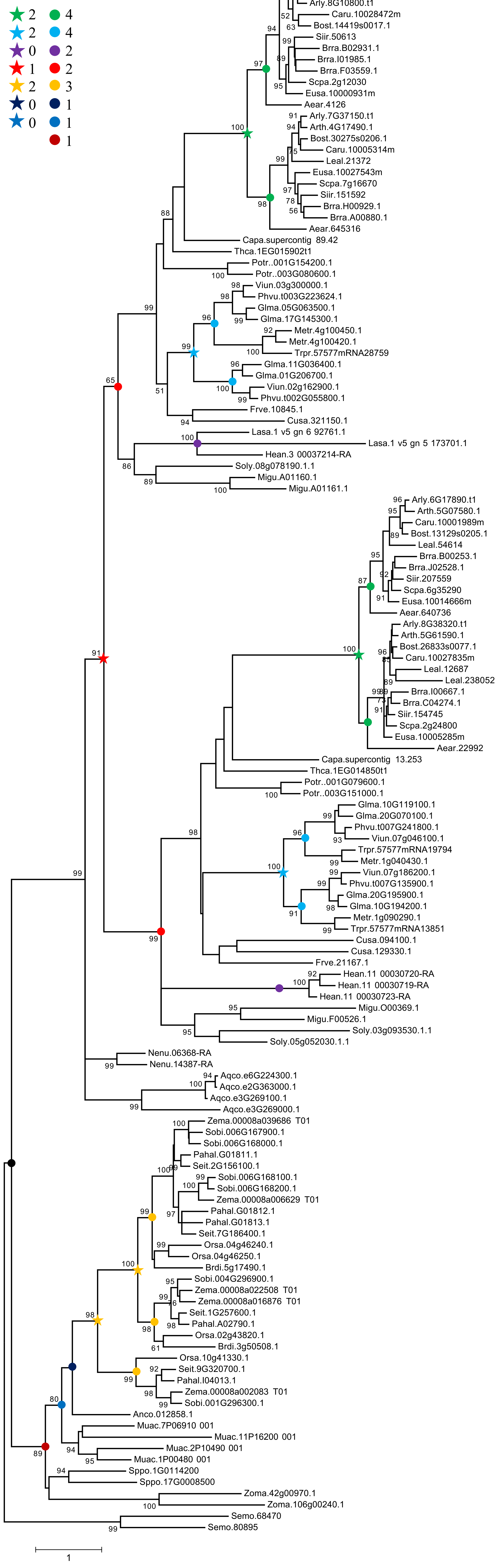

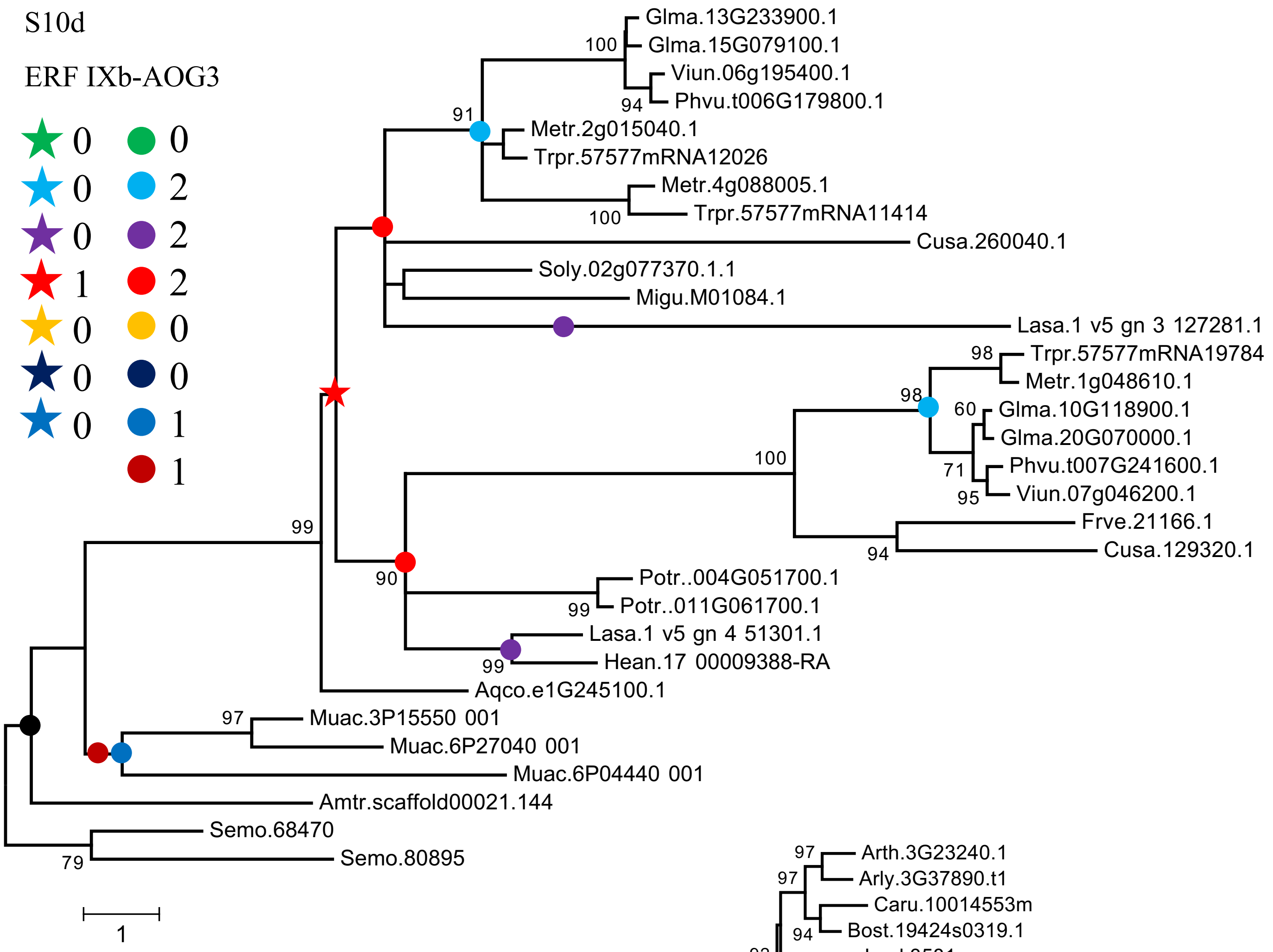
S10d
ERF IXb-AOG3
0
0
0
0
2
2
1
2
0
0
0
0
0
1
1
Glma.13G233900.1
Glma.15G079100.1
Viun.06g195400.1
Phvu.t006G179800.1
Metr.2g015040.1
Trpr.57577mRNA12026
Metr.4g088005.1
Trpr.57577mRNA11414
Cusa.260040.1
Soly.02g077370.1.1
Migu.M01084.1
Lasa.1 v5 gn 3 127281.1
Trpr.57577mRNA19784
Metr.1g048610.1
Glma.10G118900.1
Glma.20G070000.1
Phvu.t007G241600.1
Viun.07g046200.1
Frve.21166.1
Cusa.129320.1
Potr..004G051700.1
Potr..011G061700.1
Lasa.1 v5 gn 4 51301.1
Hean.17 00009388-RA
Aqco.e1G245100.1
Muac.3P15550 001
Muac.6P27040 001
Muac.6P04440 001
Amtr.scaffold00021.144
Semo.68470
Semo.80895
1

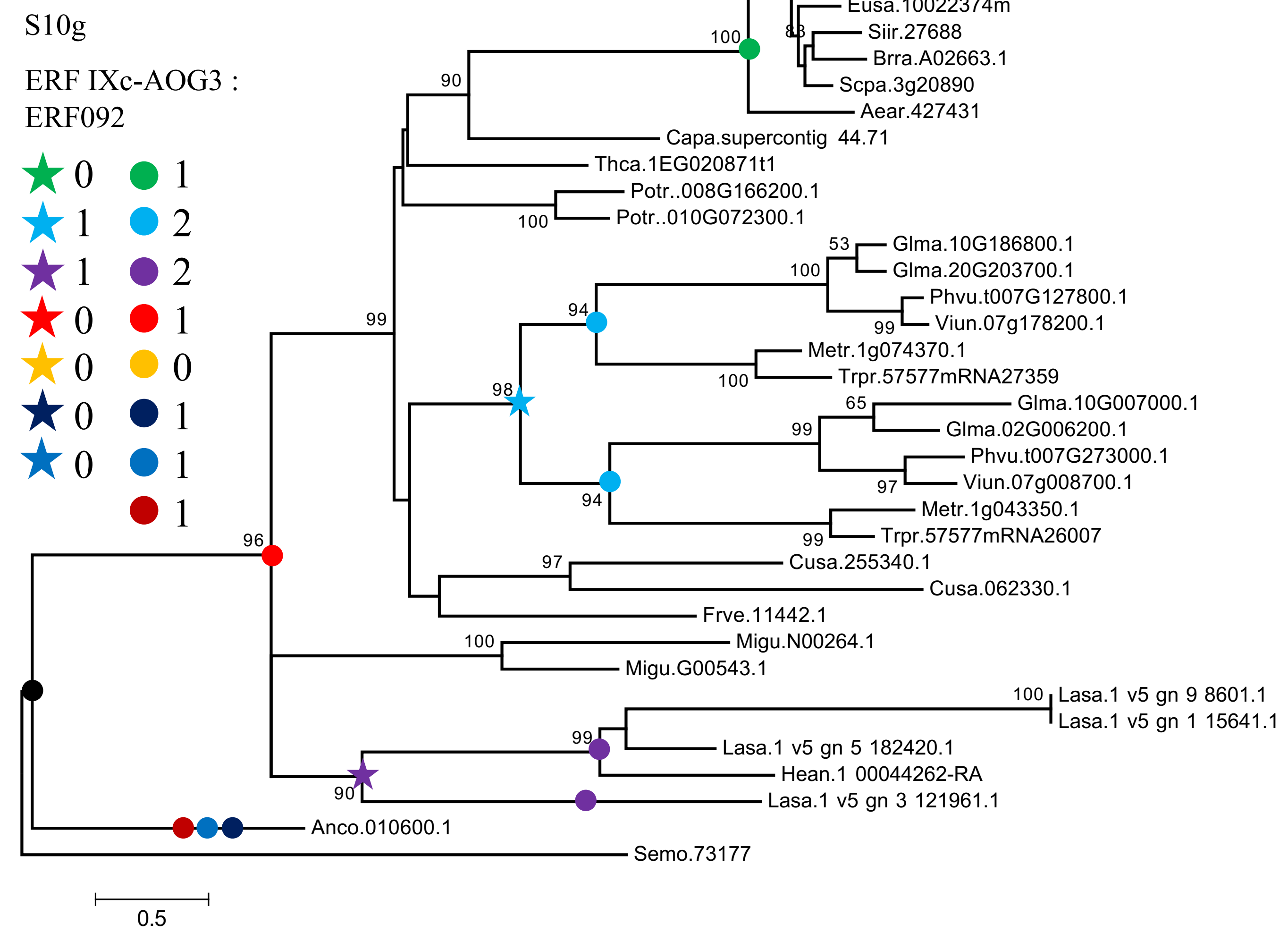
S10g
ERF IXc-AOG3 :
ERF092
0
1
1
2
1
2
0
1
0
0
0
1
0
1
1
Arth.3G23240.1
Arly.3G37890.t1
Caru.10014553m
Bost.19424s0319.1
Leal.3581
Eusa.10022374m
Siir.27688
Brra.A02663.1
Scpa.3g20890
Aear.427431
Capa.supercontig 44.71
Thca.1EG020871t1
Potr..008G166200.1
Potr..010G072300.1
Glma.10G186800.1
Glma.20G203700.1
Phvu.t007G127800.1
Viun.07g178200.1
Metr.1g074370.1
Trpr.57577mRNA27359
Glma.10G007000.1
Glma.02G006200.1
Phvu.t007G273000.1
Viun.07g008700.1
Metr.1g043350.1
Trpr.57577mRNA26007
Cusa.255340.1
Cusa.062330.1
Frve.11442.1
Migu.N00264.1
Migu.G00543.1
Lasa.1 v5 gn 9 8601.1
Lasa.1 v5 gn 1 15641.1
Lasa.1 v5 gn 5 182420.1
Hean.1 00044262-RA
Lasa.1 v5 gn 3 121961.1
Anco.010600.1
Semo.73177
0.5

S10e

ERF IXc-AOG1:
ERF091

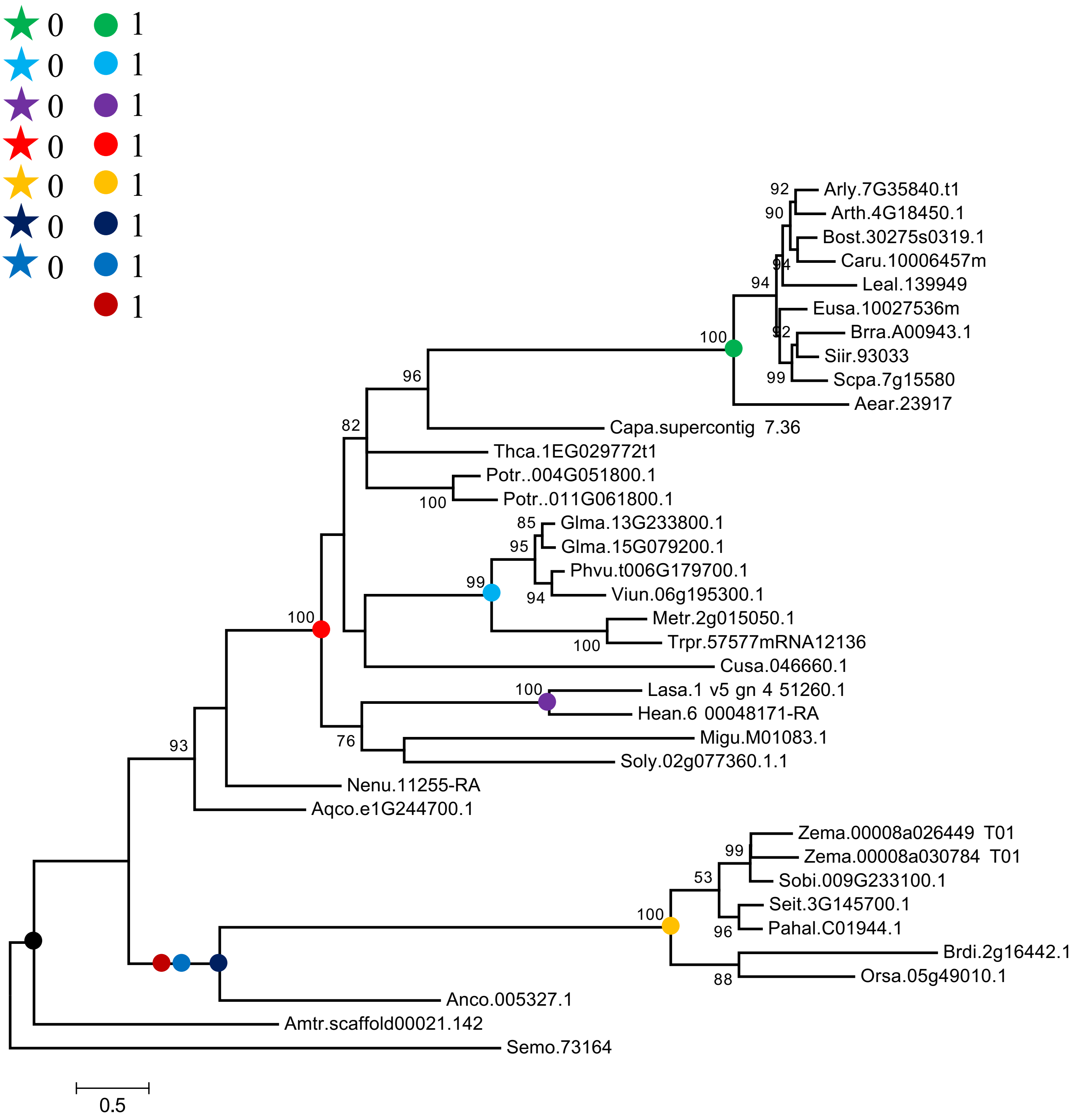

S10f

ERF IXc-AOG2:
ERF095/ERF096/ERF097/ERF098

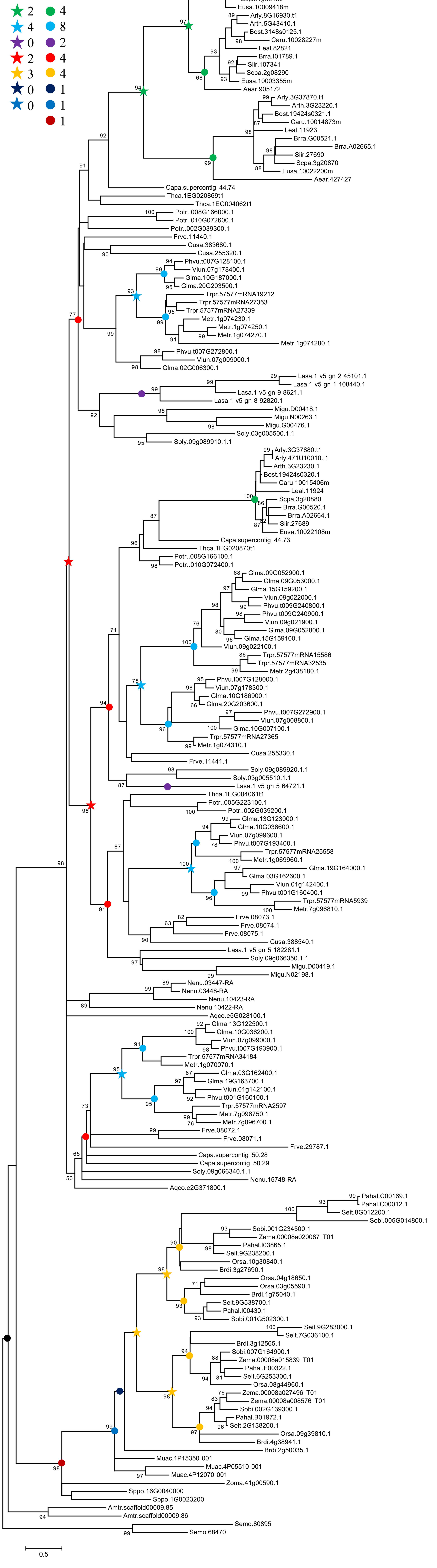

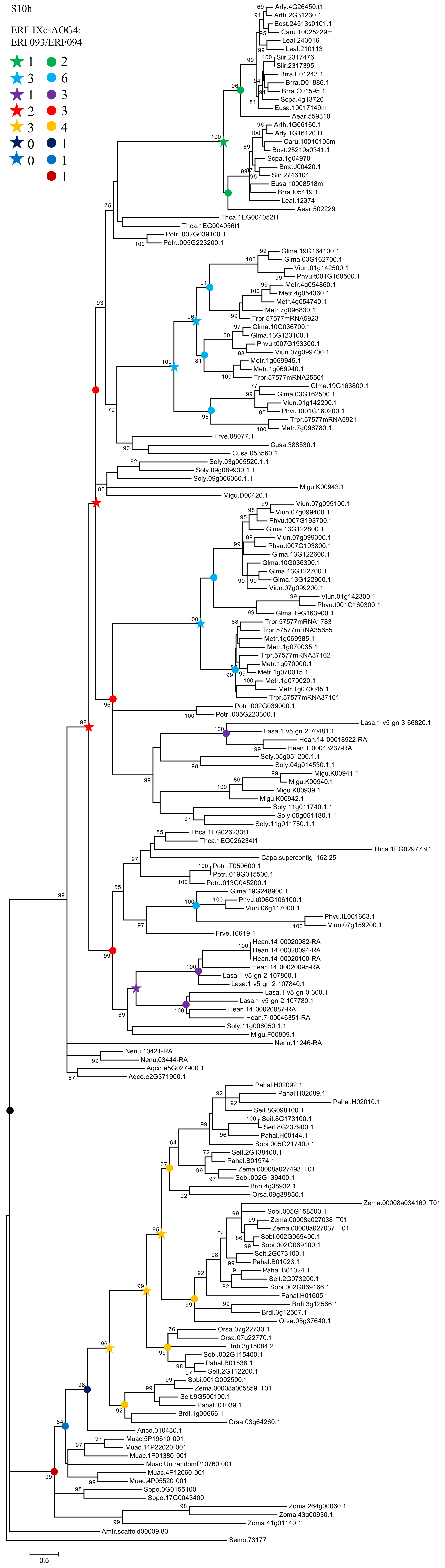

Fig. S10 Phylogenetic trees of ERF IX subfamily from representative Angiosperms. SH-aLRT supports above 50% are labeled on internal nodes. The labeling is the same as in Fig. S1.

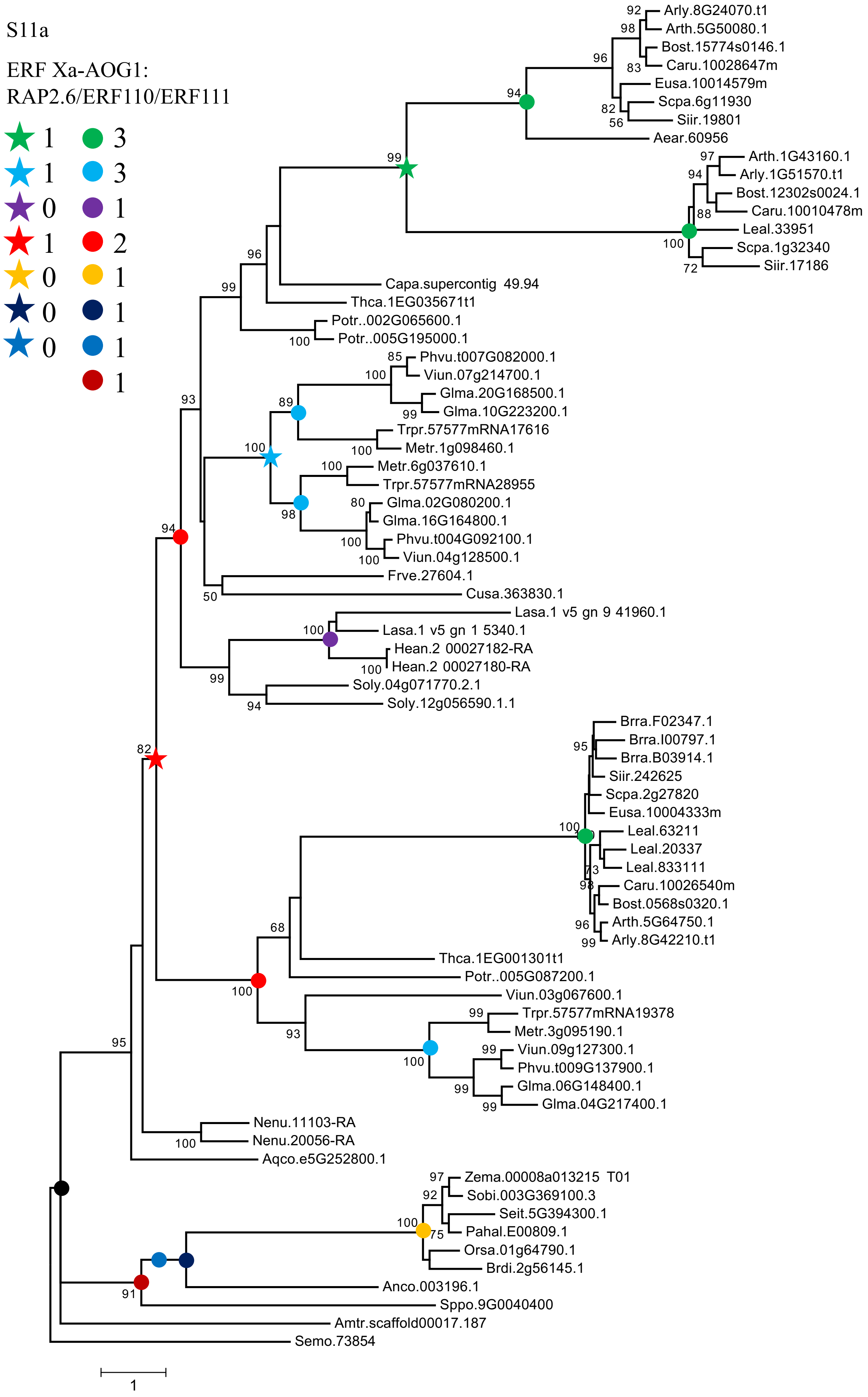

S11a
ERF Xa-AOG1:
RAP2.6/ERF110/ERF111
1 3
1 3
0 1
1 2
0 1
0 1
0 1
1
Arly.8G24070.t1
Arth.5G50080.1
Bost.15774s0146.1
Caru.10028647m
Eusa.10014579m
Scpa.6g11930
Siir.19801
Aear.60956
Arth.1G43160.1
Arly.1G51570.t1
Bost.12302s0024.1
Caru.10010478m
Leal.33951
Scpa.1g32340
Siir.17186
Capa.supercontig 49.94
Thca.1EG035671t1
Potr..002G065600.1
Potr..005G195000.1
Phvu.t007G082000.1
Viun.07g214700.1
Glma.20G168500.1
Glma.10G223200.1
Trpr.57577mRNA17616
Metr.1g098460.1
Metr.6g037610.1
Trpr.57577mRNA28955
Glma.02G080200.1
Glma.16G164800.1
Phvu.t004G092100.1
Viun.04g128500.1
Frve.27604.1
Cusa.363830.1
Lasa.1 v5 gn 9 41960.1
Lasa.1 v5 gn 1 5340.1
Hean.2 00027182-RA
Hean.2 00027180-RA
Soly.04g071770.2.1
Soly.12g056590.1.1
Brra.F02347.1
Brra.I00797.1
Brra.B03914.1
Siir.242625
Scpa.2g27820
Eusa.10004333m
Leal.63211
Leal.20337
Leal.833111
Caru.10026540m
Bost.0568s0320.1
Arth.5G64750.1
Arly.8G42210.t1
Thca.1EG001301t1
Potr..005G087200.1
Viun.03g067600.1
Trpr.57577mRNA19378
Metr.3g095190.1
Viun.09g127300.1
Phvu.t009G137900.1
Glma.06G148400.1
Glma.04G217400.1
Nenu.11103-RA
Nenu.20056-RA
Aqco.e5G252800.1
Zema.00008a013215 T01
Sobi.003G369100.3
Seit.5G394300.1
Pahal.E00809.1
Orsa.01g64790.1
Brdi.2g56145.1
Anco.003196.1
Sppo.9G0040400
Amtr.scaffold00017.187
Semo.73854
1

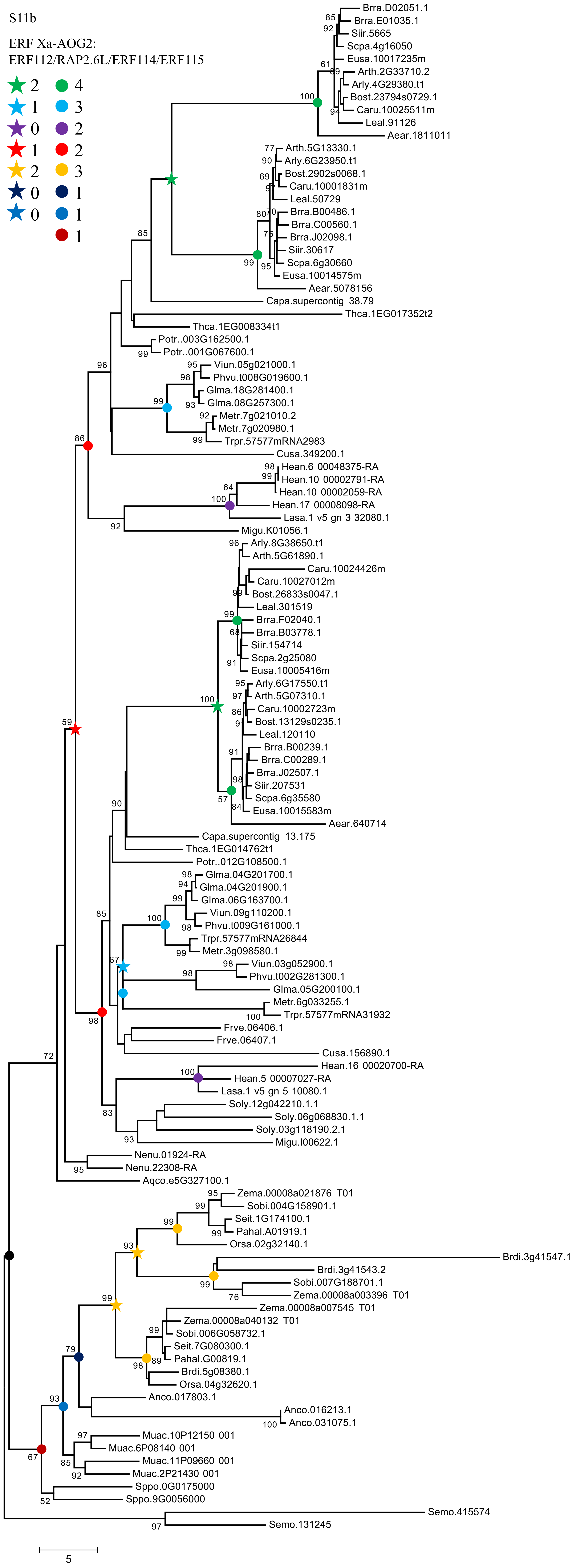

S11b
ERF Xa-AOG2:
ERF112/RAP2.6L/ERF114/ERF115
2 4
1 3
0 2
1 2
2 3
0 1
0 1
1
Brra.D02051.1
Brra.E01035.1
Siir.5665
Scpa.4g16050
Eusa.10017235m
Arth.2G33710.2
Arly.4G29380.t1
Bost.23794s0729.1
Caru.10025511m
Leal.91126
Aear.1811011
Arth.5G13330.1
Arly.6G23950.t1
Bost.2902s0068.1
Caru.10001831m
Leal.50729
Brra.B00486.1
Brra.C00560.1
Brra.J02098.1
Siir.30617
Scpa.6g30660
Eusa.10014575m
Aear.5078156
Capa.supercontig 38.79
Thca.1EG017352t2
Thca.1EG008334t1
Potr..003G162500.1
Potr..001G067600.1
Viun.05g021000.1
Phvu.t008G019600.1
Glma.18G281400.1
Glma.08G257300.1
Metr.7g021010.2
Metr.7g020980.1
Trpr.57577mRNA2983
Cusa.349200.1
Hean.6 00048375-RA
Hean.10 00002791-RA
Hean.10 00002059-RA
Hean.17 00008098-RA
Lasa.1 v5 gn 3 32080.1
Migu.K01056.1
Arly.8G38650.t1
Arth.5G61890.1
Caru.10024426m
Caru.10027012m
Bost.26833s0047.1
Leal.301519
Brra.F02040.1
Brra.B03778.1
Siir.154714
Scpa.2g25080
Eusa.10005416m
Arly.6G17550.t1
Arth.5G07310.1
Caru.10002723m
Bost.13129s0235.1
Leal.120110
Brra.B00239.1
Brra.C00289.1
Brra.J02507.1
Siir.207531
Scpa.6g35580
Eusa.10015583m
Aear.640714
Capa.supercontig 13.175
Thca.1EG014762t1
Potr..012G108500.1
Glma.04G201700.1
Glma.04G201900.1
Glma.06G163700.1
Viun.09g110200.1
Phvu.t009G161000.1
Trpr.57577mRNA26844
Metr.3g098580.1
Viun.03g052900.1
Phvu.t002G281300.1
Glma.05G200100.1
Metr.6g033255.1
Trpr.57577mRNA31932
Frve.06406.1
Frve.06407.1
Cusa.156890.1
Hean.16 00020700-RA
Hean.5 00007027-RA
Lasa.1 v5 gn 5 10080.1
Soly.12g042210.1.1
Soly.06g068830.1.1
Soly.03g118190.2.1
Migu.I00622.1
Nenu.01924-RA
Nenu.22308-RA
Aqco.e5G327100.1
Zema.00008a021876 T01
Sobi.004G158901.1
Seit.1G174100.1
Pahal.A01919.1
Orsa.02g32140.1
Brdi.3g41547.1
Brdi.3g41543.2
Sobi.007G188701.1
Zema.00008a003396 T01
Zema.00008a007545 T01
Zema.00008a040132 T01
Sobi.006G058732.1
Seit.7G080300.1
Pahal.G00819.1
Brdi.5g08380.1
Orsa.04g32620.1
Anco.017803.1
Anco.016213.1
Anco.031075.1
Muac.10P12150 001
Muac.6P08140 001
Muac.11P09660 001
Muac.2P21430 001
Sppo.0G0175000
Sppo.9G0056000
Semo.415574
Semo.131245
5

S11c

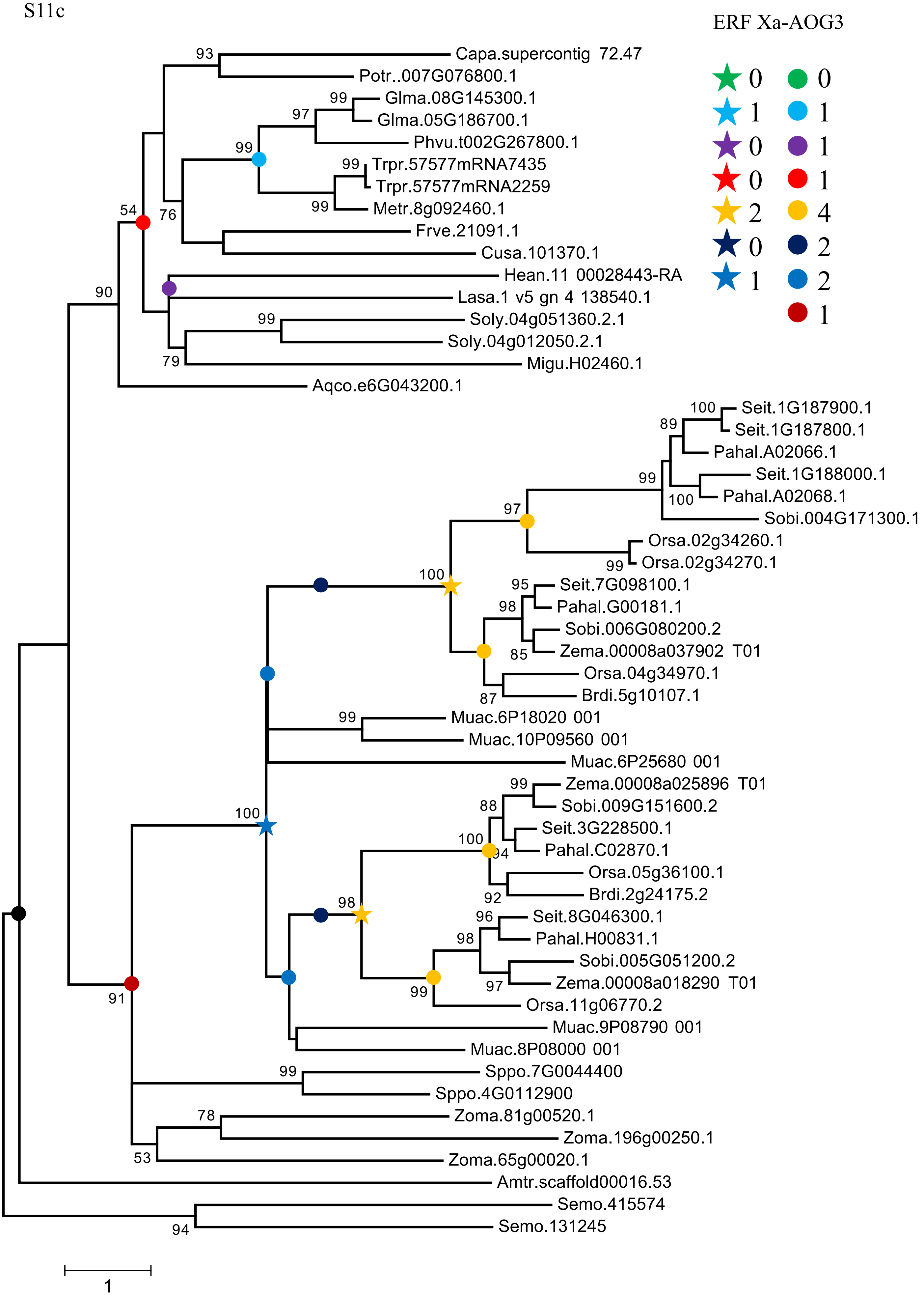
ERF Xa-AOG3
Capa.supercontig 72.47
Potr..007G076800.1
Glma.08G145300.1
Glma.05G186700.1
Phvu.t002G267800.1
Trpr.57577mRNA7435
Trpr.57577mRNA2259
Metr.8g092460.1
Frve.21091.1
Cusa.101370.1
Hean.11 00028443-RA
Lasa.1 v5 gn 4 138540.1
Soly.04g051360.2.1
Soly.04g012050.2.1
Migu.H02460.1
Aqco.e6G043200.1
Seit.1G187900.1
Seit.1G187800.1
Pahal.A02066.1
Seit.1G188000.1
Pahal.A02068.1
Sobi.004G171300.1
Orsa.02g34260.1
Orsa.02g34270.1
Seit.7G098100.1
Pahal.G00181.1
Sobi.006G080200.2
Zema.00008a037902 T01
Orsa.04g34970.1
Brdi.5g10107.1
Muac.6P18020 001
Muac.10P09560 001
Muac.6P25680 001
Zema.00008a025896 T01
Sobi.009G151600.2
Seit.3G228500.1
Pahal.C02870.1
Orsa.05g36100.1
Brdi.2g24175.2
Seit.8G046300.1
Pahal.H00831.1
Sobi.005G051200.2
Zema.00008a018290 T01
Orsa.11g06770.2
Muac.9P08790 001
Muac.8P08000 001
Sppo.7G0044400
Sppo.4G0112900
Zoma.81g00520.1
Zoma.196g00250.1
Zoma.65g00020.1
Amtr.scaffold00016.53
Semo.415574
Semo.131245
1

S11d

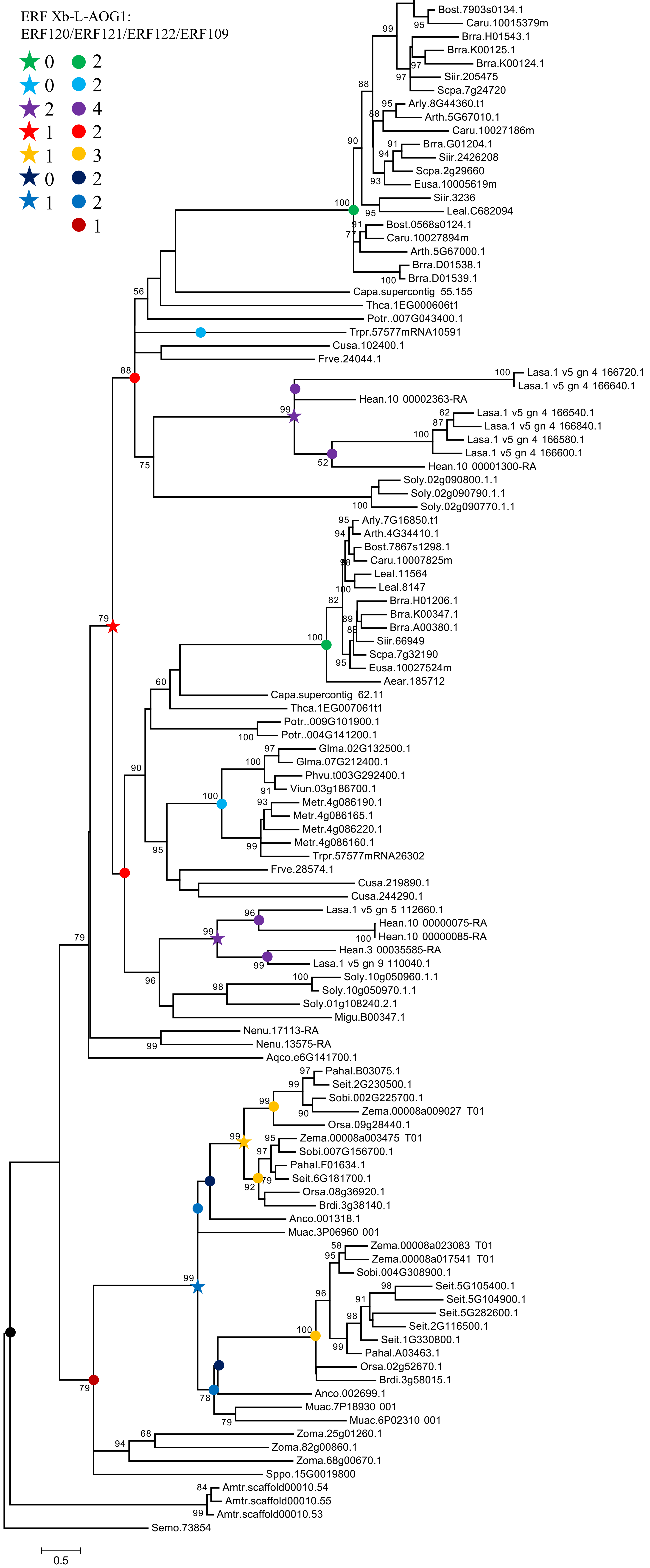

Fig. S11 Phylogenetic trees of ERF X subfamily from representative Angiosperms. SH-aLRT supports above 50% are labeled on internal nodes. The labeling is the same as in Fig. S1.

S12a

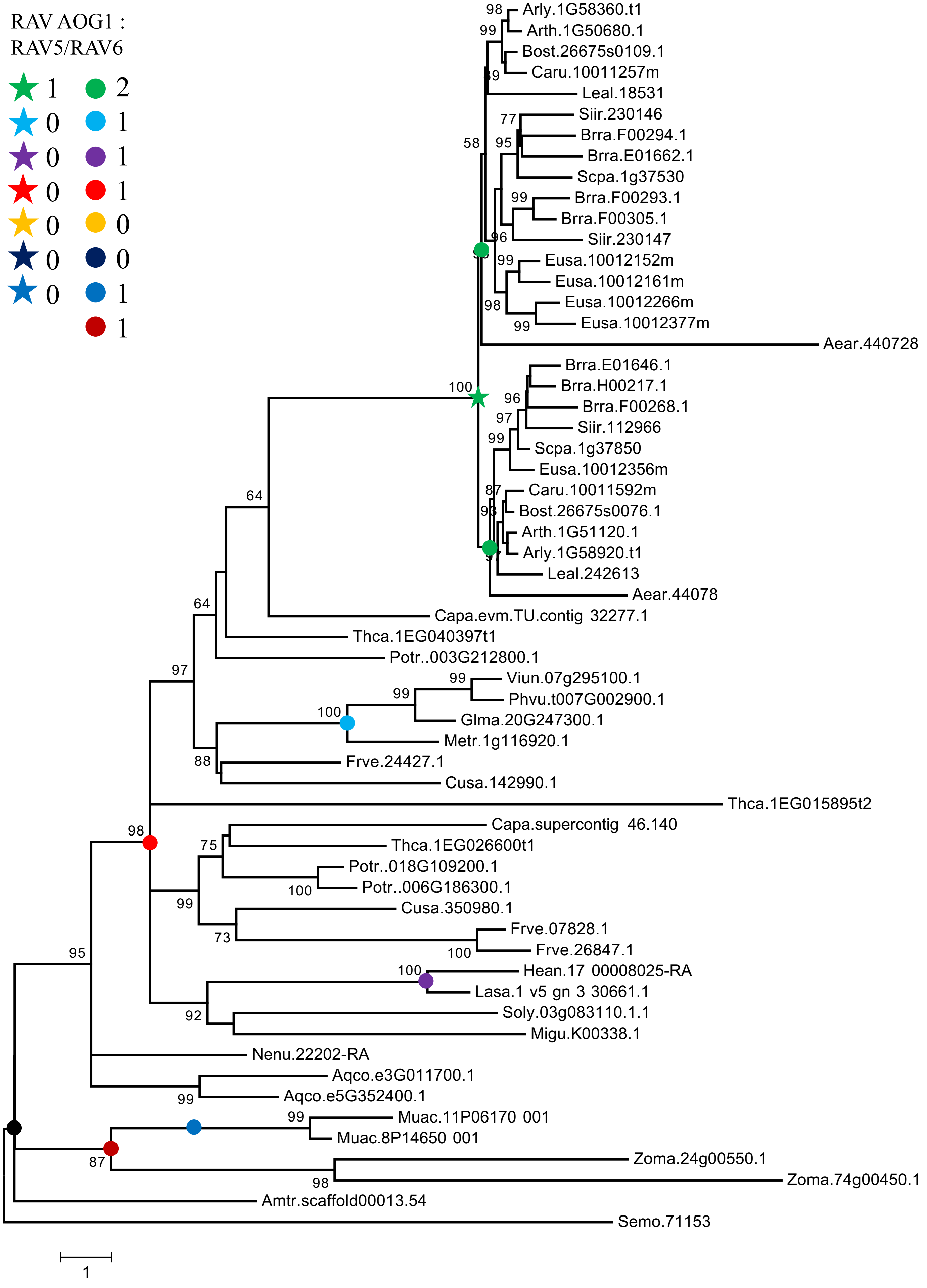

RAV AOG1 :
RAV5/RAV6
1
2
0
1
0
1
0
1
0
0
0
0
0
1
1
Arly.1G58360.t1
Arth.1G50680.1
Bost.26675s0109.1
Caru.10011257m
Leal.18531
Siir.230146
Brra.F00294.1
Brra.E01662.1
Scpa.1g37530
Brra.F00293.1
Brra.F00305.1
Siir.230147
Eusa.10012152m
Eusa.10012161m
Eusa.10012266m
Eusa.10012377m
Aear.440728
Brra.E01646.1
Brra.H00217.1
Brra.F00268.1
Siir.112966
Scpa.1g37850
Eusa.10012356m
Caru.10011592m
Bost.26675s0076.1
Arth.1G51120.1
Arly.1G58920.t1
Leal.242613
Aear.44078
Capa.evm.TU.contig 32277.1
Thca.1EG040397t1
Potr..003G212800.1
Viun.07g295100.1
Phvu.t007G002900.1
Glma.20G247300.1
Metr.1g116920.1
Frve.24427.1
Cusa.142990.1
Thca.1EG015895t2
Capa.supercontig 46.140
Thca.1EG026600t1
Potr..018G109200.1
Potr..006G186300.1
Cusa.350980.1
Frve.07828.1
Frve.26847.1
Hean.17 00008025-RA
Lasa.1 v5 gn 3 30661.1
Soly.03g083110.1.1
Migu.K00338.1
Nenu.22202-RA
Aqco.e3G011700.1
Aqco.e5G352400.1
Muac.11P06170 001
Muac.8P14650 001
Zoma.24g00550.1
Zoma.74g00450.1
Amtr.scaffold00013.54
Semo.71153
98
99
58
77
95
99
99
98
99
100
96
97
99
87
64
64
97
99
99
100
88
98
75
100
99
73
100
100
92
95
99
99
87
98
1

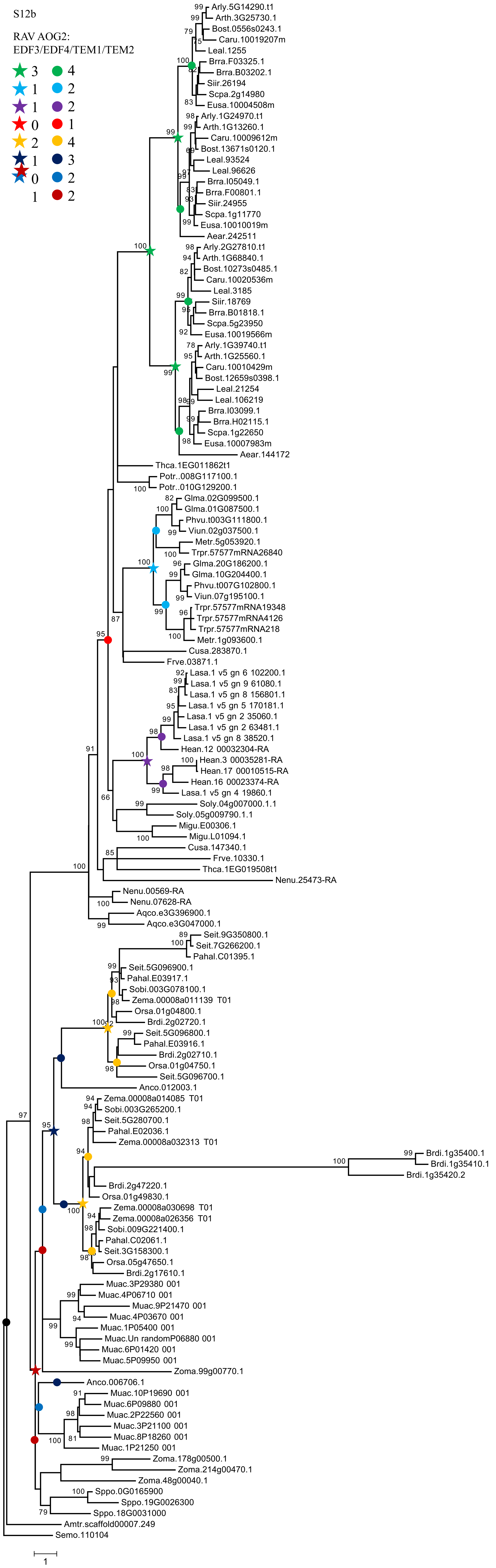

Fig. S12 Phylogenetic trees of RAV subfamily from representative Angiosperms. SH-aLRT supports above 50% are labeled on internal nodes. The labeling is the same as in Fig. S1.

S13

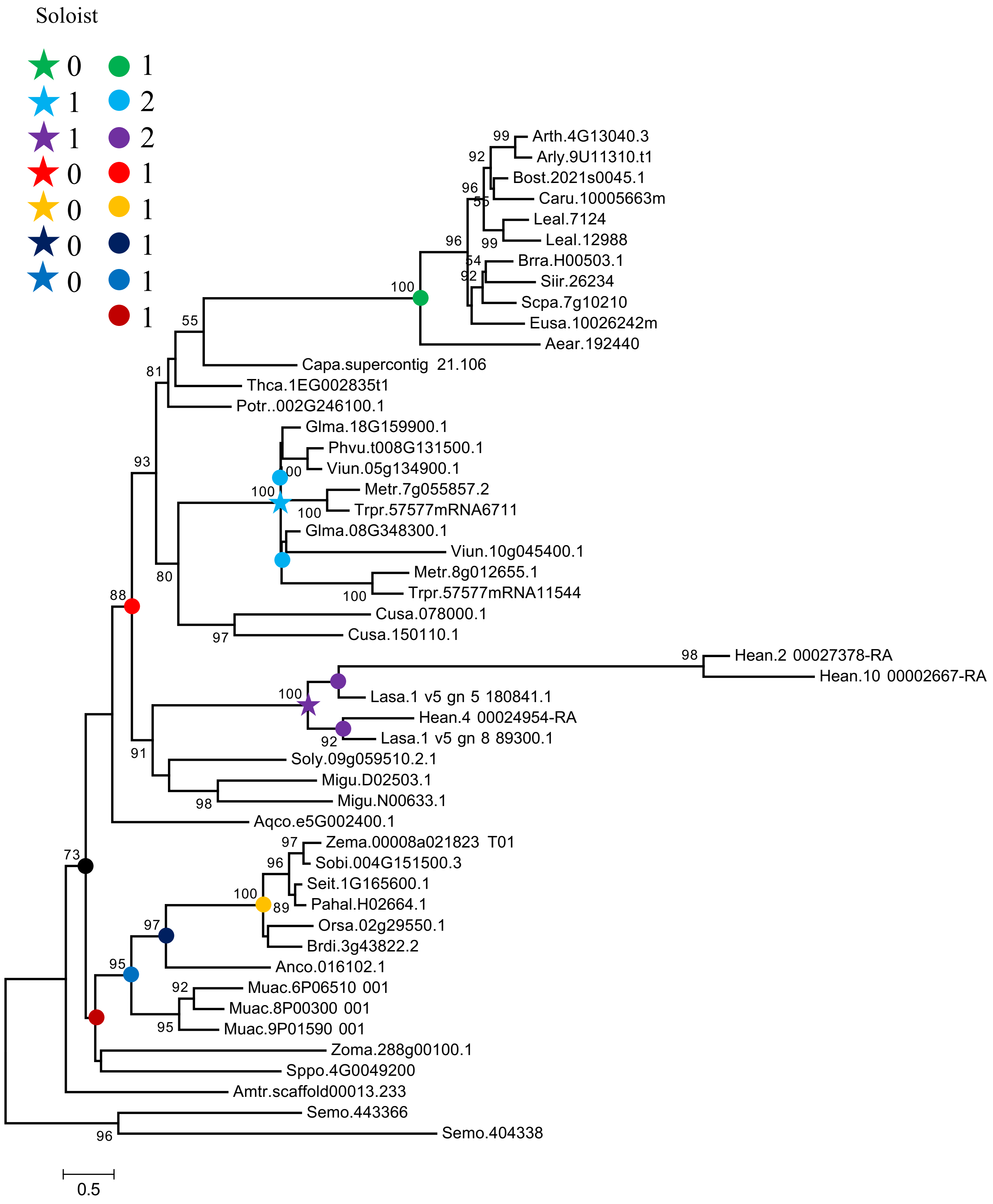

Fig. S13 A phylogenetic tree of Soloist from representative Angiosperms. SH-aLRT supports above 50% are labeled on internal nodes. The labeling is the same as in Fig. S1.

Figure S14. Wang et al.

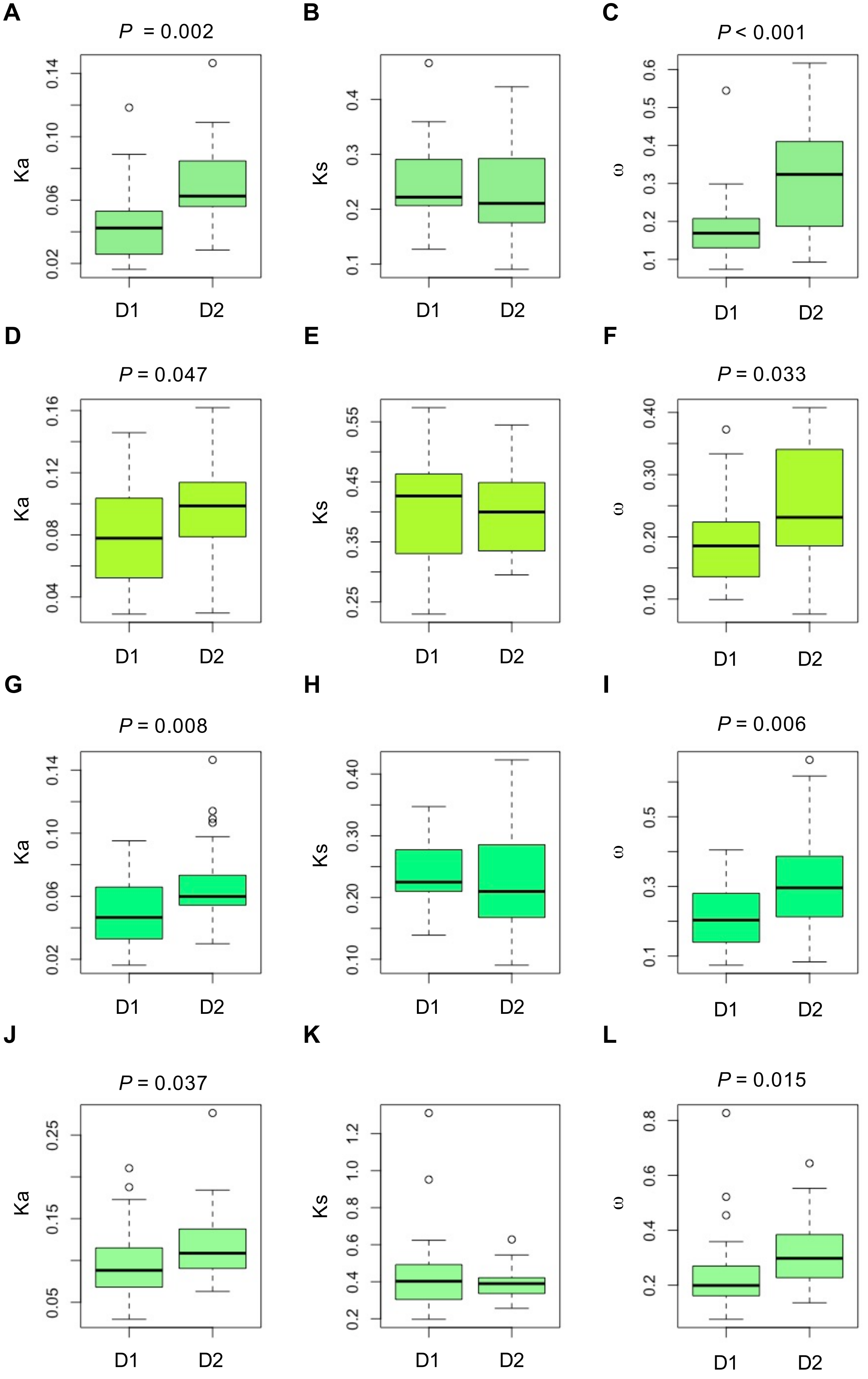

Figure S15. Wang et al.

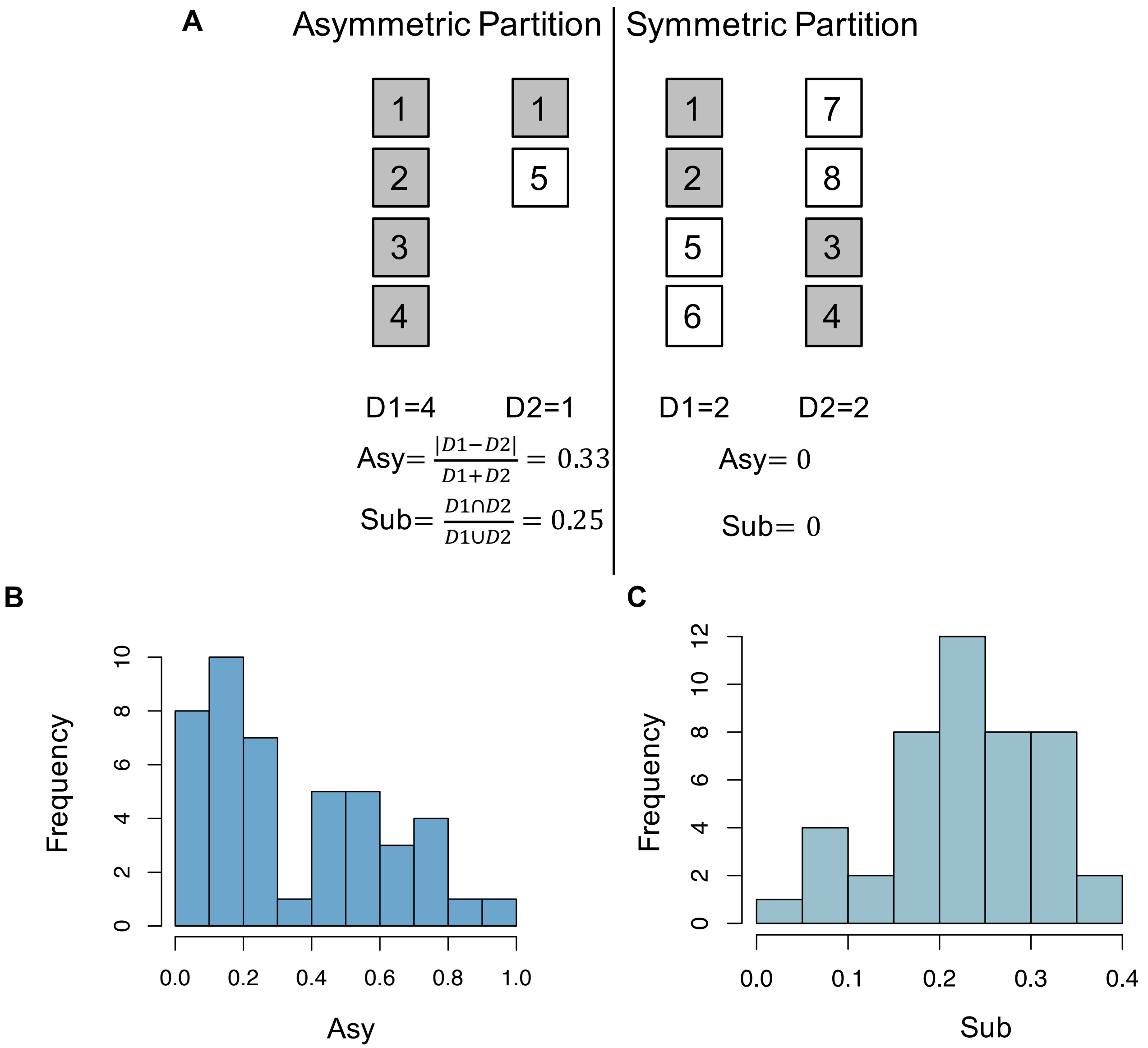

Figure S16. Wang et al.

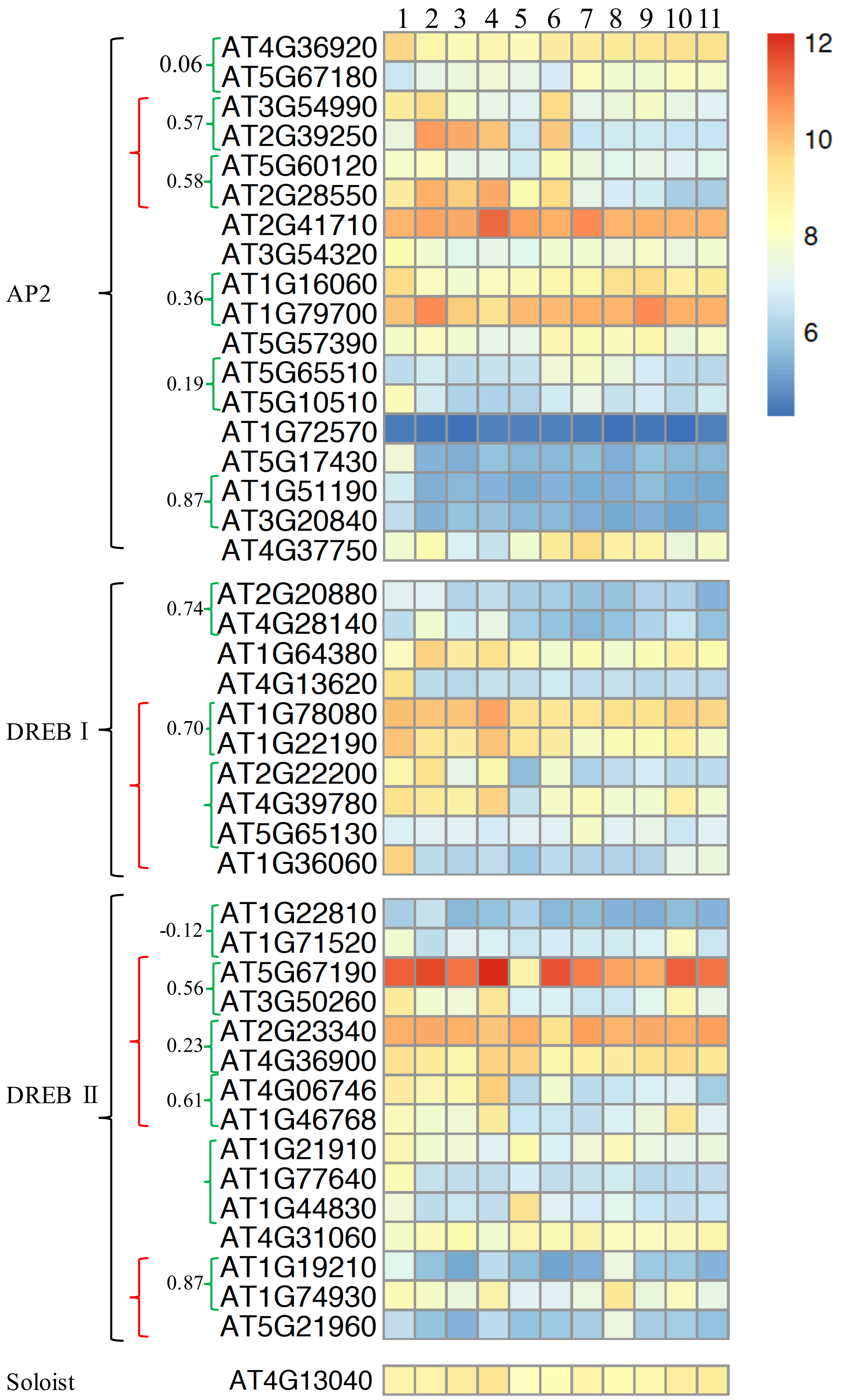

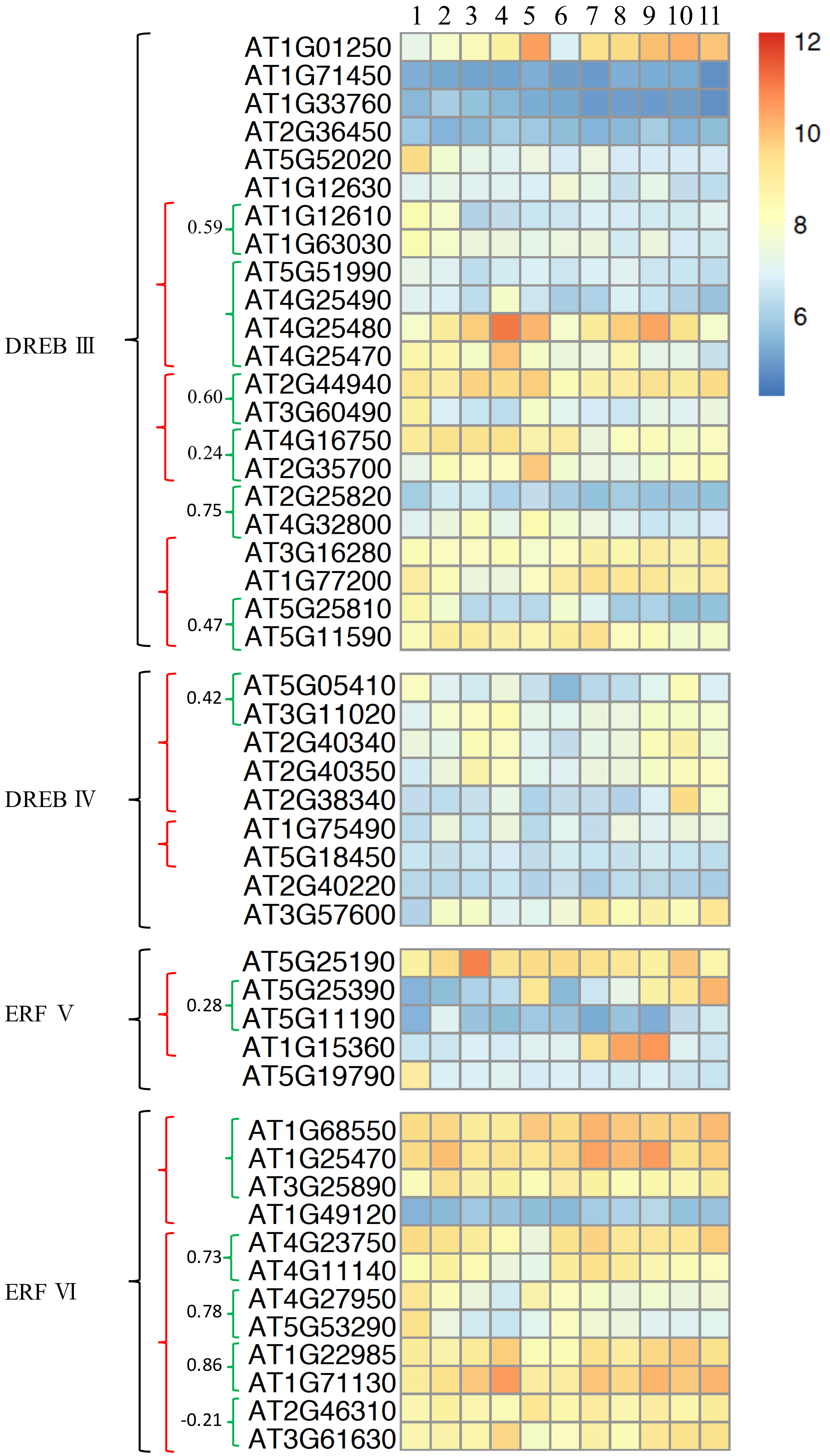

1 2 3 4 5 6 7 8 9 10 11
12
10
8
6
DREB Ⅲ
AT1G01250
AT1G71450
AT1G33760
AT2G36450
AT5G52020
AT1G12630
0.59
AT1G12610
AT1G63030
AT5G51990
AT4G25490
AT4G25480
AT4G25470
0.60
AT2G44940
AT3G60490
0.24
AT4G16750
AT2G35700
0.75
AT2G25820
AT4G32800
AT3G16280
AT1G77200
0.47
AT5G25810
AT5G11590
DREB Ⅳ
0.42
AT5G05410
AT3G11020
AT2G40340
AT2G40350
AT2G38340
AT1G75490
AT5G18450
AT2G40220
AT3G57600
ERF Ⅴ
AT5G25190
0.28
AT5G25390
AT5G11190
AT1G15360
AT5G19790
ERF Ⅵ
AT1G68550
AT1G25470
AT3G25890
AT1G49120
0.73
AT4G23750
AT4G11140
0.78
AT4G27950
AT5G53290
0.86
AT1G22985
AT1G71130
-0.21
AT2G46310
AT3G61630

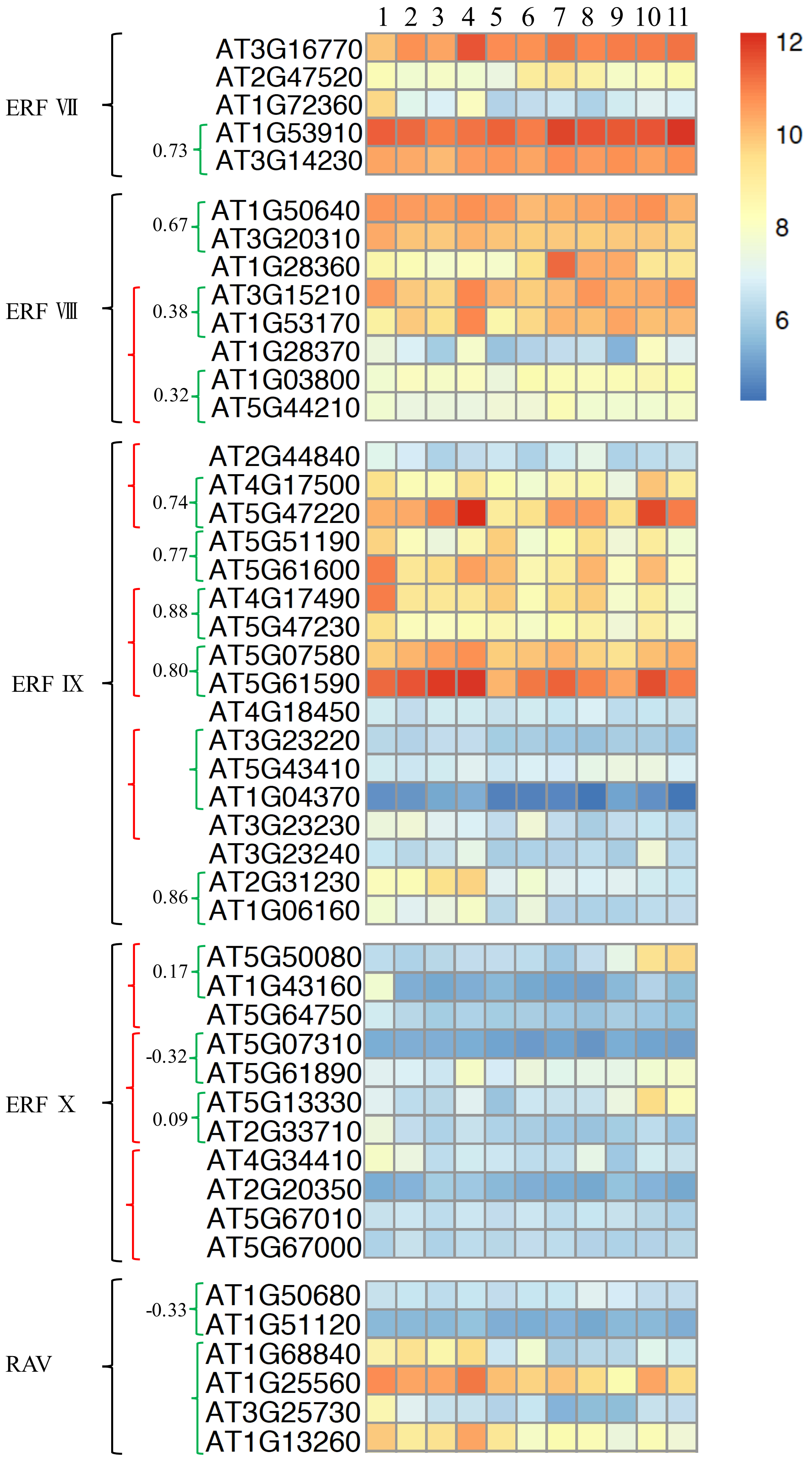

1 2 3 4 5 6 7 8 9 10 11
12
10
8
6
ERF Ⅶ
0.73
AT3G16770
AT2G47520
AT1G72360
AT1G53910
AT3G14230
ERF Ⅷ
0.67
0.38
0.32
AT1G50640
AT3G20310
AT1G28360
AT3G15210
AT1G53170
AT1G28370
AT1G03800
AT5G44210
ERF Ⅸ
0.74
0.77
0.88
0.80
0.86
AT2G44840
AT4G17500
AT5G47220
AT5G51190
AT5G61600
AT4G17490
AT5G47230
AT5G07580
AT5G61590
AT4G18450
AT3G23220
AT5G43410
AT1G04370
AT3G23230
AT3G23240
AT2G31230
AT1G06160
ERF Ⅹ
0.17
-0.32
0.09
AT5G50080
AT1G43160
AT5G64750
AT5G07310
AT5G61890
AT5G13330
AT2G33710
AT4G34410
AT2G20350
AT5G67010
AT5G67000
RAV
-0.33
AT1G50680
AT1G51120
AT1G68840
AT1G25560
AT3G25730
AT1G13260

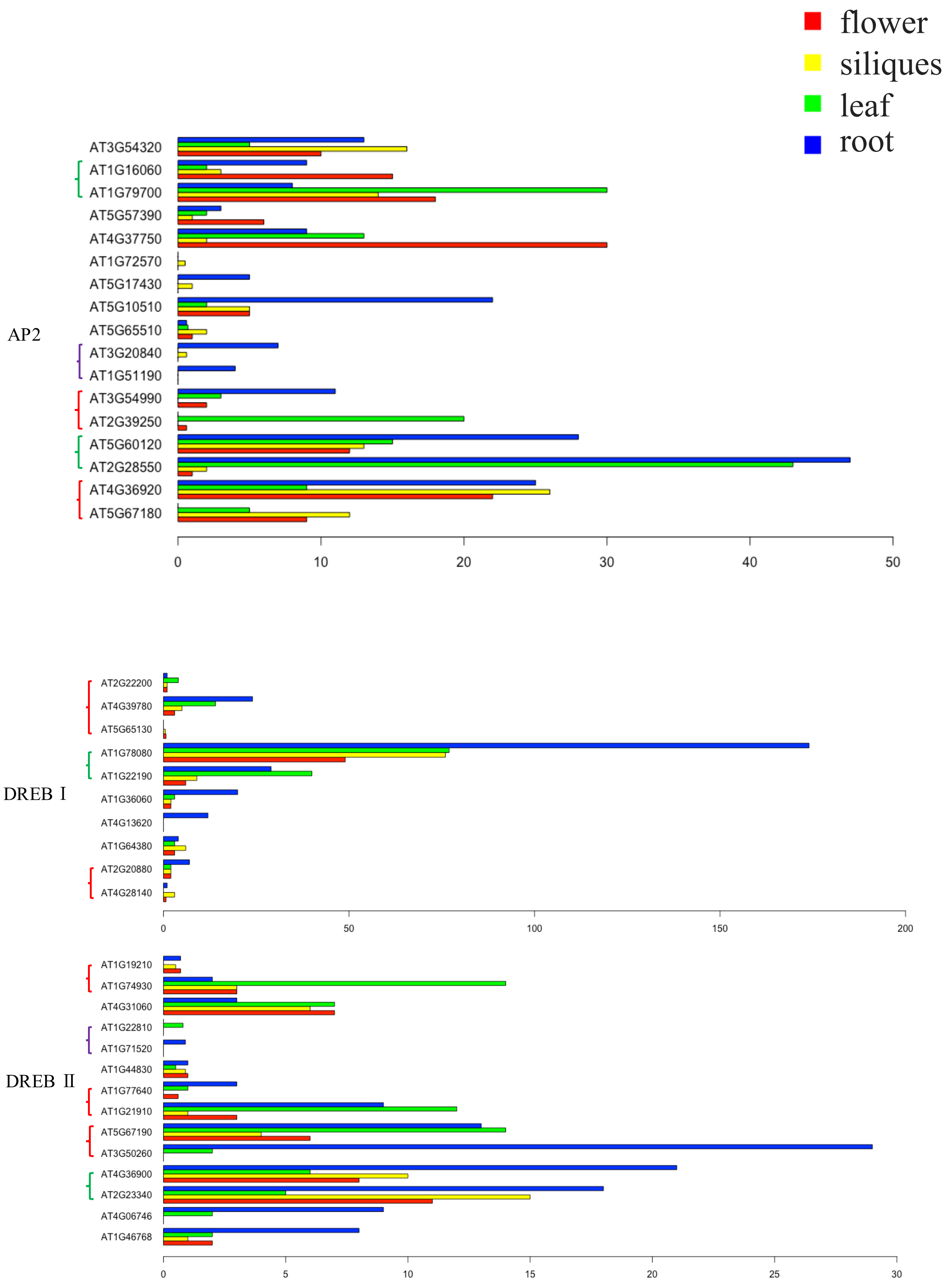

flower
siliques
leaf
root
AP2
AT3G54320
AT1G16060
AT1G79700
AT5G57390
AT4G37750
AT1G72570
AT5G17430
AT5G10510
AT5G65510
AT3G20840
AT1G51190
AT3G54990
AT2G39250
AT5G60120
AT2G28550
AT4G36920
AT5G67180
0
10
20
30
40
50
DREB Ⅰ
AT2G22200
AT4G39780
AT5G65130
AT1G78080
AT1G22190
AT1G36060
AT4G13620
AT1G64380
AT2G20880
AT4G28140
0
50
100
150
200
DREB Ⅱ
AT1G19210
AT1G74930
AT4G31060
AT1G22810
AT1G71520
AT1G44830
AT1G77640
AT1G21910
AT5G67190
AT3G50260
AT4G36900
AT2G23340
AT4G06746
AT1G46768
0
5
10
15
20
25
30

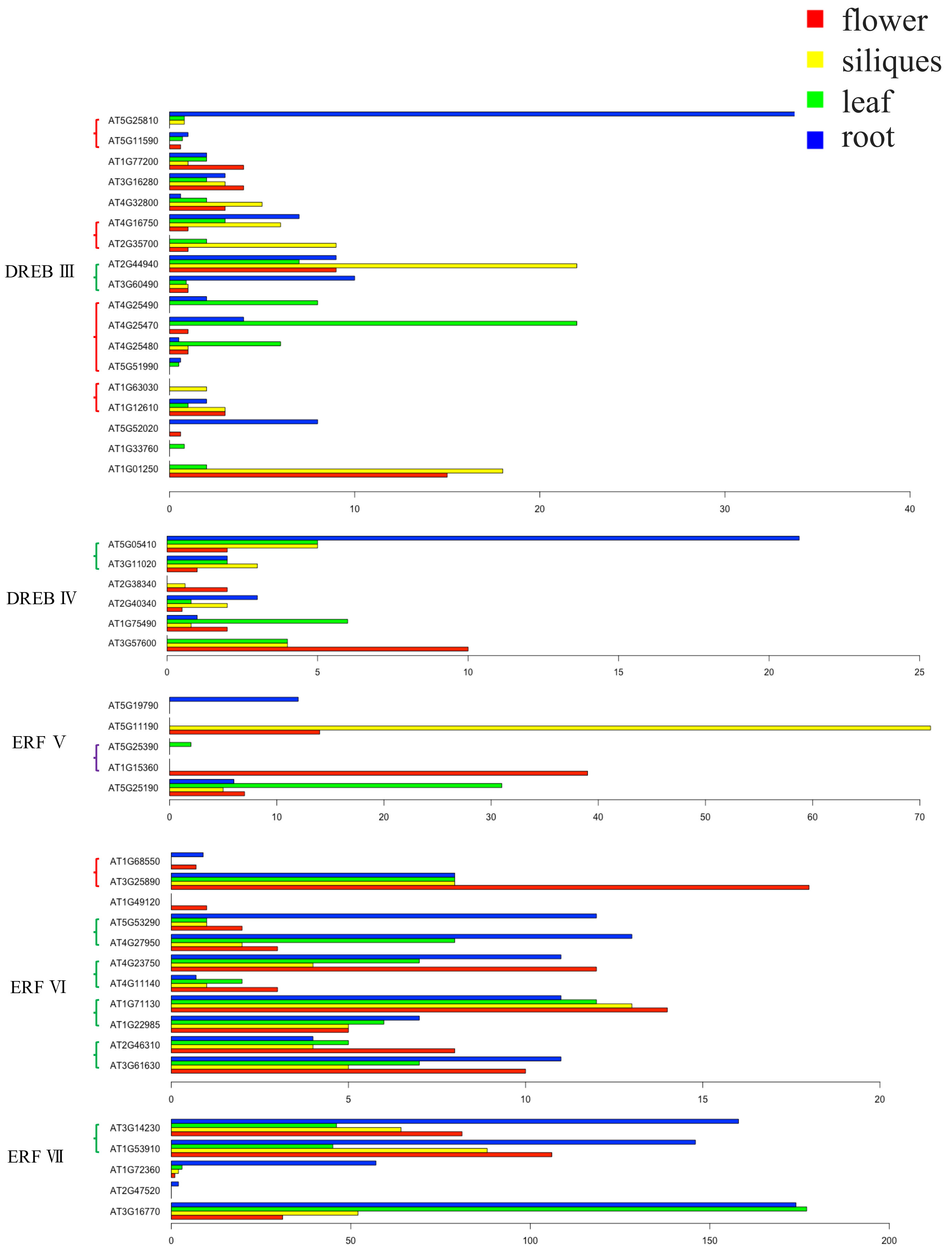
flower
siliques
leaf
root
DREB Ⅲ
AT5G25810
AT5G11590
AT1G77200
AT3G16280
AT4G32800
AT4G16750
AT2G35700
AT2G44940
AT3G60490
AT4G25490
AT4G25470
AT4G25480
AT5G51990
AT1G63030
AT1G12610
AT5G52020
AT1G33760
AT1G01250
0 10 20 30 40
DREB Ⅳ
AT5G05410
AT3G11020
AT2G38340
AT2G40340
AT1G75490
AT3G57600
0 5 10 15 20 25
ERF Ⅴ
AT5G19790
AT5G11190
AT5G25390
AT1G15360
AT5G25190
0 10 20 30 40 50 60 70
ERF Ⅵ
AT1G68550
AT3G25890
AT1G49120
AT5G53290
AT4G27950
AT4G23750
AT4G11140
AT1G71130
AT1G22985
AT2G46310
AT3G61630
0 5 10 15 20
ERF Ⅶ
AT3G14230
AT1G53910
AT1G72360
AT2G47520
AT3G16770
0 50 100 150 200

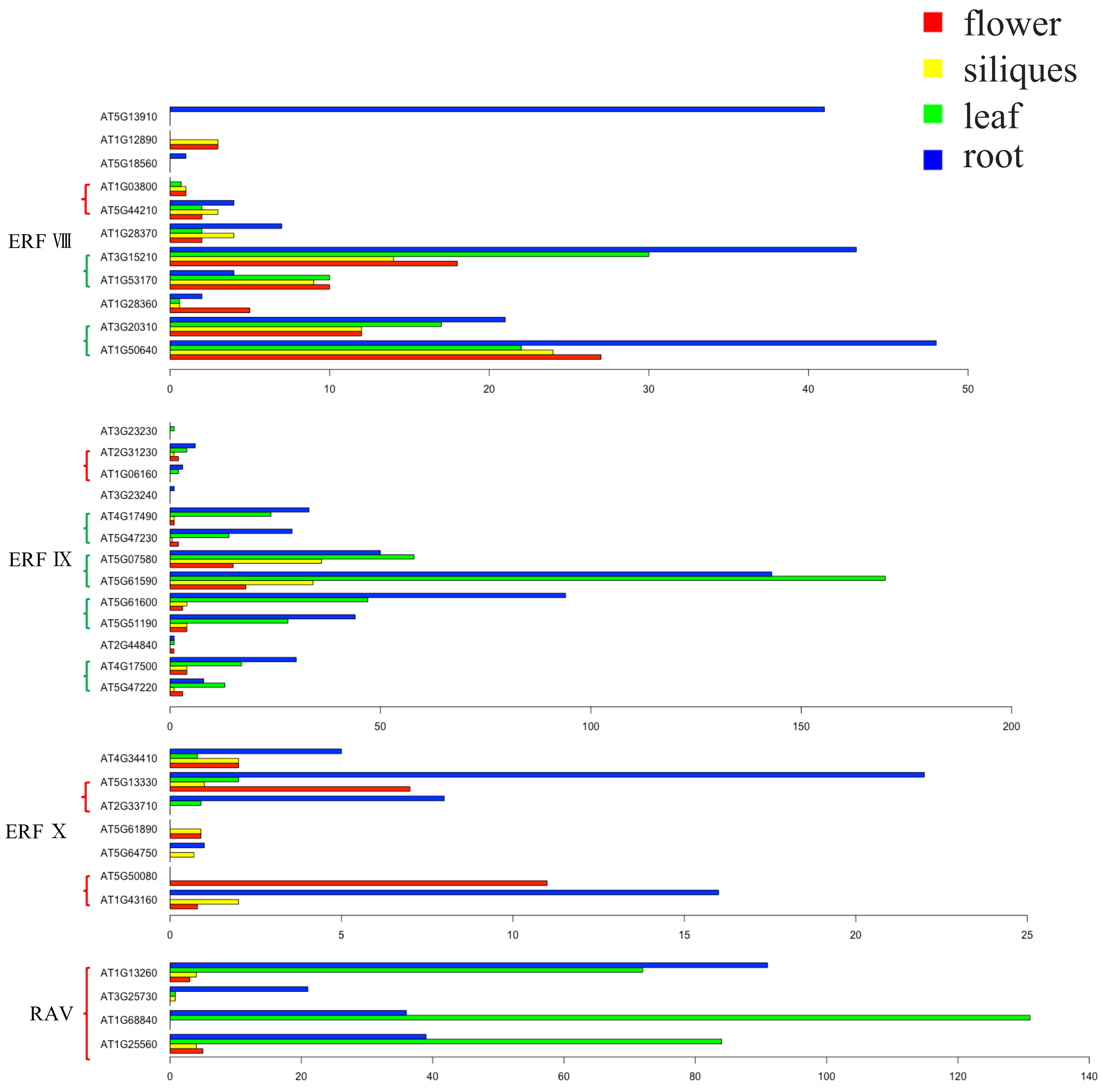
flower
siliques
leaf
root
ERF Ⅷ
AT5G13910
AT1G12890
AT5G18560
AT1G03800
AT5G44210
AT1G28370
AT3G15210
AT1G53170
AT1G28360
AT3G20310
AT1G50640
0
10
20
30
40
50
ERF Ⅸ
AT3G23230
AT2G31230
AT1G06160
AT3G23240
AT4G17490
AT5G47230
AT5G07580
AT5G61590
AT5G61600
AT5G51190
AT2G44840
AT4G17500
AT5G47220
0
50
100
150
200
ERF Ⅹ
AT4G34410
AT5G13330
AT2G33710
AT5G61890
AT5G64750
AT5G50080
AT1G43160
0
5
10
15
20
25
RAV
AT1G13260
AT3G25730
AT1G68840
AT1G25560
0
20
40
60
80
100
120
140

Figure S17. Wang et al.

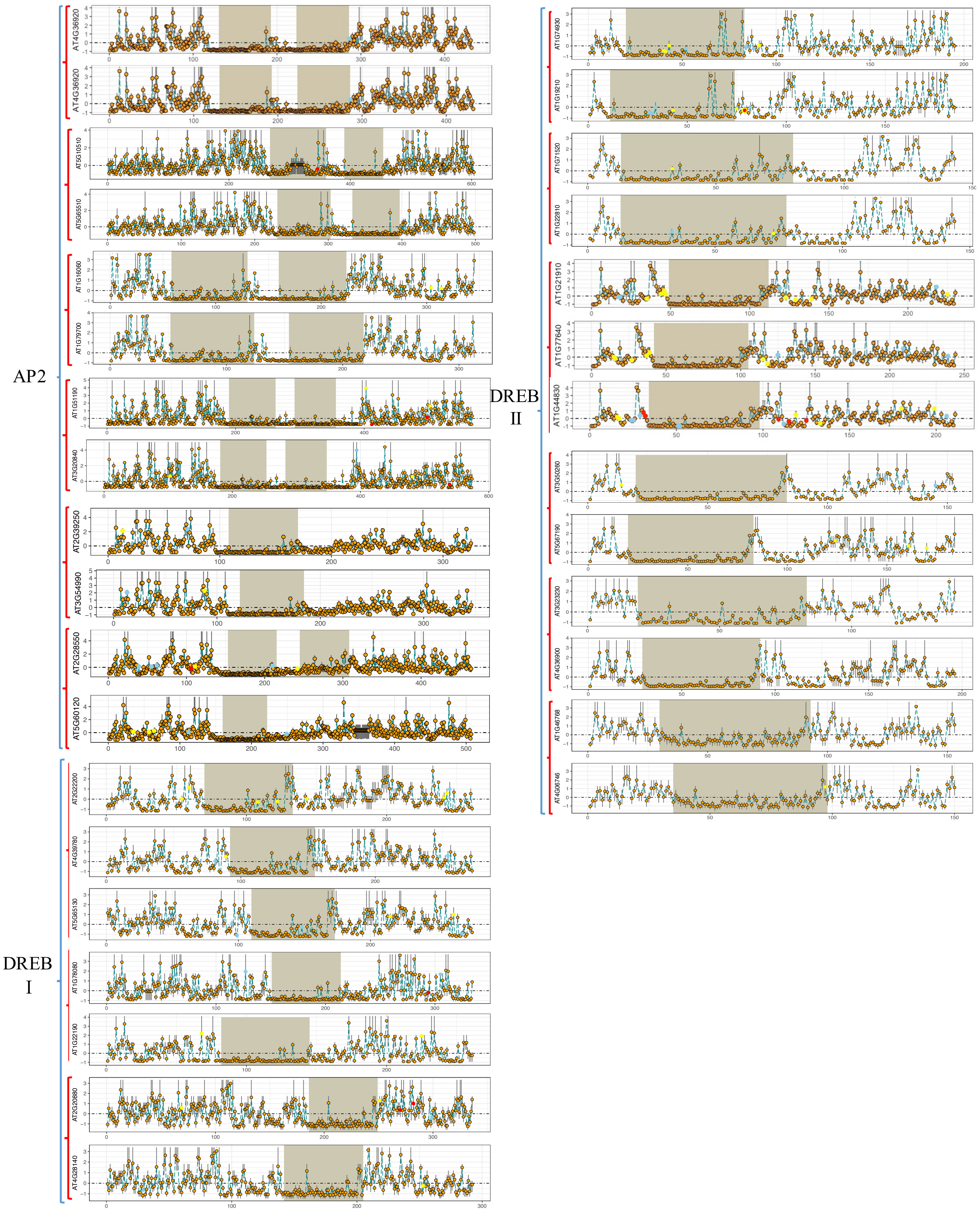

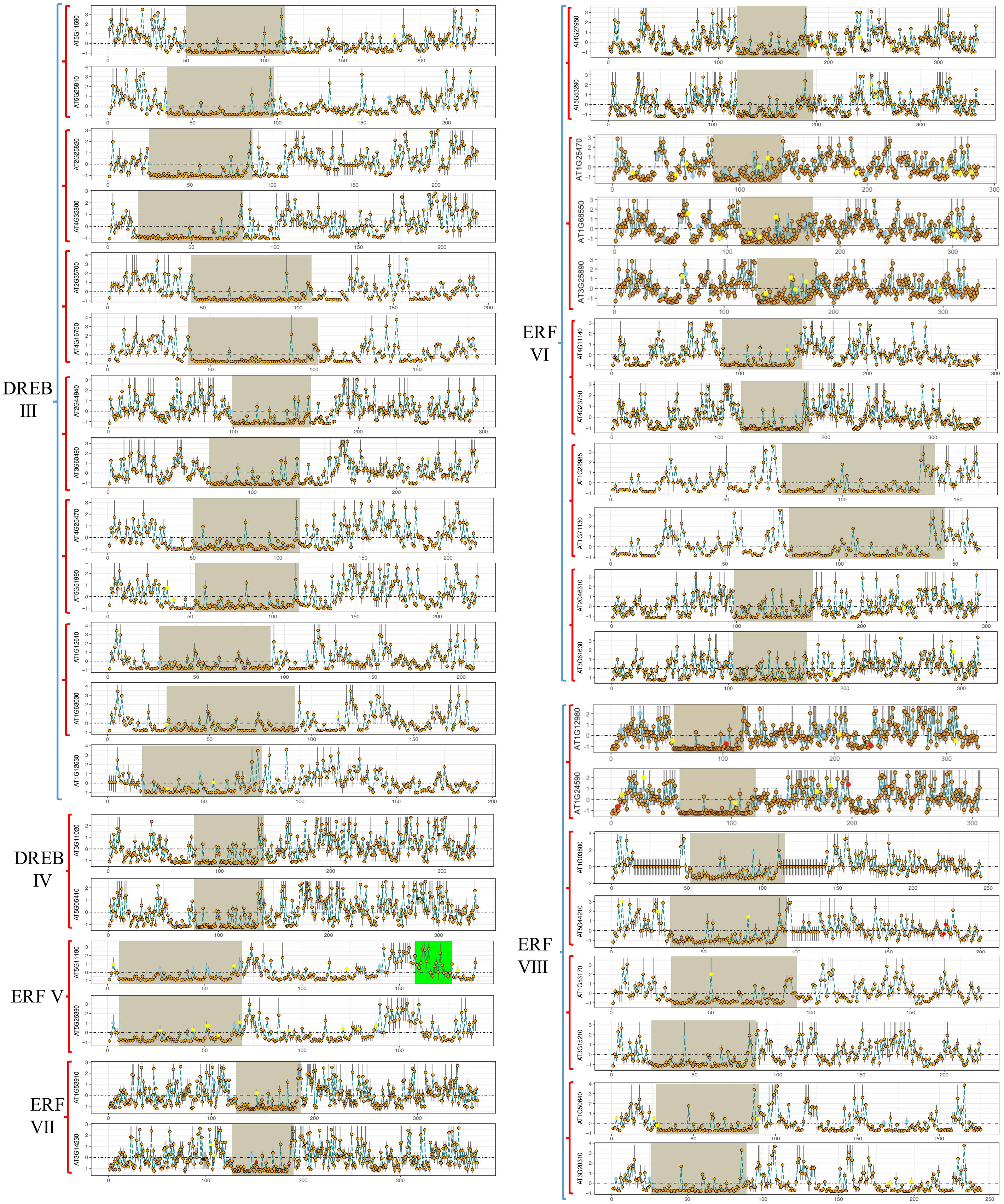

DREB III
AT5G11590
AT5G25810
AT2G25820
AT4G32800
AT2G35700
AT4G16750
AT2G44940
AT3G60490
AT4G25470
AT5G51990
AT1G12610
AT1G63030
AT1G12630
DREB IV
AT3G11020
AT5G05410
ERF V
AT5G11190
AT5G25390
ERF VII
AT1G53910
AT3G14230
ERF VI
AT4G27950
AT5G53290
AT1G25470
AT1G68550
AT3G25890
AT4G11140
AT4G23750
AT1G22985
AT1G71130
AT2G46310
AT3G61630
ERF VIII
AT1G12980
AT1G24590
AT1G03800
AT5G44210
AT1G53170
AT3G15210
AT1G50640
AT3G20310

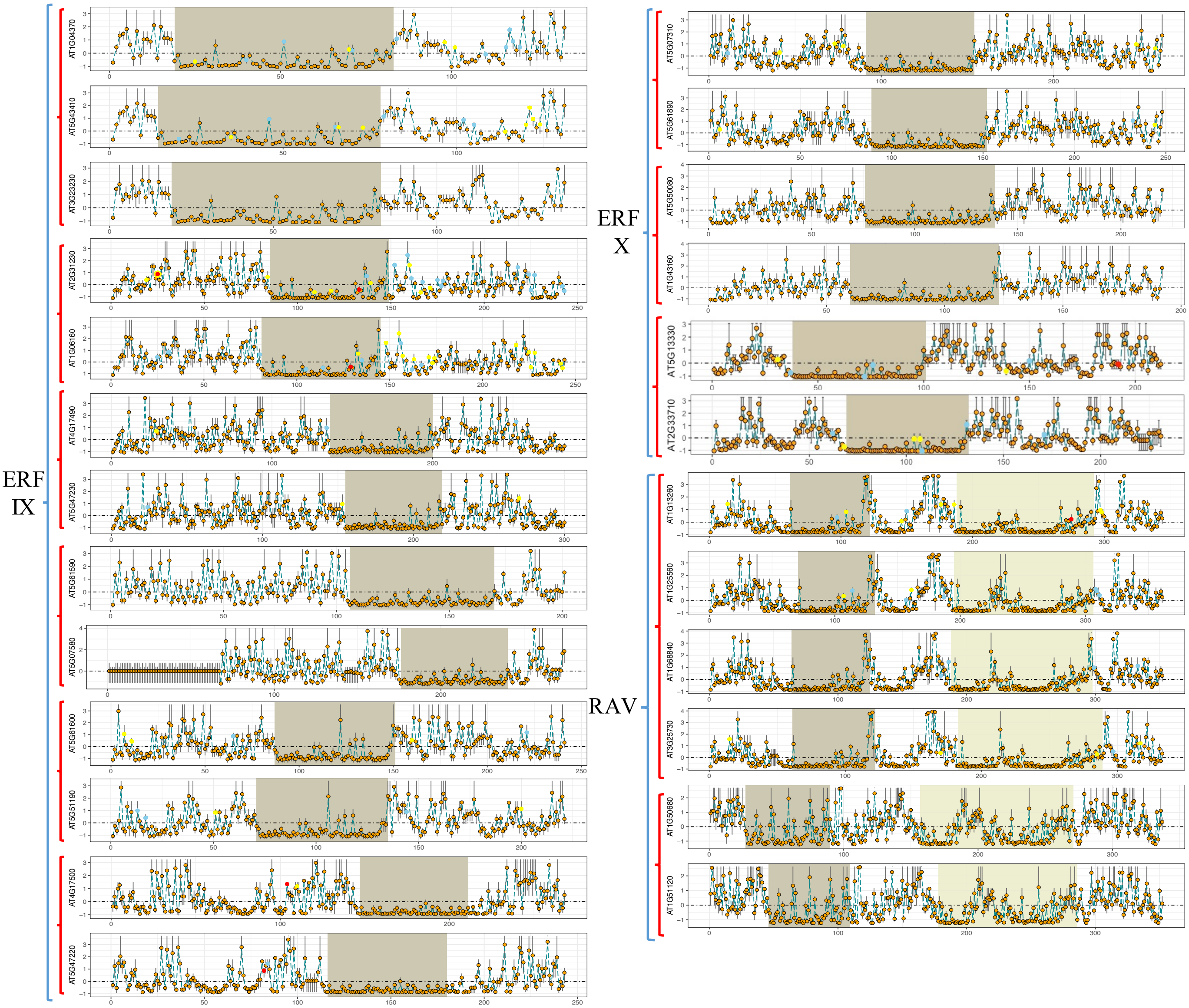

ERF IX
AT1G04370
AT5G43410
AT3G23230
AT2G31230
AT1G06160
AT4G17490
AT5G47230
AT5G61590
AT5G07580
AT5G61600
AT5G51190
AT4G17500
AT5G47220
ERF X
AT5G07310
AT5G61890
AT5G50080
AT1G43160
AT5G13330
AT2G33710
RAV
AT1G13260
AT1G25560
AT1G68840
AT3G25730
AT1G50680
AT1G51120

Figure S18. Wang et al.

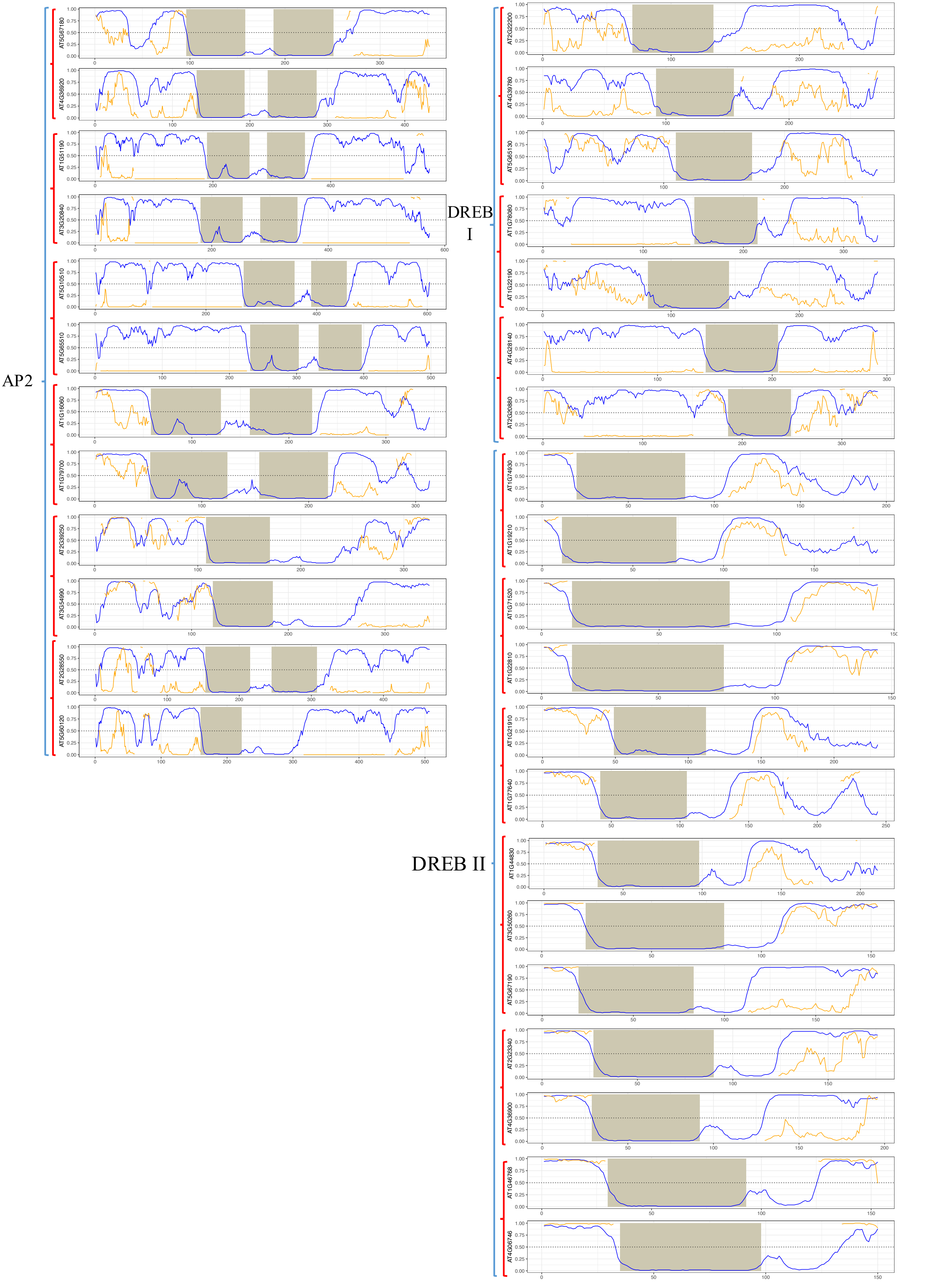

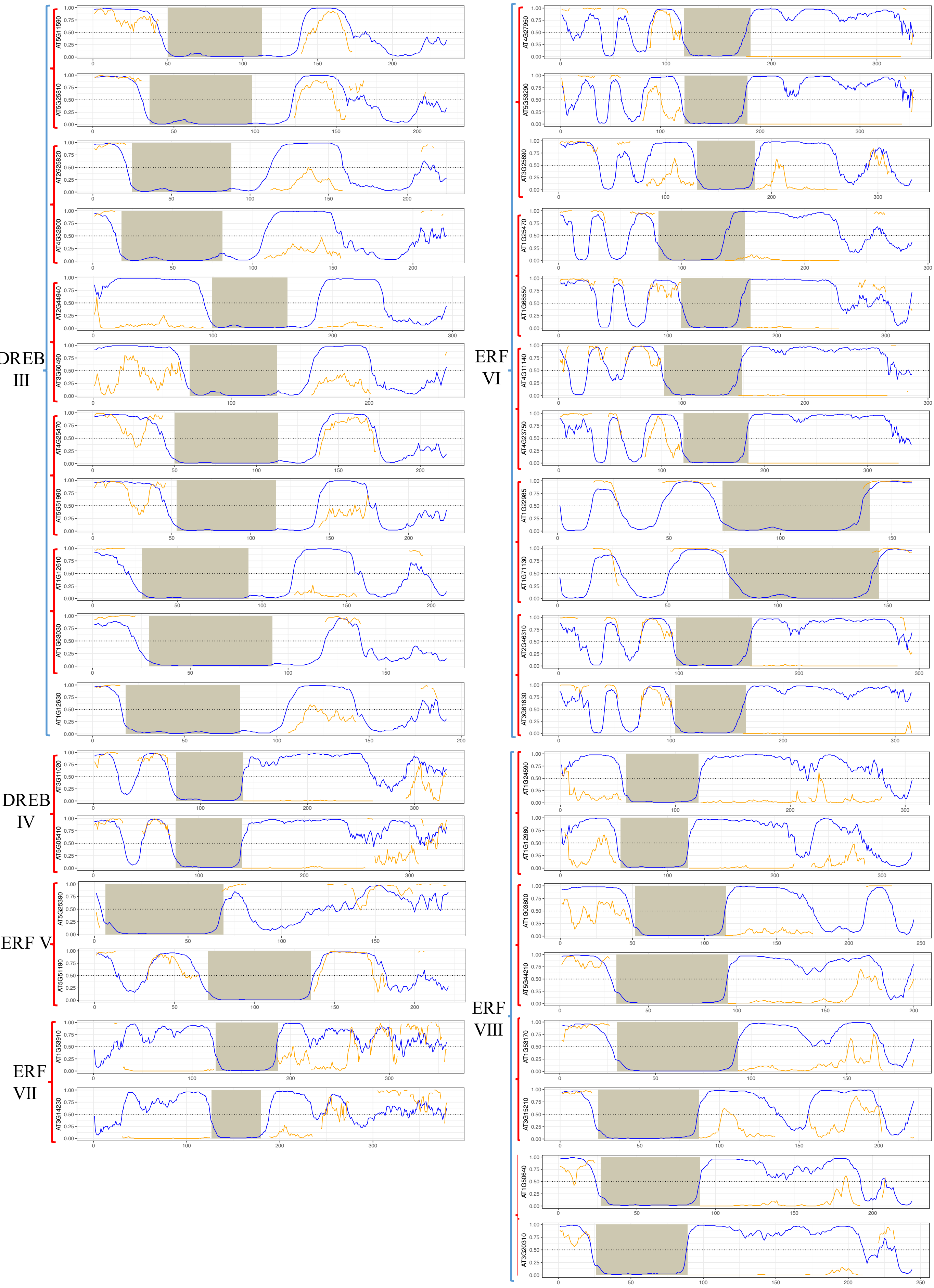
DREB III
AT5G11590
AT5G25810
AT2G25820
AT4G32800
AT2G44940
AT3G60490
AT4G25470
AT5G51990
AT1G12610
AT1G63030
AT1G12630
DREB IV
AT3G11020
AT5G05410
ERF V
AT5G25390
AT5G51190
ERF VII
AT1G53910
AT3G14230
ERF VI
AT4G27950
AT5G53290
AT3G25890
AT1G25470
AT1G68550
AT4G11140
AT4G23750
AT1G22985
AT1G71130
AT2G46310
AT3G61630
ERF VIII
AT1G24590
AT1G12980
AT1G03800
AT5G44210
AT1G53170
AT3G15210
AT1G50640
AT3G20310

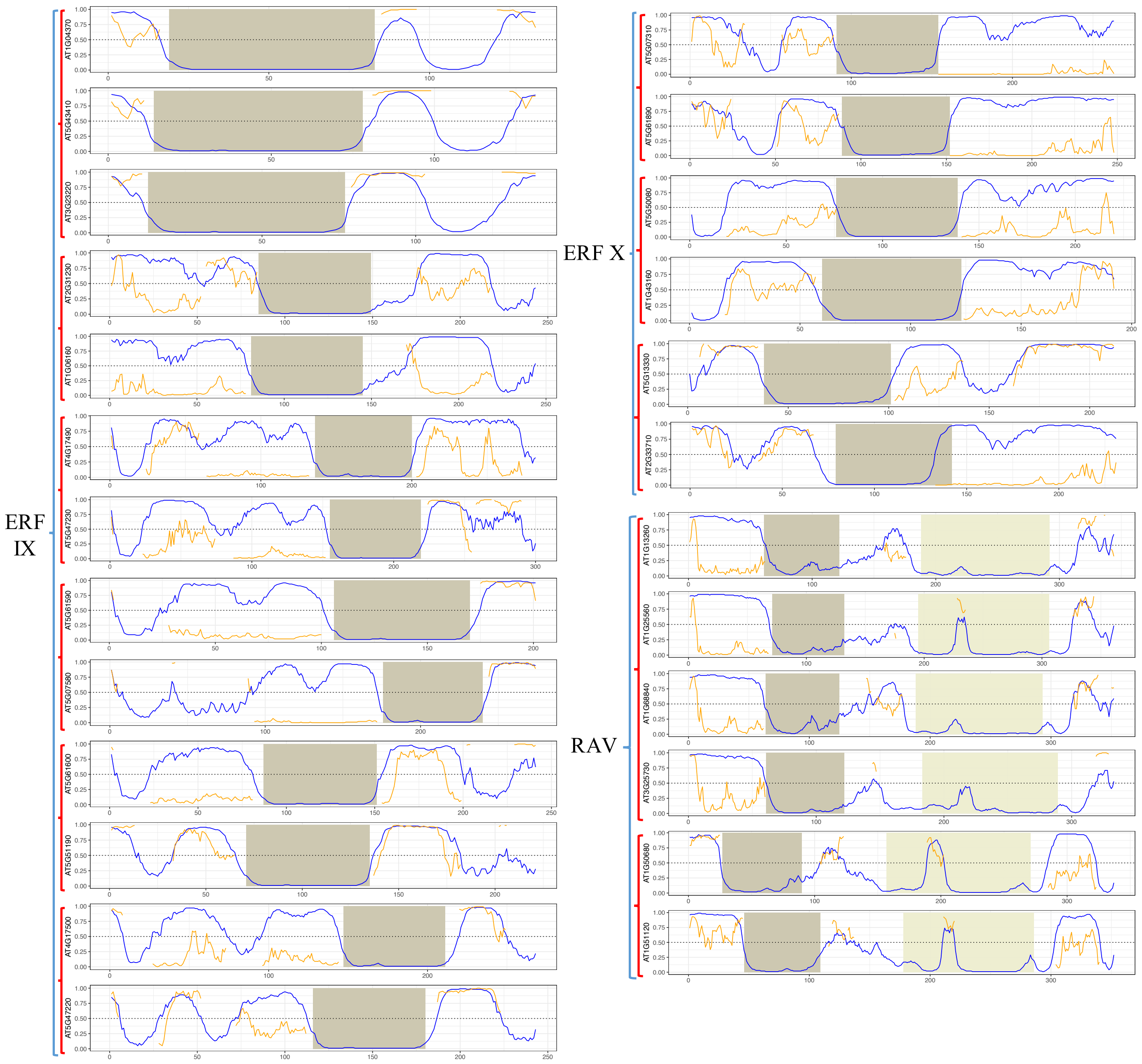
ERF IX
AT1G04370
AT5G43410
AT3G23220
AT2G31230
AT1G06160
AT4G17490
AT5G47230
AT5G61590
AT5G07580
AT5G61600
AT5G51190
AT4G17500
AT5G47220
ERF X
AT5G07310
AT5G61890
AT5G50080
AT1G43160
AT5G13330
AT2G33710
RAV
AT1G13260
AT1G25560
AT1G68840
AT3G25730
AT1G50680
AT1G51120

Figure S19. Wang et al

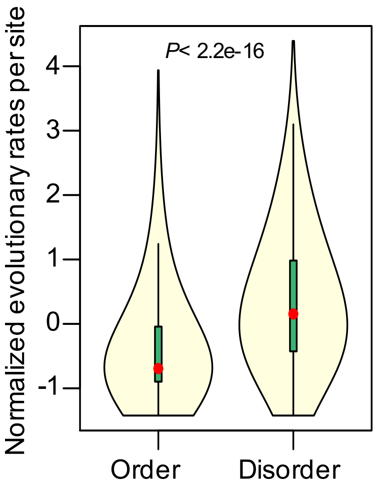

Figure S20. Wang et al.

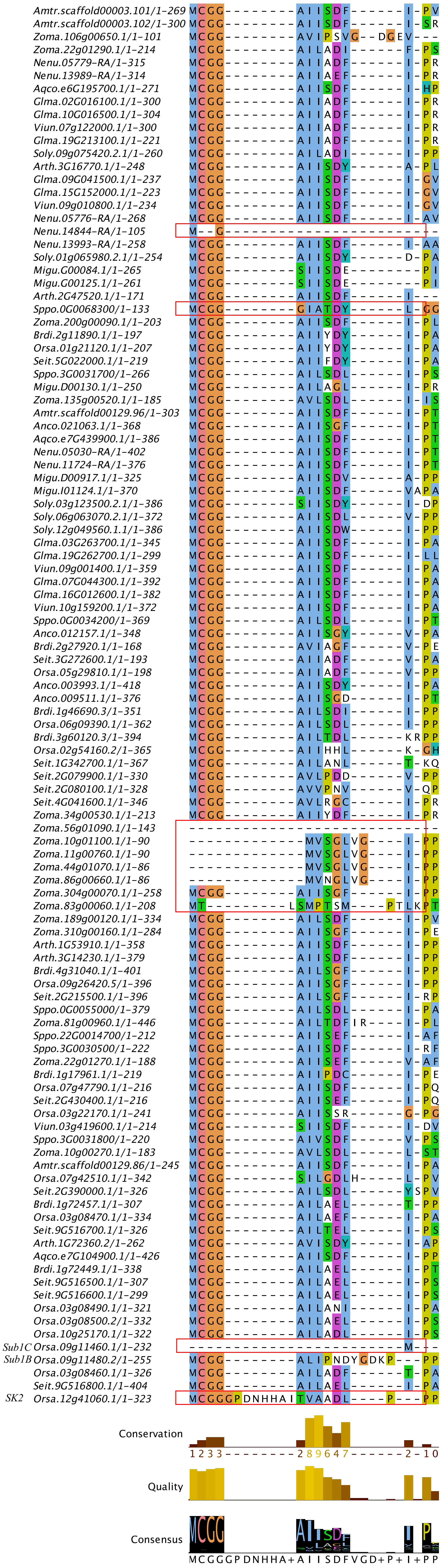

Supplement: Figure S1 — Phylogenetic trees of the AP2 subfamily from representative Angiosperms. SH-aLRT supports (approximate likelihood-ratio test and relies on a nonparametric, Shimodaira–Hasegawa–like procedure) above 50% are labeled on internal nodes. The MRCA (circles) and gene duplication events (stars) of each major plant group (Brassicaceae, Fabaceae, Asteraceae, core eudicots, Poaceae, Poales, Commelinids, Monocots, and Angiosperms) are indicated on internal nodes in different colors (green, light blue, purple, red, orange, deep blue, blue, deep red, and black). The numbers in the top left textbox represent the minimum inferred AP2/ERF protein complement and gene duplication events in the last common ancestor of each major plant group. [file Data_Sheet_1.PDF]
